# Causal association between 1400 metabolites and dilated cardiomyopathy: a bidirectional two-sample Mendelian randomization analysis

**DOI:** 10.3389/fendo.2024.1423142

**Published:** 2024-09-12

**Authors:** Xianghui Zeng, Qingfeng Zeng, Xianggui Wang, Kening Li, Jincheng Wu, Jianping Luo

**Affiliations:** ^1^ Department of Cardiology, Ganzhou Hospital of Traditional Chinese Medicine, Ganzhou, Jiangxi, China; ^2^ Department of Cardiology, Ganzhou People’s Hospital, Ganzhou, Jiangxi, China

**Keywords:** dilated cardiomyopathy, metabolites, Mendelian randomization, causal analysis, genetic variants, epidemiology

## Abstract

**Background:**

Dilated cardiomyopathy (DCM) is a cardiac disease with a poor prognosis of unclear etiology. Previous studies have shown that metabolism is associated with DCM. This study investigates the causal relationship between 1400 metabolites and DCM using a two-sample Mendelian randomization (MR) approach.

**Methods:**

The study utilized data from the OpenGWAS database, comprising 355,381 Europeans, including 1,444 DCM cases. A total of 1,400 metabolites were evaluated for their causal association with DCM. Instrumental variables (IVs) were selected based on genetic variation and used in the MR analysis. The primary analysis method was inverse variance weighting (IVW), supplemented by weighted median-based estimation and sensitivity analyses.

**Results:**

Of the 1,400 metabolites analyzed, 52 were identified as causally associated with DCM. The analysis revealed both positively and negatively correlated metabolites with DCM risk. Notable findings include the positive correlation of Tryptophan betaine and 5-methyluridine (ribothymidine) levels, and an inverse association of Myristoleate and Erythronate levels with DCM.

**Conclusions:**

The study provides significant insights into the metabolites potentially involved in the pathogenesis of DCM. These findings could pave the way for new therapeutic strategies and biomarker identification in DCM management.

## 1 Introduction

Dilated cardiomyopathy (DCM) is a serious cardiac disease, the pathogenesis of which is still unclear (1). DCM is mainly characterized by the dilation and impaired contraction of one or both ventricles. It is a common cause of heart failure and has a high mortality rate (2). The incidence of DCM is about 36 cases per 100,000 people. It is a common cause of heart failure and has a high mortality rate, with an incidence of about 36 cases per 100,000 people (3). The risk of DCM is 1.5-2 times higher in men than in women. DCM may occur in families, but most cases are sporadic. DCM can be caused by a variety of factors, including genetic factors, viral infections, autoimmune reactions, malnutrition, and alcoholism (4, 5). Some genetic variants are known to increase susceptibility to DCM, such as genes encoding proteins such as myosin and nuclear membrane proteins. Titin (TTN) is recognized as the major candidate gene for DCM, with mutations in TTN accounting for a significant proportion of familial and idiopathic cases (6), and LMNA, known for its role in laminopathies affecting cardiac function. Missense mutations in SCN5A have also been identified in DCM and carry a higher risk for arrythmias (7, 8). Mutations in RBM20 are responsible for 1–5% of genetic DCM (62) (9). These genetic abnormalities disrupt normal cardiac structure and function, exacerbating the effects of diabetes and leading to the development of DCM. Understanding these genetic contributions is essential for identifying at-risk individuals and developing targeted therapeutic strategies.

The development of metabolomics technology has provided new insights into the pathogenesis of DCM. Metabolomics can detect changes in metabolites in body fluids and tissues that reflect underlying biological processes (10). Recent metabolomics studies have identified altered serum and urinary metabolic profiles in patients with DCM. For example, serum levels of amino acids, carnitine, and creatine are elevated in patients with DCM, while levels of guanosine and inositol are reduced. This suggests that metabolic abnormalities may be involved in the pathogenesis of DCM. However, large-scale metabolomics studies have not systematically assessed the role of a wide range of metabolites in the pathogenesis of DCM (11). Clarifying the causal relationship between metabolites and the risk of DCM is crucial, as it could inform mechanistic studies and drug target discovery in DCM.

Current statistical methods for inferring causal relationships between metabolites and disease risk have limitations. Traditional observational studies are susceptible to confounding factors (12). In addition, it is difficult to distinguish whether metabolite changes are a consequence or a cause of DCM from observational data alone. To overcome these limitations, the present study adopted the Mendel randomization (MR) method, which uses genetic variation as an instrumental variable to assess the causal relationship between metabolites and the risk of DCM (13). The MR method utilizes the principle of random assignment of alleles inherited from parents to enhance the reliability of causal inference. It is less susceptible to confounding than observational studies. Therefore, the MR method provides more reliable causal hypothesis testing (14).

In this study, the causal associations between 1400 metabolites and DCM risk were assessed by a two-sample MR design using genetic and DCM data from the OpenGWAS database. The identified candidate causal metabolites may provide insights into the pathogenesis and therapeutic targets of DCM. More broadly, this study demonstrates the potential of MR methods in elucidating the metabolic mechanisms of complex diseases.

## 2 Materials and methods

### 2.1 Study design

Based on two-sample MR Analysis, we evaluated the causal relationship between 1400 metabolites and dilated cardiomyopathy. MR Uses genetic variation to represent risk factors, so valid instrumental variables (IVs) in causal reasoning must satisfy three key assumptions: (1) genetic variation is directly associated with exposure; (2) genetic variation is not associated with possible confounders between exposure and outcome; and (3) genetic variation does not affect outcome through pathways other than exposure (15, 16). This study used dilated cardiomyopathy data from the OpenGWAS, which included 355381 Europeans, including 1444 cases and 353937 as the control group.

### 2.2 Metabolite GWAS data sources

Aggregate GWAS statistics for each metabolite are publicly available from the GWAS catalog (registration numbers from GCST90199621 to GCST90201020). This is a large-scale GWAS study that includes 1091 metabolites and 309 metabolite ratios from 8299 individuals in the Canadian Longitudinal Aging Study (CLSA) cohort (17, 18).

### 2.3 Selection of instrumental variables

Since genetic variation is directly related to exposure, the significance level of IVs for each metabolite was set at 1×10^−5^, the significance level of IVs for each metabolite was set at 1×10^−5^. To obtain IVs for independent sites, we used the “Two Sample MR” packet data with a linkage unbalance (LD) threshold set to R^2^<0.001 and an aggregation distance of 10,000 kb (19, 20). For dilated cardiomyopathy, we adjusted the significance level to 5×10^−6^, which is commonly used to represent genome-wide significance in GWAS, with a LD threshold of R^2^<0.001 and an aggregation distance of 10,000 kb (21).

### 2.4 Statistical analysis

**Figure 1:**
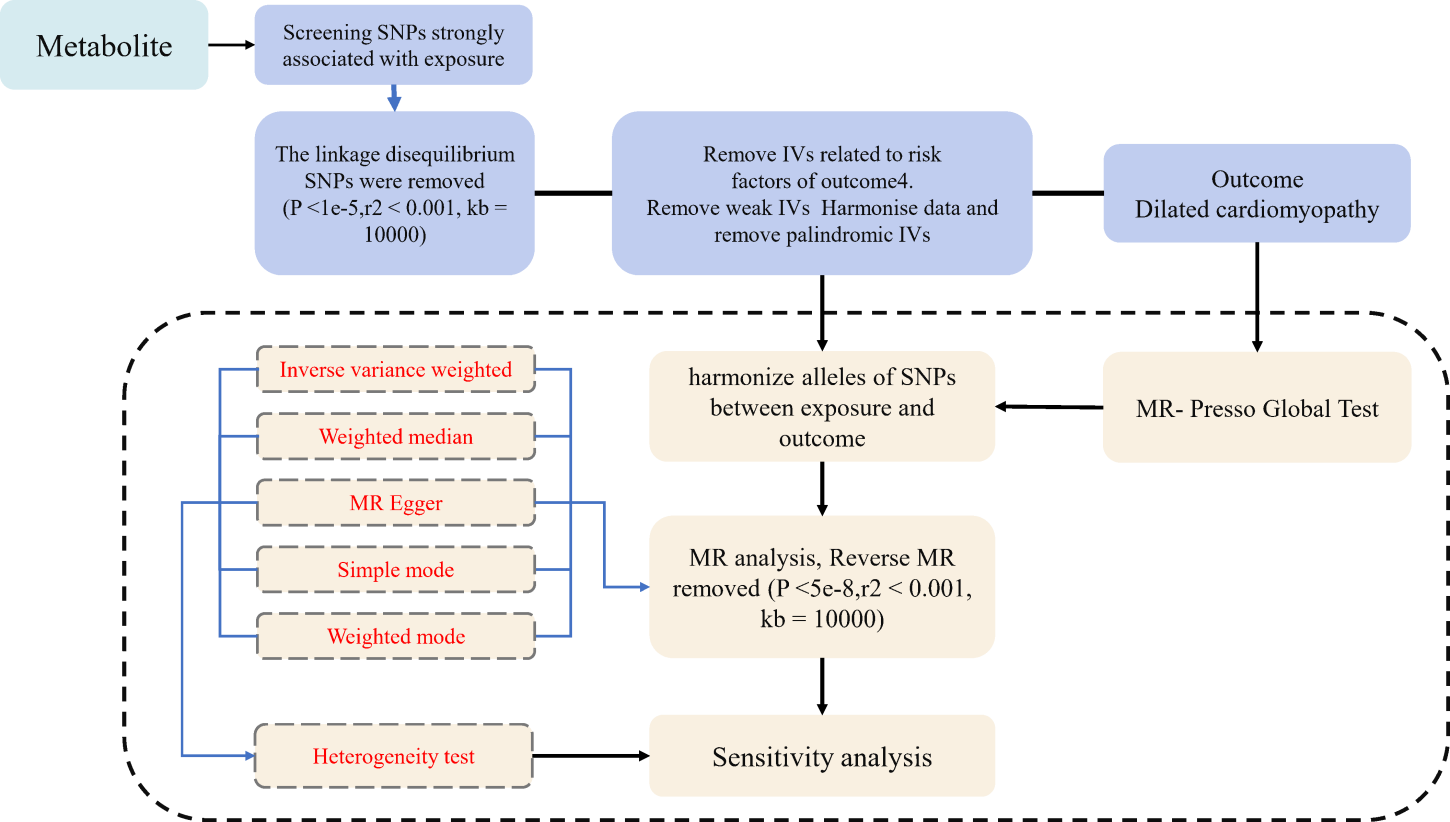
Flow diagram for quality control of the instrumental variables (IVs) and the entire Mendelian Randomization (MR) analysis process. SNPs, single-nucleotide polymorphisms; IVW, inverse variance weighted; MR, Mendelian Randomization; MR Presso, Mendelian Randomization Pleiotropy RESidual Sum and Outlier.

For the statistical analysis portion of our study examining the causal influence of metabolite on dilated cardiomyopathy risk, all procedures were conducted using R software, version 4.2.1, which is a widely used environment for statistical computing and graphics, available at (http://www.Rproject.org). To ascertain the causal relationships between the 1400 metabolite and dilated cardiomyopathy, we primarily employed methods including inverse variance weighting (IVW), weighted median-based estimation. These analyses were facilitated by the ‘TwoSampleMR’ package, version 0.5.7, within the R software environment. This package is specifically designed for conducting MR analyses, providing tools for estimation, testing, and sensitivity analysis of causal effects (22). The IVW method is a standard approach in MR that combines the Wald estimates (ratio of the SNP-outcome association to the SNP-exposure association) from multiple genetic variants, weighting by the inverse variance of each SNP-outcome association. The weighted median and mode-based methods serve as supplementary approaches that provide robust causal estimates even when some of the instrumental variables are invalid, as long as certain assumptions are met. These analyses were backed up by rigorous sensitivity analyses, including Cochran’s Q test to examine heterogeneity amongst the instrumental variables (23). Such thorough statistical evaluation ensures that the findings regarding the relationship between metabolite and dilated cardiomyopathy are as reliable and accurate as possible given the data. The whole process was shown in **Figure 1**.

## 3 Results

### 3.1 Exploration of the causal effect of metabolite on dilated cardiomyopathy risk

**Figure 2:**
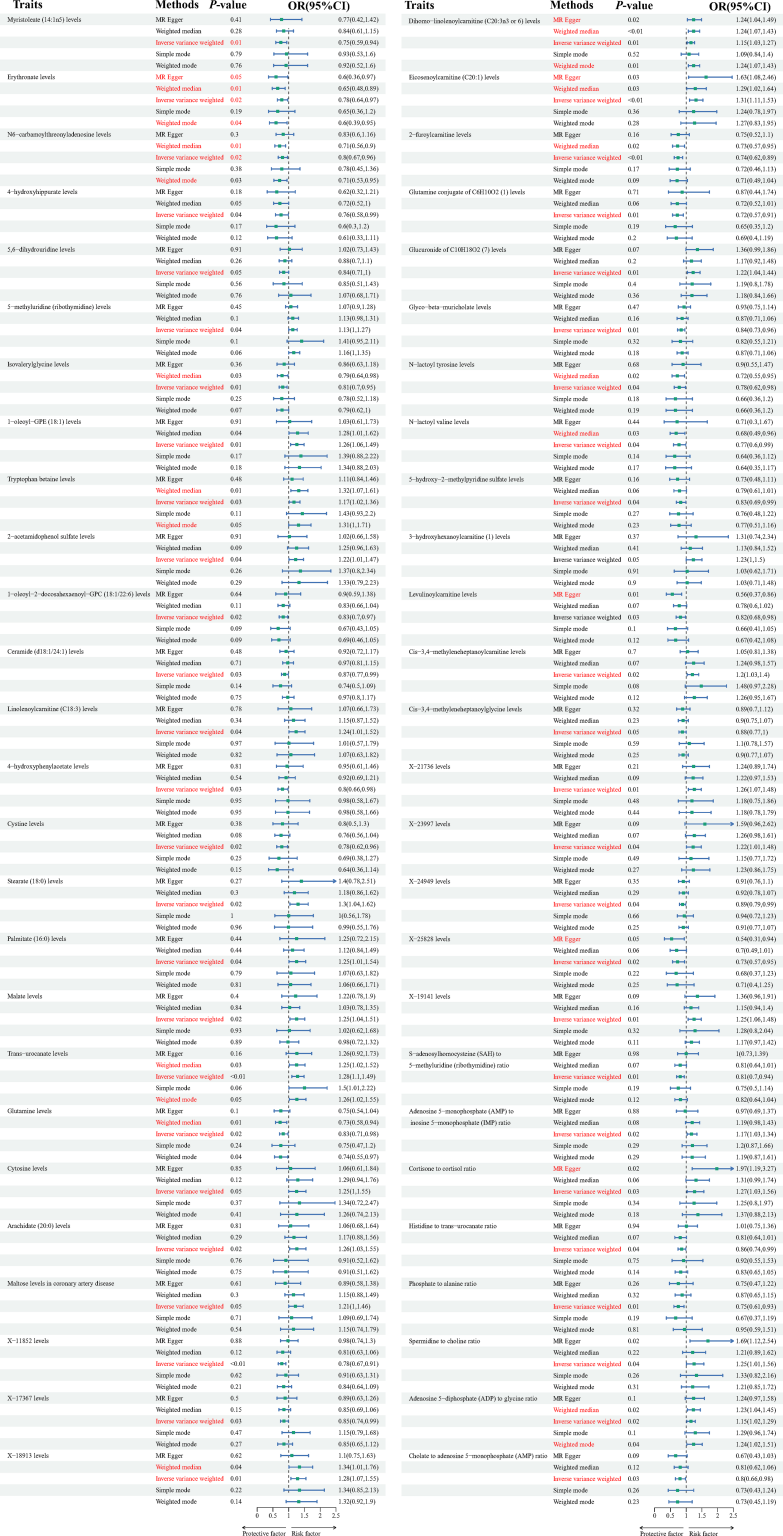
Forest plots depicting the causal associations between dilated cardiomyopathy and specific metabolites. IVW, inverse variance weighting; CI, confidence interval.

At the significance level of 0.05, a total of 52 metabolite were identified as causally associated with the development of dilated cardiomyopathy (as shown in **Figure 2**).

The Tryptophan betaine levels(P=0.029,OR=1.174, 95%CI =1.016~1.356),5-methyluridine (ribothymidine) levels (P=0.042, OR=1.130, 95%CI =1.004~1.271), 2-acetamidophenol sulfate levels (P=0.039, OR=1.219, 95%CI =1.009~1.473), Linolenoylcarnitine (C18:3) levels(P=0.041, OR=1.238, 95%CI =1.008~1.520), Dihomo-linolenoylcarnitine (C20:3n3 or 6) levels (P=0.009,OR=1.146, 95%CI =1.034~1.271), Glucuronide of C10H18O2 (7) levels (P=0.014,OR=1.223, 95%CI =1.040~1.438), Eicosenoylcarnitine (C20:1) levels (P=0.001, OR=1.305, 95%CI =1.110~1.534), 3-hydroxyhexanoylcarnitine (1) levels(P=0.047,OR=1.225, 95%CI =1.002~1.496), Cis-3,4-methyleneheptanoylcarnitine levels (P=0.016, OR=1.203, 95%CI =1.034~1.399), Stearate (18:0) levels (P=0.022,OR=1.297, 95%CI =1.037~1.623), Palmitate (16:0) levels (P=0.038,OR=1.246, 95%CI =1.011~1.535), Malate levels (P=0.017, OR=1.253, 95%CI =1.040~1.511), Trans-urocanate levels (P=0.001,OR=1.282, 95%CI =1.101~1.492), Cytosine levels (P=0.047,OR=1.248, 95%CI =1.002~1.554), Arachidate (20:0) levels (P=0.023,OR=1.262, 95%CI =1.031~1.545), Maltose levels in coronary artery disease (P=0.045,OR=1.208, 95%CI =1.003~1.455), X-18913 levels (P=0.007, OR=1.284, 95%CI =1.067~1.545), X-21736 levels (P=0.005,OR=1.255, 95%CI =1.067~1.476), X-23997 levels (P=0.038,OR=1.224, 95%CI =1.011~1.483),X-19141 levels (P=0.009, OR=1.249, 95%CI =1.055~1.478), Adenosine 5’-monophosphate (AMP) to inosine 5’-monophosphate (IMP) ratio (P=0.017,OR=1.173, 95%CI =1.027~1.339), Cortisone to cortisol ratio (P=0.025,OR=1.269, 95%CI =1.030~1.564), Spermidine to choline ratio(P=0.042, OR=1.254, 95%CI =1.007~1.562), Adenosine 5’-diphosphate (ADP) to glycine ratio (P=0.020, OR=1.149, 95%CI =1.021~1.293),1-oleoyl-GPE (18:1) levels (P=0.008,OR=1.258, 95%CI =1.061~1.493)are positively correlated with dilated cardiomyopathy.

While Myristoleate (14:1n5) levels (P=0.012,OR=0.746, 95%CI =0.592~0.940), Erythronate levels (P=0.021,OR=0.783, 95%CI =0.635~0.965), N6-carbamoylthreonyladenosine levels (P=0.018,OR=0.804, 95%CI =0.671~0.964), 4-hydroxyhippurate levels (P=0.039, OR=0.755, 95%CI =0.577~0.987), 5,6-dihydrouridine levels (P=0.047,OR=0.840, 95%CI =0.707~0.998), Isovalerylglycine levels (P=0.009, OR=0.812, 95%CI =0.695~0.950), 1-oleoyl-2-docosahexaenoyl-GPC (18:1/22:6) levels (P=0.021, OR=0.827, 95%CI =0.704~0.972), Ceramide (d18:1/24:1) levels (P=0.029,OR=0.871, 95%CI =0.769~0.986), Glutamine conjugate of C6H10O2 (1) levels (P=0.005,OR=0.717, 95%CI =0.567~0.906), 2-furoylcarnitine levels(P=0.001,OR=0.742, 95%CI =0.618~0.890), Glyco-beta-muricholate levels (P=0.010,OR=0.835, 95%CI =0.728~0.957), N-lactoyl tyrosine levels (P=0.035,OR=0.783, 95%CI =0.623~0.984), N-lactoyl valine levels (P=0.040,OR=0.768, 95%CI =0.596~0.988), 5-hydroxy-2-methylpyridine sulfate levels (P=0.038,OR=0.825, 95%CI =0.688~0.990), Levulinoylcarnitine levels (P=0.027, OR=0.818, 95%CI =0.684~0.978), Cis-3,4-methyleneheptanoylglycine levels (P=0.046, OR=0.879, 95%CI =0.774~0.998), 4-hydroxyphenylacetate levels (P=0.033, OR=0.802, 95%CI =0.655~0.982), Cystine levels (P=0.021,OR=0.775, 95%CI =0.624~0.962), Glutamine levels (P=0.023, OR=0.832, 95%CI =0.710~0.976), X-11852 levels (P=0.001,OR=0.780, 95%CI =0.667~0.912),X-17367 levels (P=0.033,OR=0.854, 95%CI =0.739~0.987),X-24949 levels(P=0.040,OR=0.887, 95%CI =0.790~0.994),X-25828 levels (P=0.019,OR=0.734, 95%CI =0.567~0.952),S-adenosylhomocysteine (SAH) to 5-methyluridine (ribothymidine) ratio (P=0.006,OR=0.813, 95%CI =0.701~0.944), Histidine to trans-urocanate ratio (P=0.039,OR=0.856, 95%CI =0.739~0.992), Phosphate to alanine ratio(P=0.008,OR=0.750, 95%CI =0.606~0.928), Cholate to adenosine 5’-monophosphate (AMP) ratio(P=0.029,OR=10,802, 95%CI =0.658~0.977) are inversely associated with dilated cardiomyopathy. Results from sensitivity analyses demonstrate the robustness of the observed causal association (**Supplementary Figure 1**). Scatter plot and funnel plot also show the stability of the results (**Supplementary Figures 2****,** **3**).

### 3.2 Exploration of the causal effect of dilated cardiomyopathy risk on metabolite

To investigate the causal relationship between dilated cardiomyopathy and metabolites, a two-sample Mendelian randomization (MR) analysis was employed, with the Inverse Variance Weighting (IVW) method as the primary analytical approach and other methods serving as supplementary. Subsequently, reverse MR was used to explore the impact of dilated cardiomyopathy onset on the aforementioned 50 metabolites. The results showed that there was no causal relationship between dilated cardiomyopathy and the 52 aforementioned diseases.

## 4 Discussion

In this study, we performed a comprehensive Mendelian randomization analysis to assess causal associations between circulating metabolites and dilated cardiomyopathy (DCM) risk. Our analysis identified 52 metabolites across multiple classes exhibiting significant causal relationships with DCM. These results provide unique insights into the metabolic pathways that may be involved in DCM development and progression. More broadly, this work highlights the utility of MR methods in elucidating complex disease mechanisms.

We found metabolites from diverse biochemical families, including amino acids, lipids, microbial metabolites, nucleotides, and carbohydrates, that demonstrated causal impacts on DCM risk. Both positive and inverse associations were observed, indicating potential pathogenic and protective effects of various metabolites. Several of the identified compounds have known links to cardiac physiology or dysfunction, further supporting their causal implications in DCM revealed by this analysis.

For instance, we found that higher levels of tryptophan betaine were causally associated with increased DCM risk. Tryptophan betaine, also known as ergothioneine, is a dietary phytochemical and antioxidant. While its cardioprotective properties have been previously described, our findings suggest it may exert adverse effects in DCM (24). Consistent with our conclusions, one recent reports supporting the association of typtophan betaine with heart failure (25). This compound is transported into tissues via organic cation transporters, whose expression and activity are altered in cardiovascular disease (26). The resultant accumulation of tryptophan betaine could potentially disturb redox homeostasis or cation homeostasis in ways that promote cardiomyopathy. Additional work is warranted to clarify the mechanisms underlying its causal relationship with DCM observed here.

Among the lipids, myristoleate was found to be negatively associated with DCM risk, indicating a potential protective effect. Myristoleate is an omega-5 unsaturated fatty acid that can act as a bioactive lipid mediator. In mice, it was shown to attenuate cardiac dysfunction and remodeling following experimental myocardial infarction (27). Myristoleate supplementation also improved left ventricular performance in a rat model of ischemic cardiomyopathy (28). Our results provide orthogonal population-level evidence that higher myristoleate levels may preserve cardiac structure and function, thereby lowering susceptibility to DCM. The cytoprotective effects are thought to derive from its capacity to resolve macrophage inflammation.

Another notable finding was the positive causal association between ribothymidine levels and increased DCM risk. Ribothymidine is a modified nucleoside found in transfer RNA. Though its functional roles are incompletely defined, it may be involved in mediating cellular responses to stress. Our results further indicate this nucleoside associates with and may promote cardiomyopathic changes. Altered ribothymidine metabolism could perturb RNA biology in ways that undermine myocardial viability.

We also found multiple microbial metabolites exhibiting causal links with DCM, including p-cresol sulfate, phenyllactate, and imidazole propionate. This aligns with the recognized role of the gut microbiome and its products in shaping cardiovascular health (29). For example, p-cresol sulfate, generated by gut bacterial fermentation, positively associated with major adverse cardiovascular events in chronic kidney disease patients (30). The absorptive transport of microbial metabolites and their effects on factors like vasoreactivity, inflammation, and redox balance may thus be an important conduit through which the microbiota influences DCM development.

Overall, our findings nominate a number of metabolites across diverse classes that may be involved in DCM pathogenesis through both novel and established mechanisms. By leveraging genetic anchors for improved causal inference, this study moves beyond simply observing metabolite associations to provide evidence these molecules may directly modulate DCM susceptibility. Additional research is warranted to elucidate their underlying molecular mechanisms in cardiac pathogenesis.

Notably, our MR results revealed several metabolites exhibiting inverse causal relationships with DCM, including glutamine, erythronate, glycocholate, and cystine. Lower levels of these compounds associated with higher DCM risk, suggesting potential cardioprotective effects. Glutamine supports myocardial metabolism and function, particularly under stressed conditions (31). Its depletion could starve cardiac myocytes of a vital substrate. Erythronate is involved in carnitine synthesis, which is essential for fatty acid oxidation. Diminished erythronate could thereby reduce energy production. Finally, bile acids like glycocholate possess signaling activities affecting diverse physiological processes (32, 33). Their reduction may remove beneficial signaling that helps maintain cardiac performance. These protective metabolites or their related pathways warrant further evaluation as therapeutic targets or biomarkers in DCM.

A major strength of our study was the use of two-sample MR leveraging data from large GWAS datasets. This enabled interrogation of a broad panel of metabolites for causal effects on DCM in a sample size sufficiently powered to detect modest effects for this rare disease. Furthermore, the extensive panel of genetic instruments from metabolomics GWAS enhanced specificity for assessing metabolites. Application of multiple MR methods and sensitivity analyses ensured robust causal estimates. Overall, MR overcomes limitations of conventional observational studies for assessing metabolite-disease relationships.

However, some limitations should be considered when interpreting the findings. The MR results require confirmation through experimental models given the constraints of population-level analyses. The study population was predominantly of European ancestry, warranting caution in extrapolating conclusions to other ethnic groups (34). Only metabolites with available GWAS data could be evaluated, providing an incomplete metabolomic portrait. Additionally, we were unable to account for potential effects of DCM medications on circulating metabolites. Nevertheless, this work provides a significant advance in evaluating metabolite causal effects in DCM free of confounds using genetic anchors. Furthermore, MR offers substantial insights into causal relationships, yet it has inherent limitations. One key issue is the potential for pleiotropy, where genetic variants influence multiple traits, which can confound causal inferences. To address this, we have employ methods such as multivariable MR or MR-Egger regression, which help to detect and adjust for pleiotropic effects. the reliance on large sample sizes may limit applicability in smaller or underrepresented populations. To mitigate this, future studies could integrate data from biobanks or consortiums, thereby enhancing power and ensuring diversity. Measurement error in exposures or outcomes can further complicate interpretations. Utilizing more accurate phenotyping methods and conducting sensitivity analyses can help strengthen findings. Lastly, while MR suggests causation, it often lacks insight into underlying mechanisms. Incorporating complementary approaches, such as bioinformatics analyses or functional studies, can provide a more comprehensive understanding of the relationships under investigation. By acknowledging these limitations and implementing rigorous methodologies, the robustness of MR findings can be significantly improved, enhancing its utility in epidemiological research.

## 5 Conclusion

The findings have several important clinical implications. First, delineation of causal metabolites enhances our mechanistic understanding of DCM etiopathogenesis. The identified metabolic pathways could be leveraged for development of prognostic biomarkers, which are lacking for DCM. For example, panels incorporating multiple MR-supported metabolites may enable earlier DCM diagnosis or risk stratification. Second, the results reveal novel targets for potential pharmacologic intervention. Therapeutic normalization of harmful metabolites like tryptophan betaine or elevation of beneficial metabolites like glutamine could ameliorate DCM severity. Nutritional or microbiome-based approaches to modulate implicated metabolites may also hold promise. Overall, the findings pave the way for metabolite-centered precision prevention and treatment strategies. Finally, this work highlights the broader utility of MR for dissecting complex disease mechanisms. Two-sample MR with large GWAS datasets is a powerful tool for probing cause-effect relationships, free of confounds affecting conventional observational studies. Our study demonstrates the promise of applying this technique to map causal metabolic pathways involved in cardiovascular diseases. Integrating MR with multi-omics data represents an emerging frontier to unravel disease pathogenesis. Elucidating causal molecular networks through genetic instrumental variable approaches will be critical for guiding development of targeted diagnostics and therapies. As GWAS data continues expanding, MR applications leveraging massive sample sizes for precise etiologic insights will become increasingly feasible and impactful.

## Data Availability

The original contributions presented in the study are included in the article/**Supplementary Material**. Further inquiries can be directed to the corresponding author.
