# Supplementary figures and images for: Causal association between 1400 metabolites and dilated cardiomyopathy: a bidirectional two-sample Mendelian randomization analysis
Source: Front Endocrinol (Lausanne). 2024 Sep 12;15:1423142. doi: 10.3389/fendo.2024.1423142 (PMC11424463; doi:10.3389/fendo.2024.1423142)

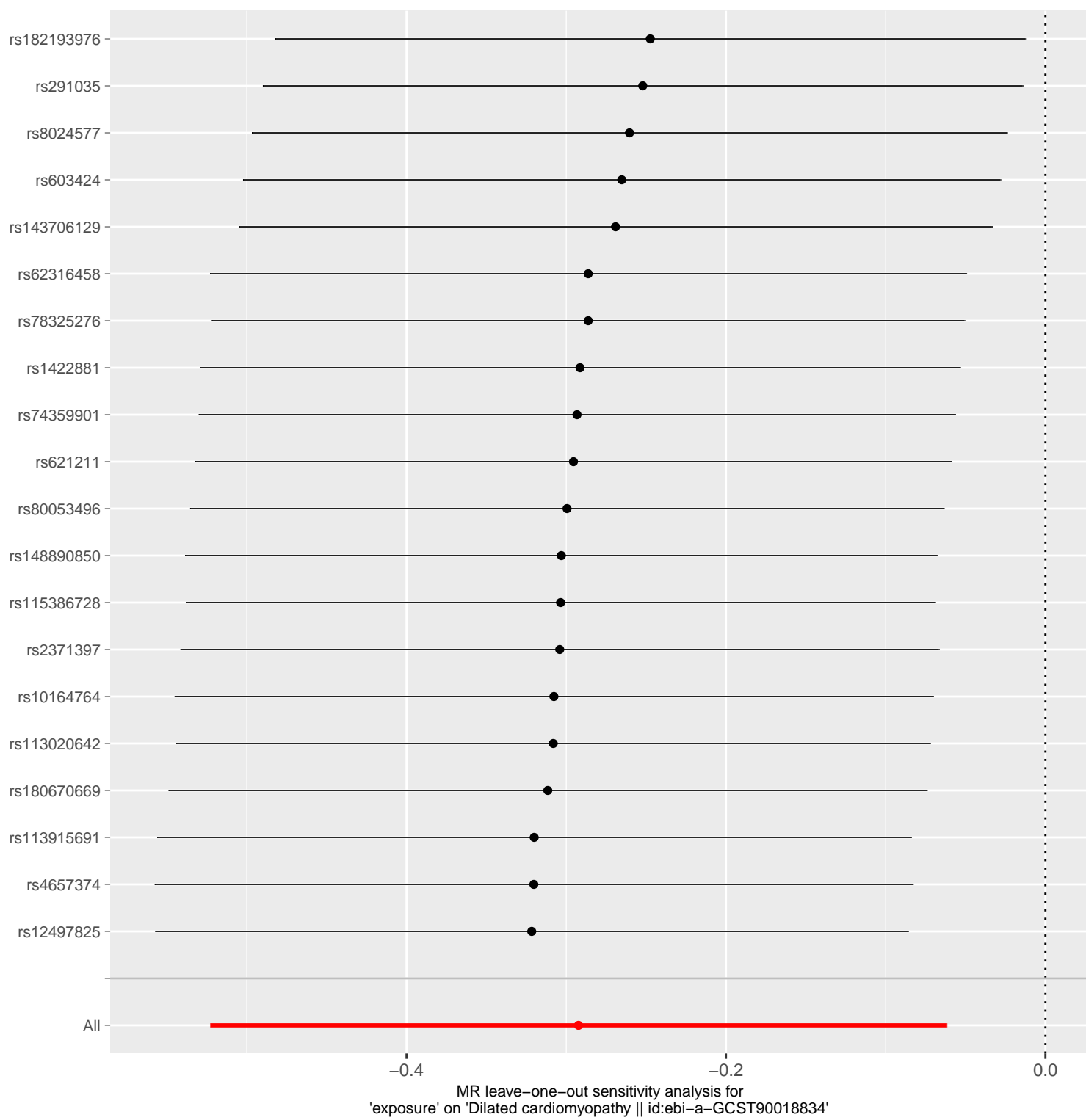

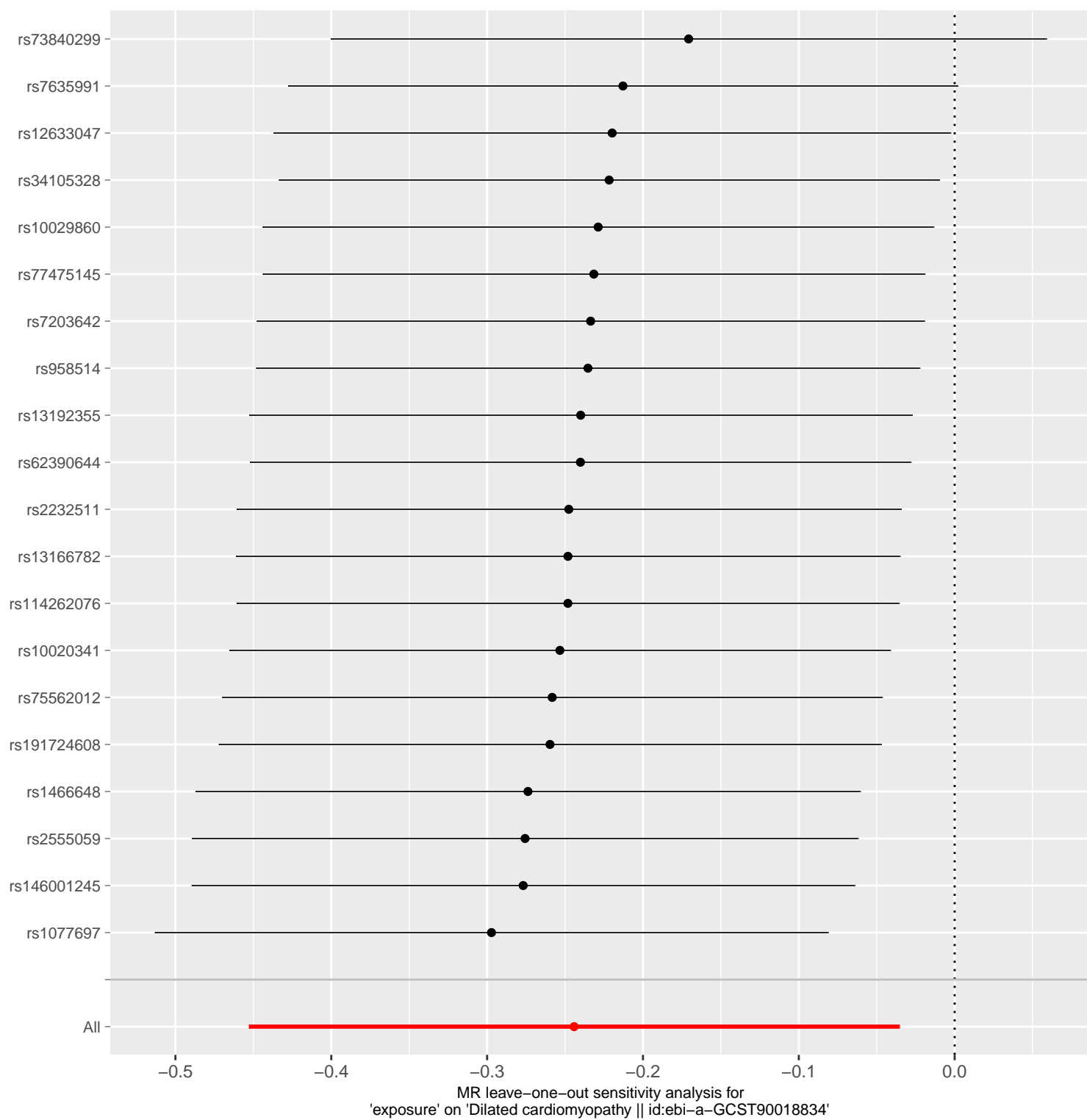

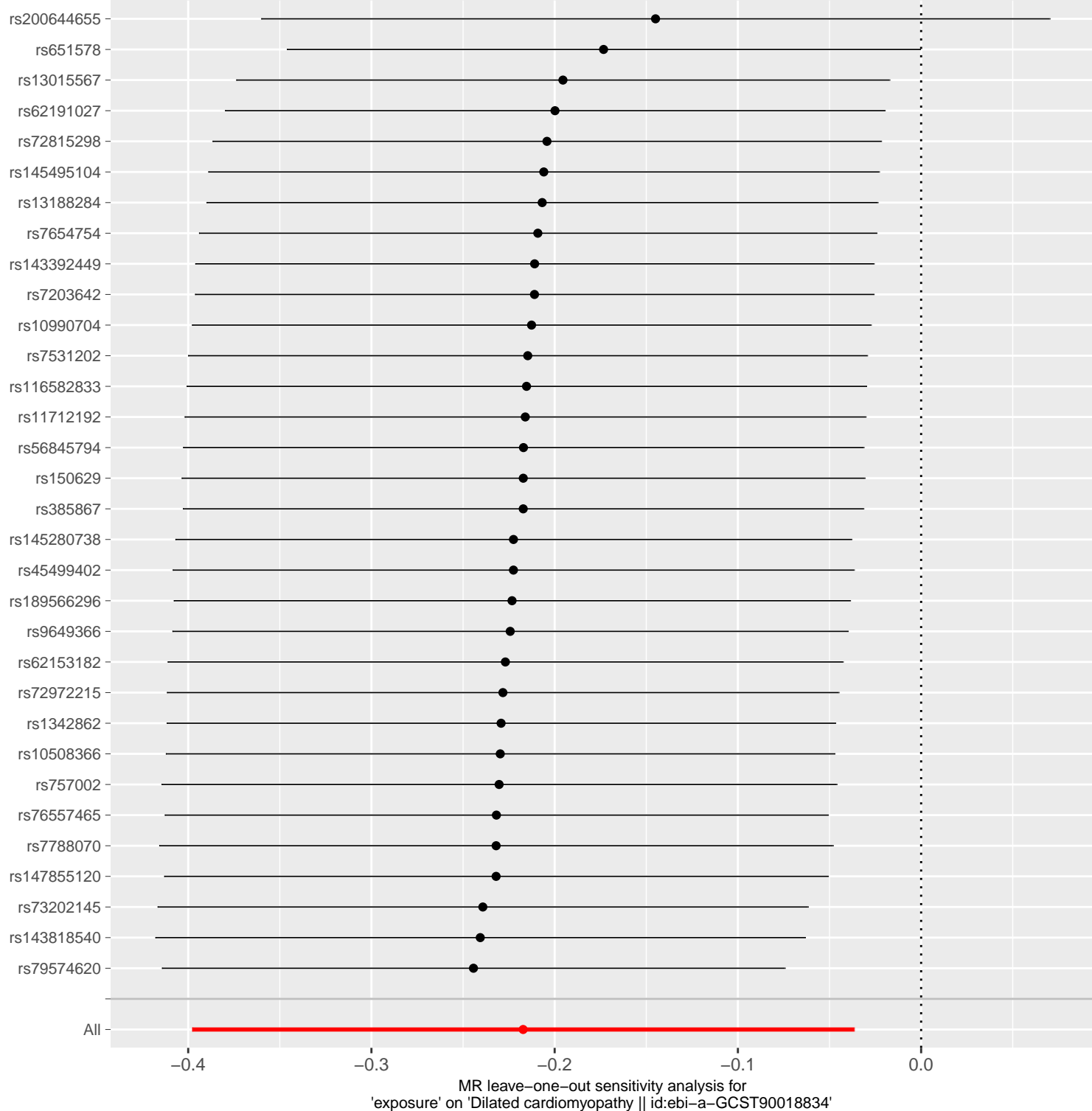

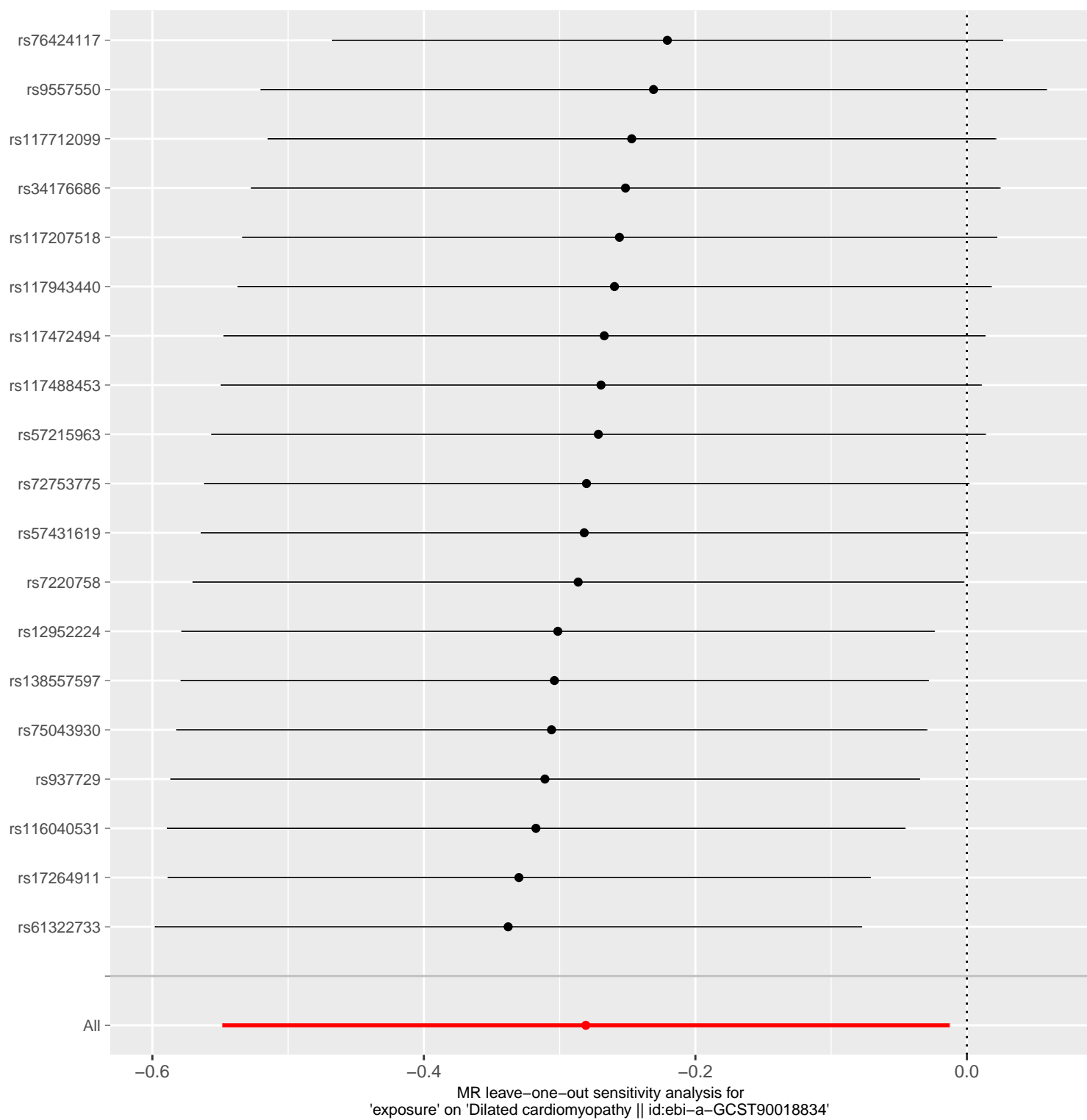

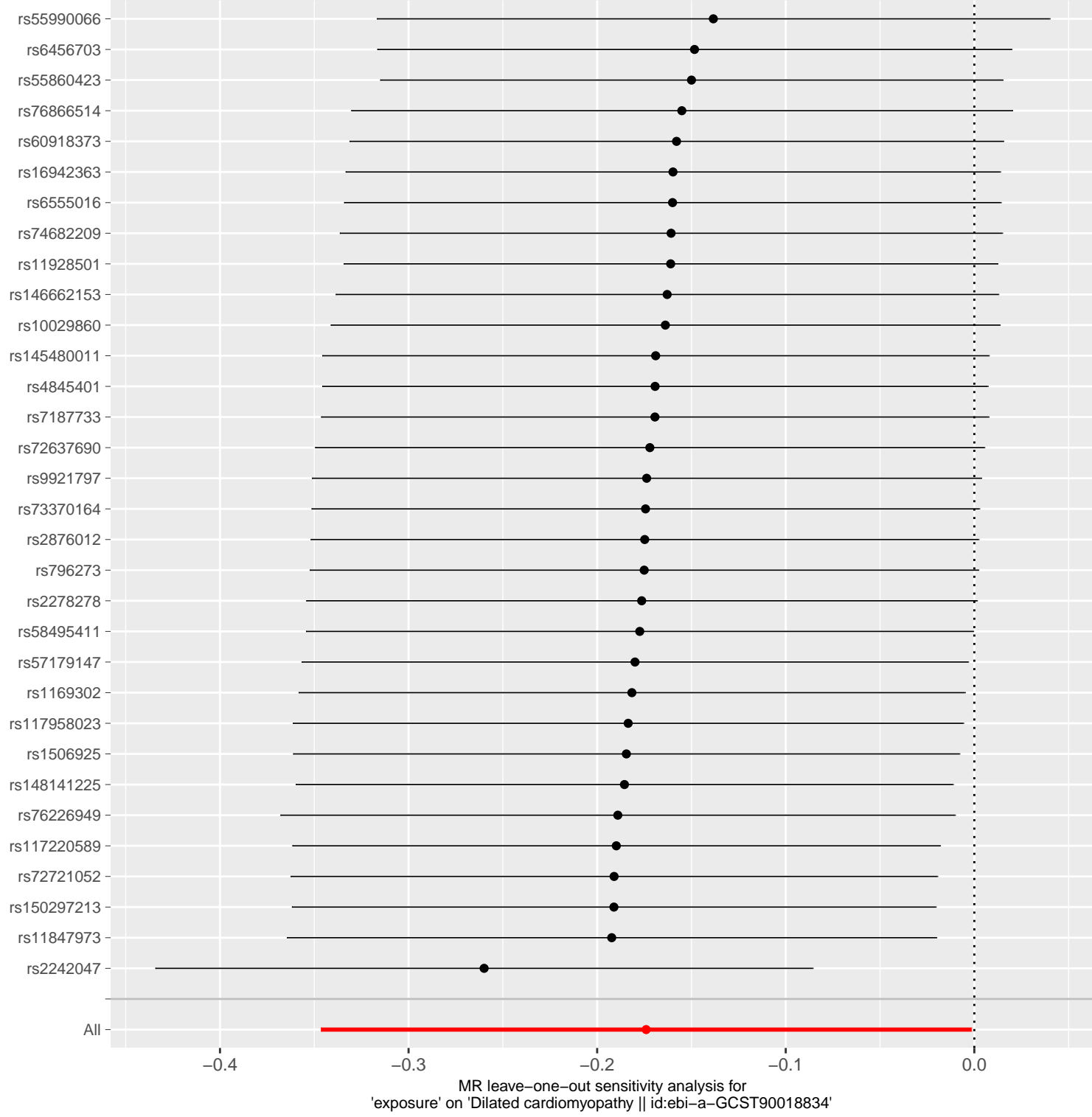

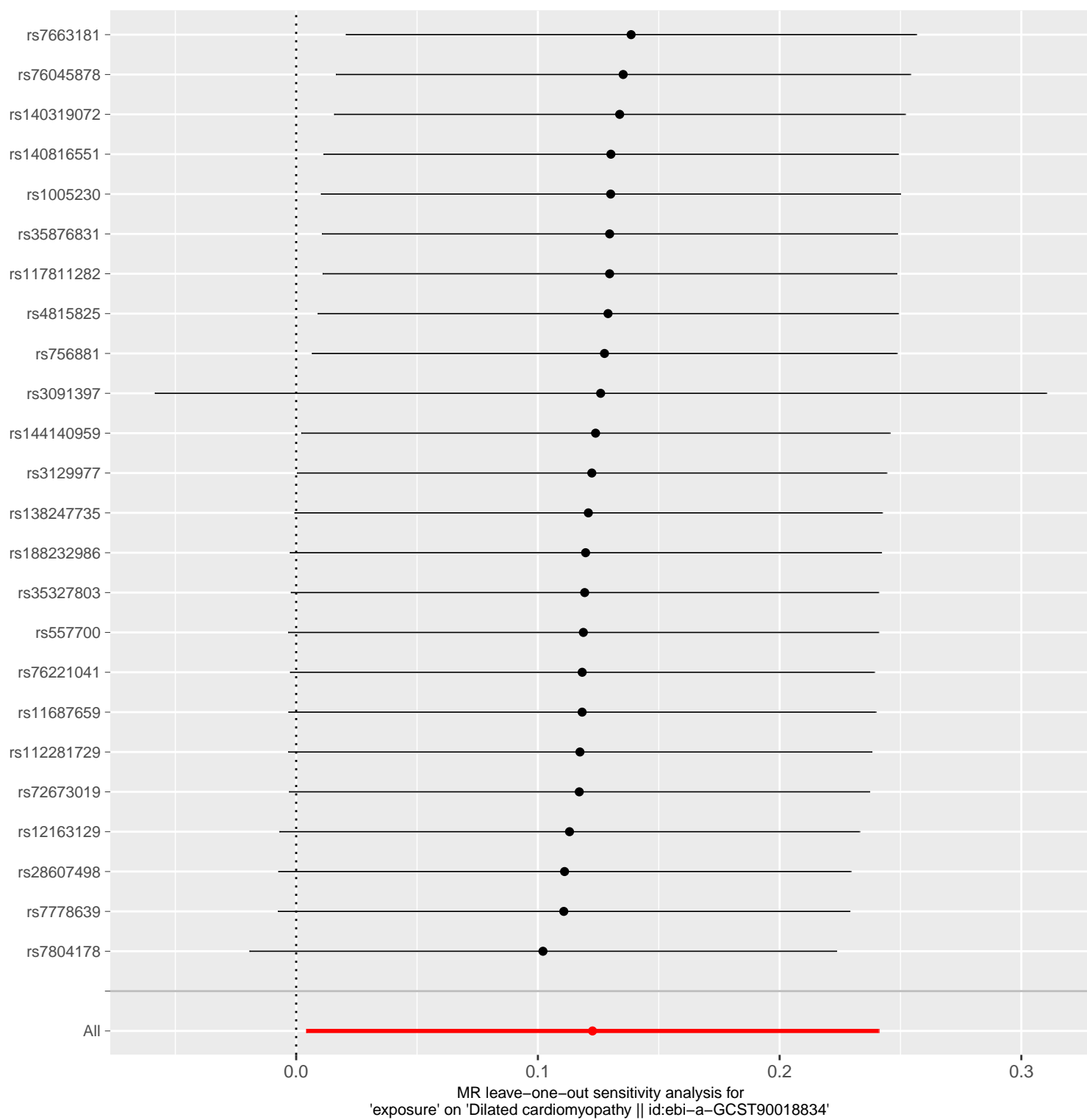

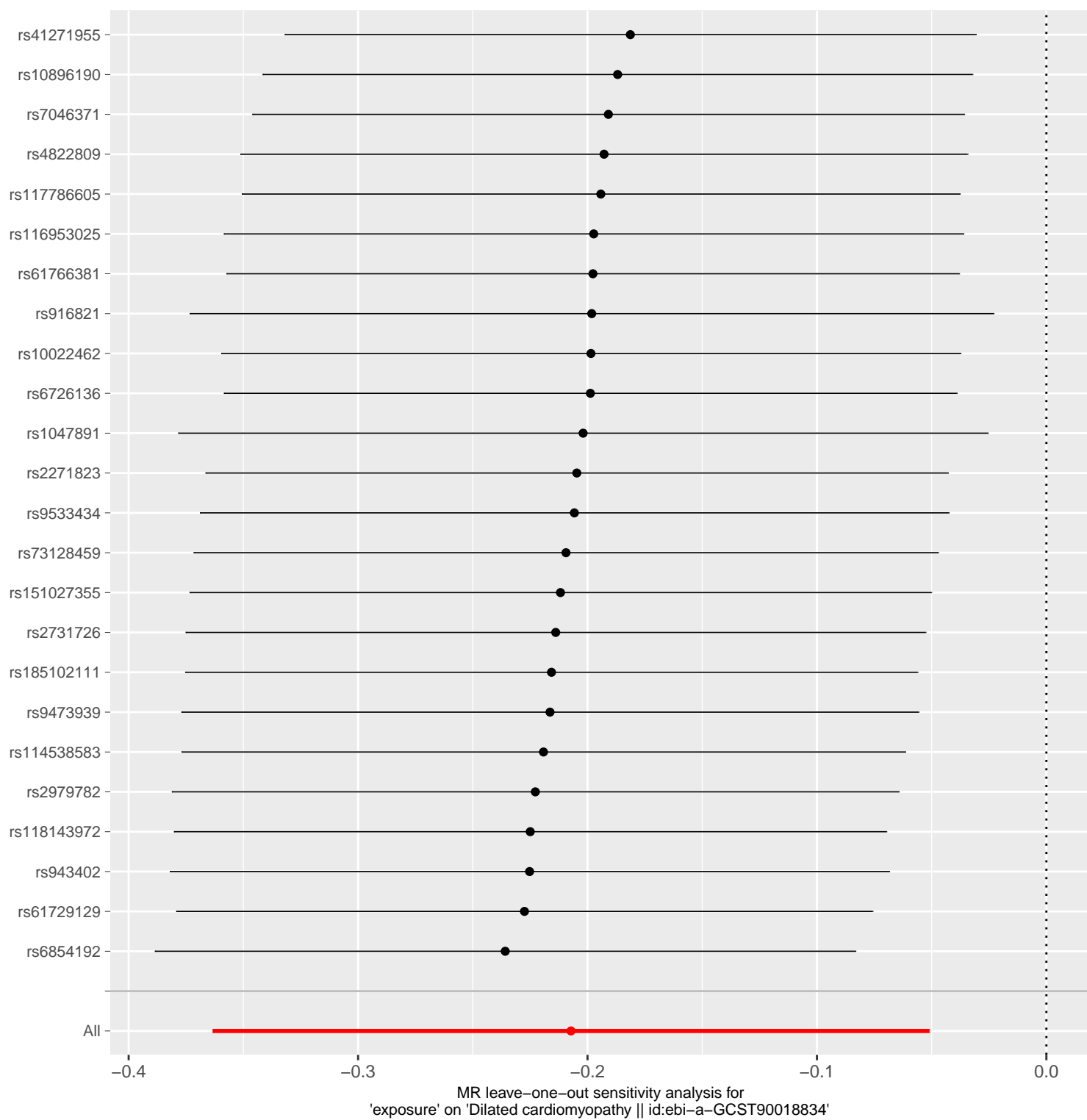

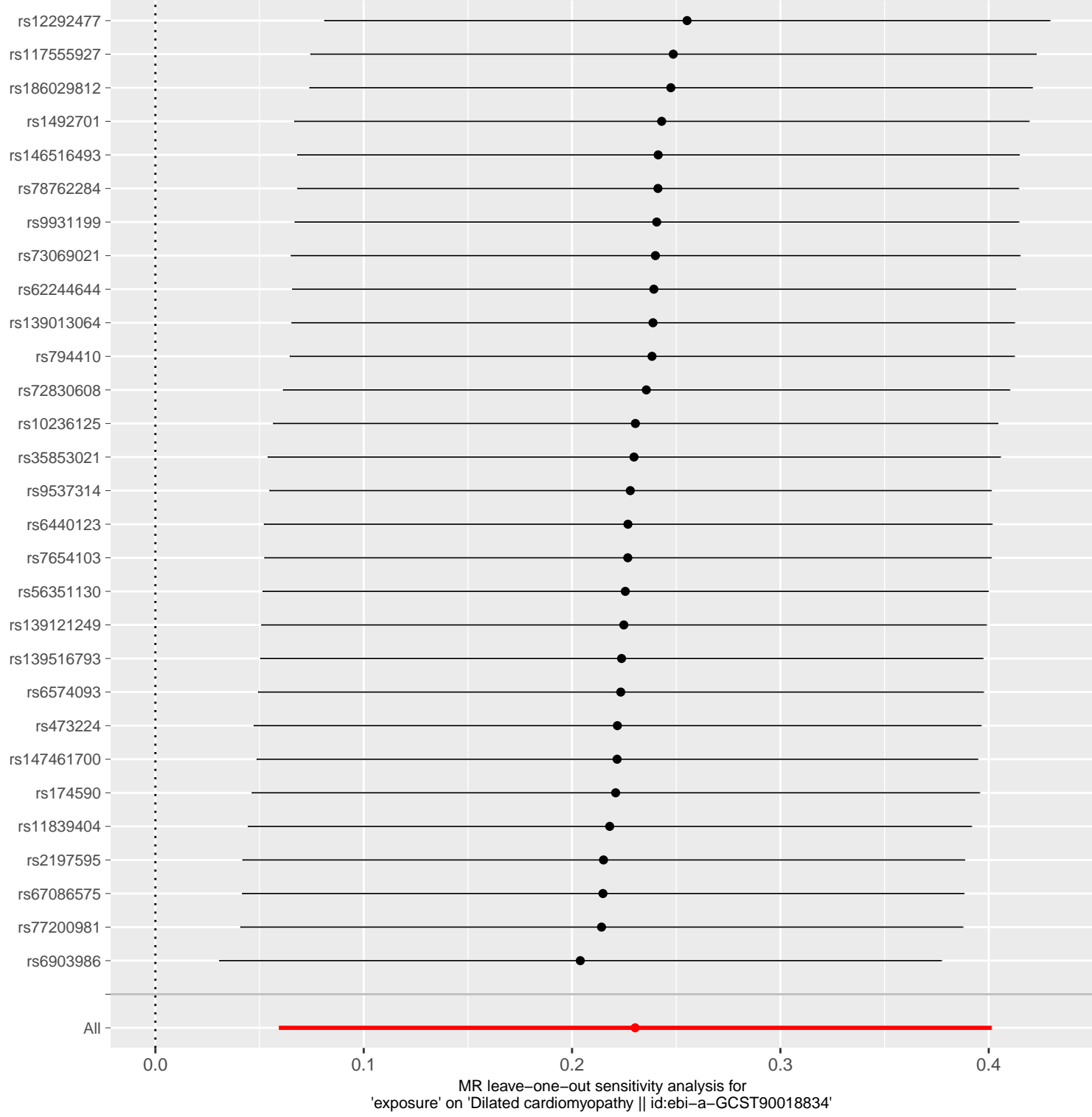

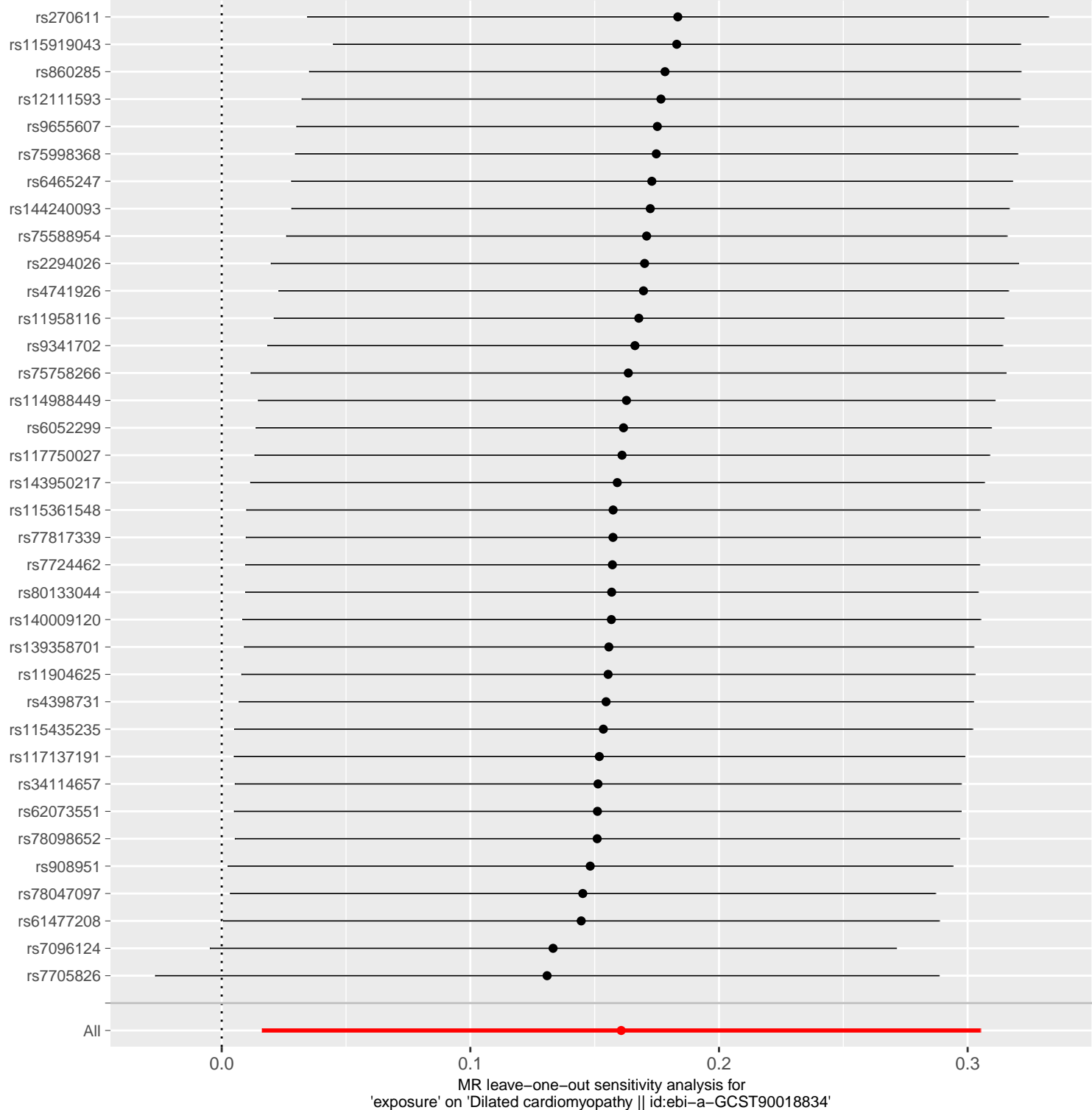

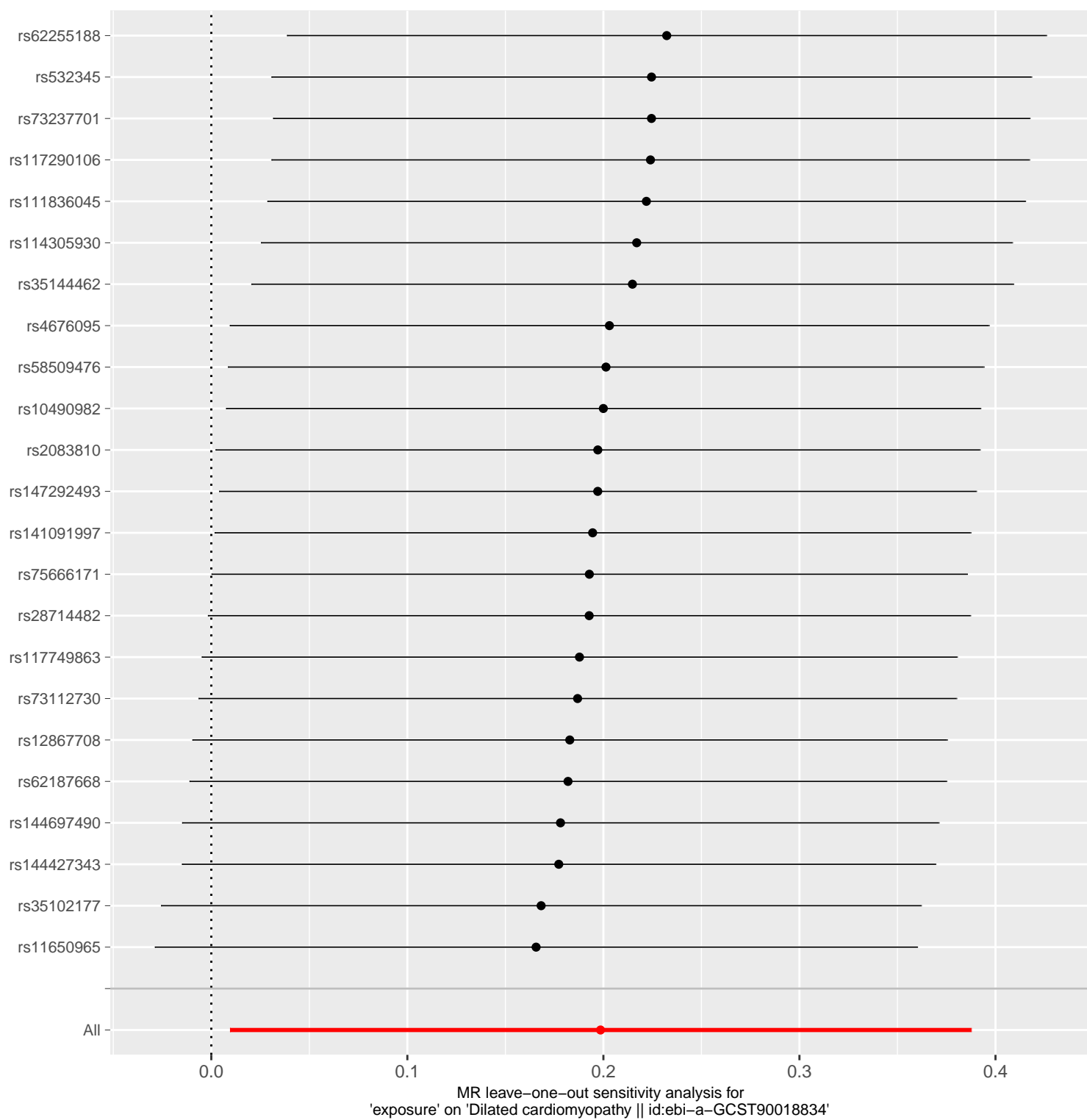

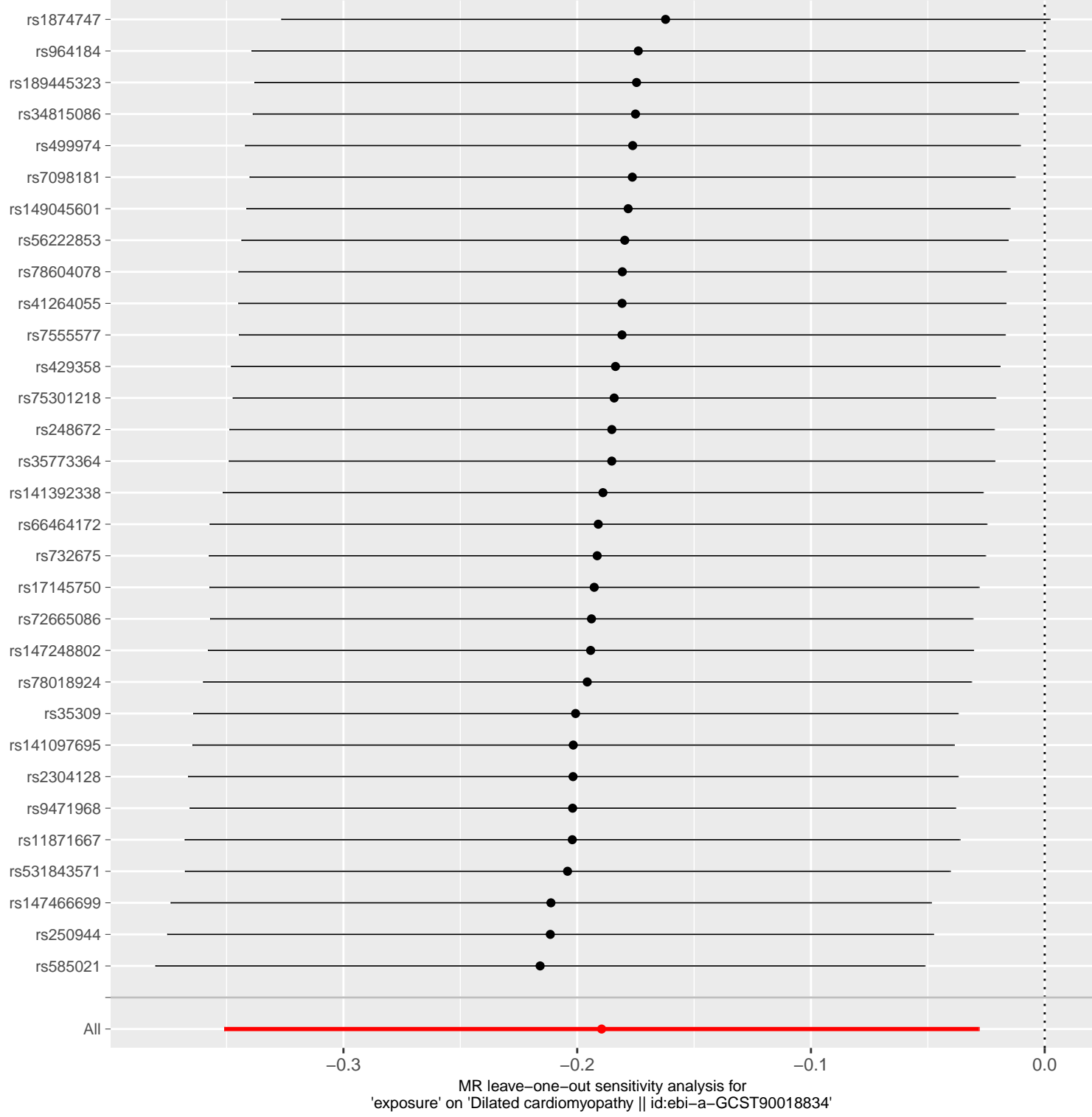

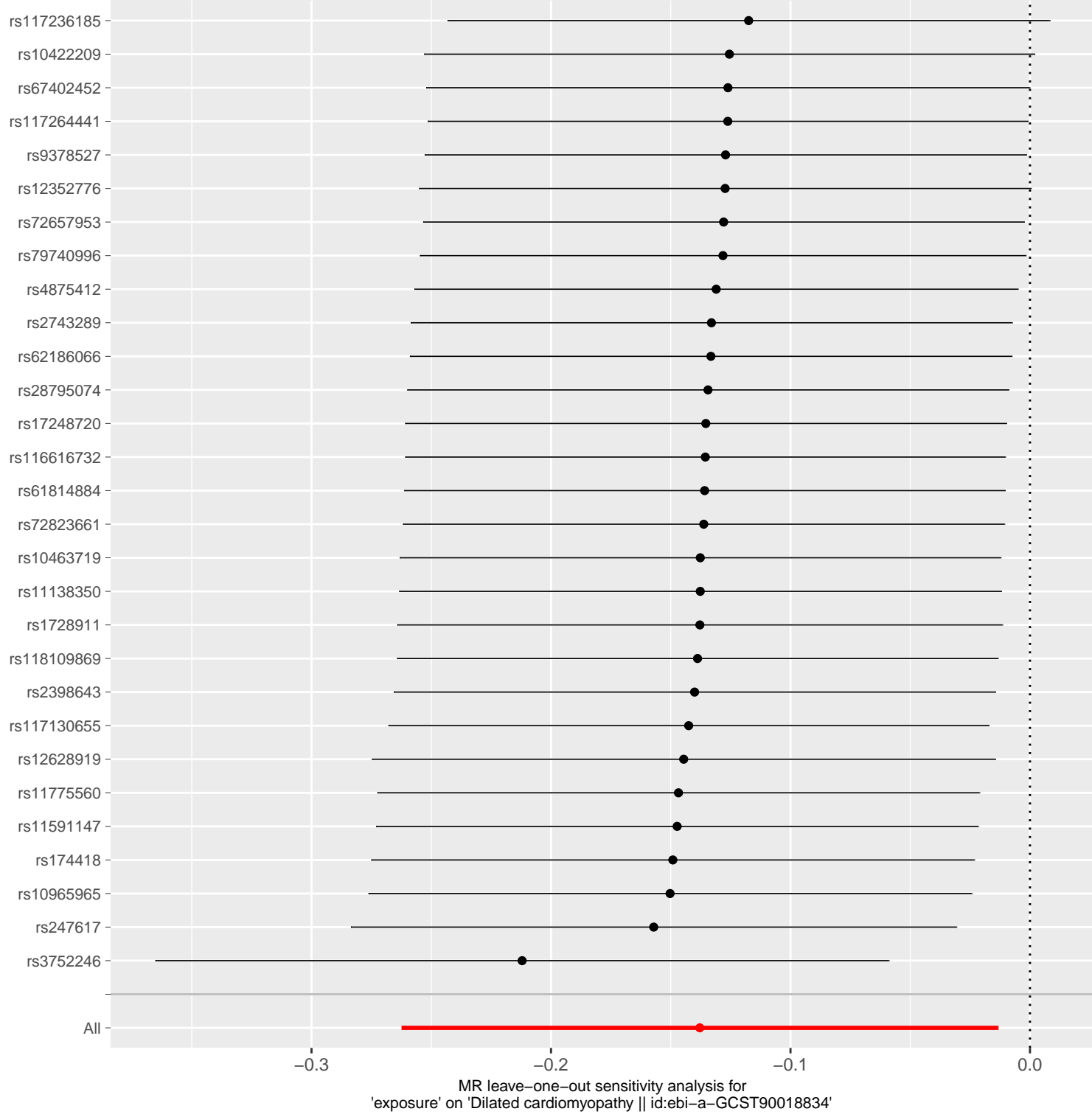

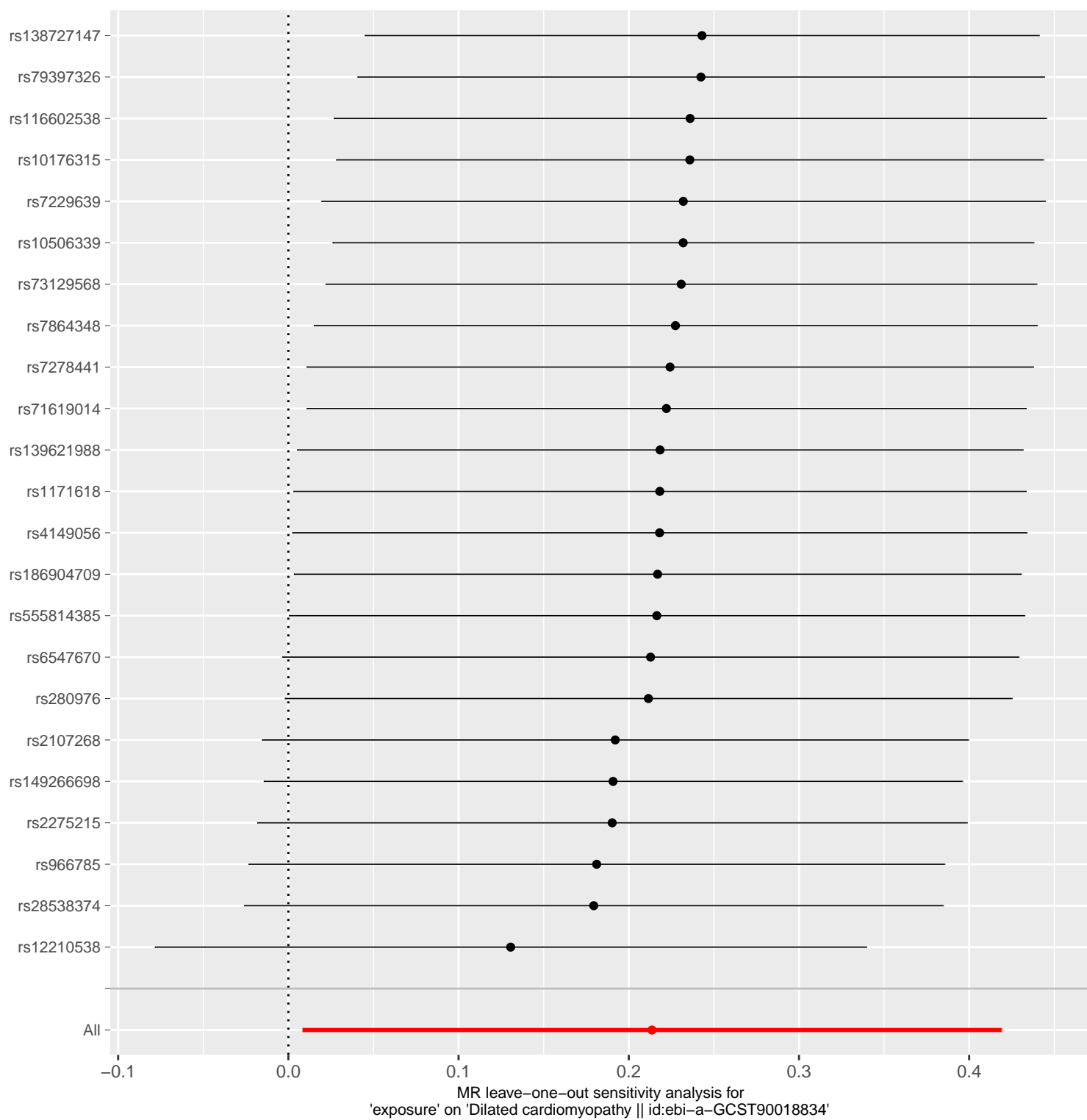

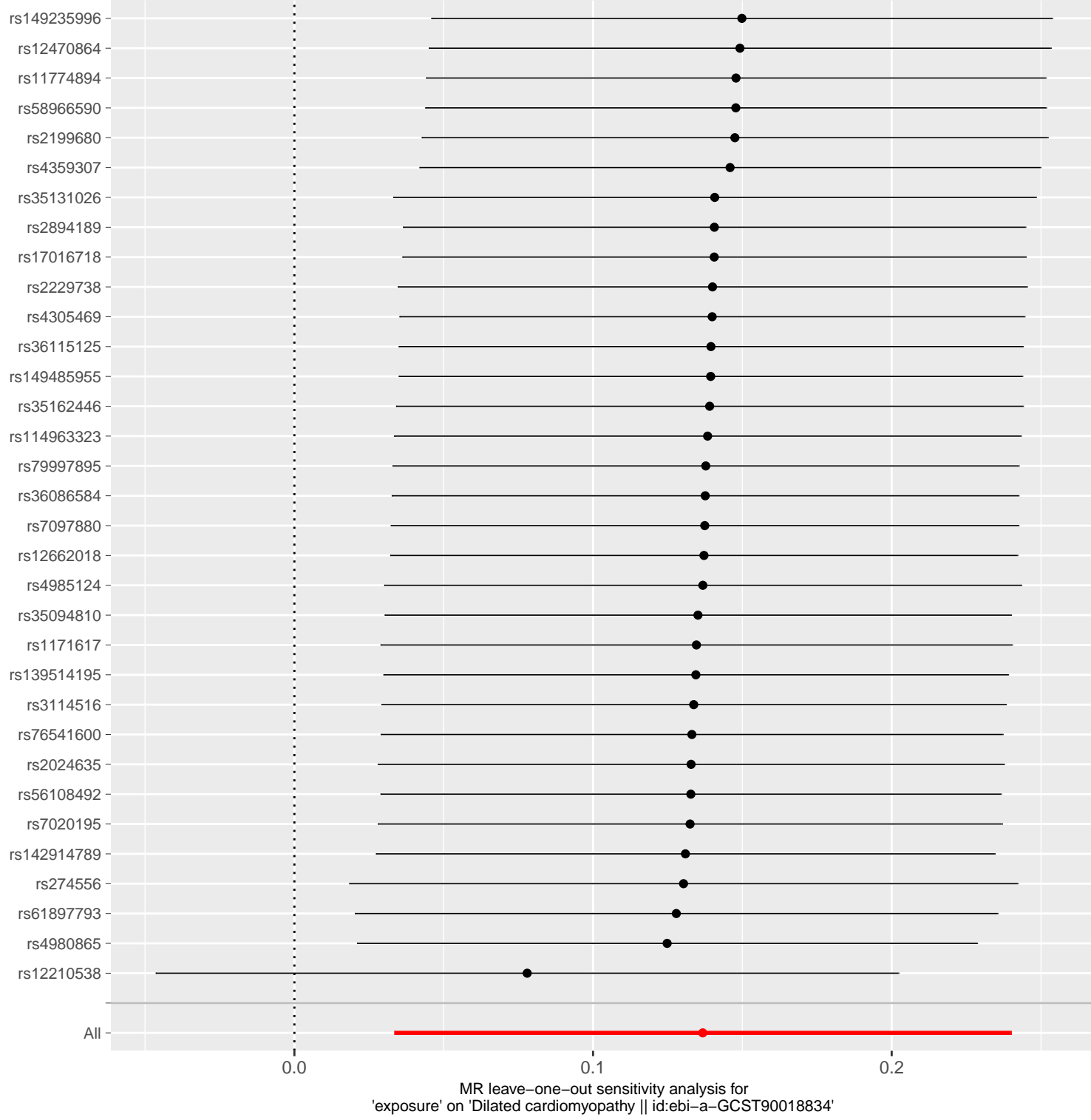

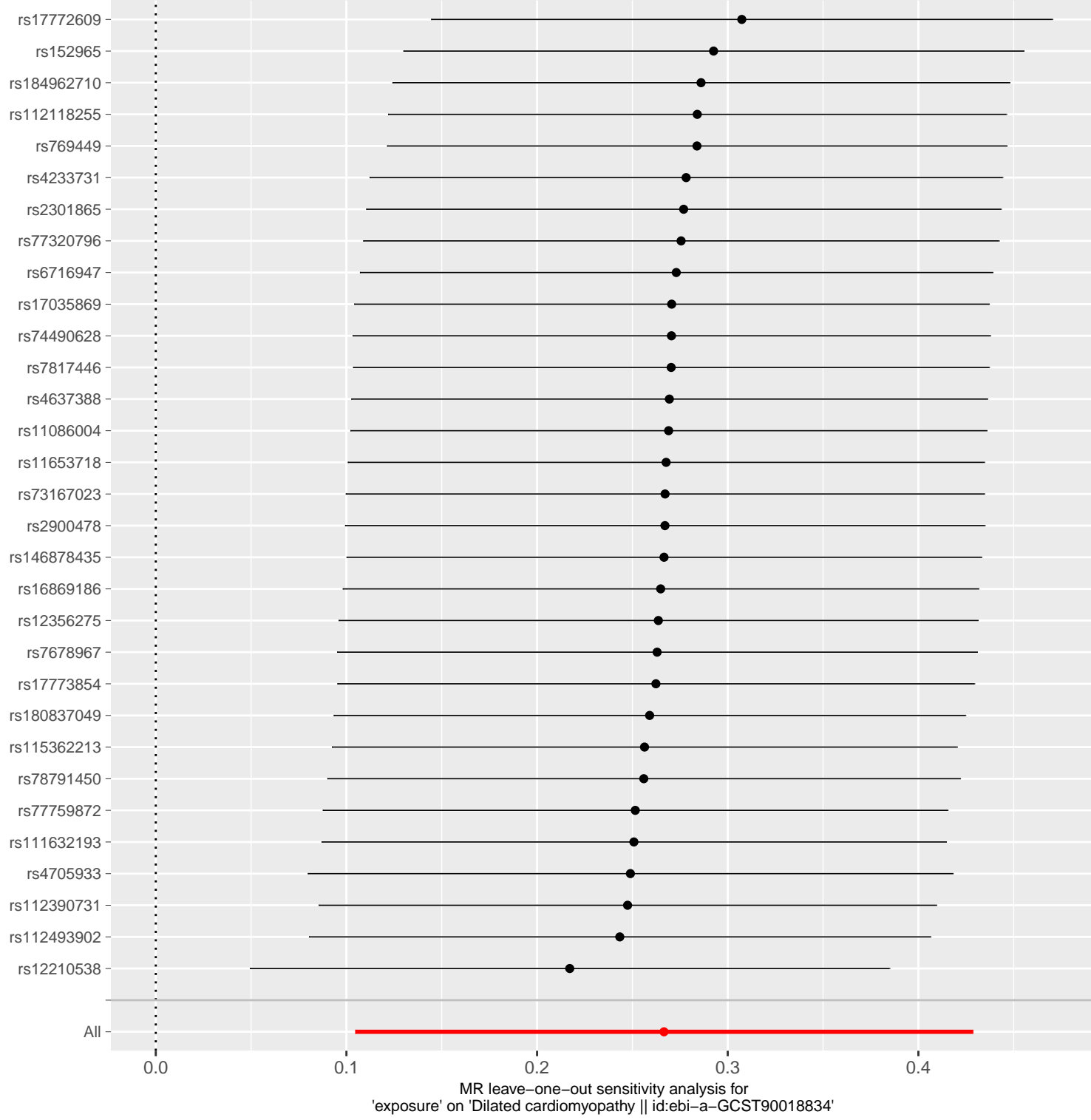

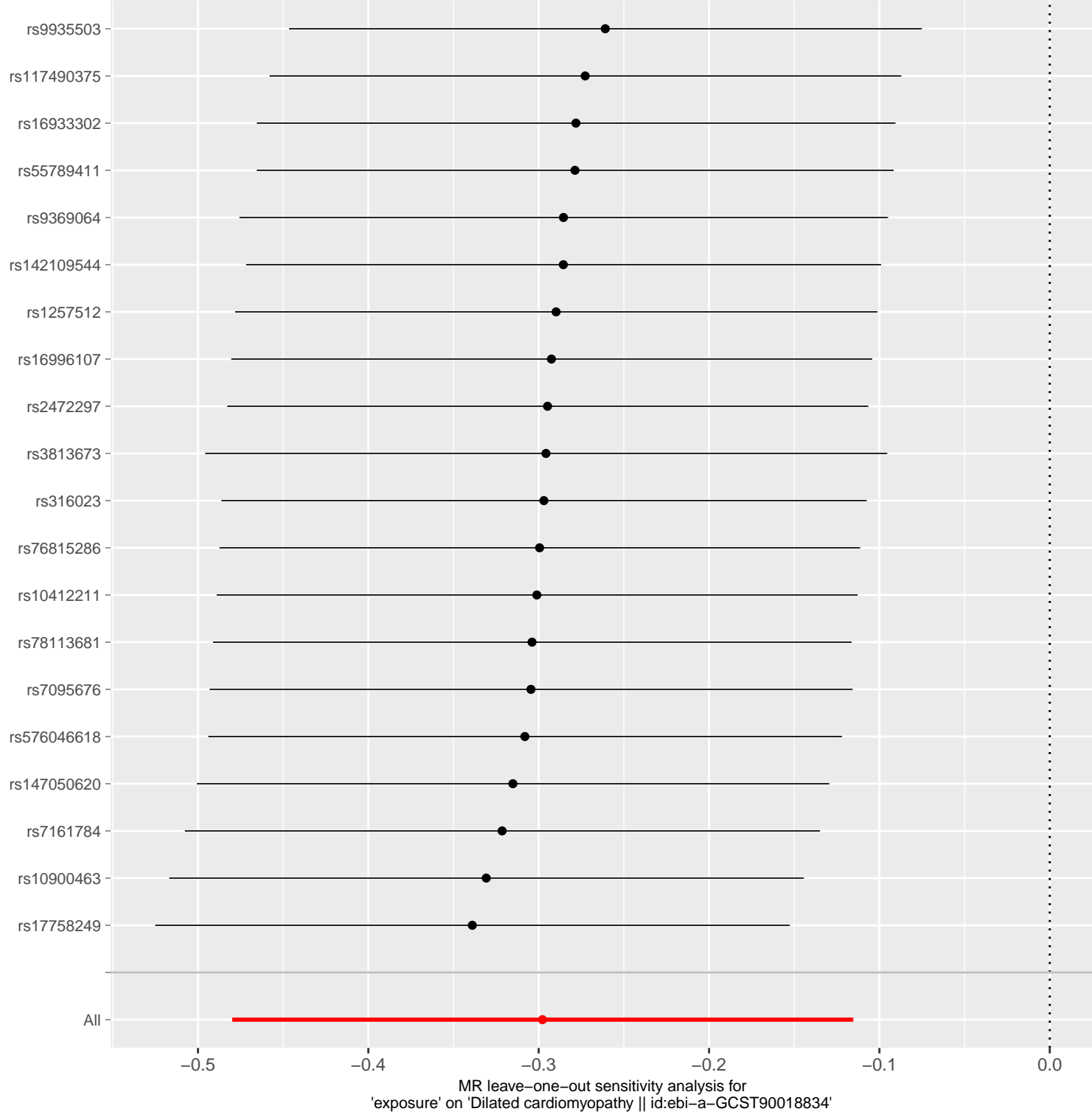

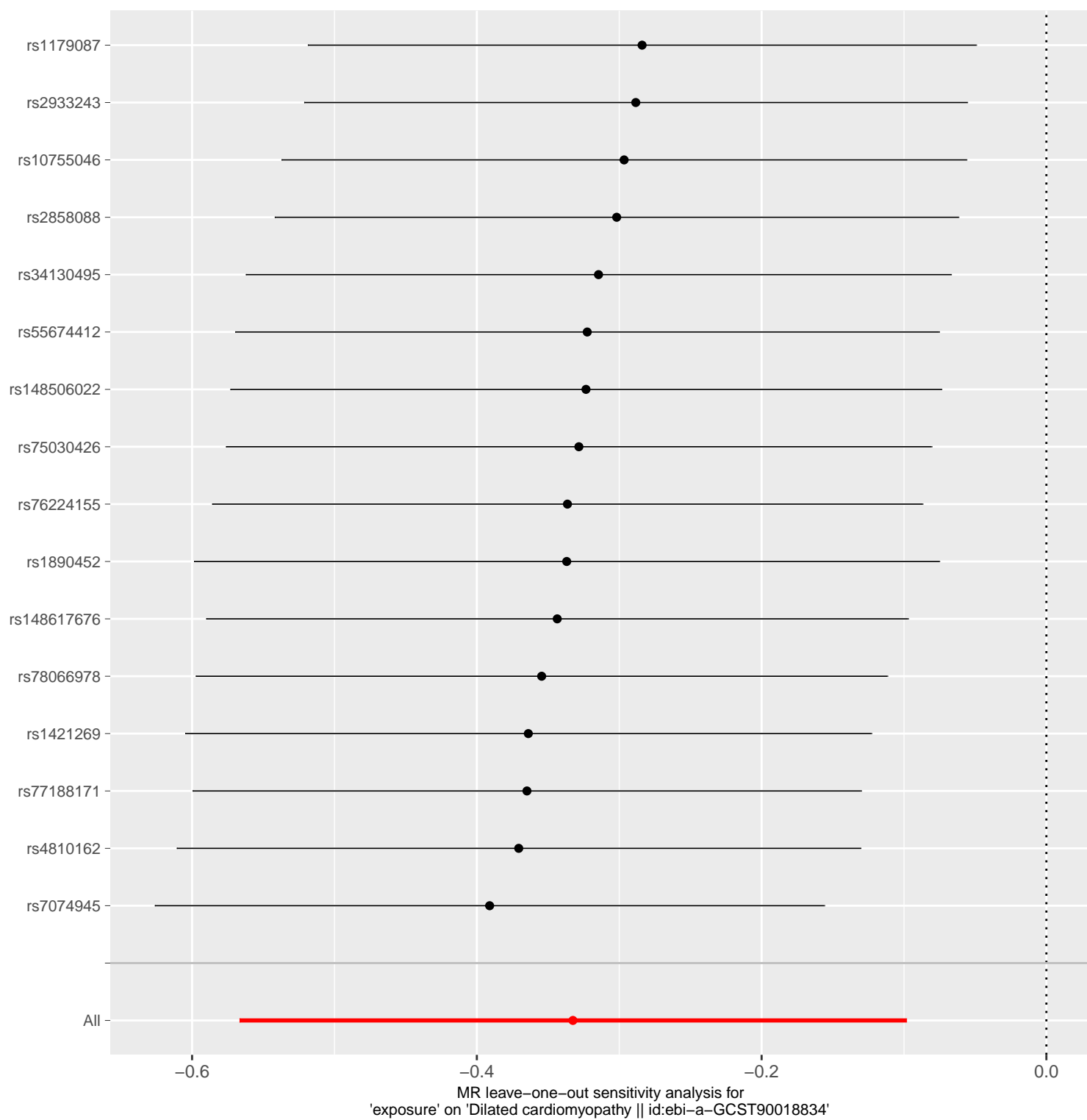

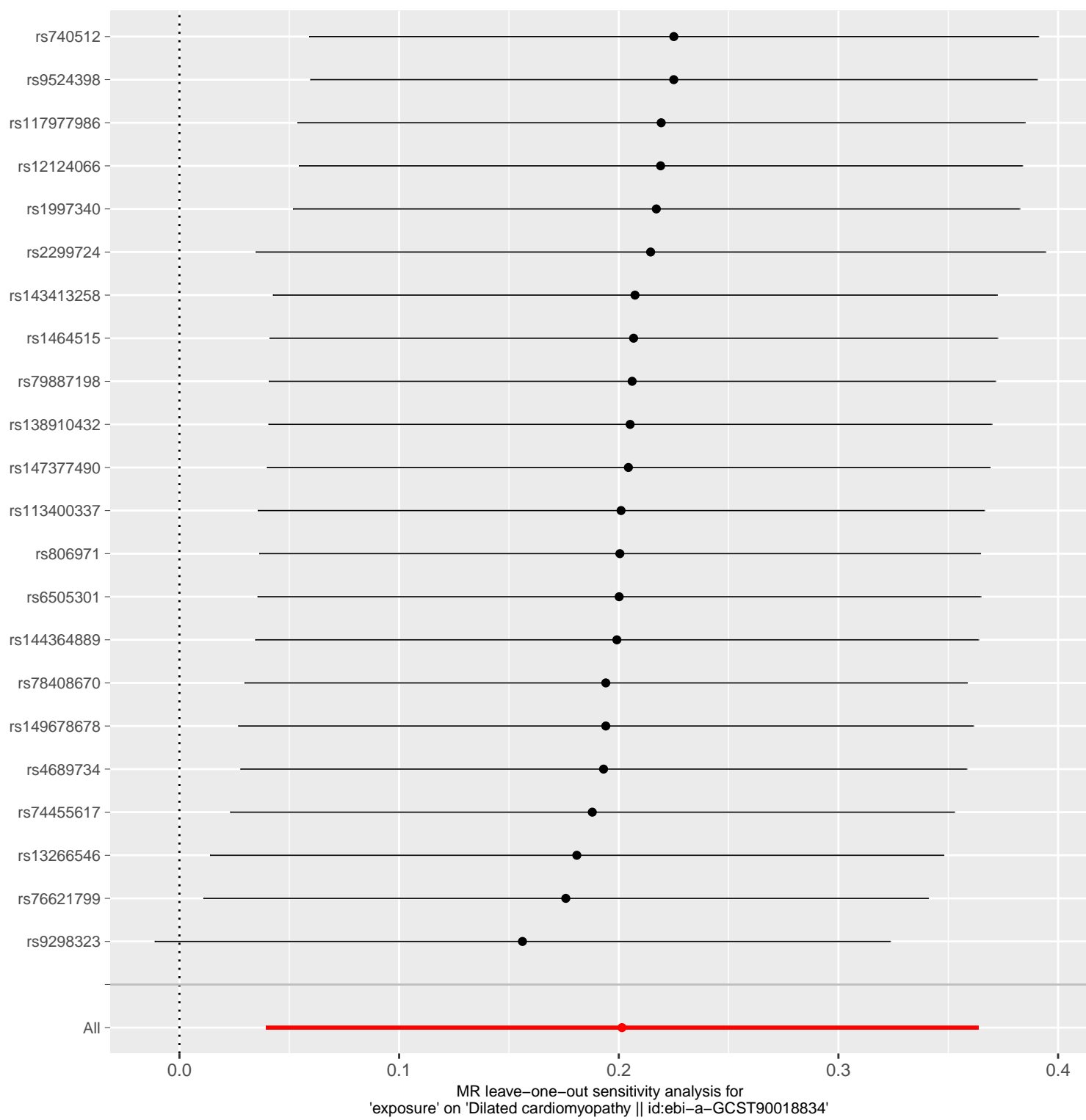

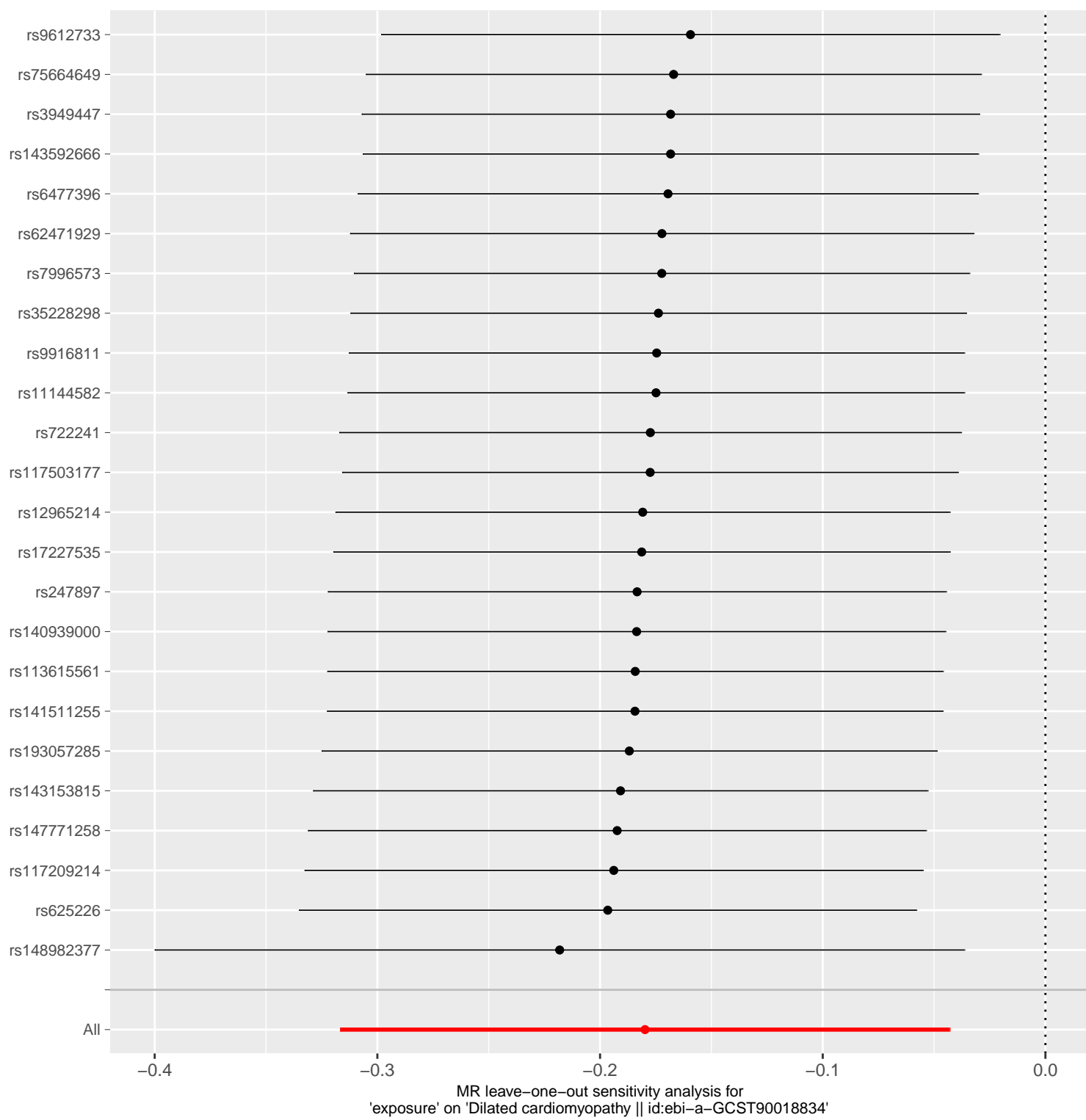

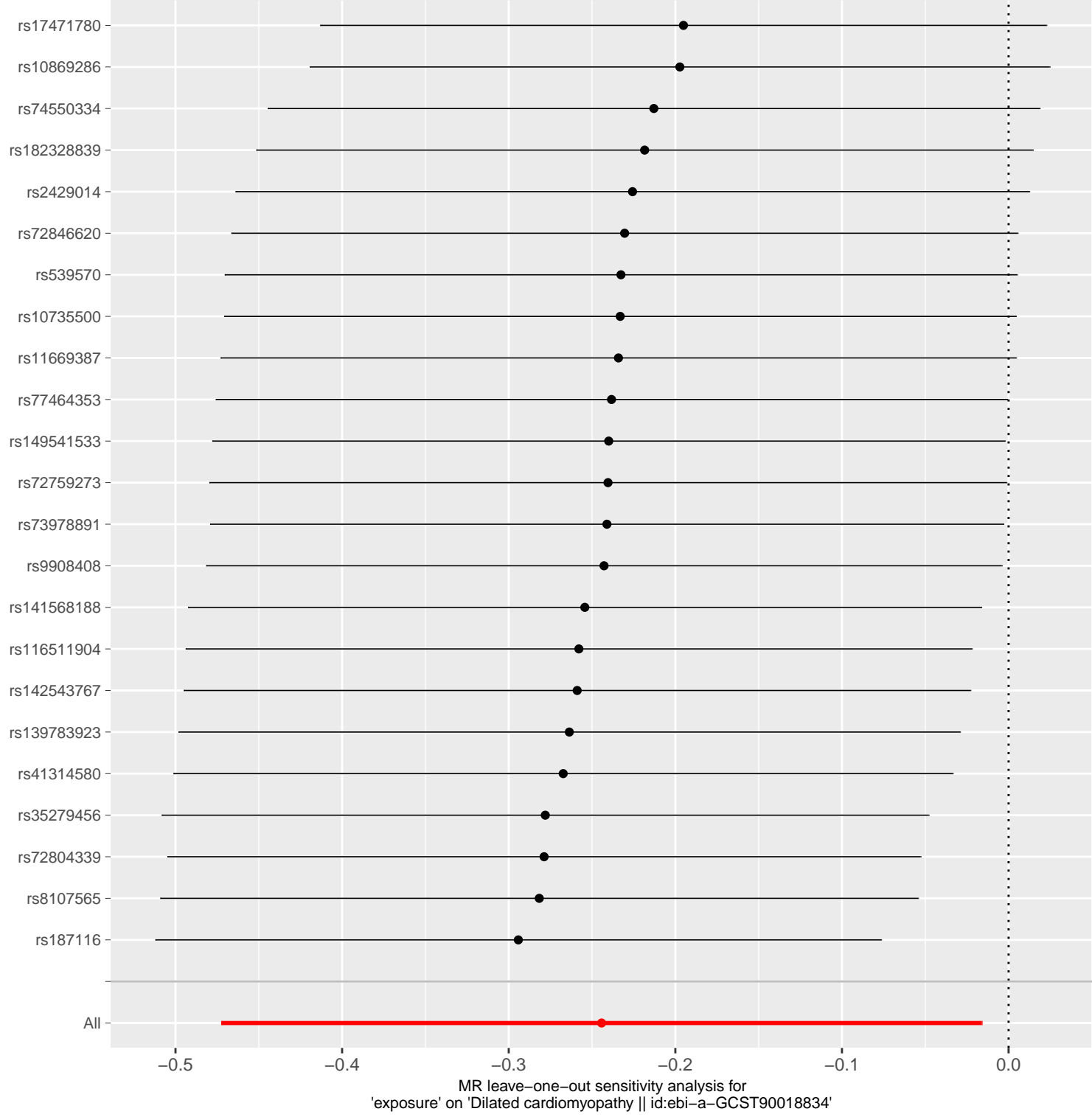

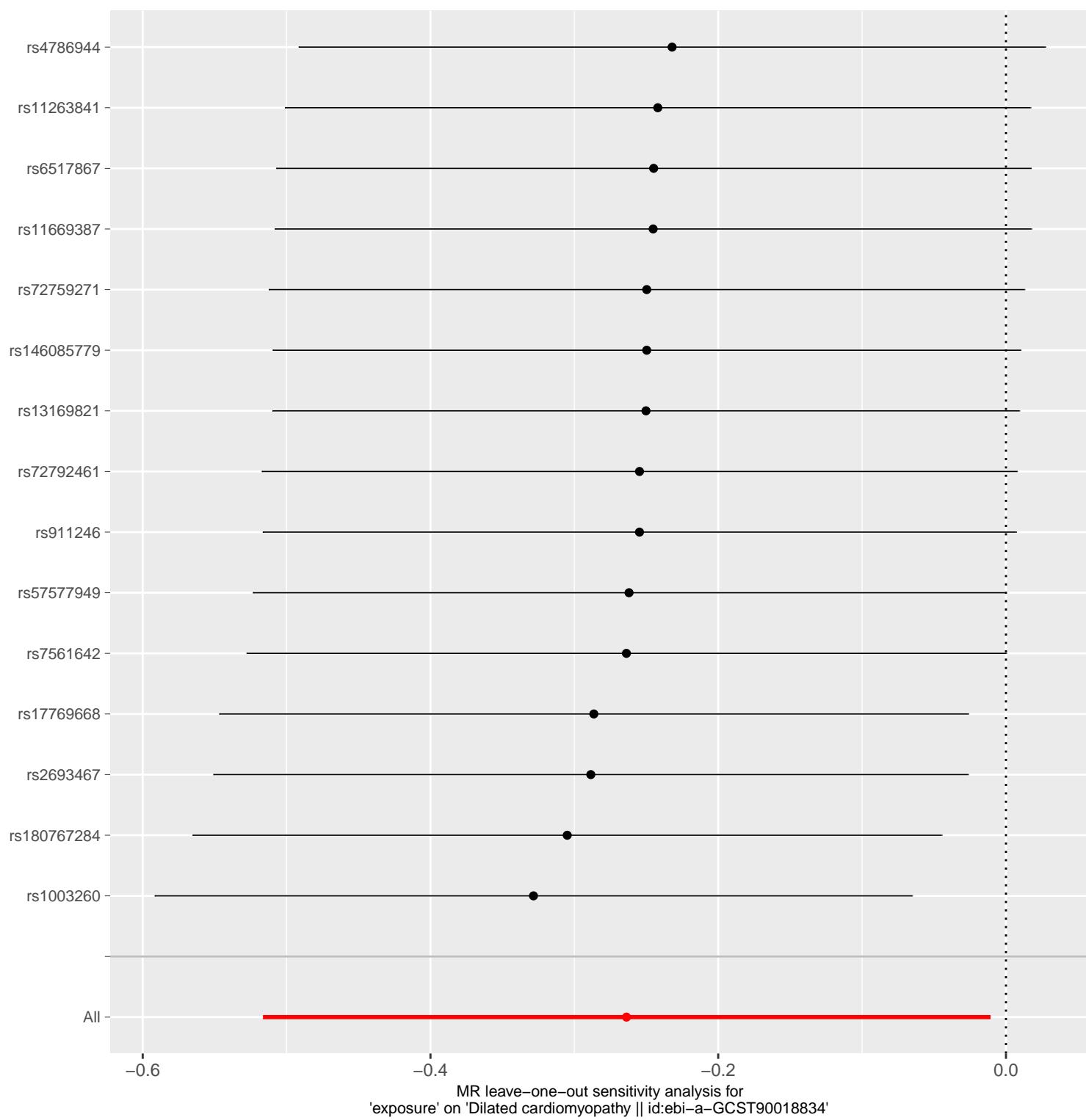

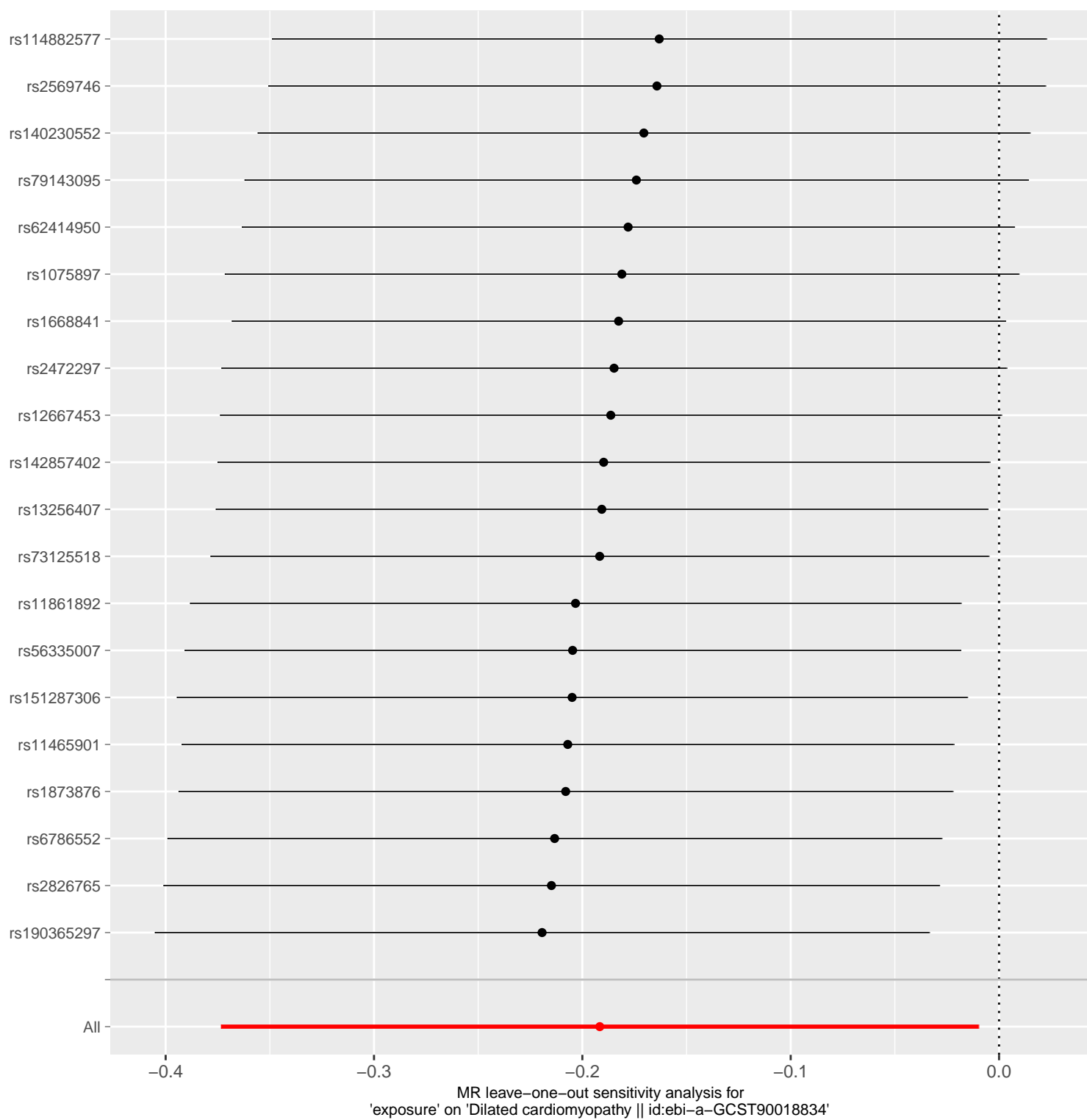

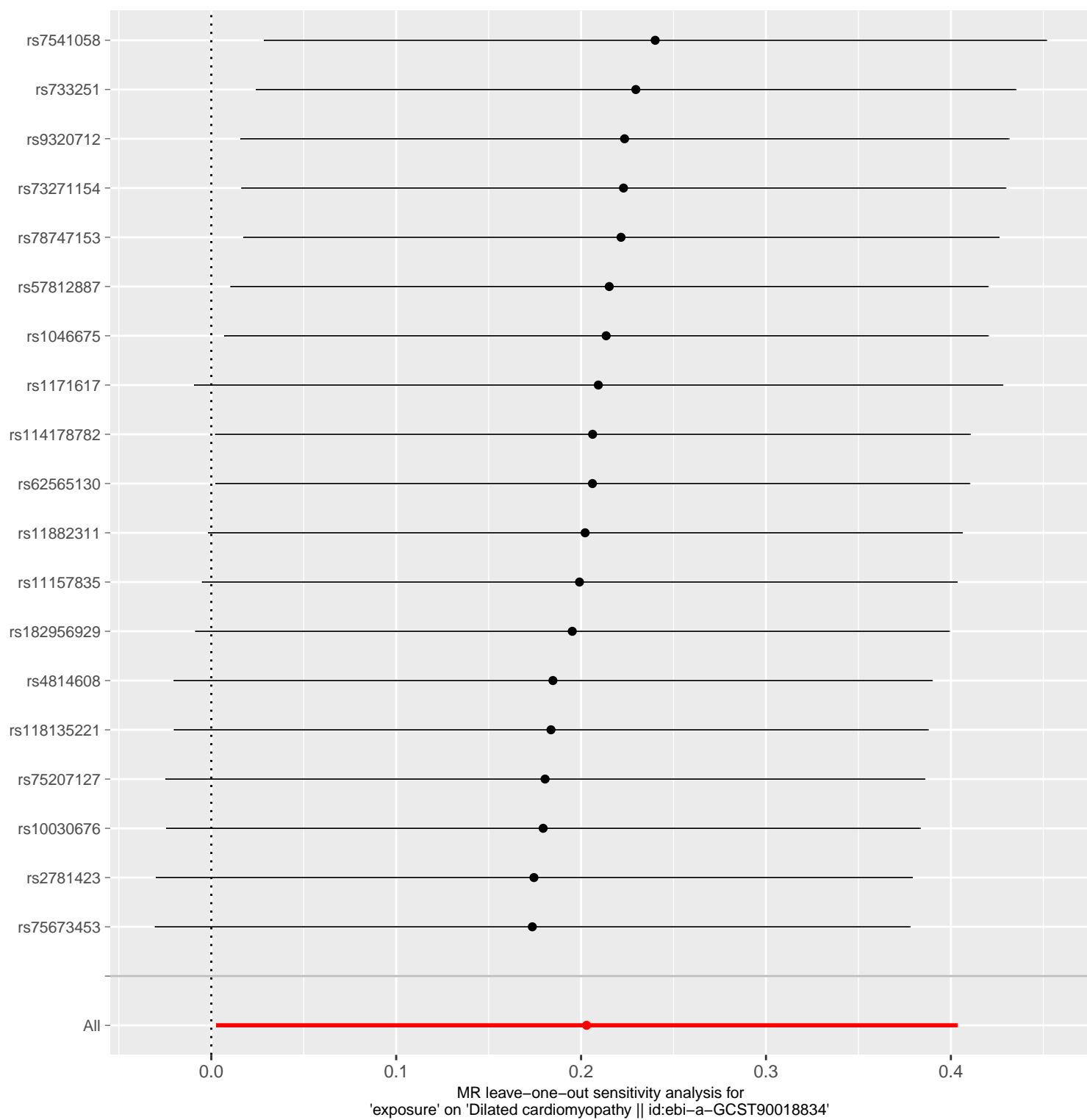

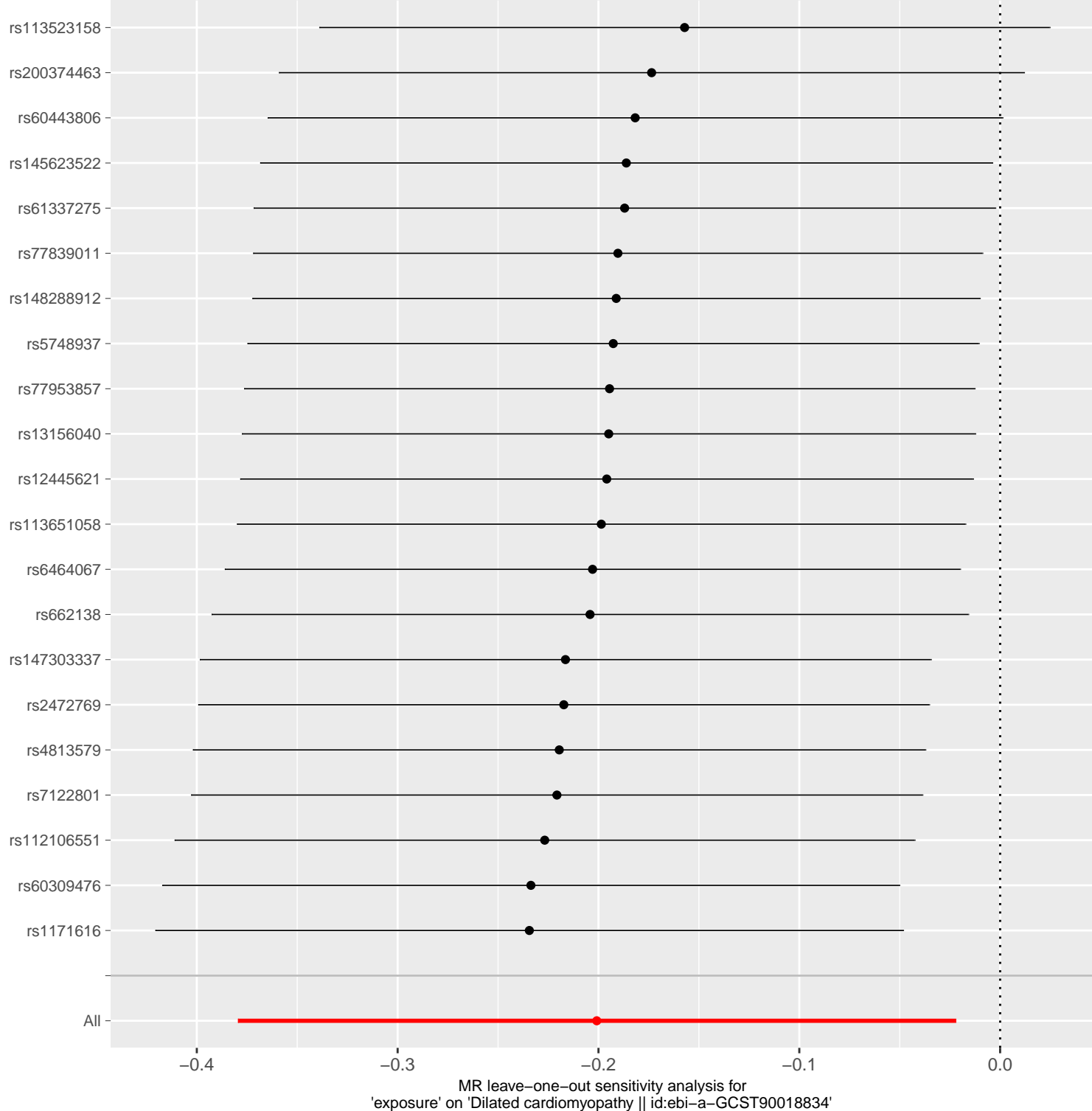

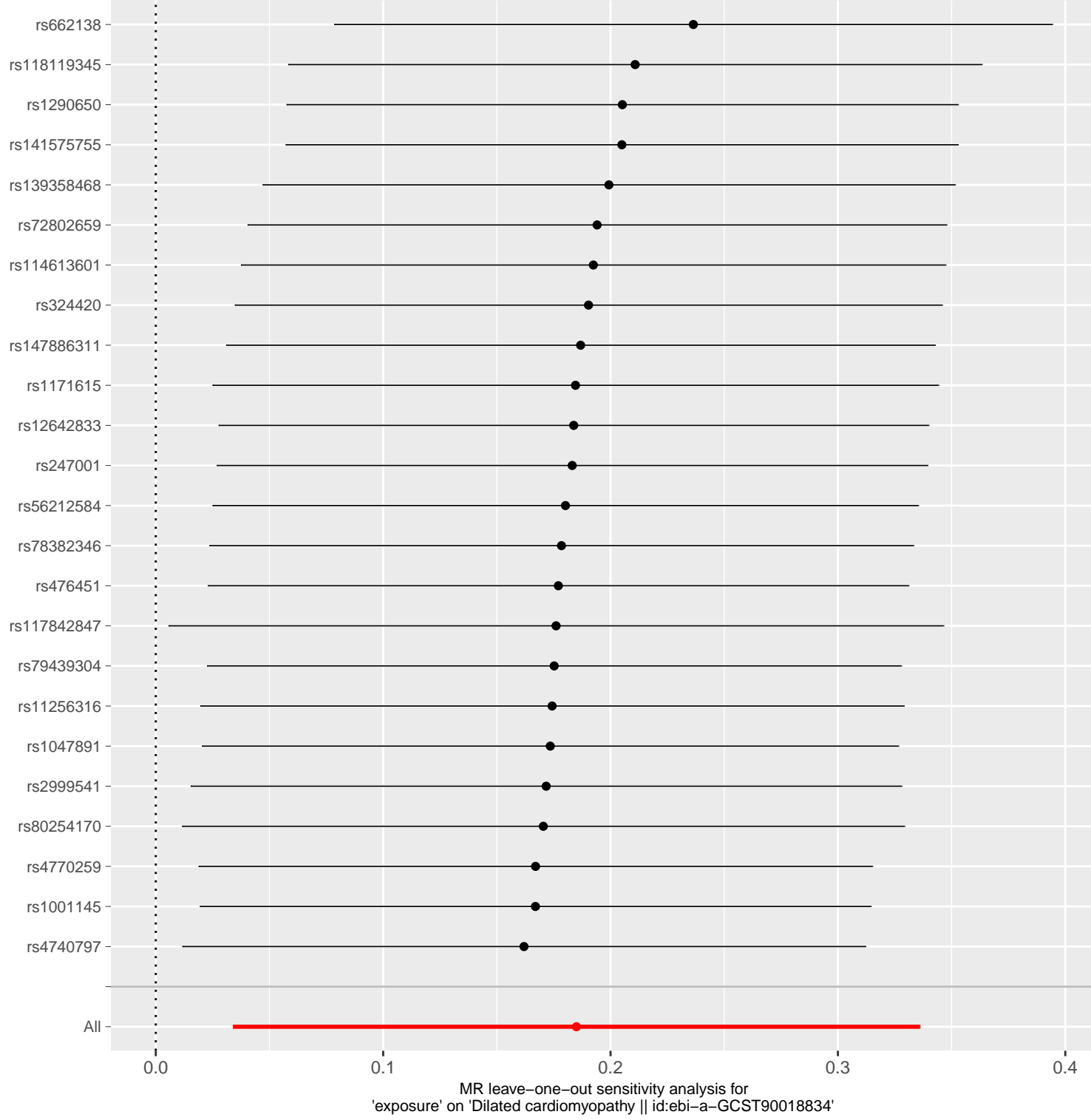

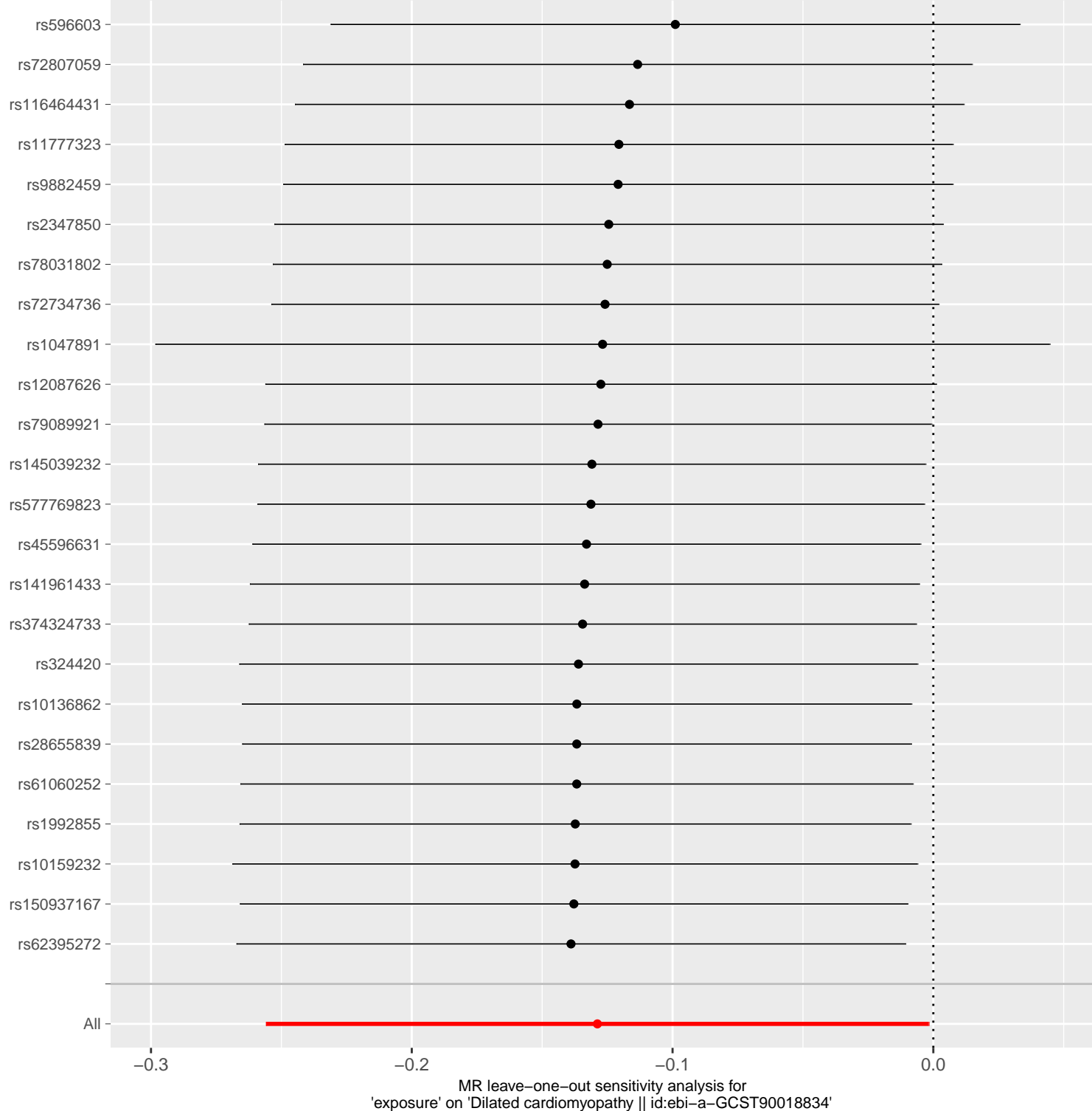

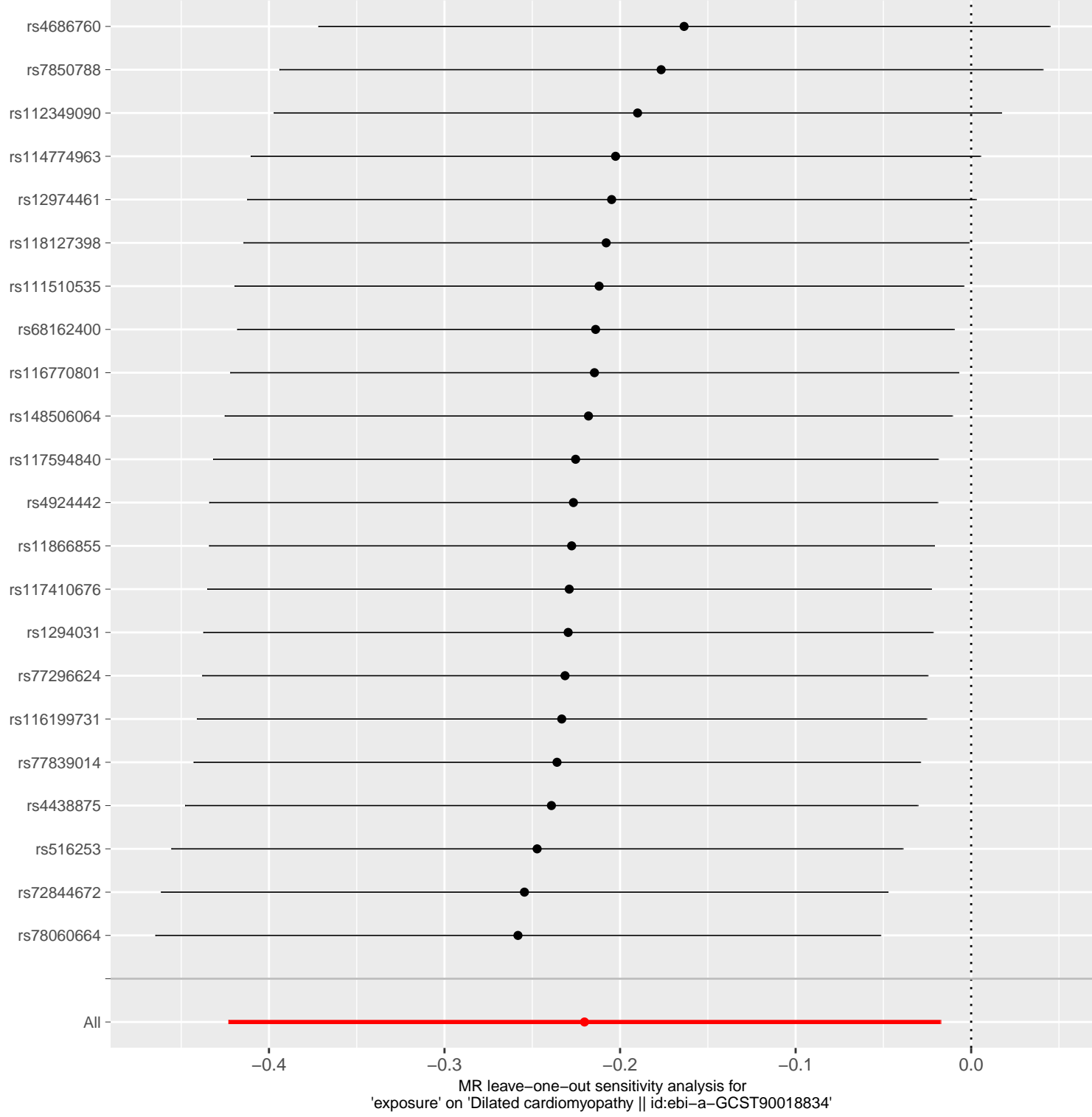

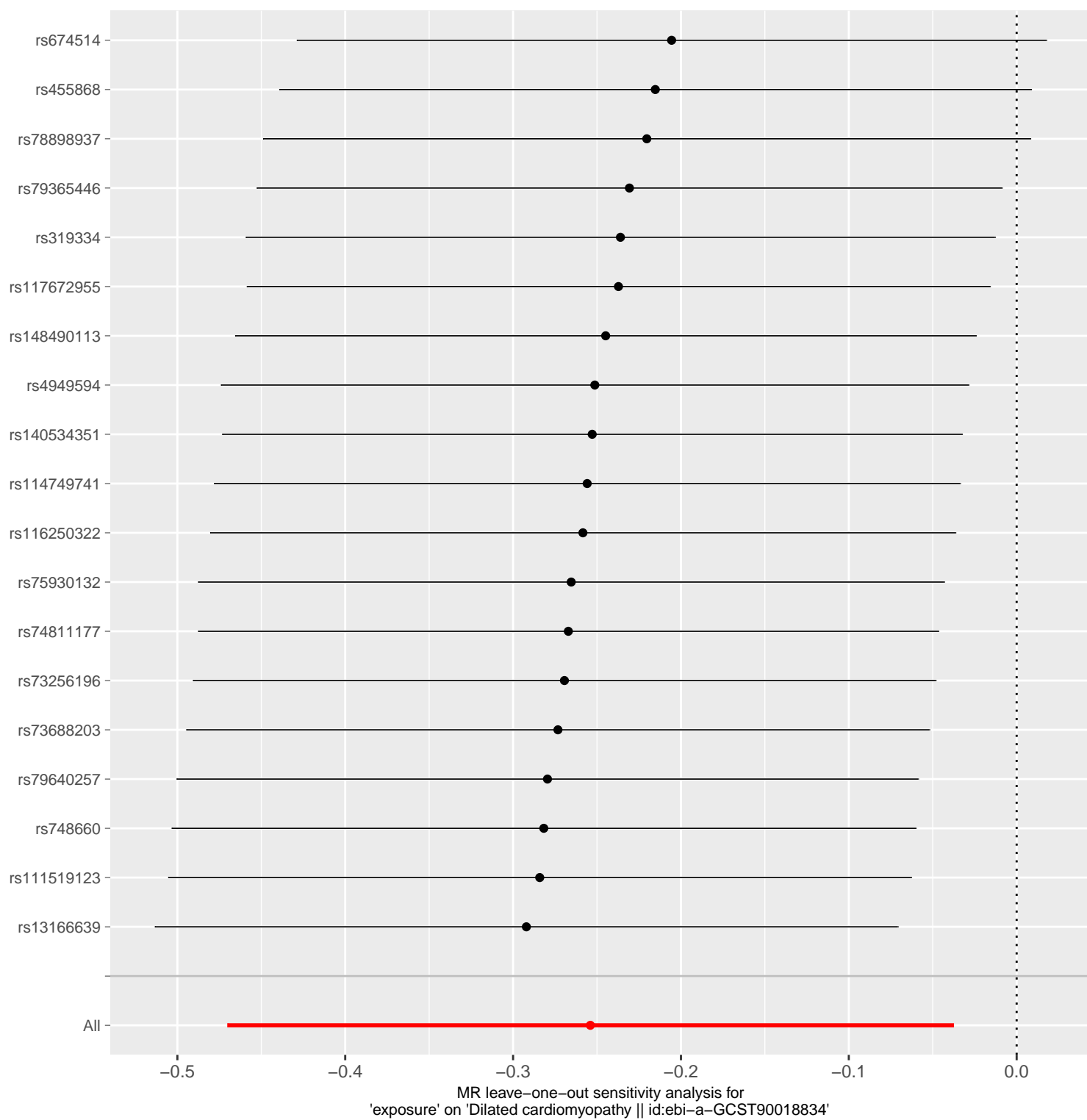

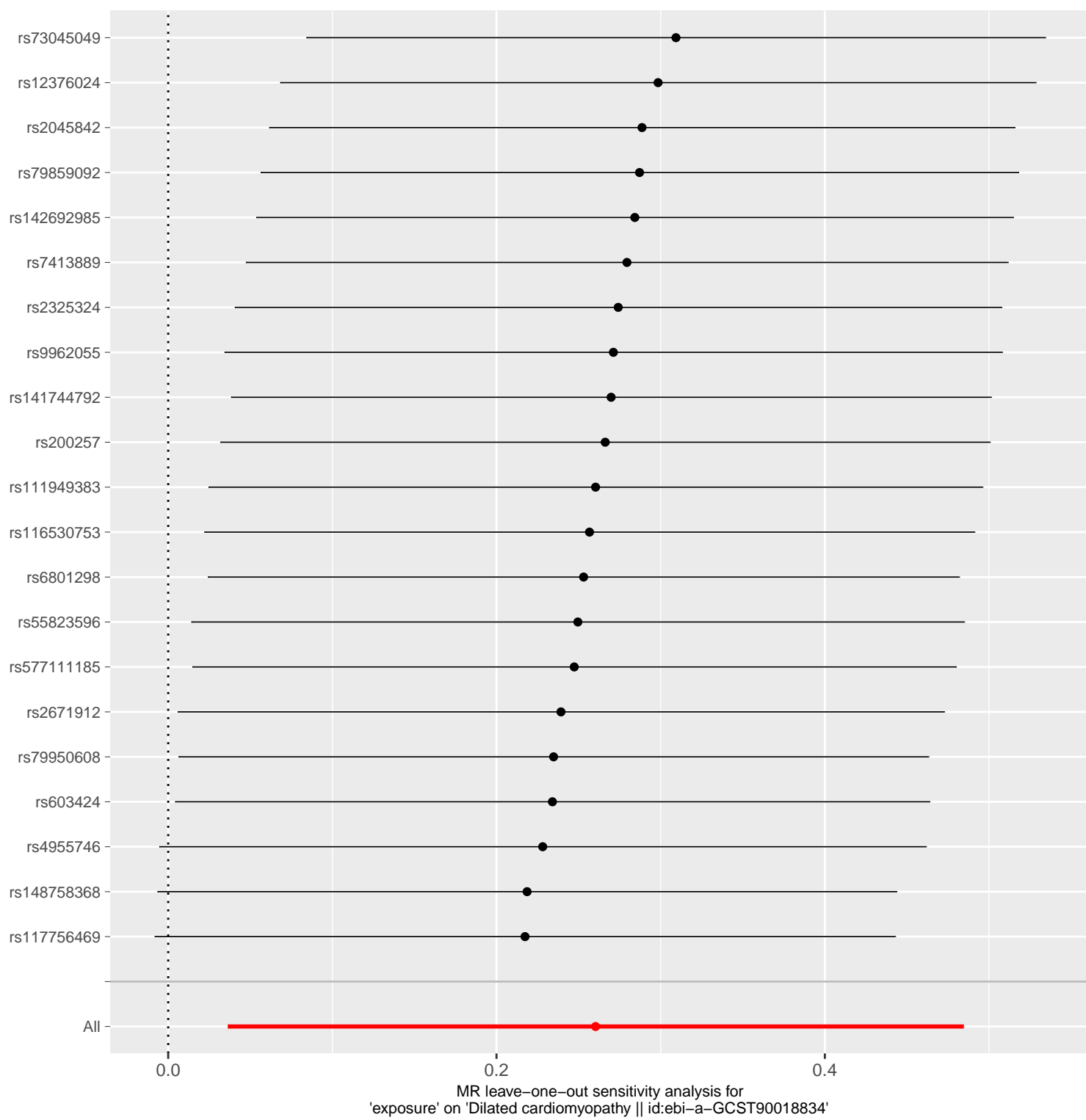

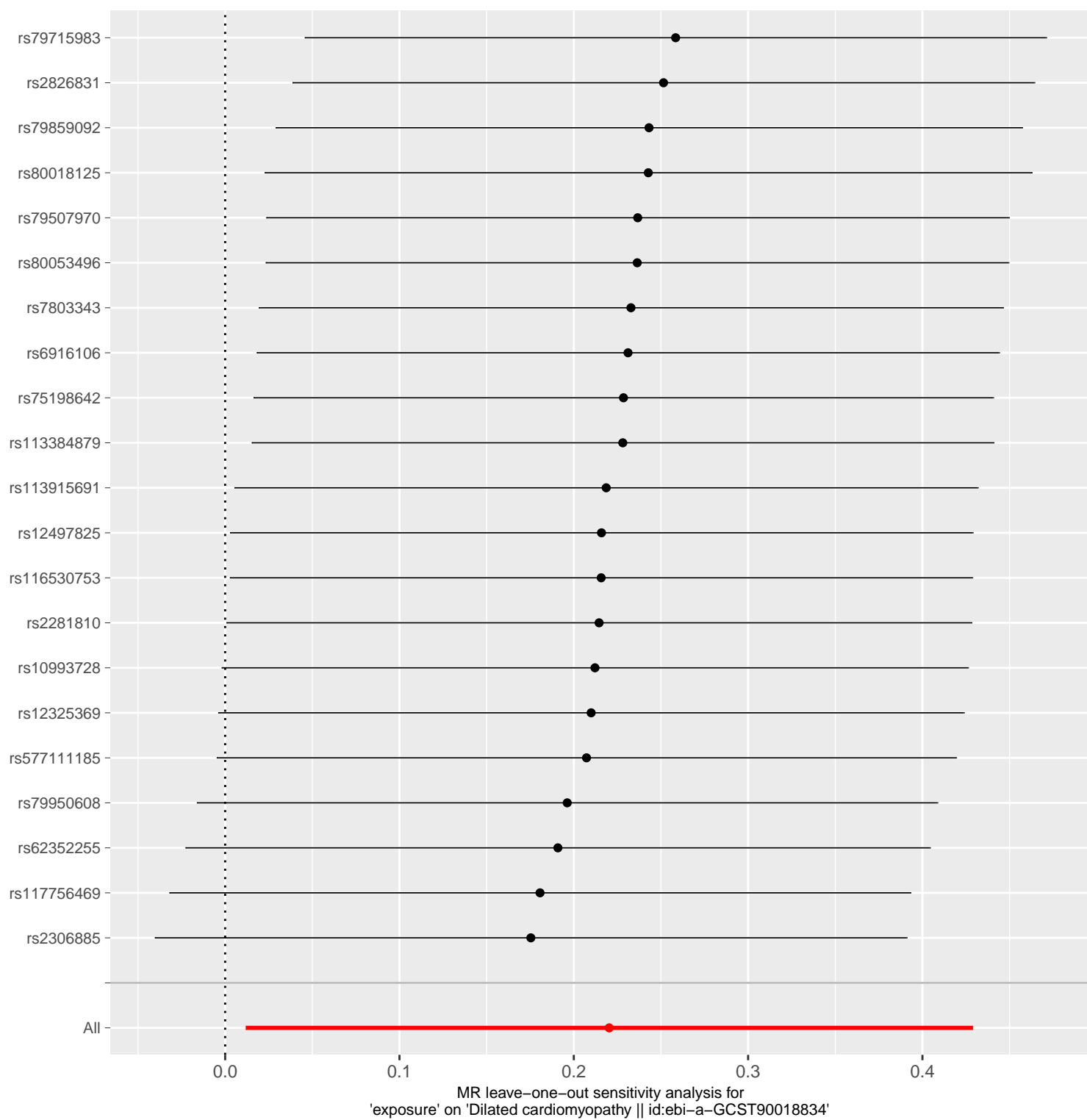

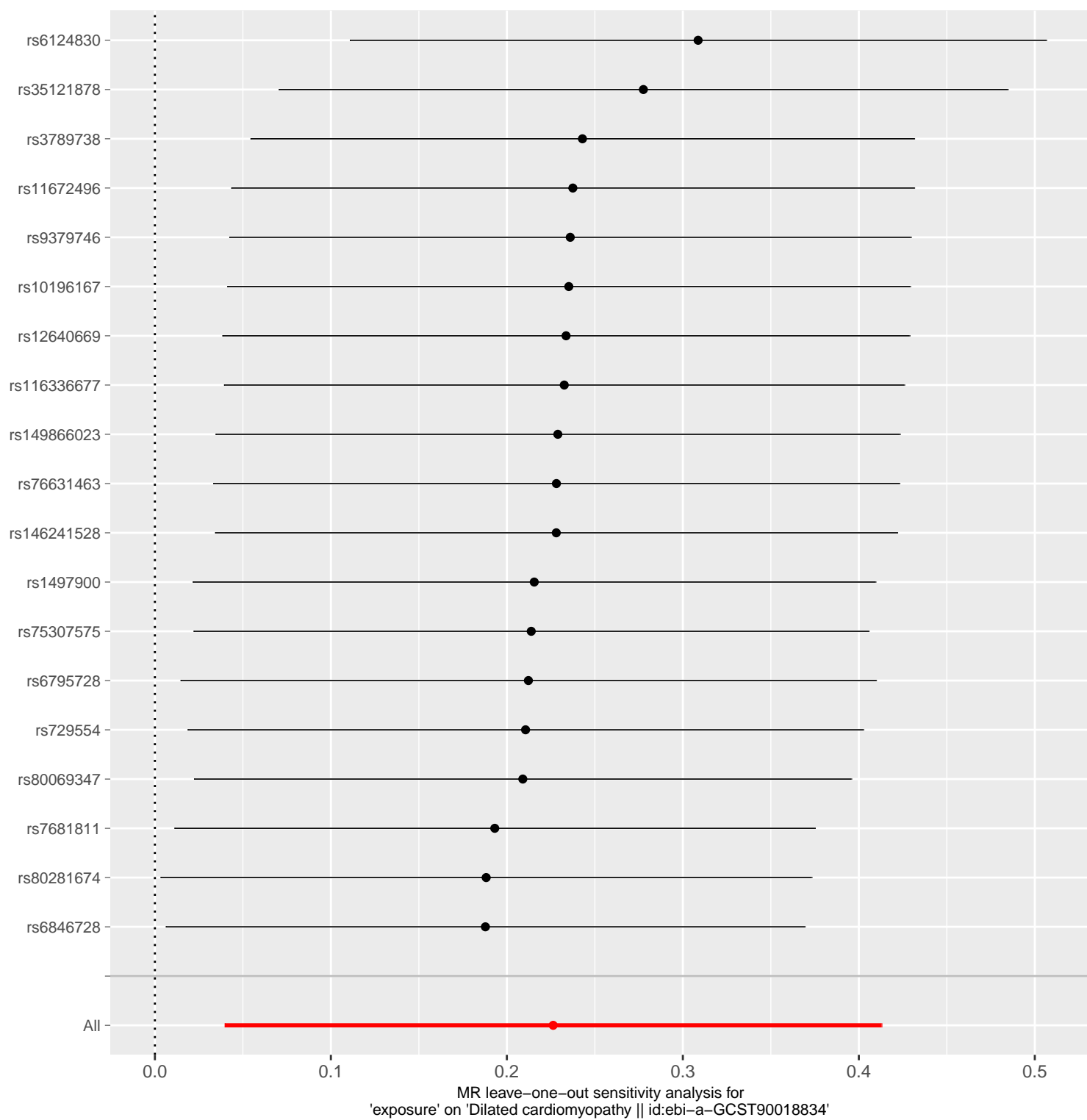

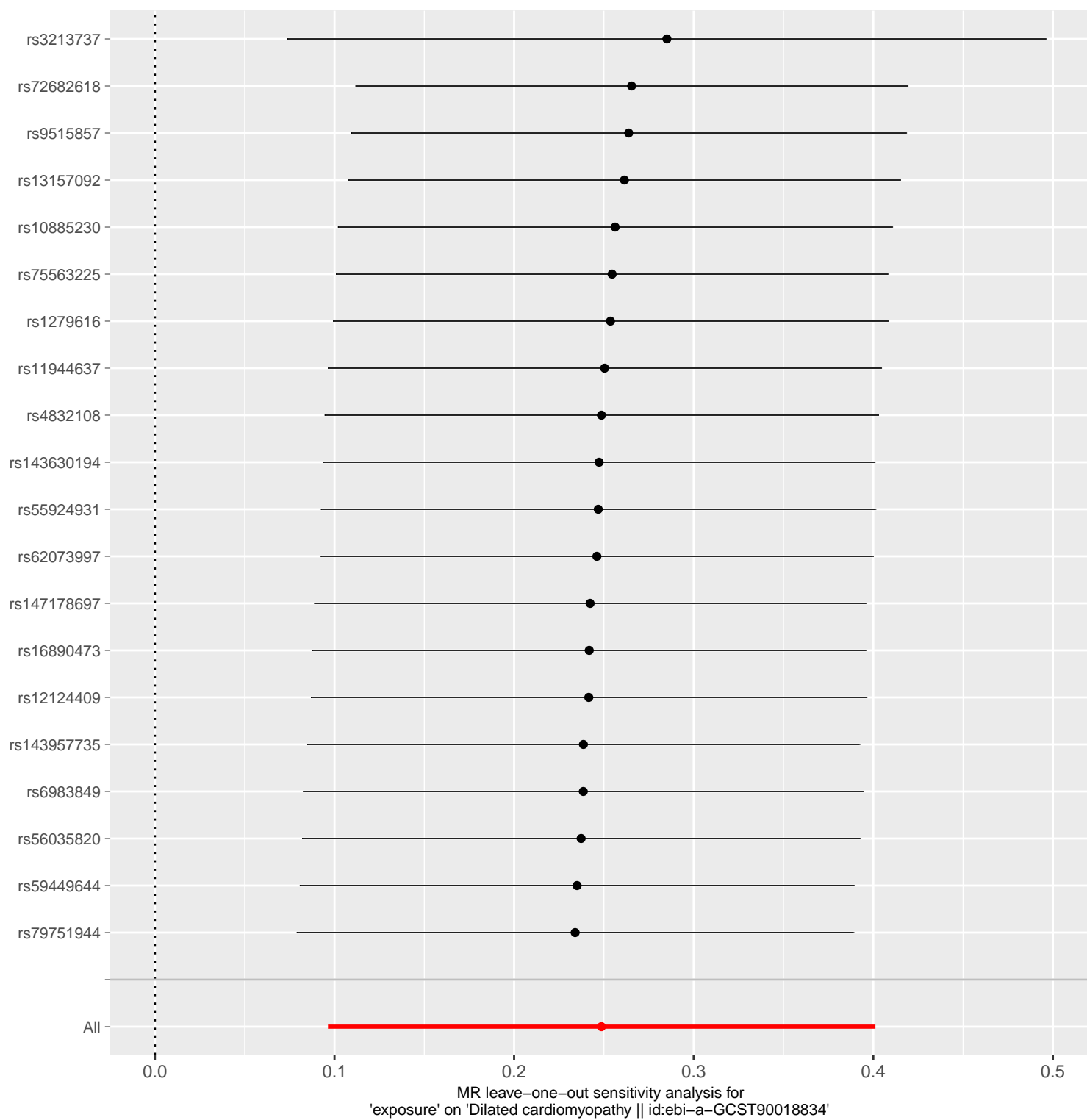

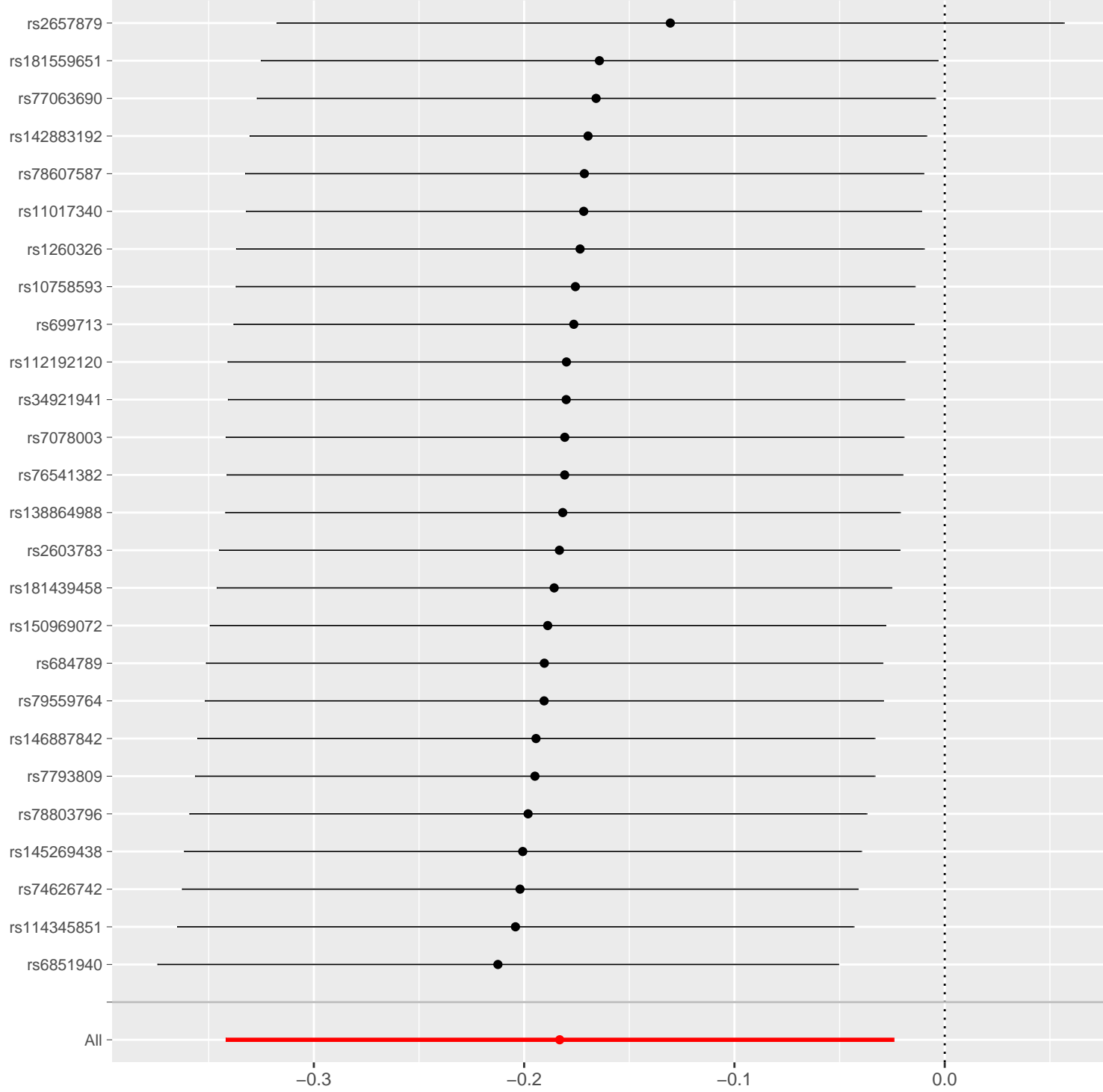

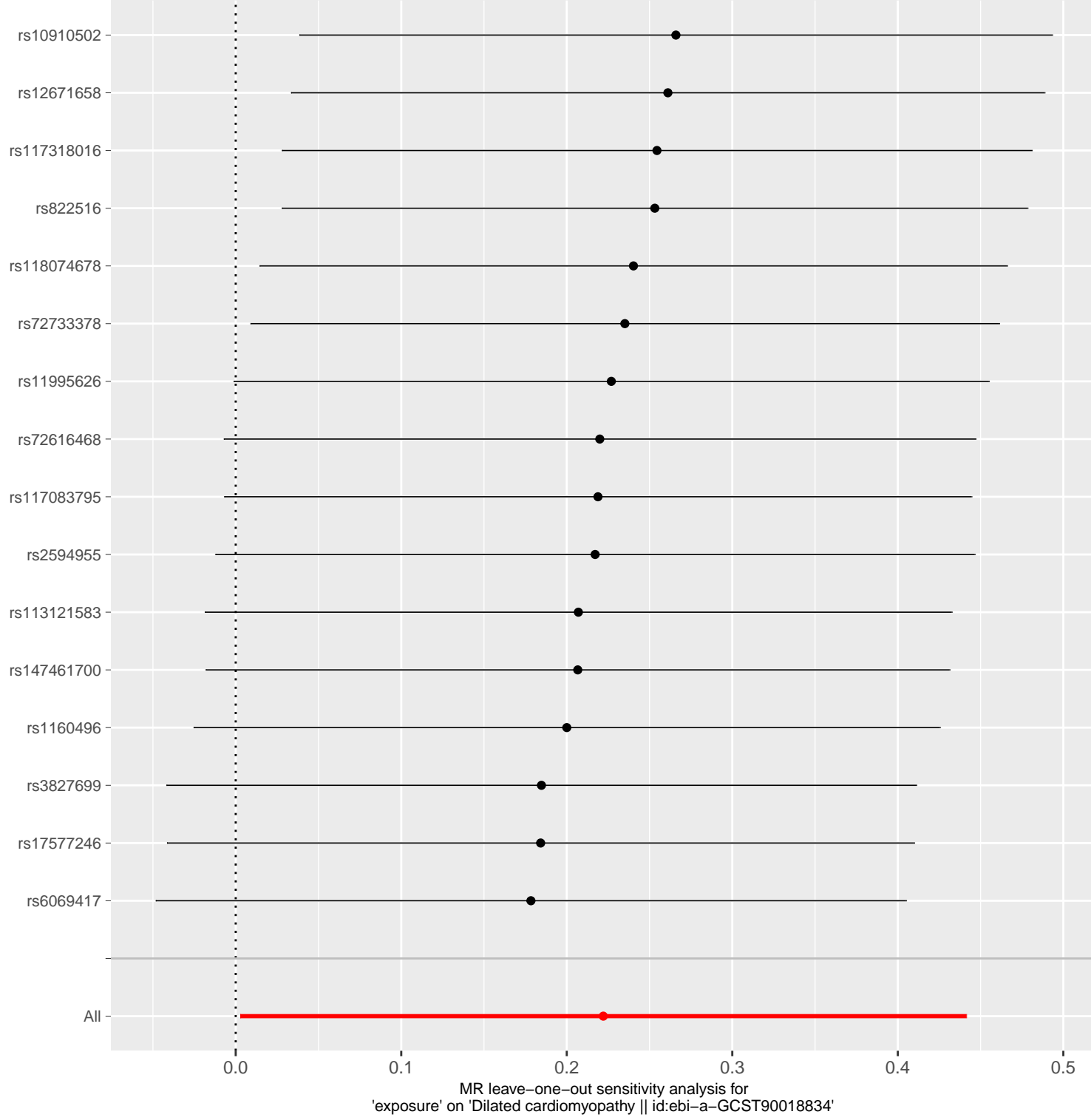

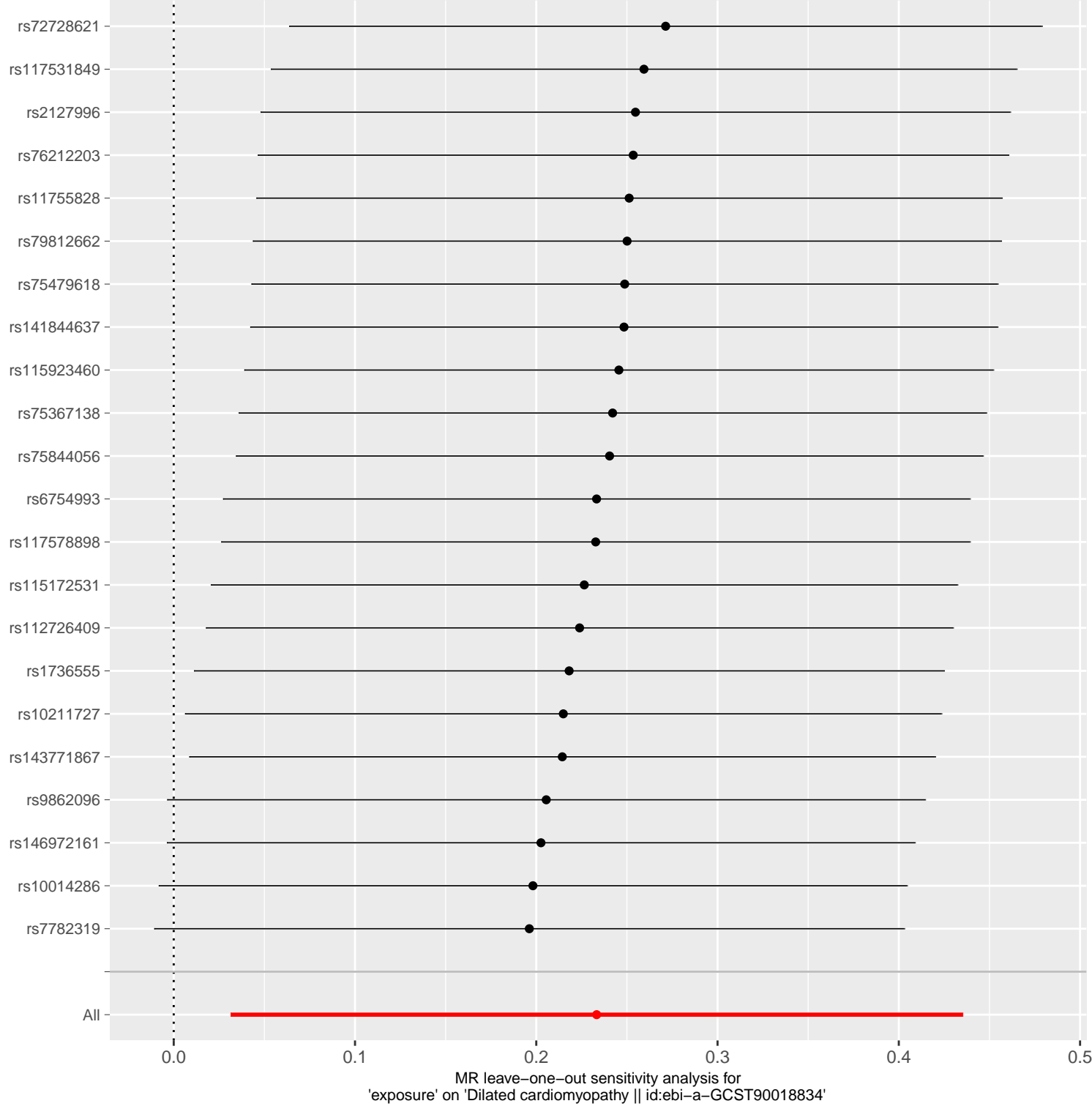

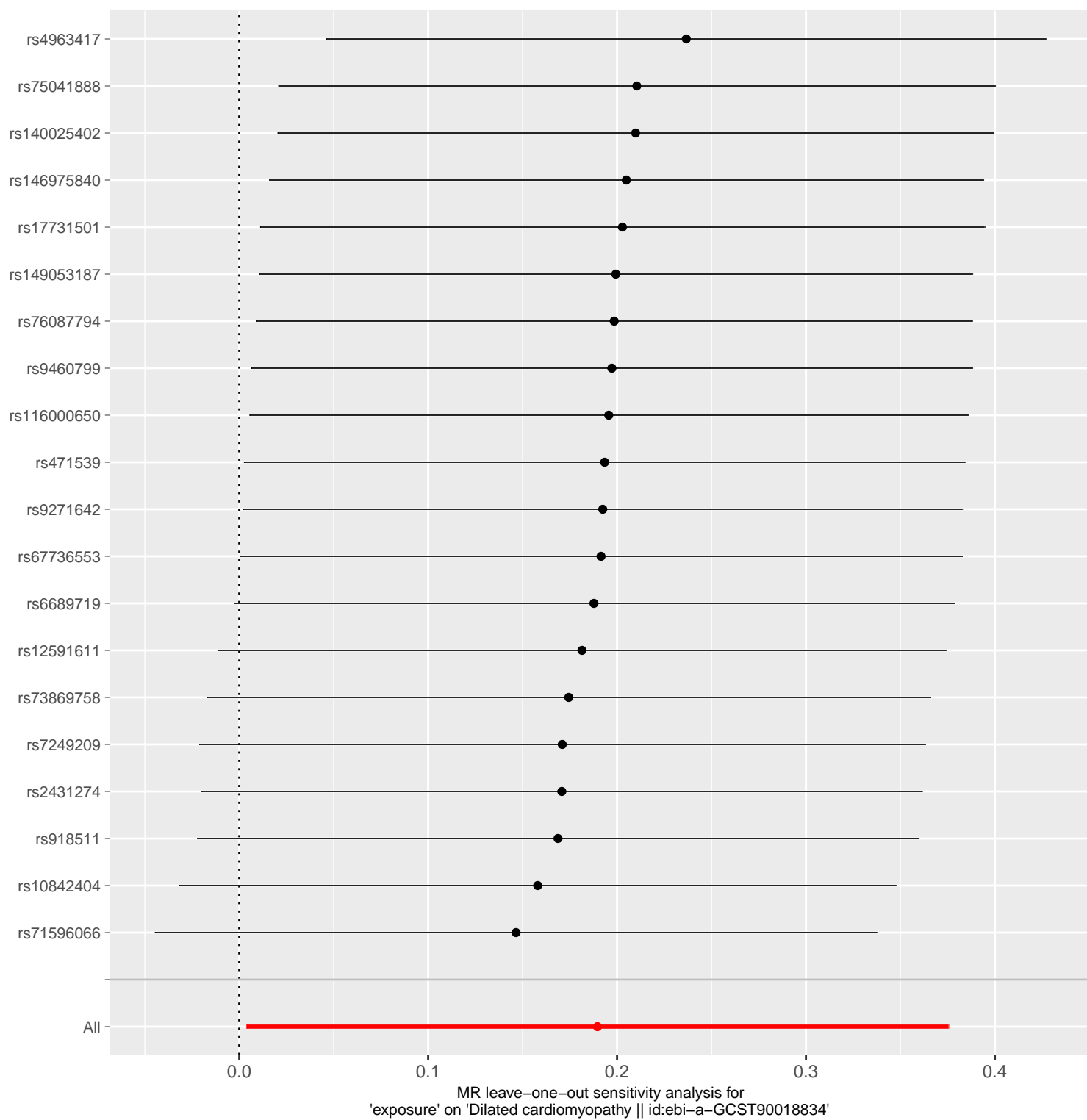

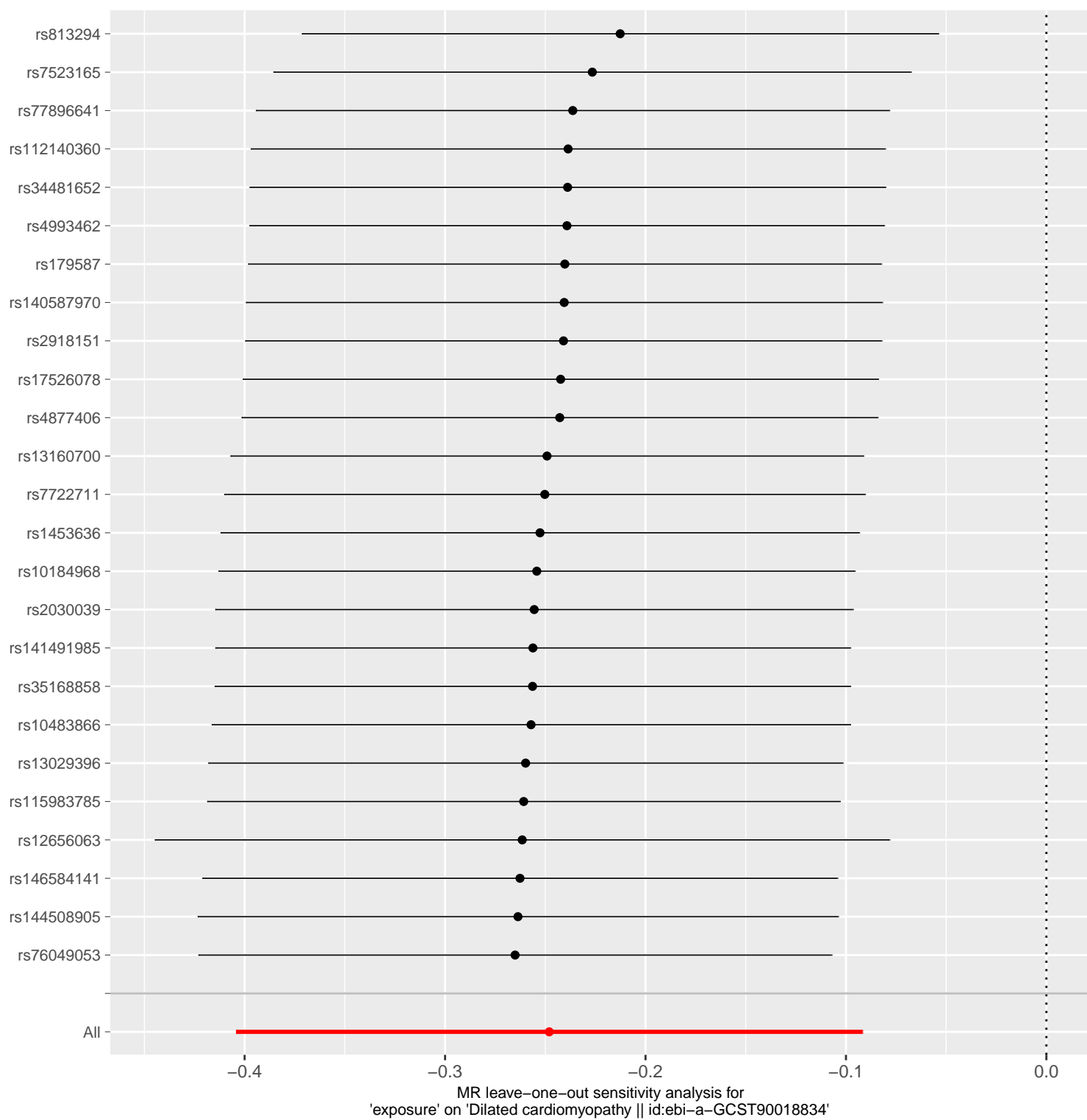

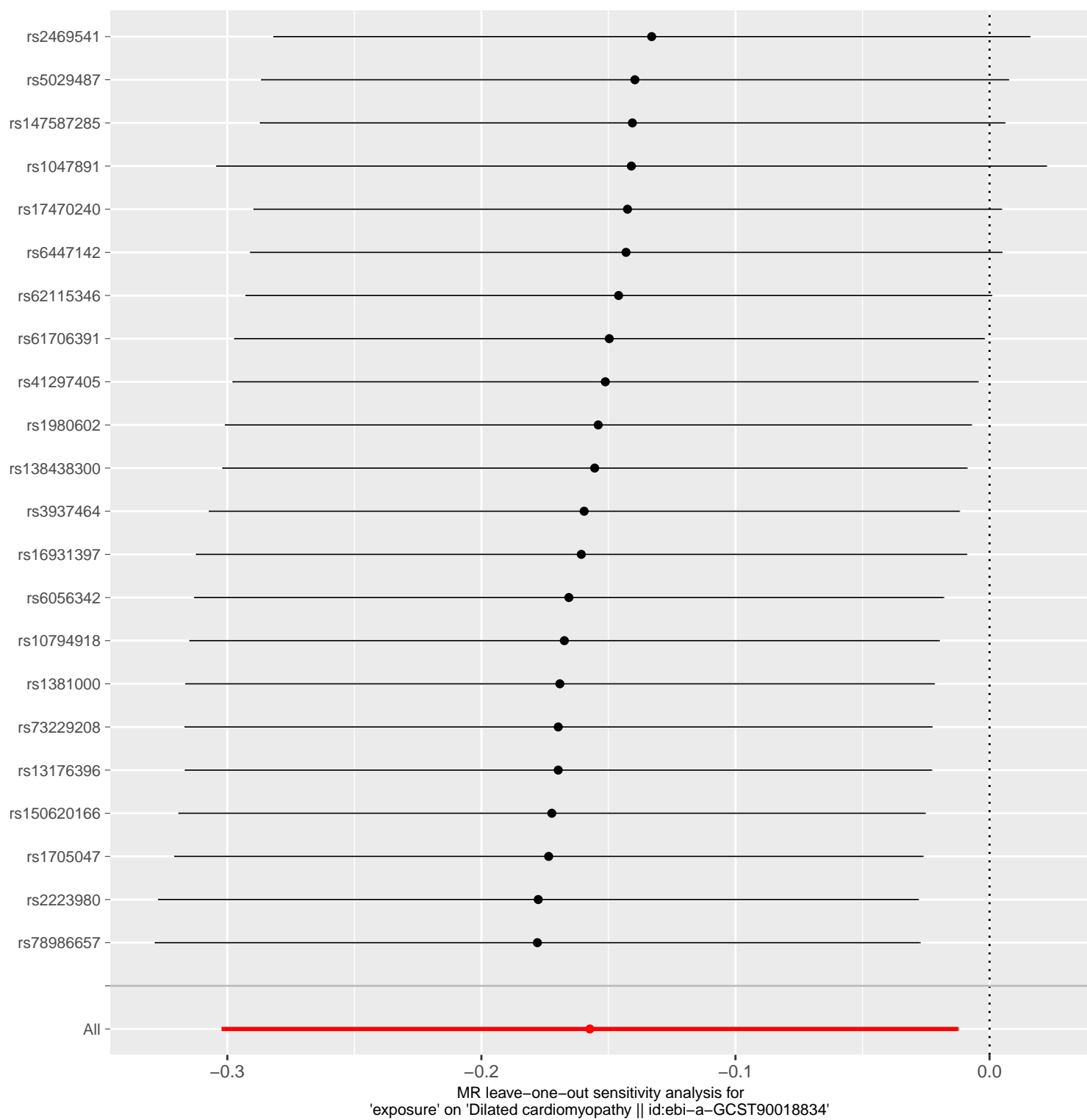

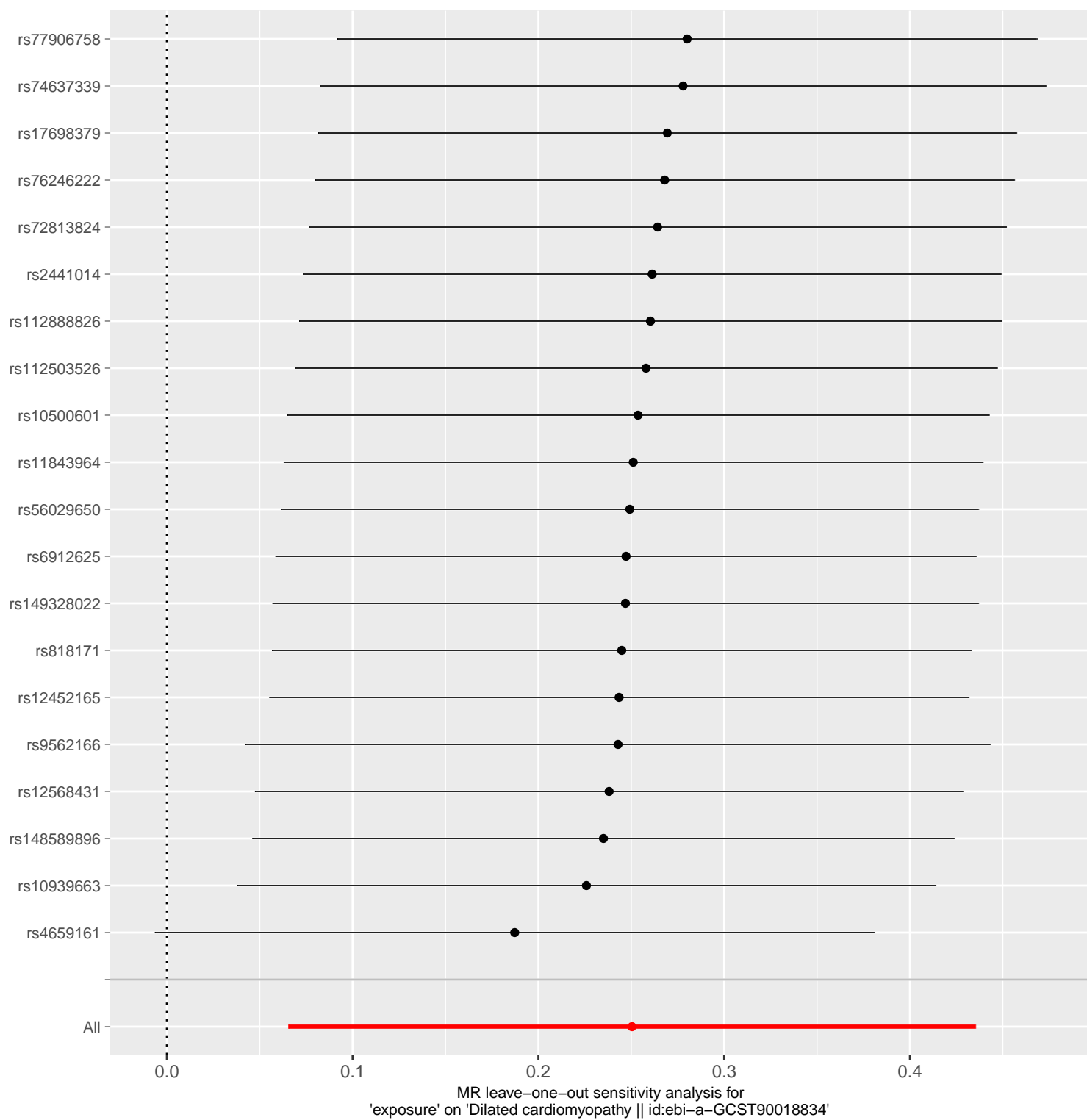

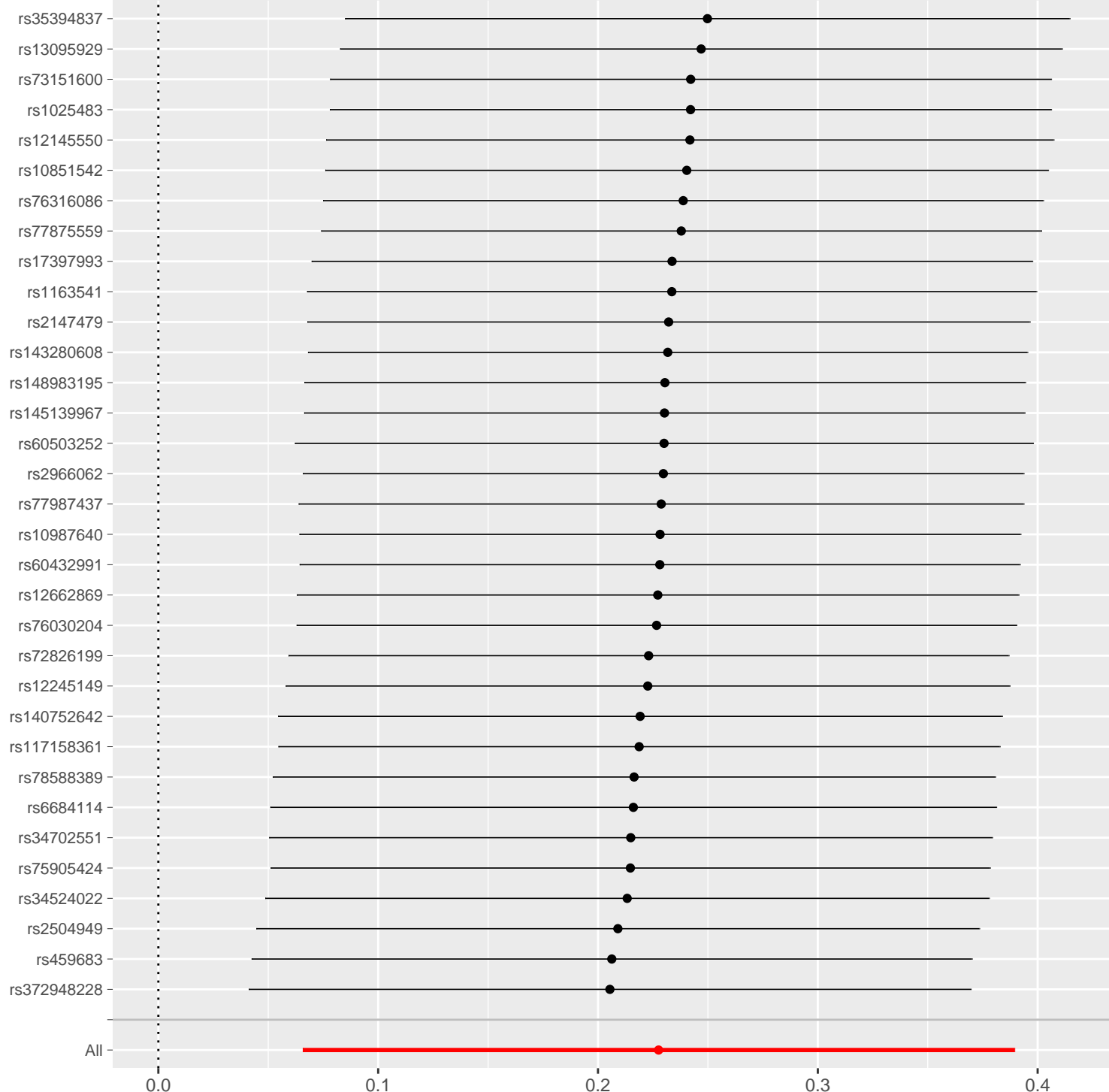

MR leave-one-out sensitivity analysis for  
'exposure' on 'Dilated cardiomyopathy || id:ebi-a-GCST90018834'

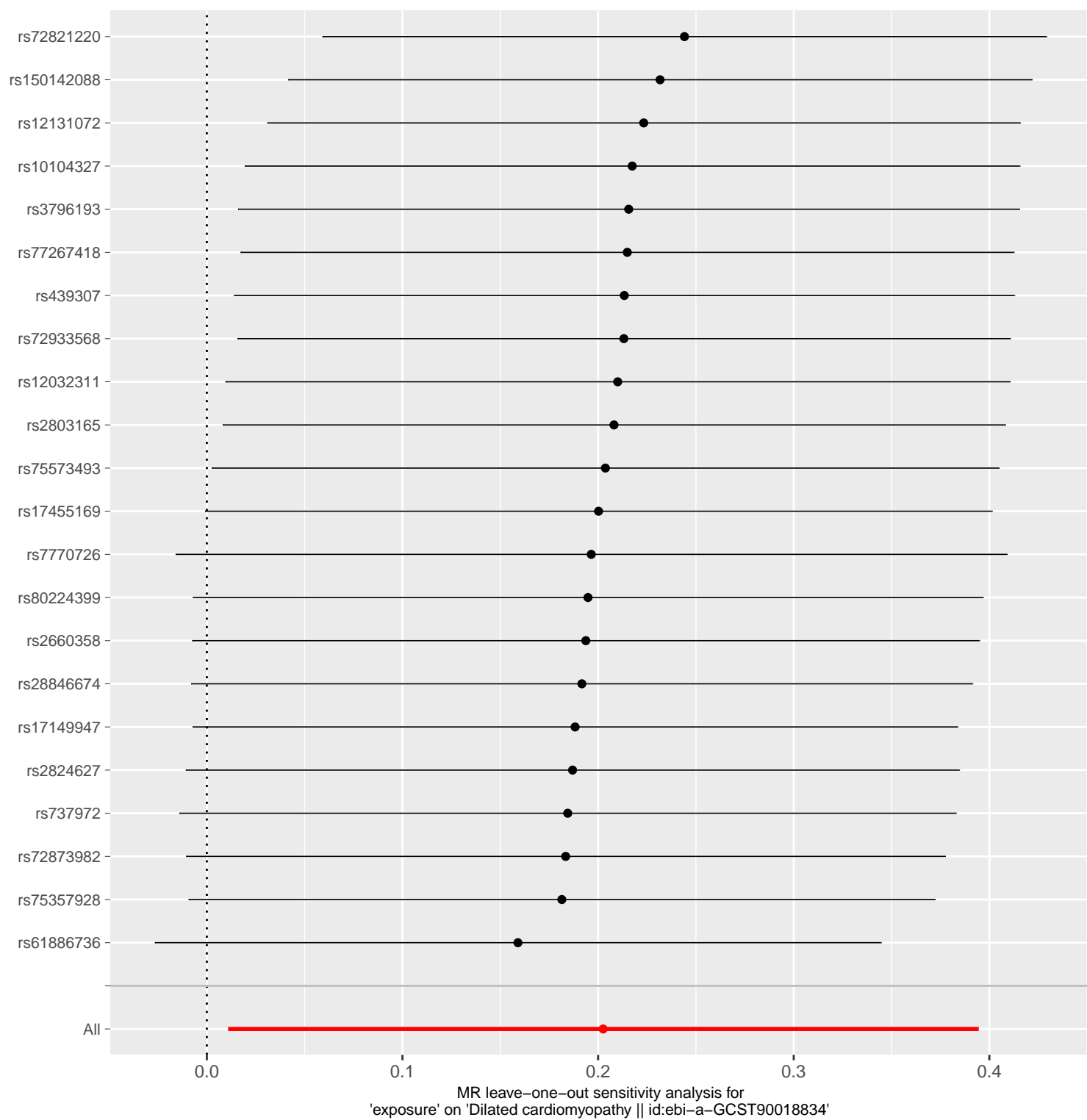

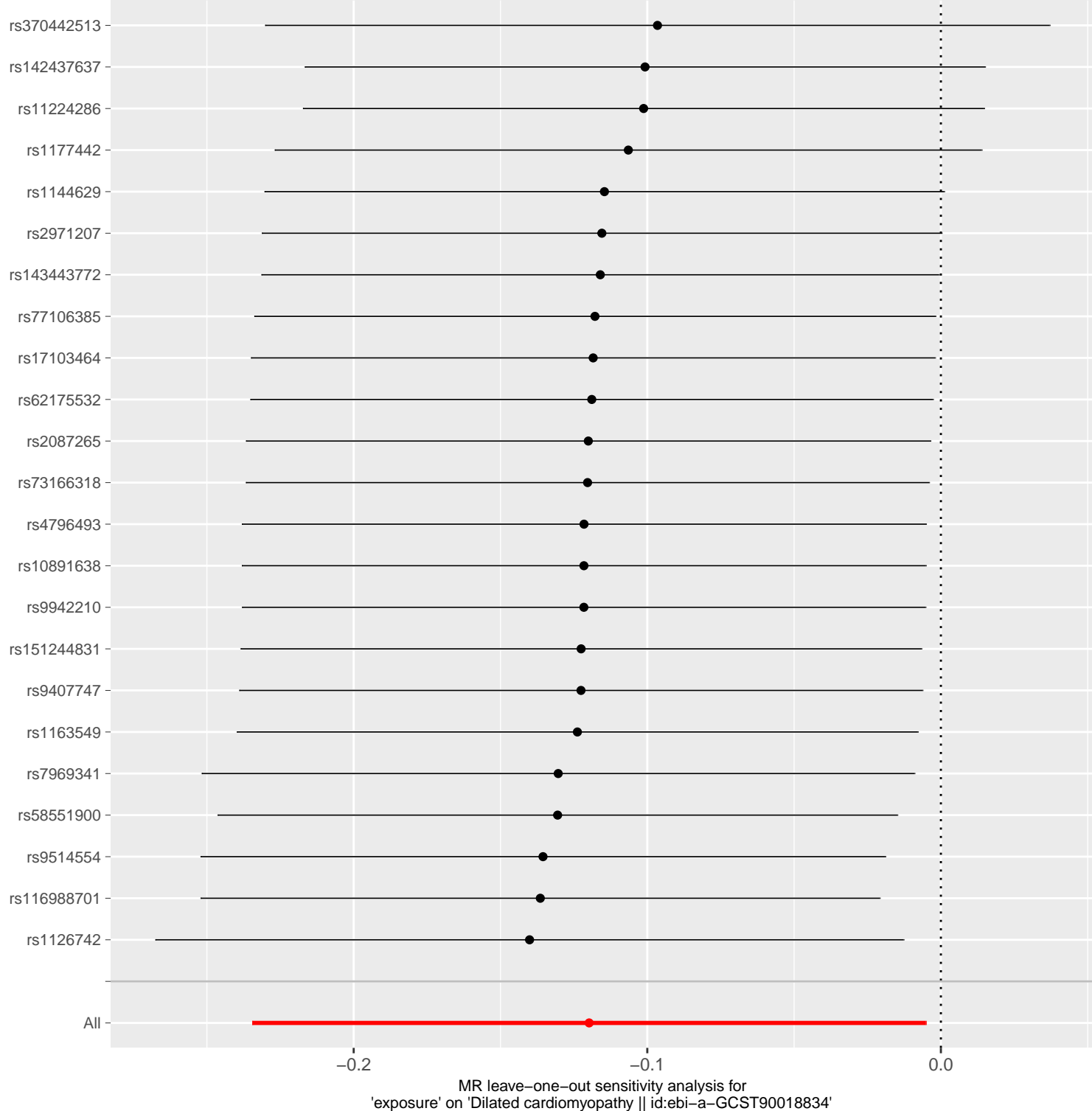

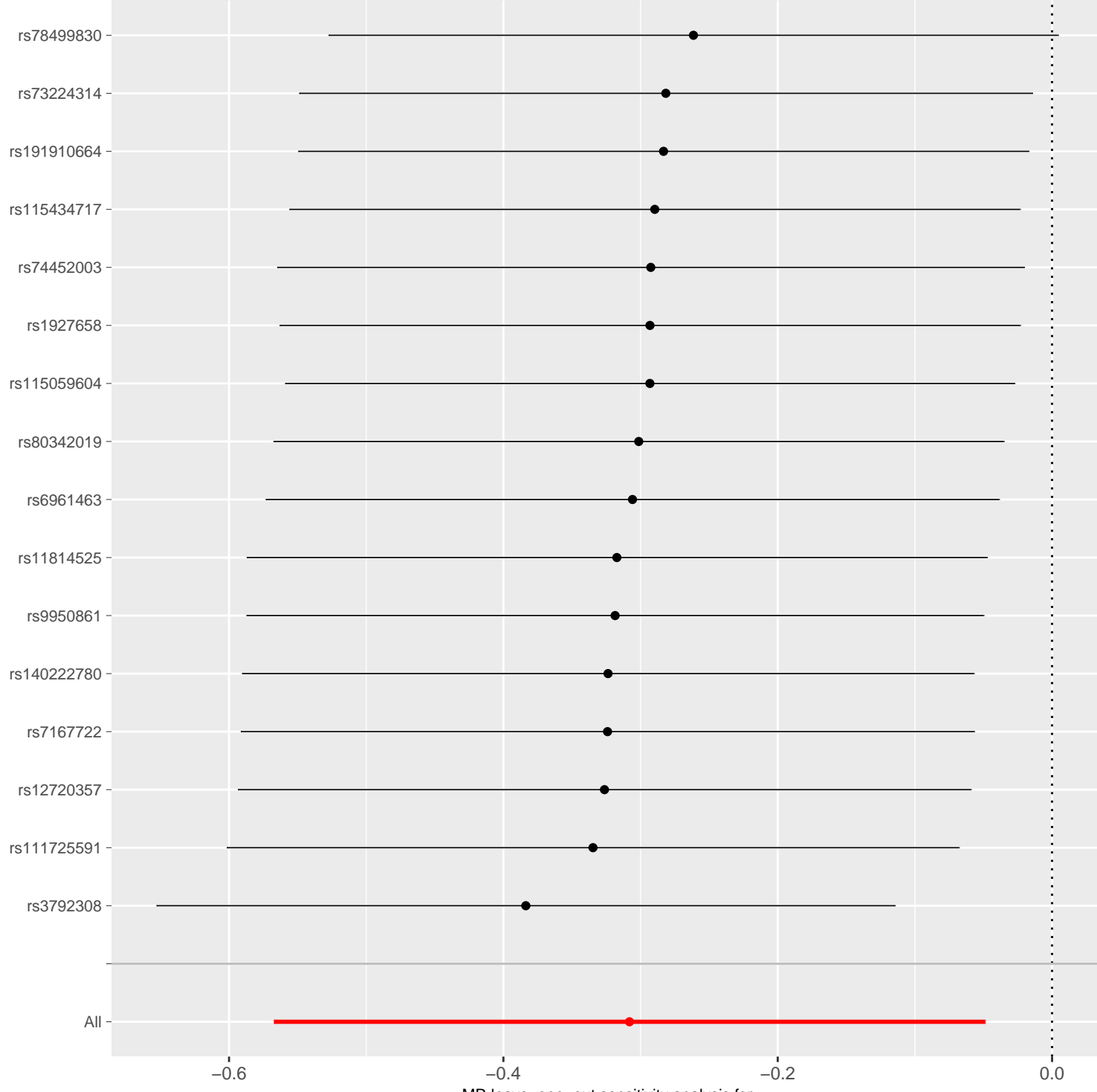

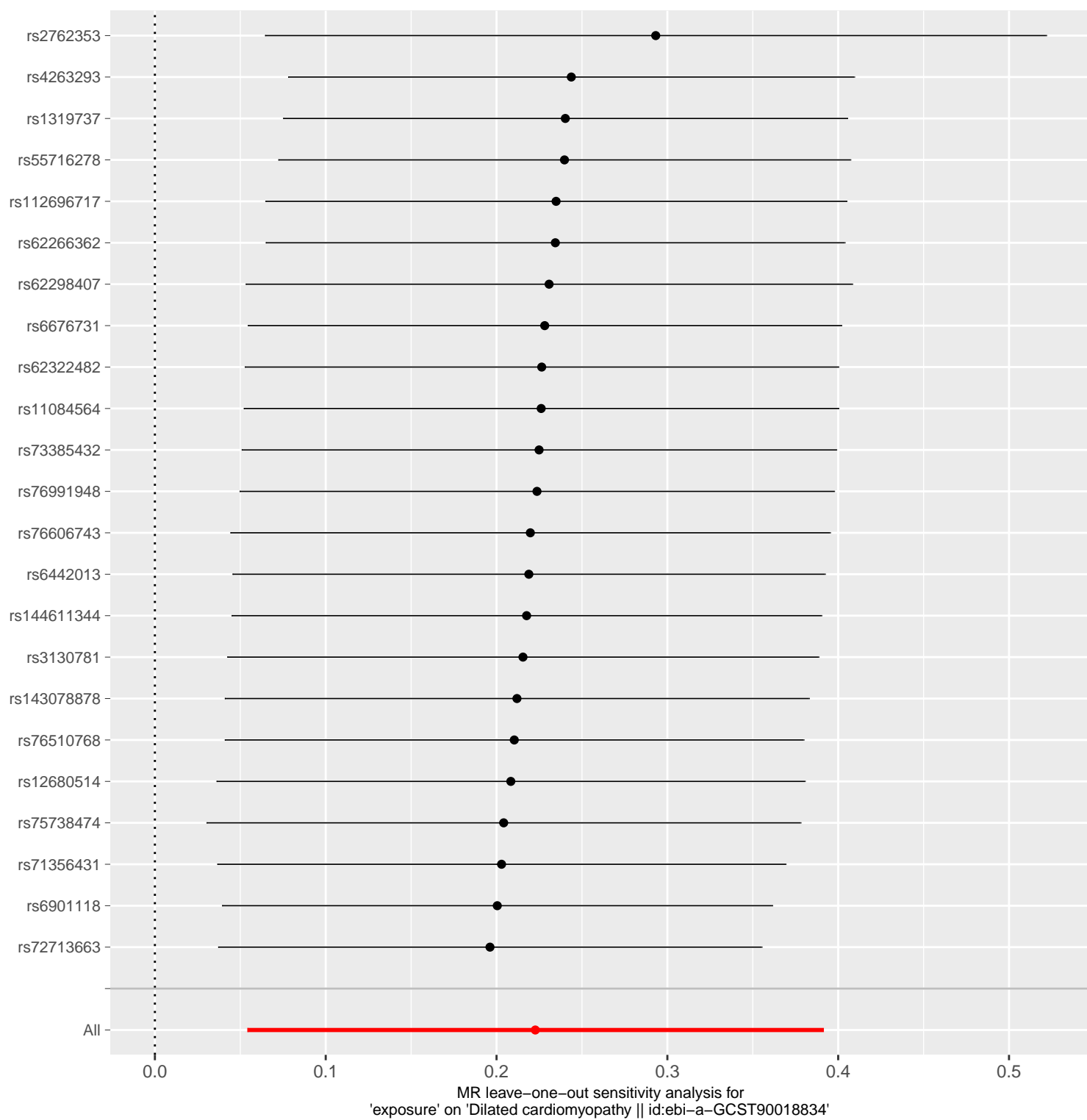

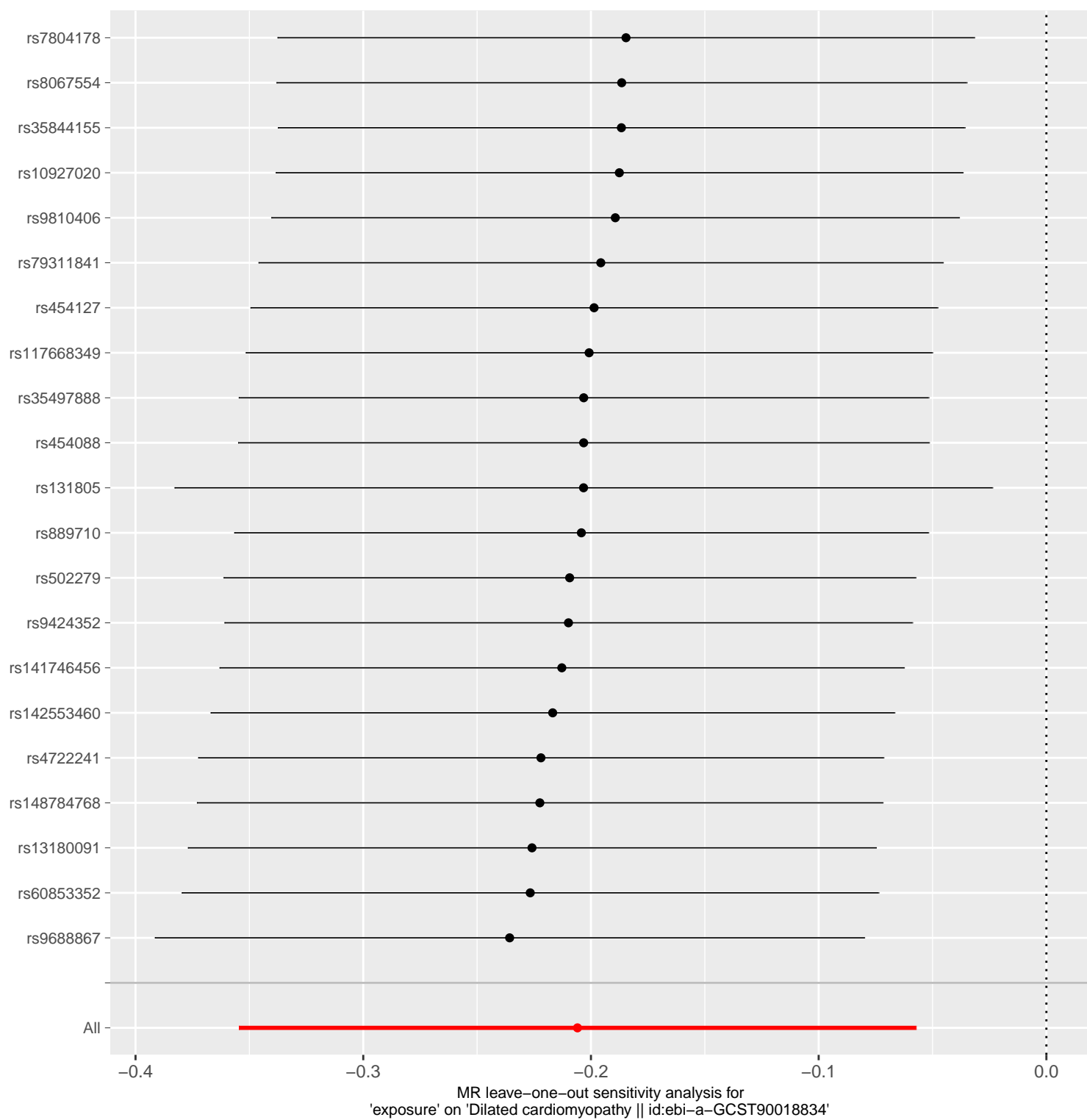

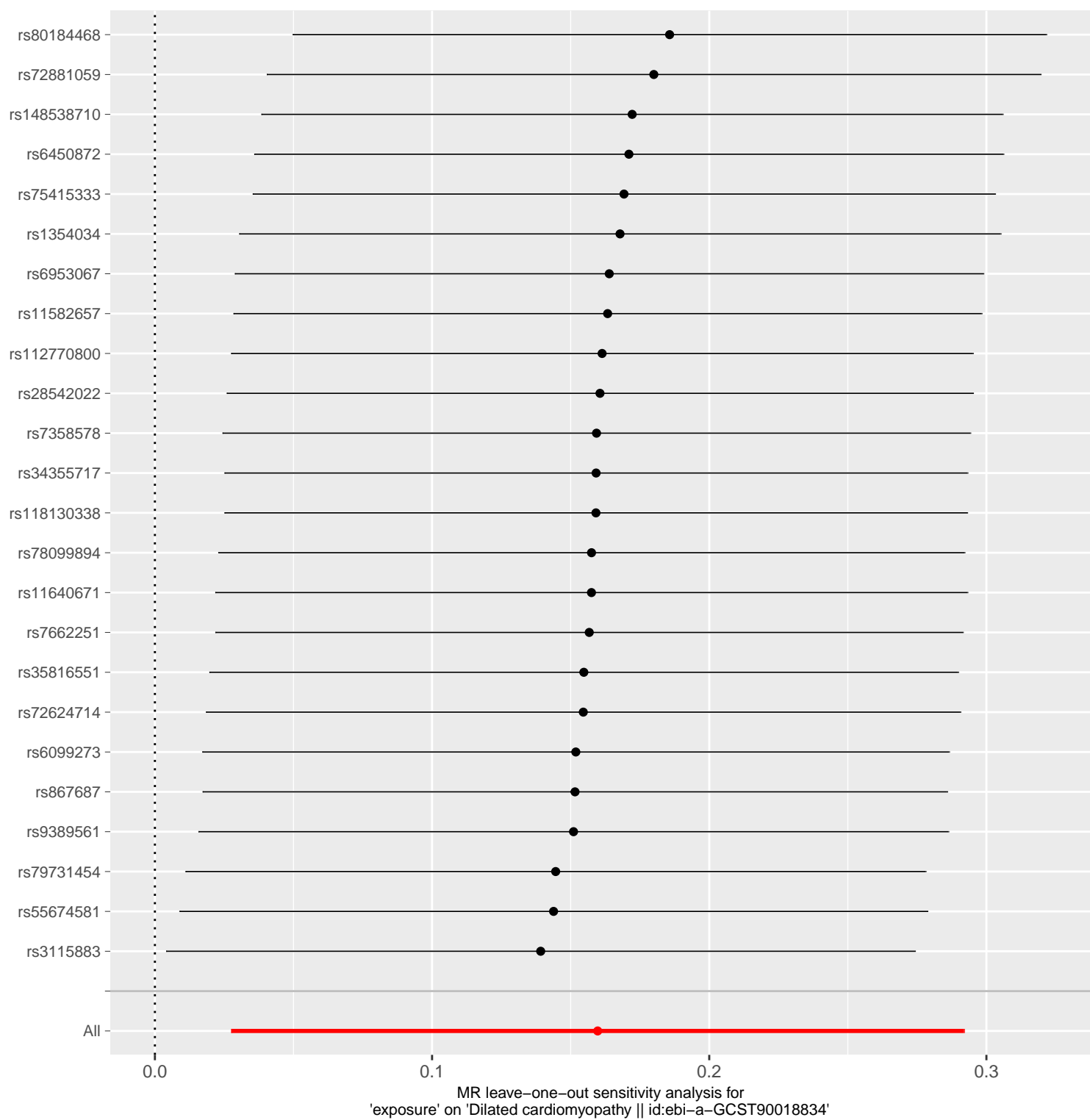

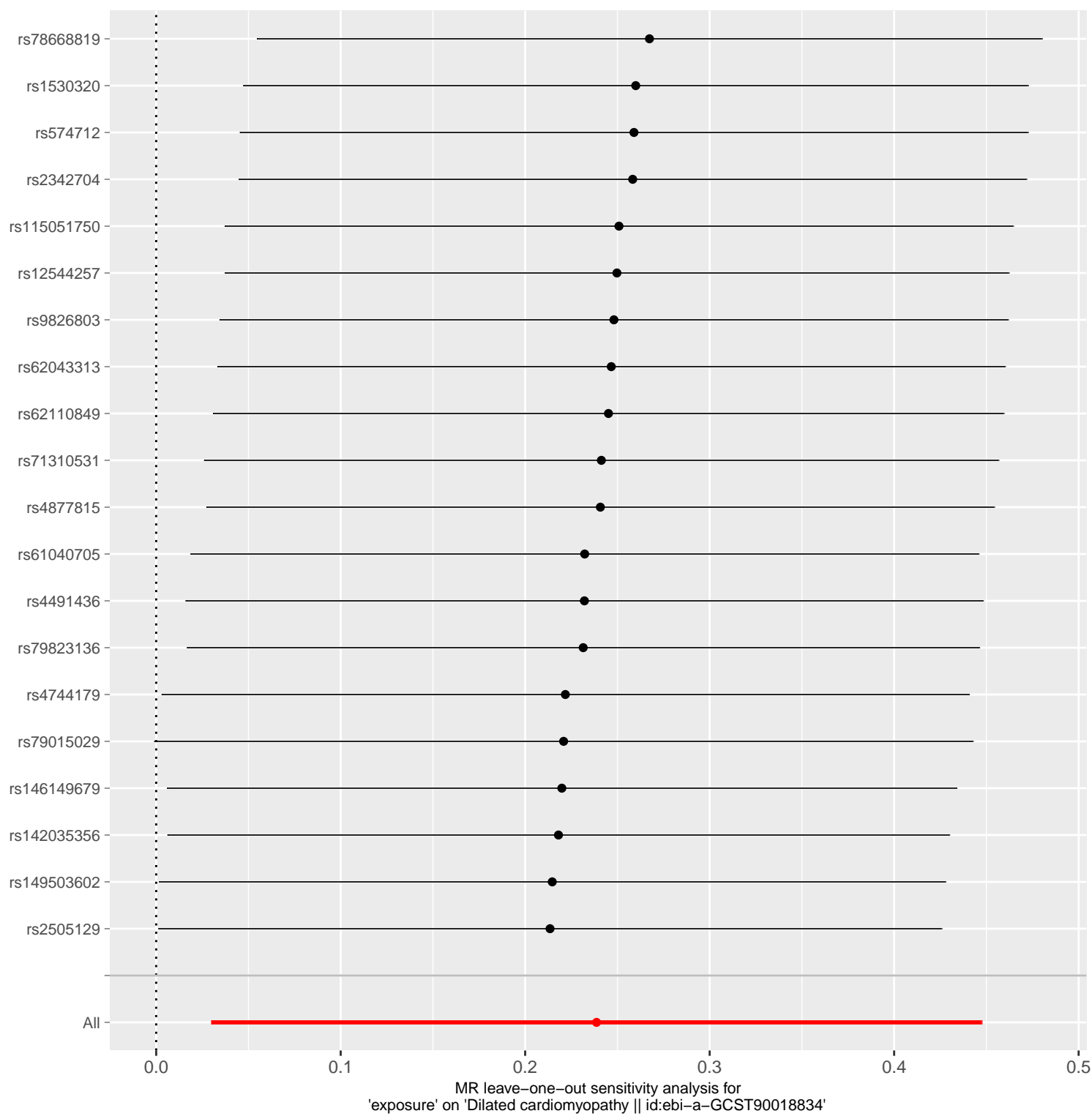

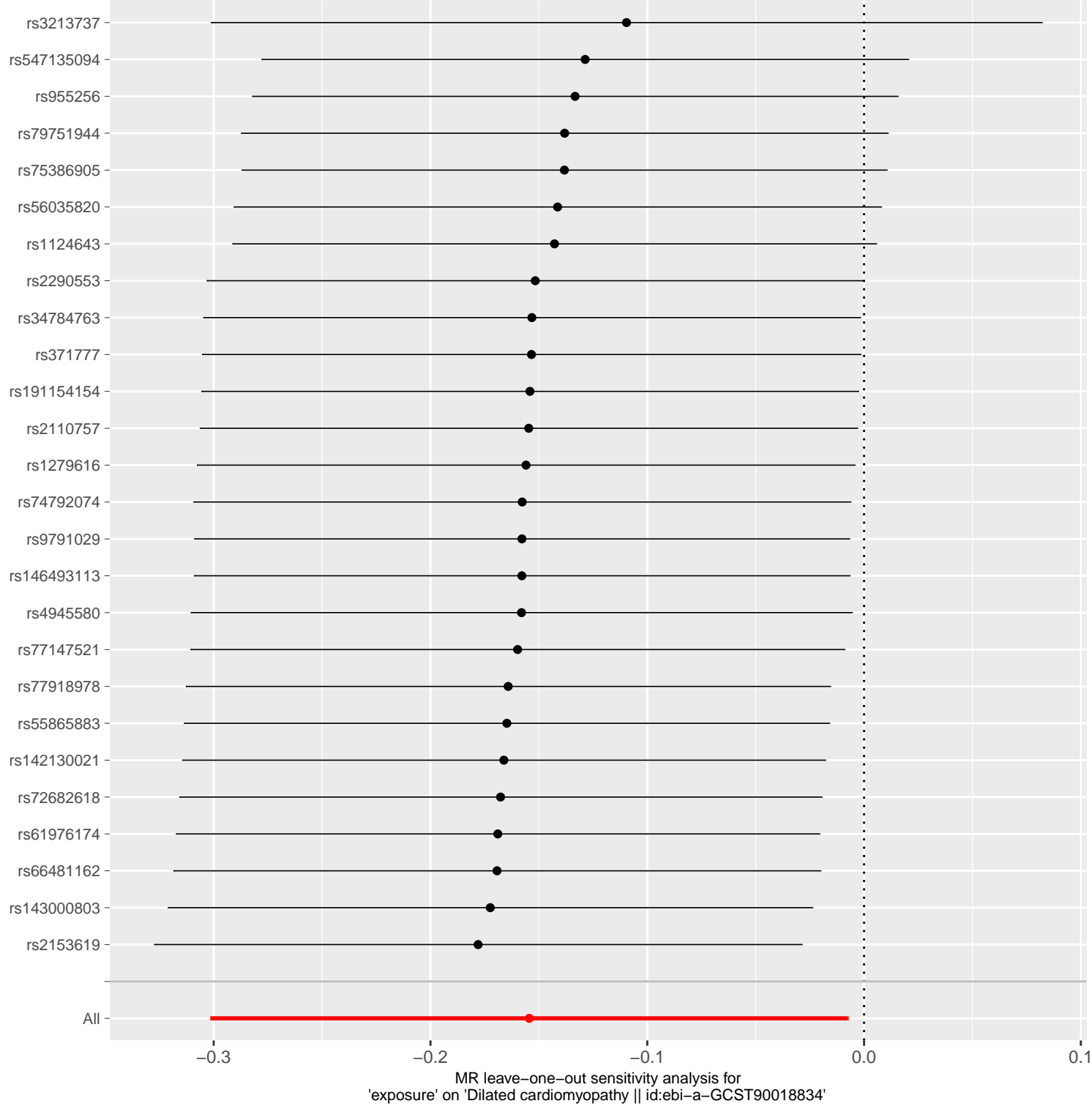

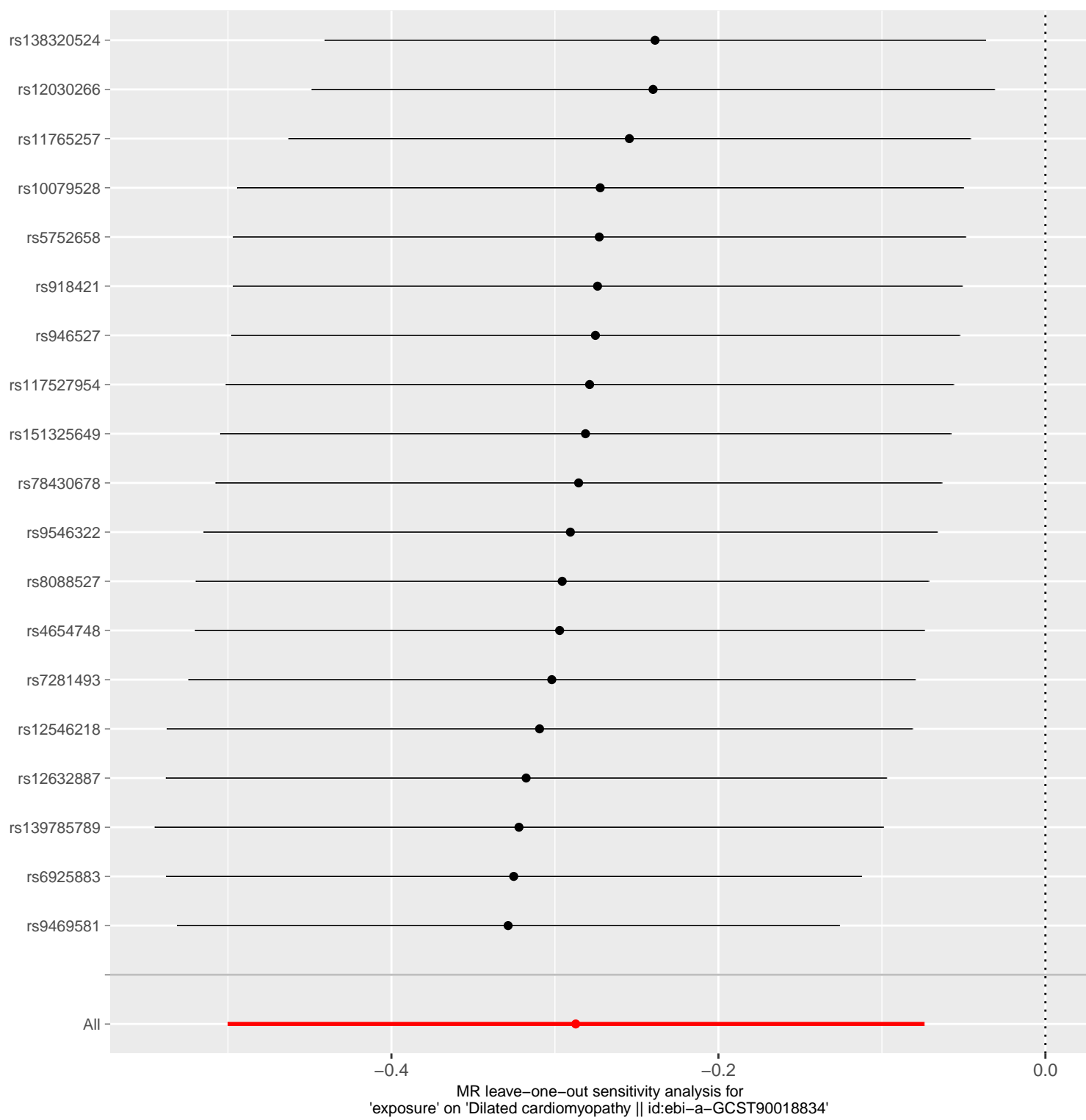

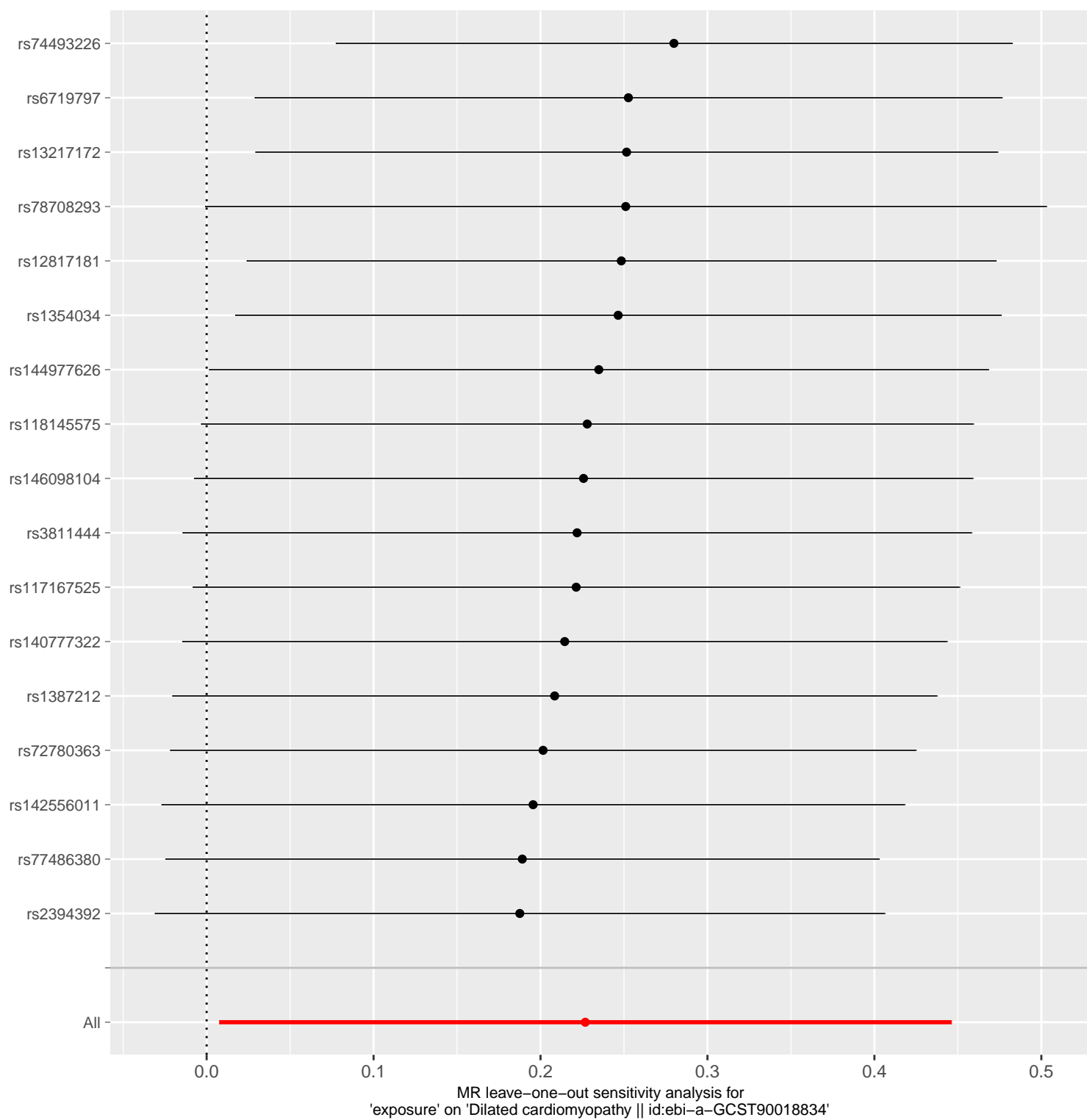

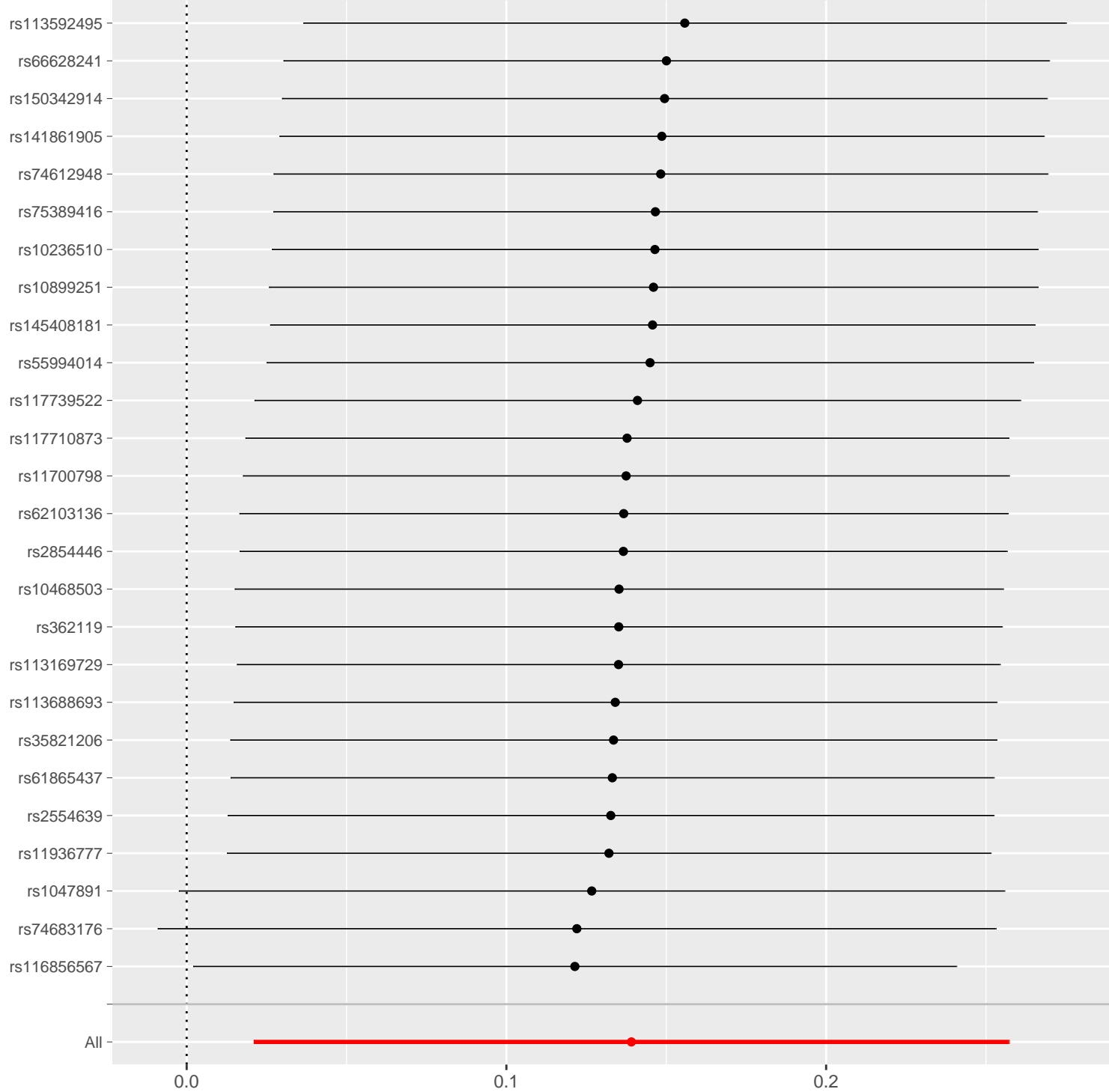

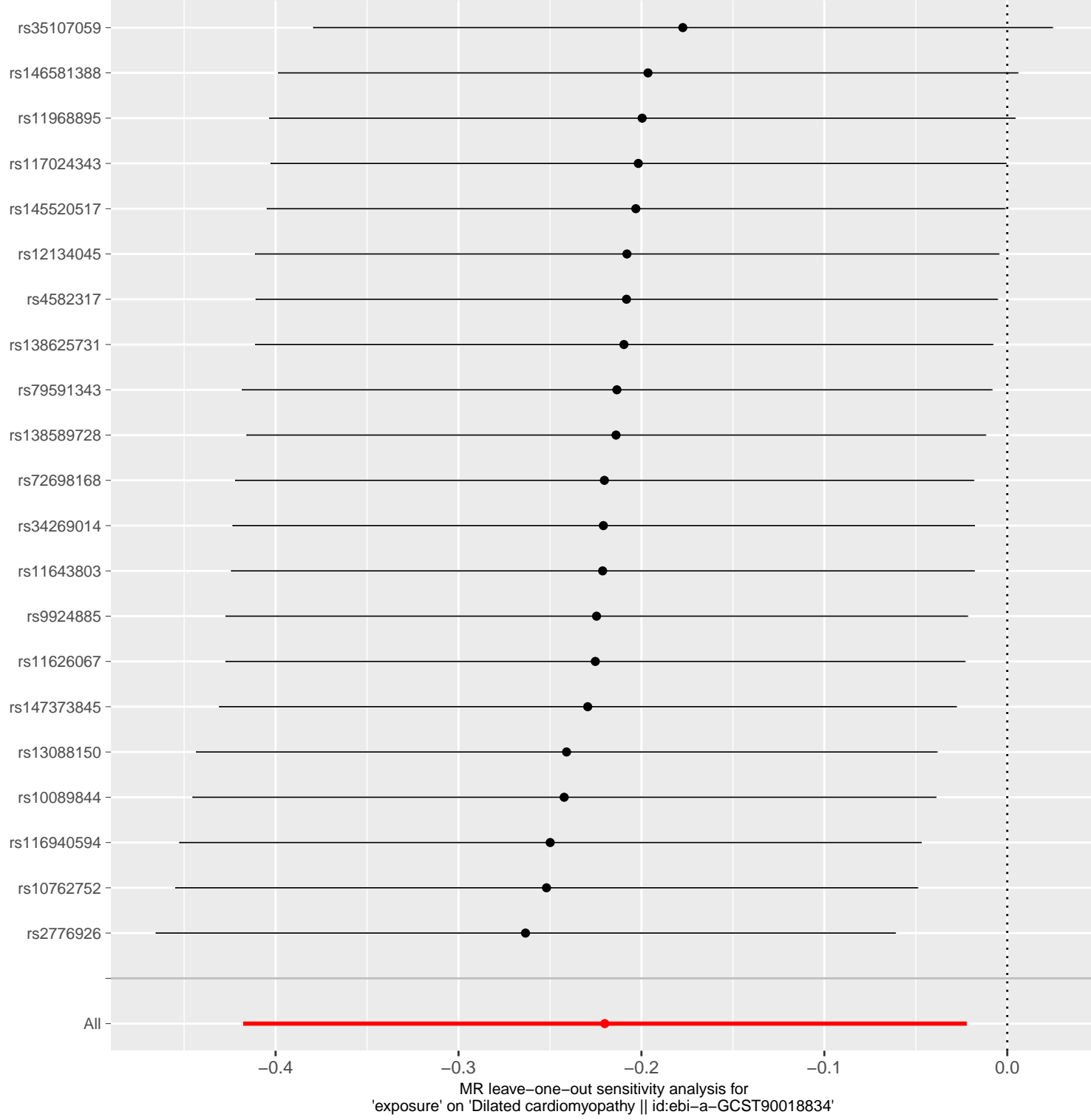

Supplement: Supplementary file 1 [file DataSheet1.pdf]
