# Supplementary material for: Causal association between 1400 metabolites and dilated cardiomyopathy: a bidirectional two-sample Mendelian randomization analysis
Source: Front Endocrinol (Lausanne). 2024 Sep 12;15:1423142. doi: 10.3389/fendo.2024.1423142 (PMC11424463; doi:10.3389/fendo.2024.1423142)
Supplement: Supplementary file 2 [file DataSheet2.pdf]

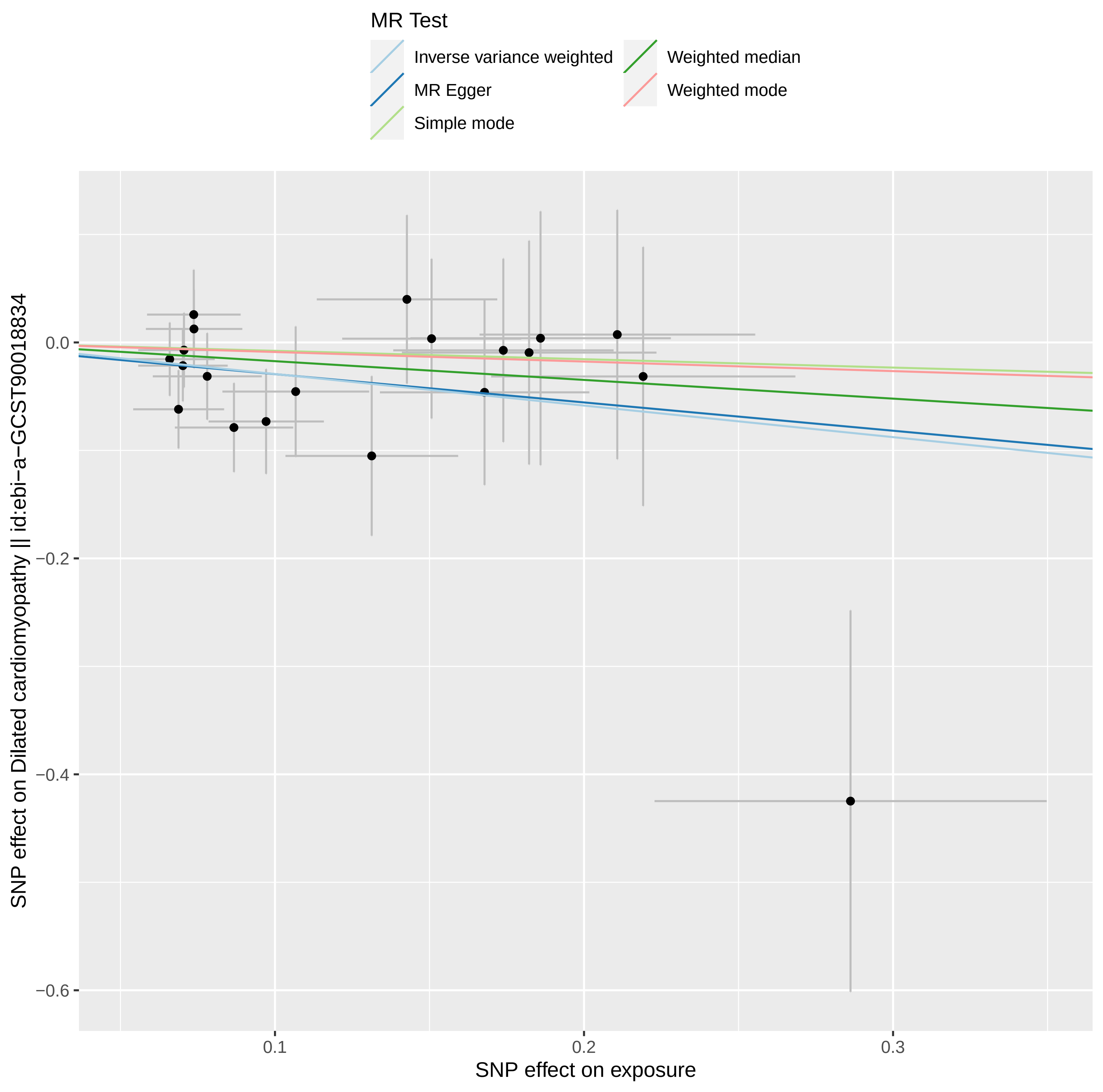
MR Test
Inverse variance weighted
MR Egger
Simple mode
Weighted median
Weighted mode
0.0
−0.2
−0.4
−0.6
0.1
0.2
0.3
SNP effect on Dilated cardiomyopathy || id:ebi−a−GCST90018834
SNP effect on exposure

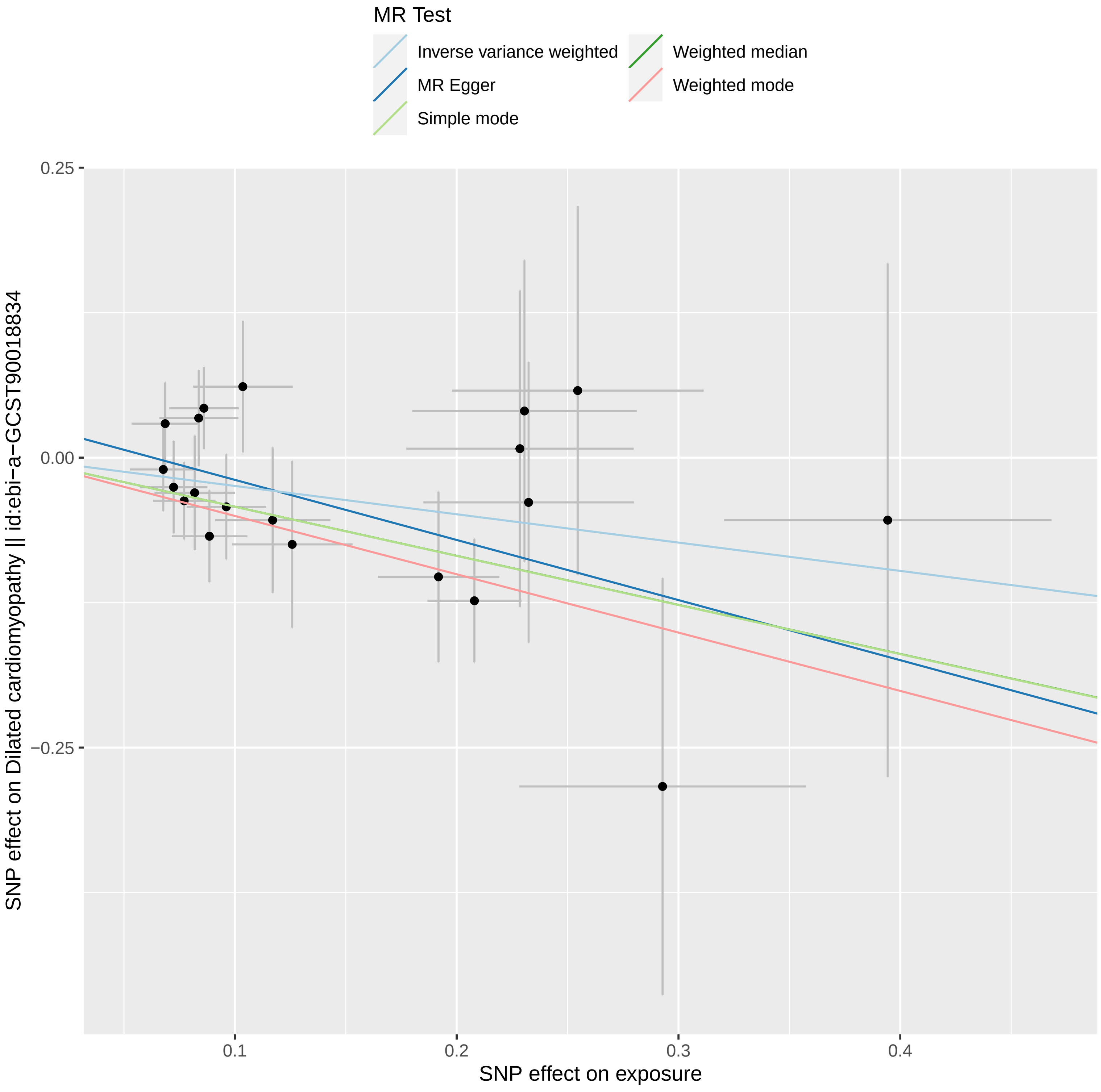
MR Test
Inverse variance weighted
MR Egger
Simple mode
Weighted median
Weighted mode
0.25
0.00
−0.25
0.1
0.2
0.3
0.4
SNP effect on Dilated cardiomyopathy || id:ebi−a−GCST90018834
SNP effect on exposure

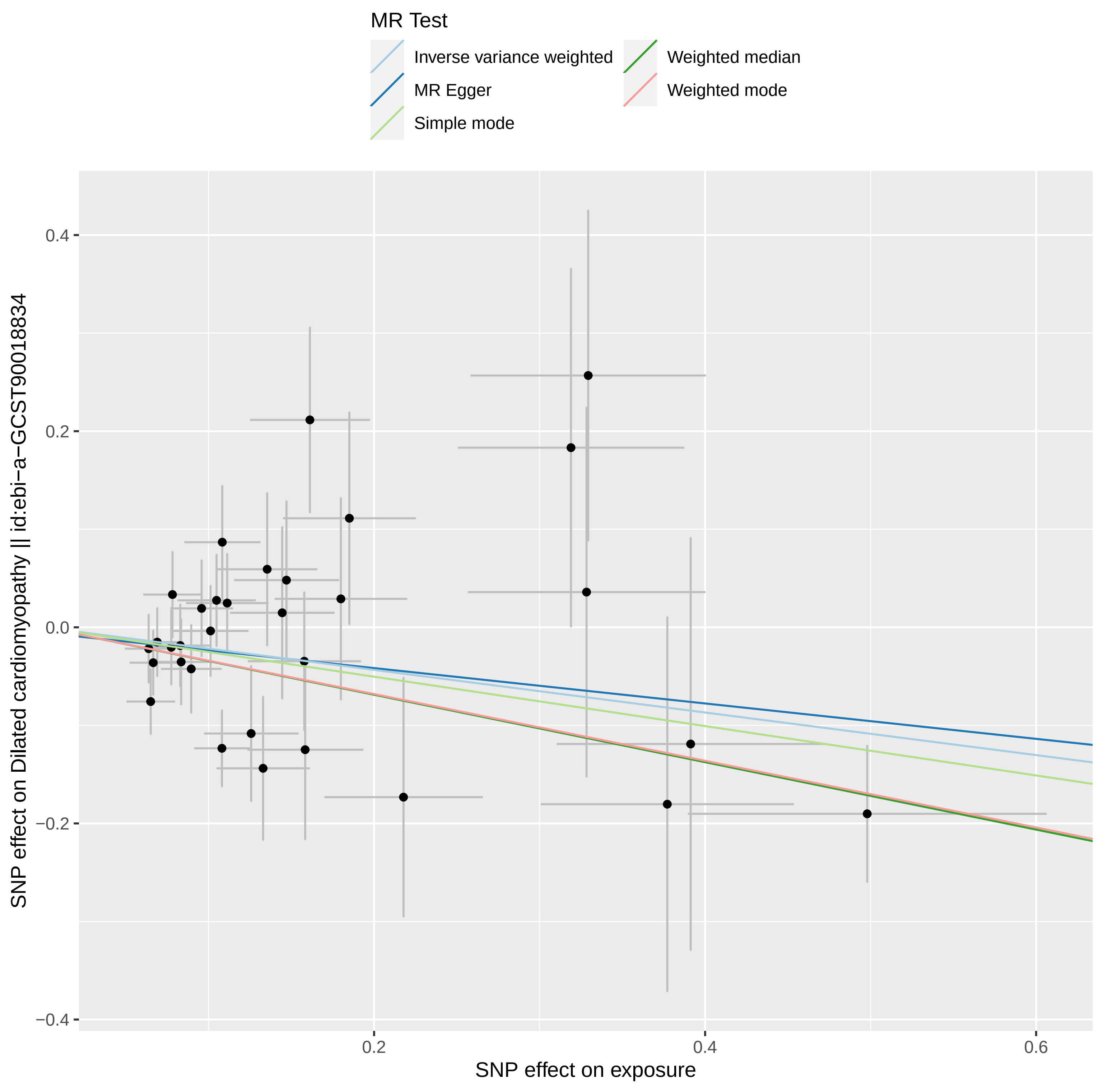
MR Test
Inverse variance weighted
MR Egger
Simple mode
Weighted median
Weighted mode
SNP effect on Dilated cardiomyopathy || id:ebi−a−GCST90018834
SNP effect on exposure
0.4
0.2
0.0
−0.2
−0.4
0.2
0.4
0.6

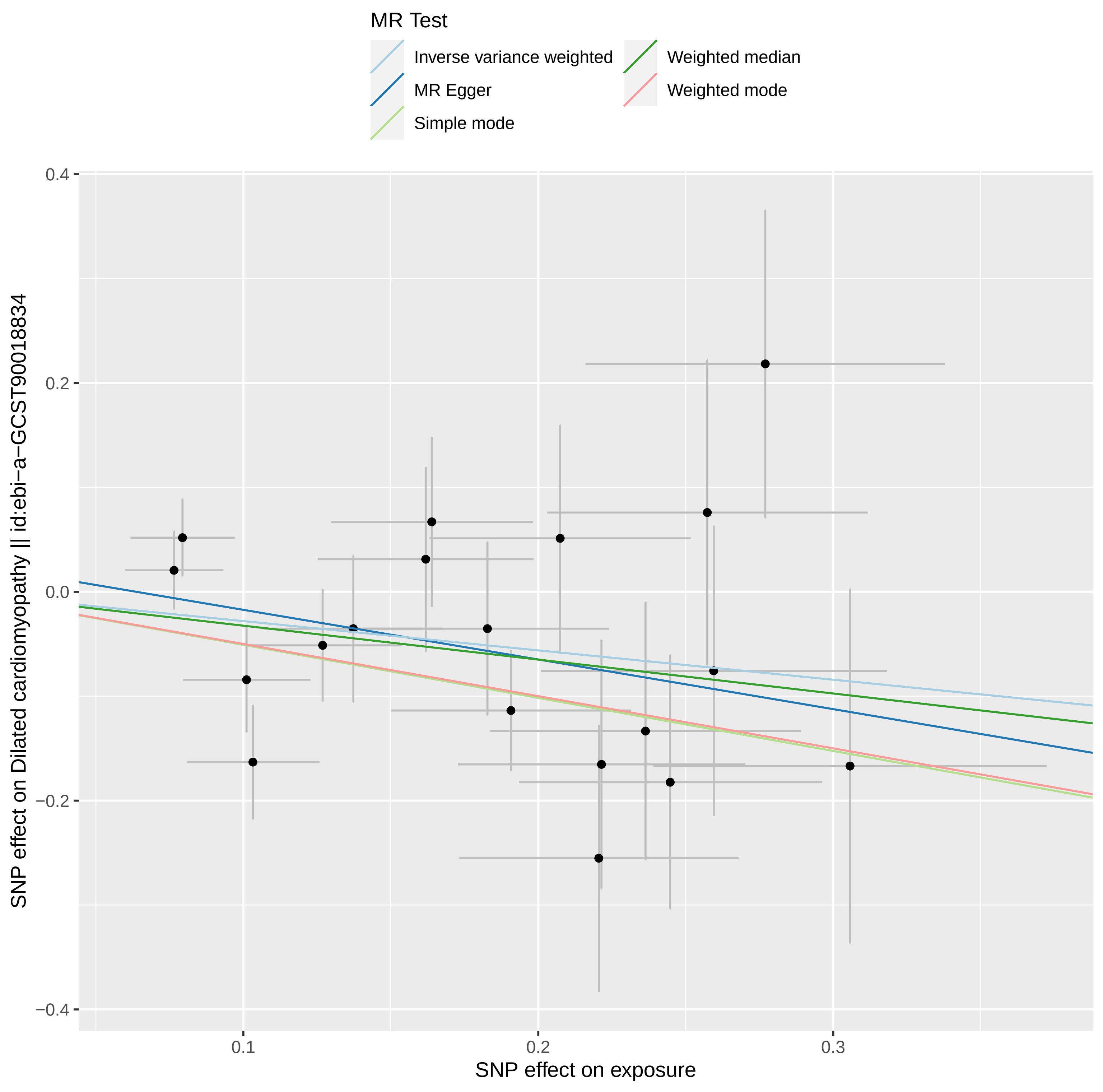

MR Test
Inverse variance weighted
MR Egger
Simple mode
Weighted median
Weighted mode
SNP effect on Dilated cardiomyopathy || id:ebi−a−GCST90018834
SNP effect on exposure
0.4
0.2
0.0
−0.2
−0.4
0.1
0.2
0.3

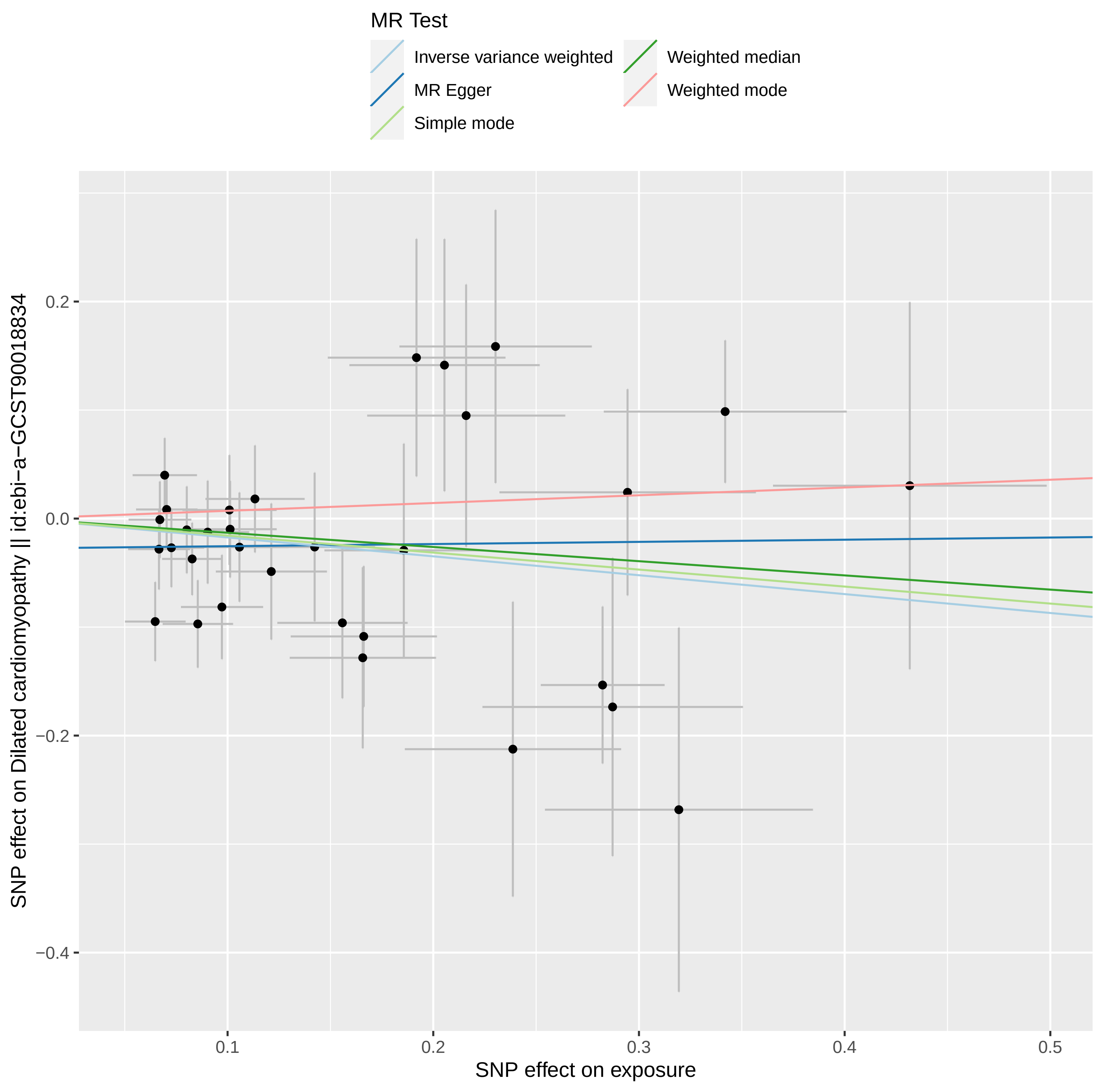

MR Test
Inverse variance weighted
MR Egger
Simple mode
Weighted median
Weighted mode
0.2
0.0
−0.2
−0.4
SNP effect on Dilated cardiomyopathy || id:ebi−a−GCST90018834
0.1
0.2
0.3
0.4
0.5
SNP effect on exposure

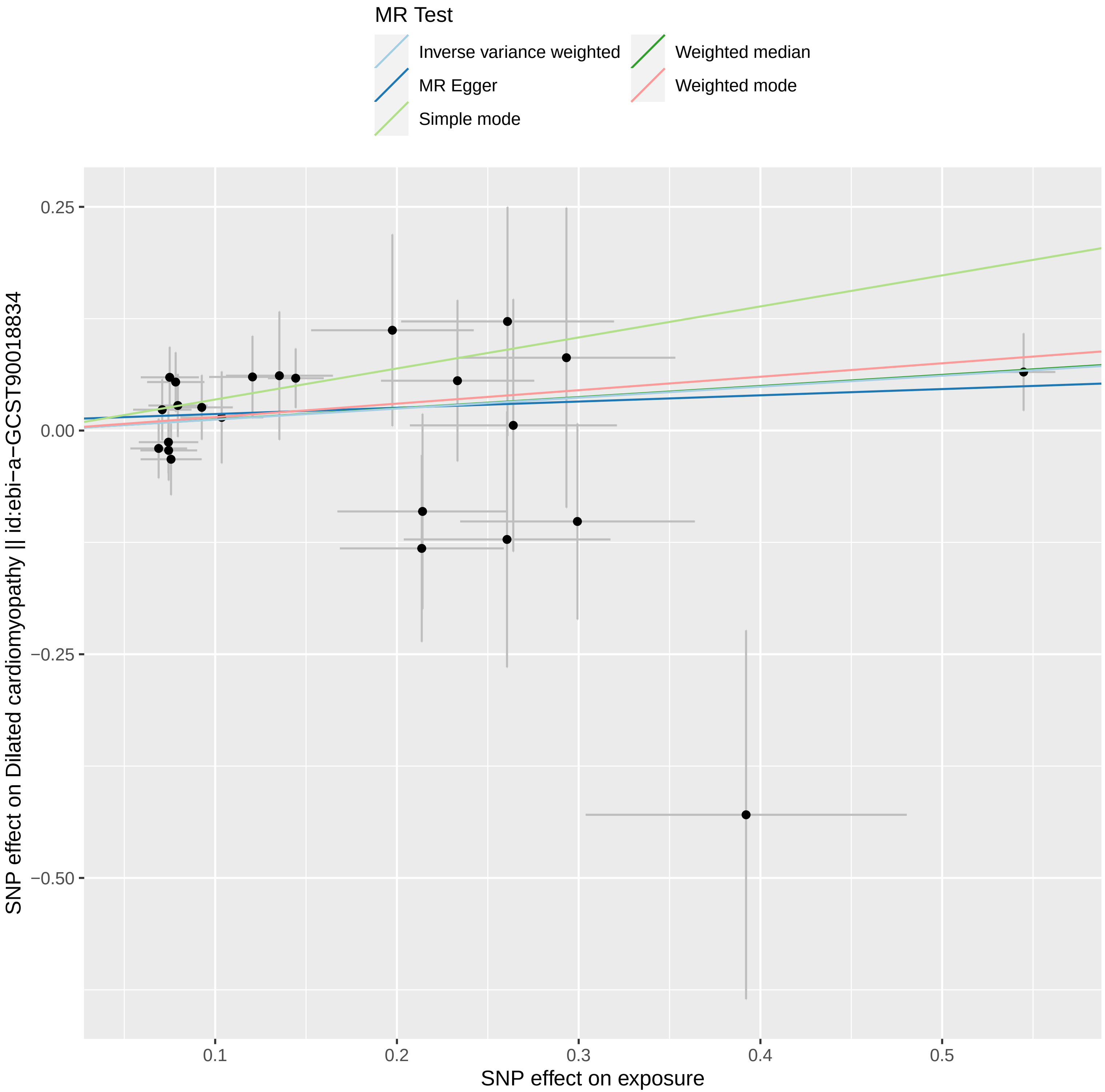
MR Test
Inverse variance weighted
MR Egger
Simple mode
Weighted median
Weighted mode
0.25
0.00
−0.25
−0.50
0.1
0.2
0.3
0.4
0.5
SNP effect on exposure
SNP effect on Dilated cardiomyopathy || id:ebi−a−GCST90018834

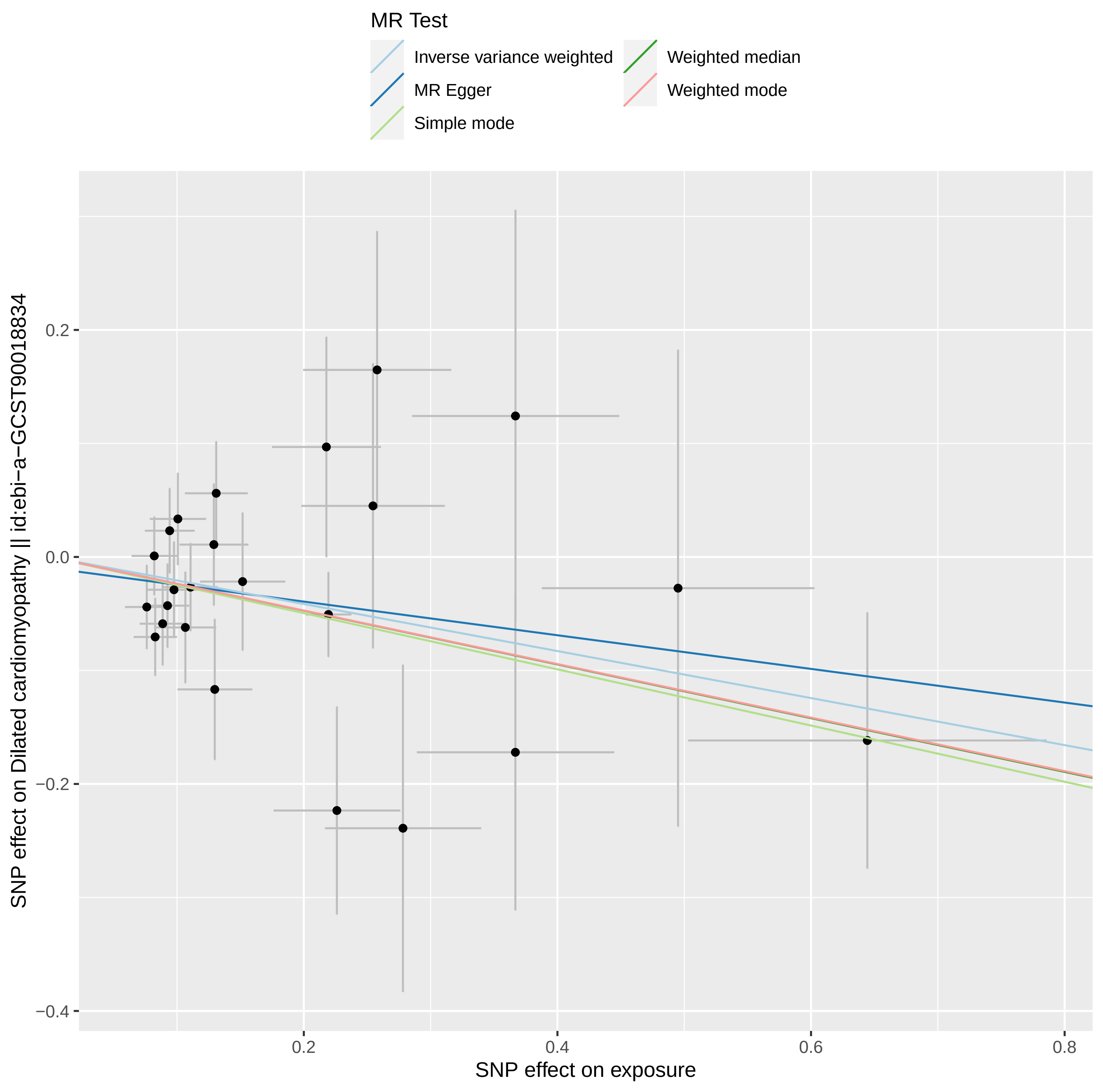
MR Test
Inverse variance weighted
MR Egger
Simple mode
Weighted median
Weighted mode
SNP effect on Dilated cardiomyopathy || id:ebi−a−GCST90018834
0.2
0.0
−0.2
−0.4
0.2
0.4
0.6
0.8
SNP effect on exposure

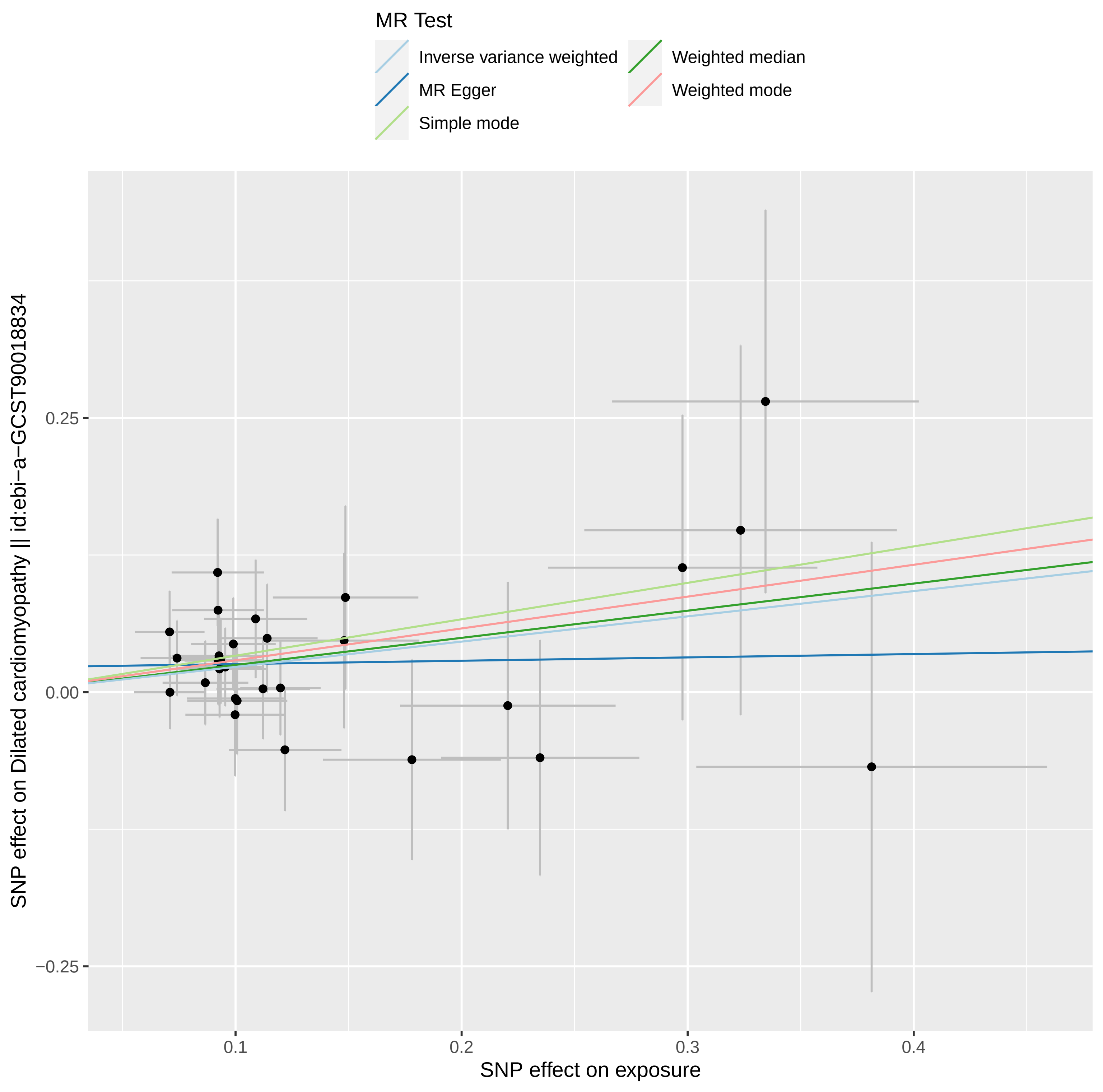
MR Test
Inverse variance weighted
MR Egger
Simple mode
Weighted median
Weighted mode
SNP effect on Dilated cardiomyopathy || id:ebi−a−GCST90018834
0.25
0.00
−0.25
0.1
0.2
0.3
0.4
SNP effect on exposure

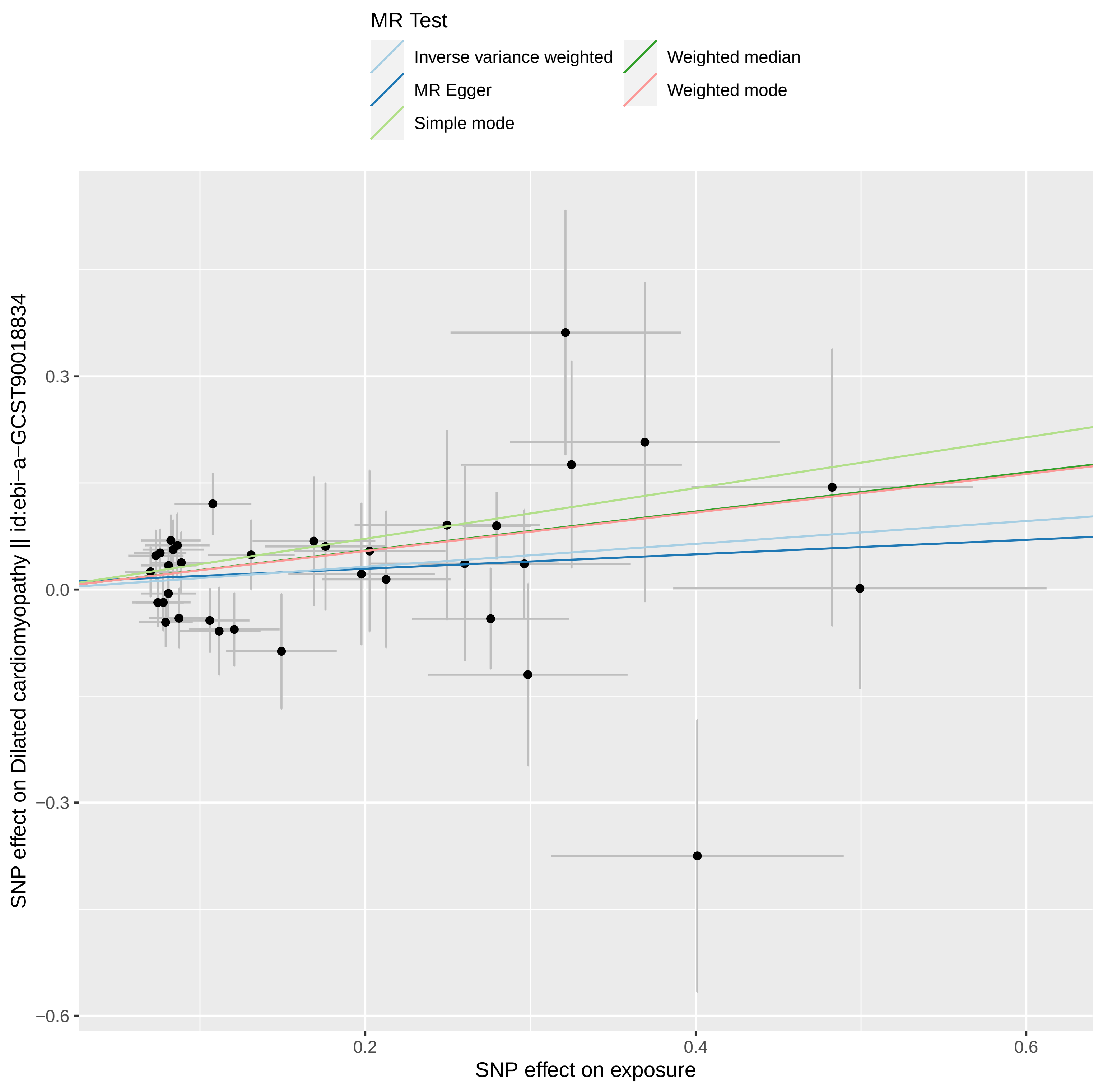
MR Test
Inverse variance weighted
MR Egger
Simple mode
Weighted median
Weighted mode
0.3
0.0
−0.3
−0.6
0.2
0.4
0.6
SNP effect on exposure
SNP effect on Dilated cardiomyopathy || id:ebi−a−GCST90018834

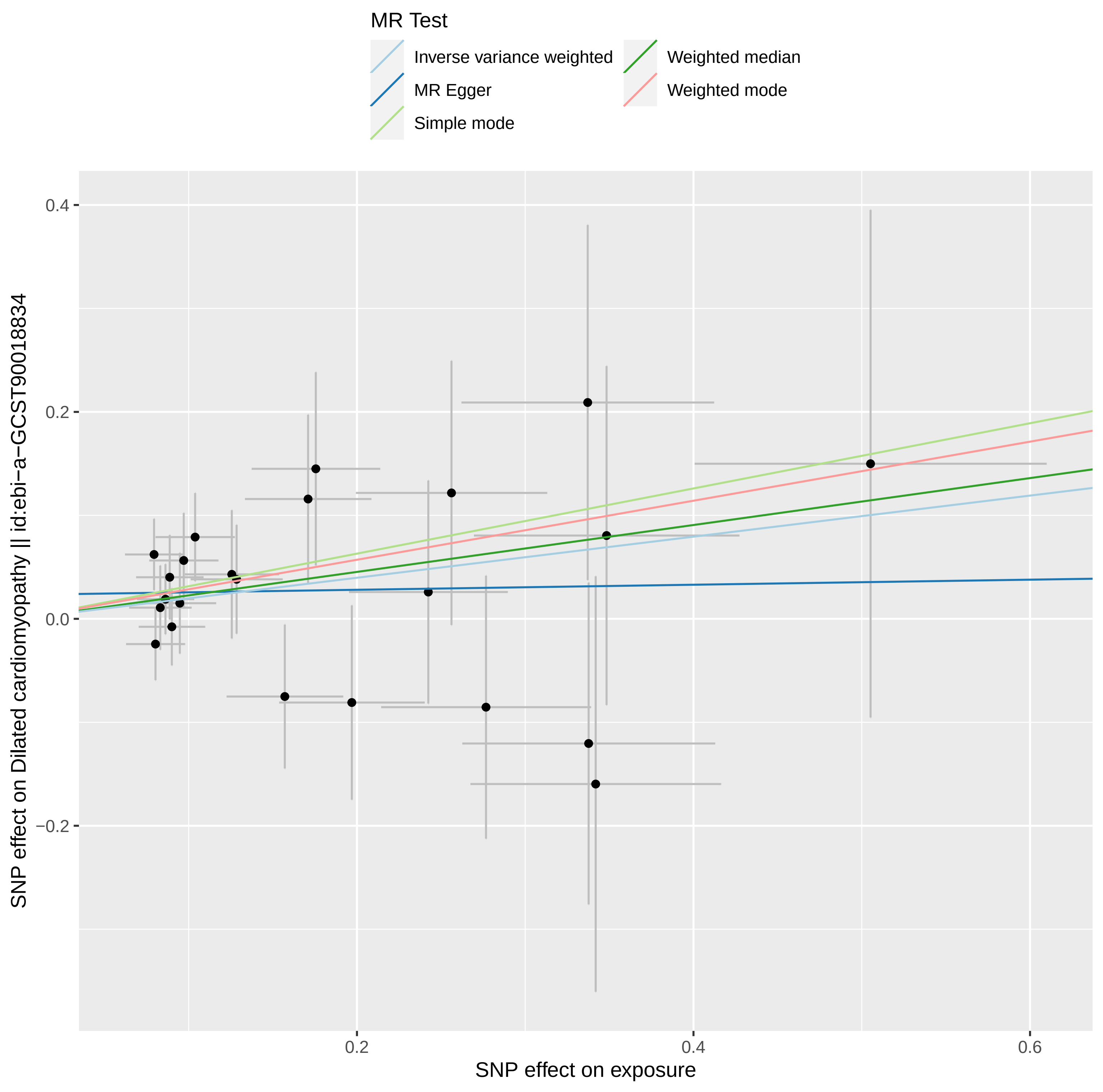

MR Test
Inverse variance weighted
MR Egger
Simple mode
Weighted median
Weighted mode
0.4
0.2
0.0
−0.2
0.2
0.4
0.6
SNP effect on Dilated cardiomyopathy || id:ebi−a−GCST90018834
SNP effect on exposure

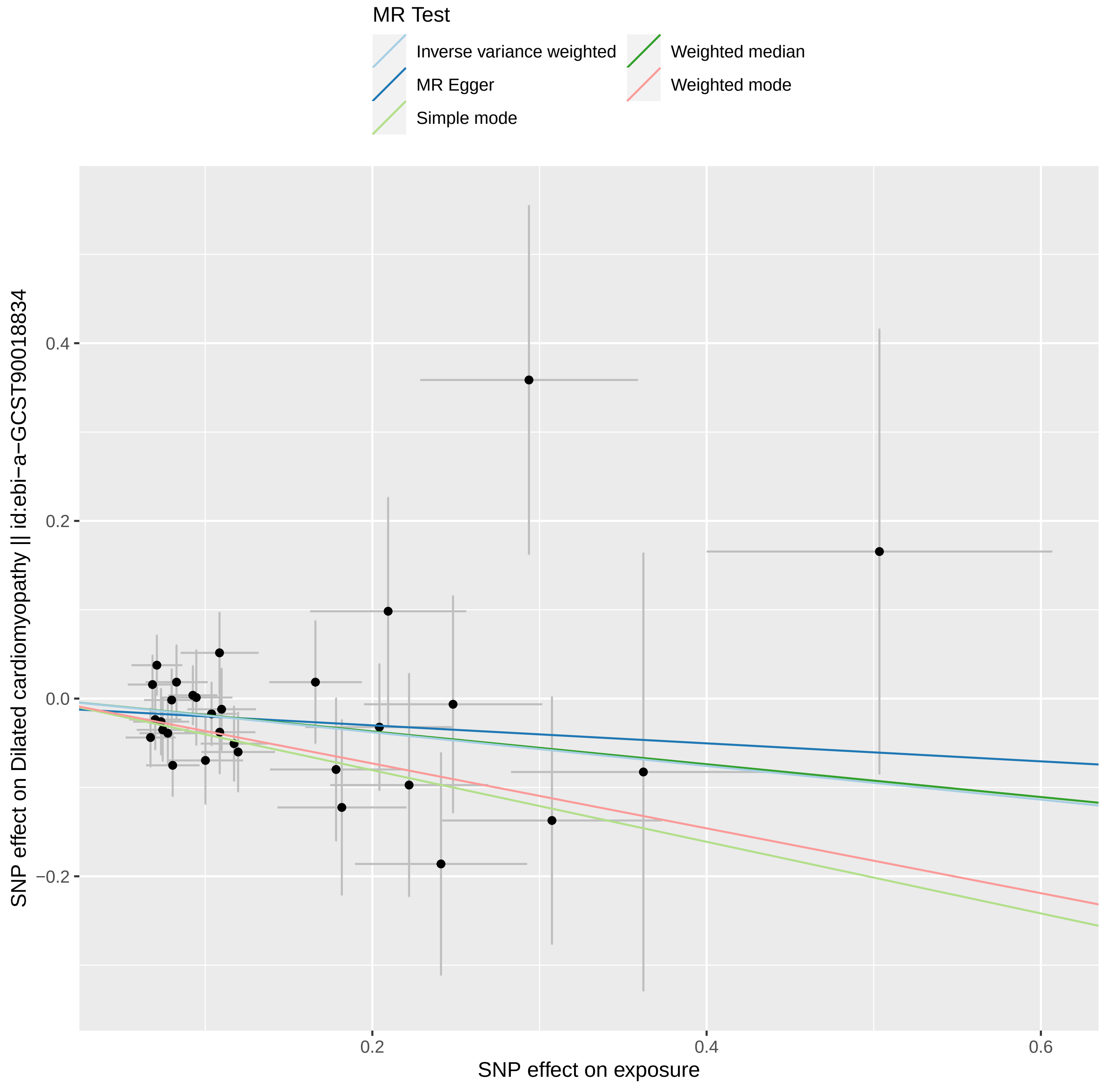

MR Test
Inverse variance weighted
MR Egger
Simple mode
Weighted median
Weighted mode
SNP effect on Dilated cardiomyopathy || id:ebi−a−GCST90018834
0.4
0.2
0.0
−0.2
SNP effect on exposure
0.2
0.4
0.6

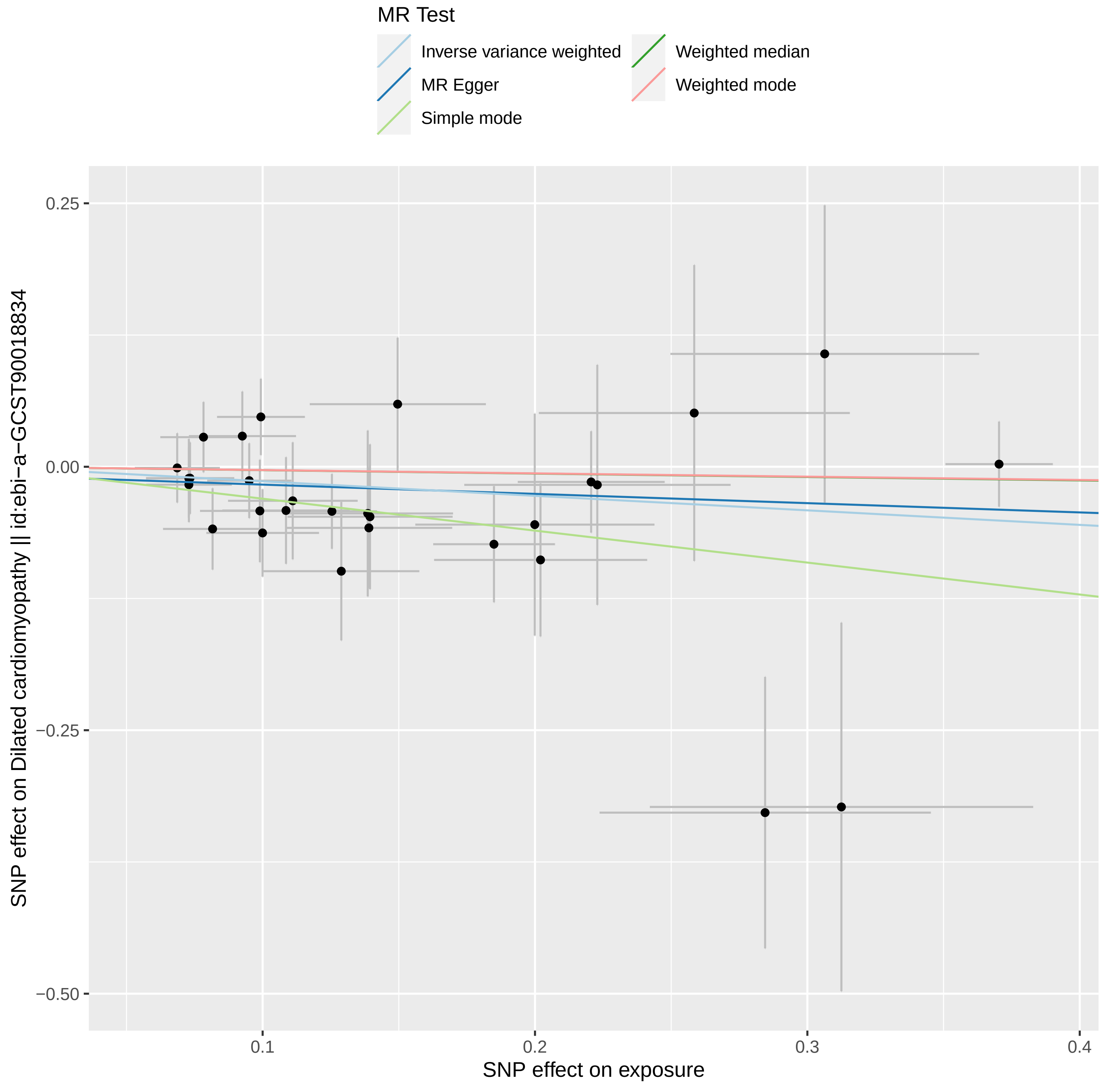

MR Test
Inverse variance weighted
MR Egger
Simple mode
Weighted median
Weighted mode
0.25
0.00
−0.25
−0.50
SNP effect on Dilated cardiomyopathy || id:ebi−a−GCST90018834
0.1
0.2
0.3
0.4
SNP effect on exposure

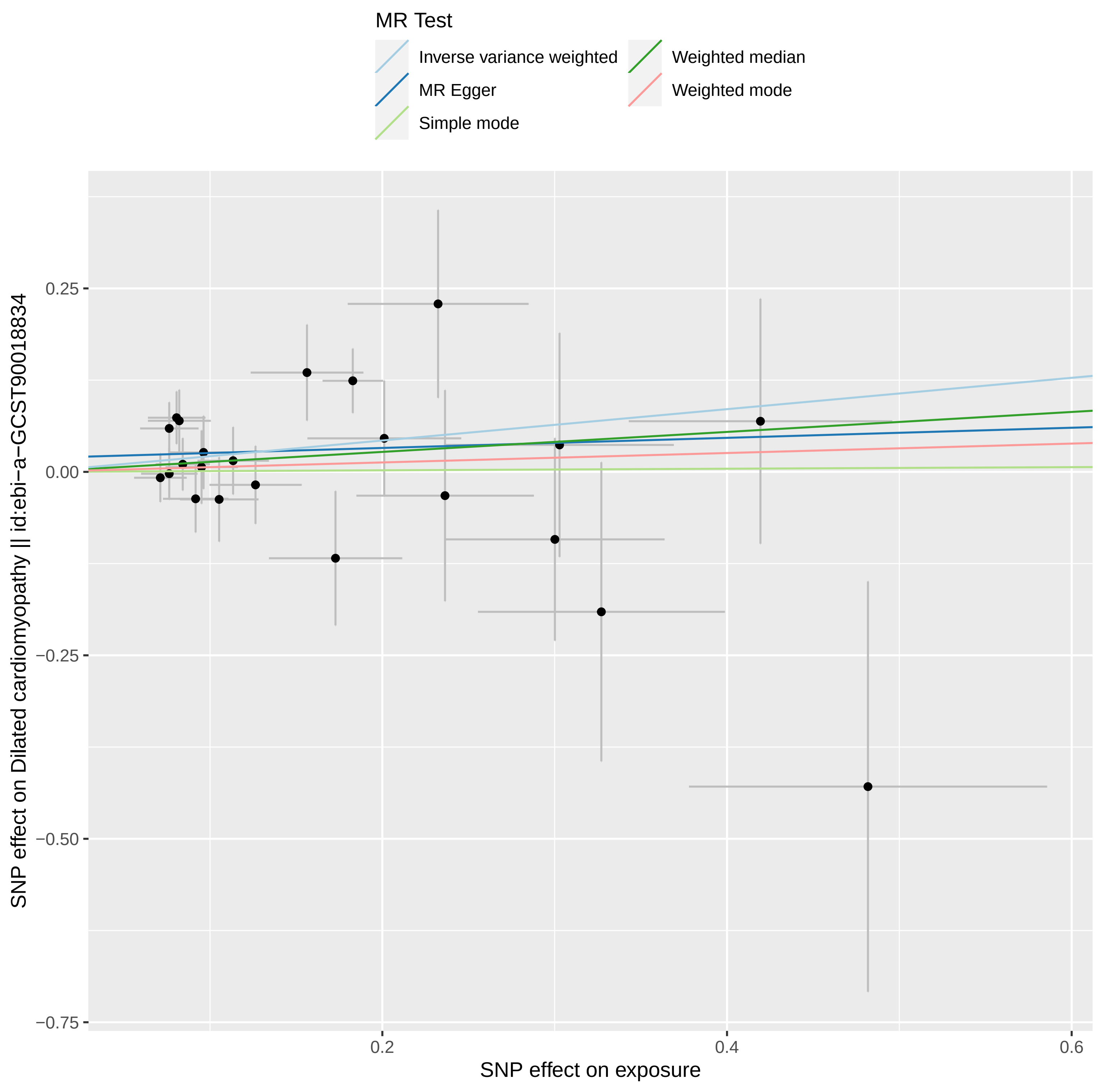

MR Test
Inverse variance weighted
MR Egger
Simple mode
Weighted median
Weighted mode
0.25
0.00
−0.25
−0.50
−0.75
0.2
0.4
0.6
SNP effect on Dilated cardiomyopathy || id:ebi−a−GCST90018834
SNP effect on exposure

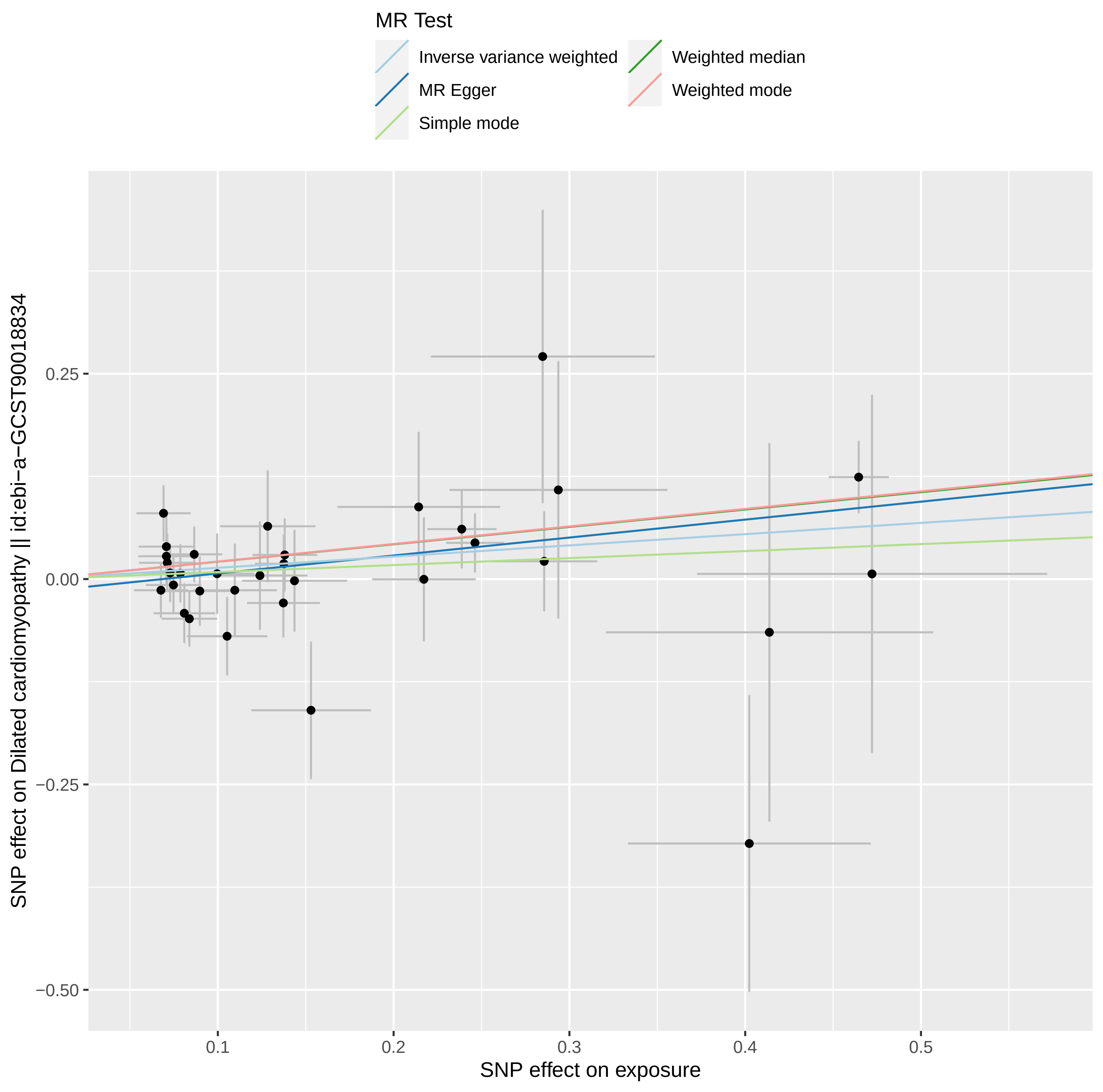
MR Test
Inverse variance weighted
MR Egger
Simple mode
Weighted median
Weighted mode
SNP effect on Dilated cardiomyopathy || id:ebi−a−GCST90018834
0.25
0.00
−0.25
−0.50
0.1
0.2
0.3
0.4
0.5
SNP effect on exposure

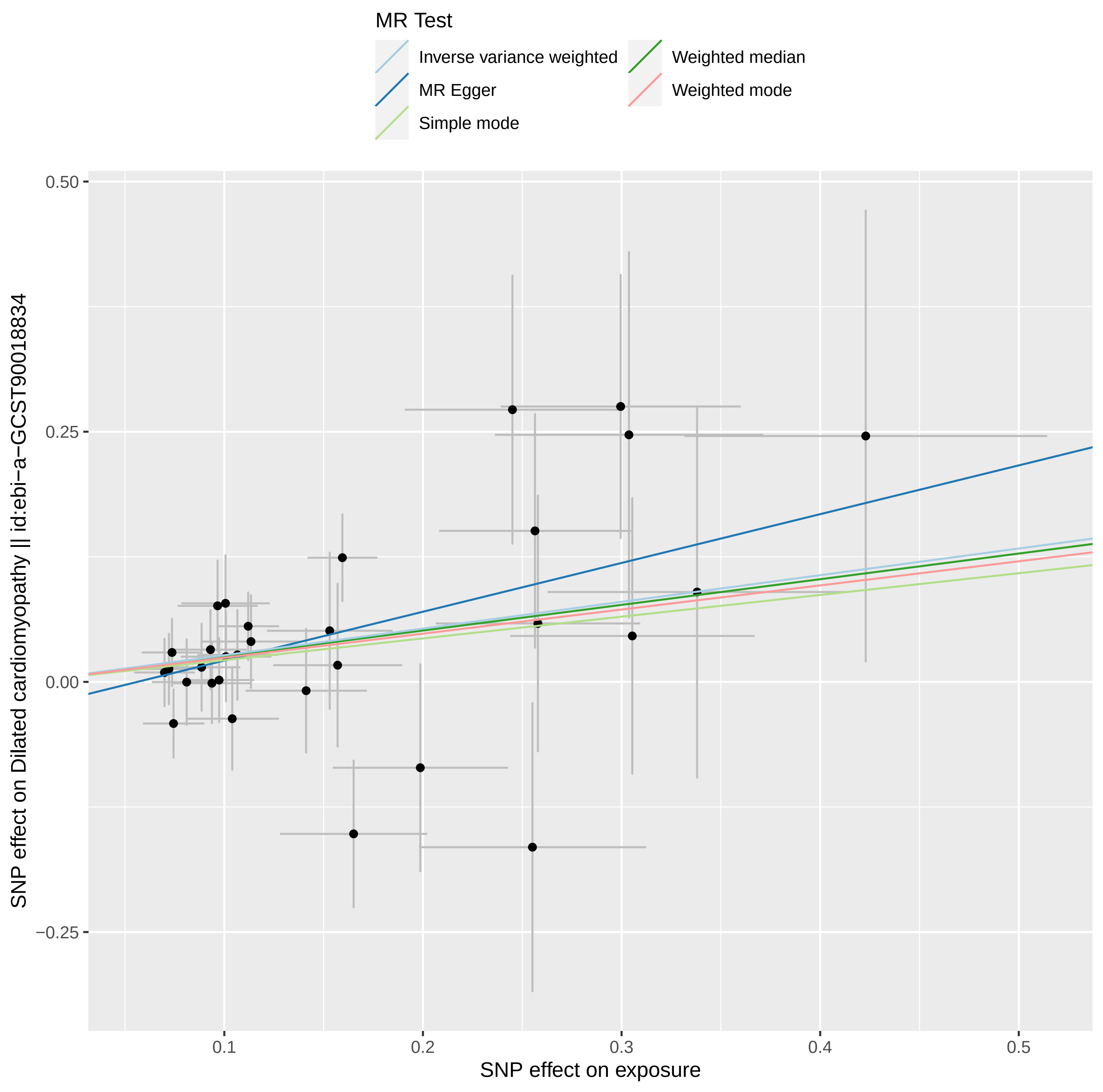
MR Test
Inverse variance weighted
MR Egger
Simple mode
Weighted median
Weighted mode
SNP effect on Dilated cardiomyopathy || id:ebi−a−GCST90018834
0.50
0.25
0.00
−0.25
0.1
0.2
0.3
0.4
0.5
SNP effect on exposure

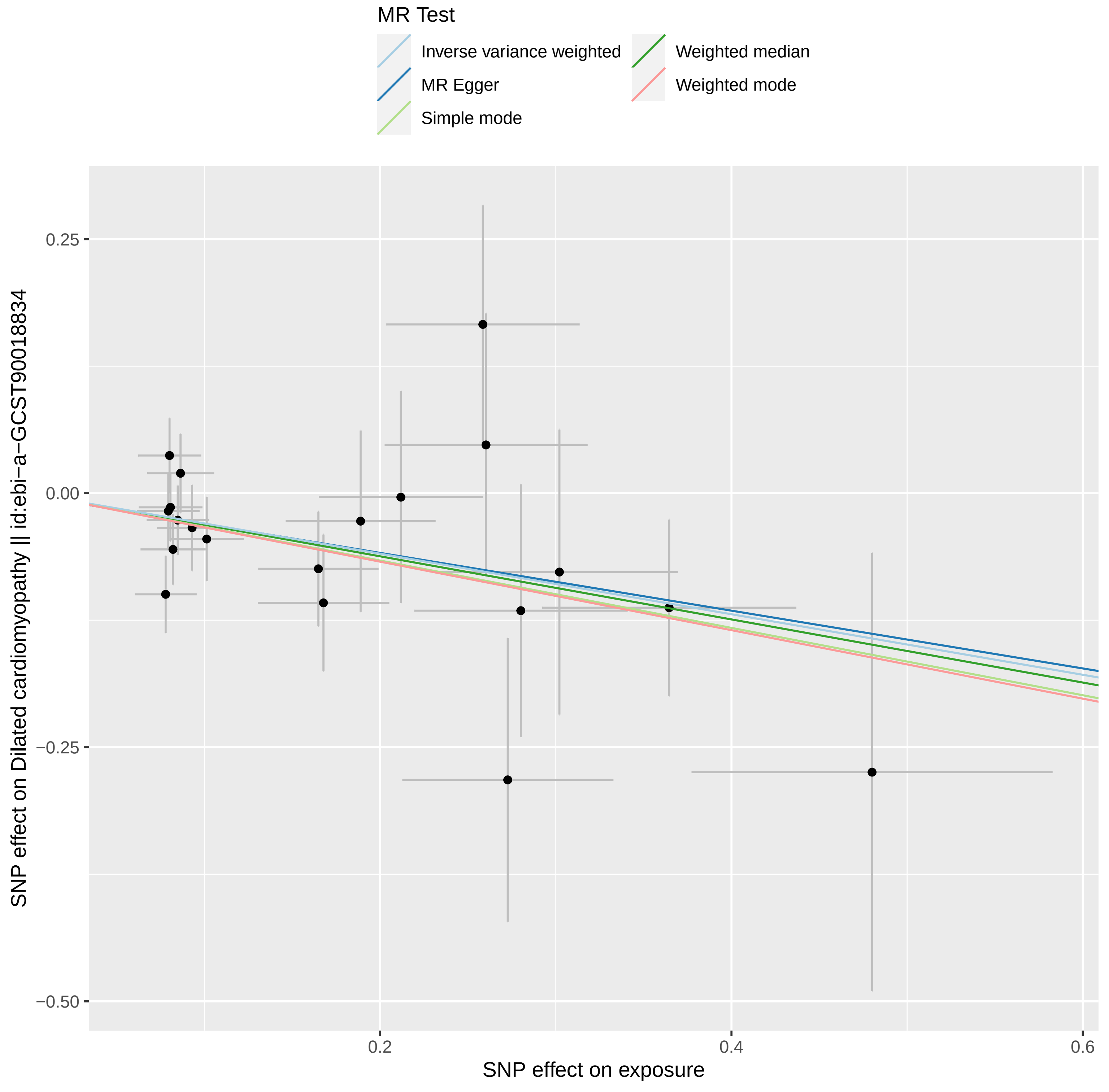

MR Test
Inverse variance weighted
MR Egger
Simple mode
Weighted median
Weighted mode
SNP effect on Dilated cardiomyopathy || id:ebi-a-GCST90018834
0.25
0.00
−0.25
−0.50
0.2
0.4
0.6
SNP effect on exposure

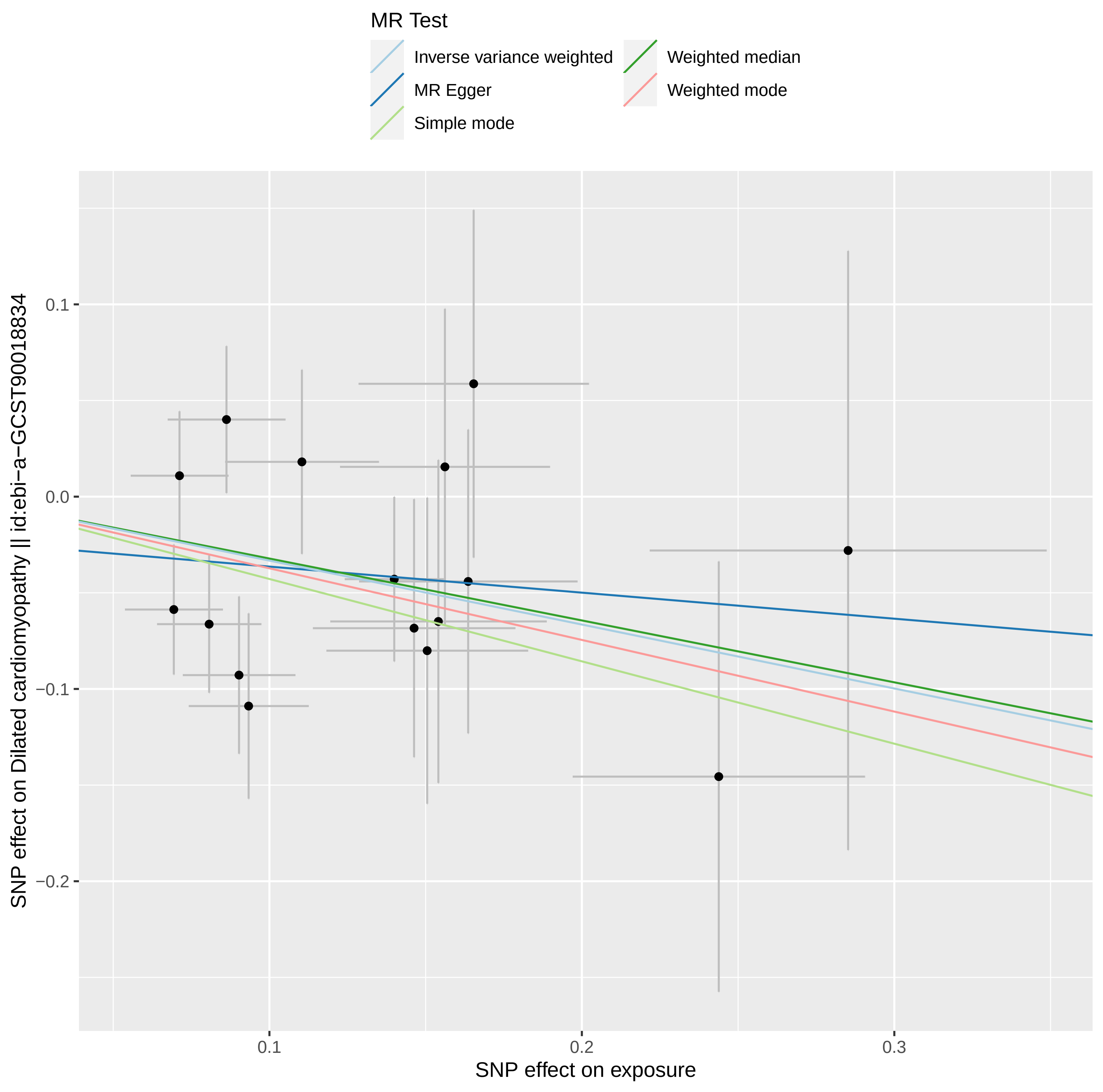
MR Test
Inverse variance weighted
MR Egger
Simple mode
Weighted median
Weighted mode
0.1
0.0
−0.1
−0.2
0.1
0.2
0.3
SNP effect on Dilated cardiomyopathy || id:ebi−a−GCST90018834
SNP effect on exposure

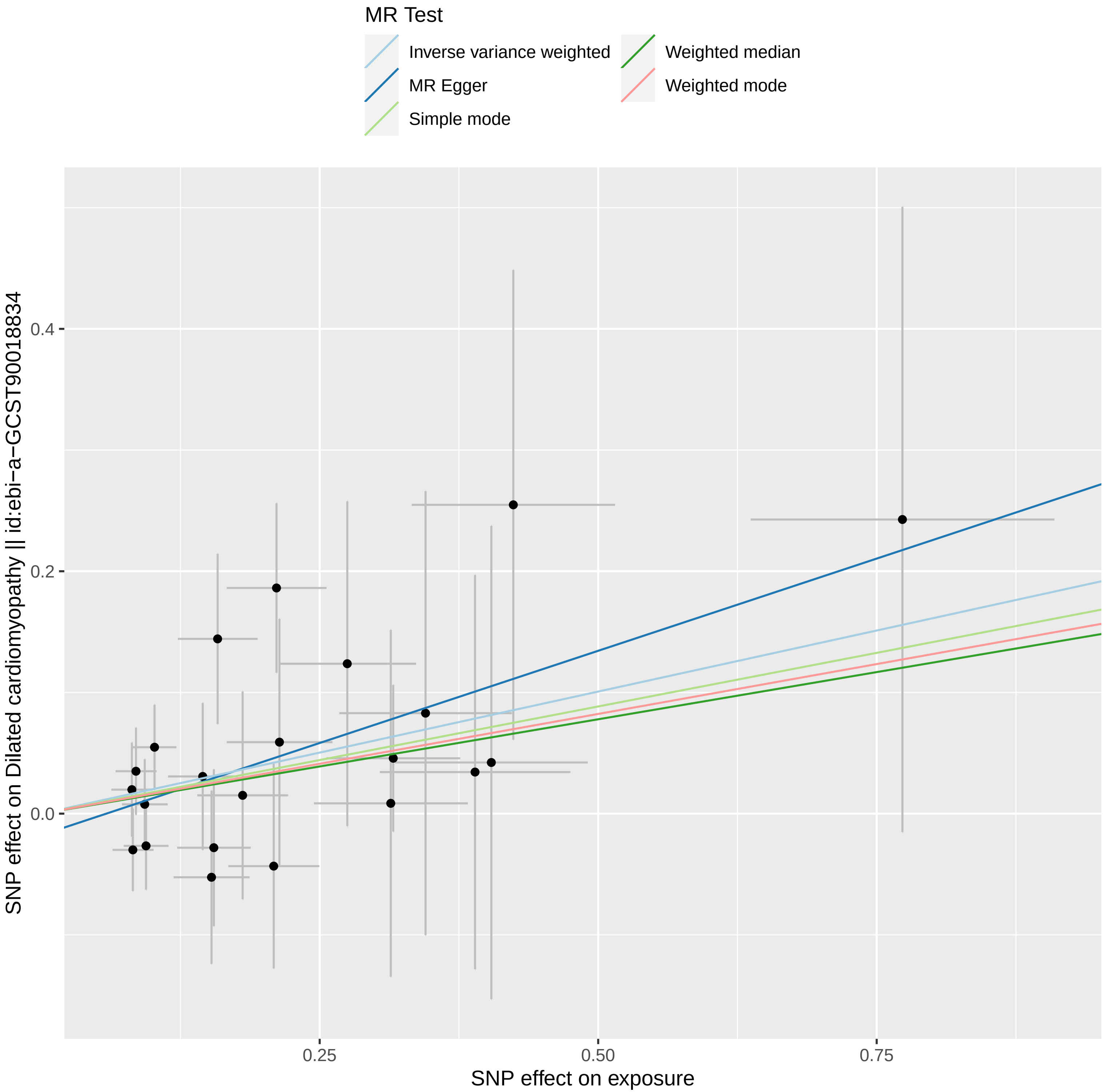
MR Test
Inverse variance weighted
MR Egger
Simple mode
Weighted median
Weighted mode
SNP effect on Dilated cardiomyopathy || id:ebi−a−GCST90018834
0.4
0.2
0.0
0.25
0.50
0.75
SNP effect on exposure

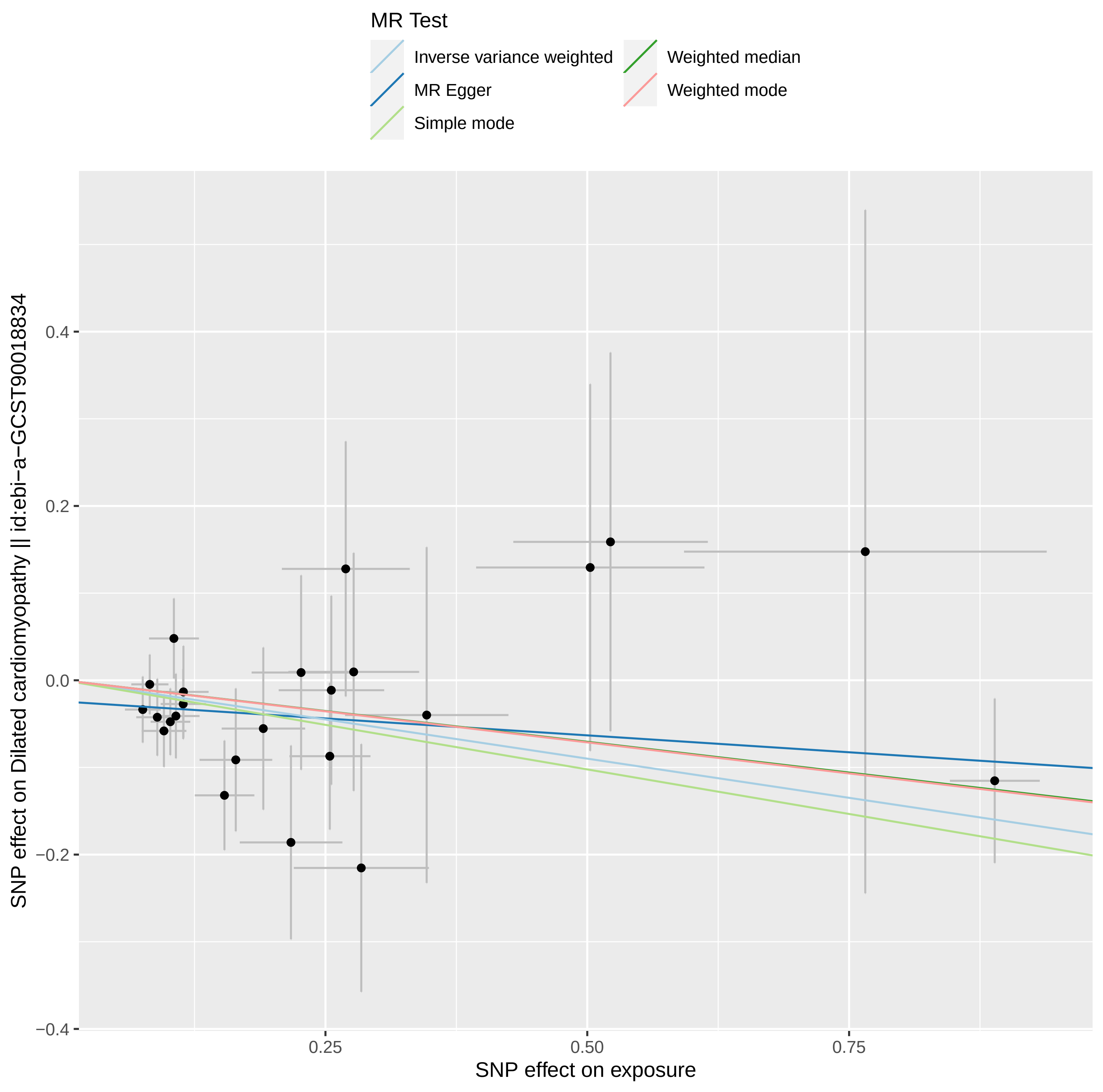
MR Test
Inverse variance weighted
MR Egger
Simple mode
Weighted median
Weighted mode
SNP effect on Dilated cardiomyopathy || id:ebi-a-GCST90018834
0.4
0.2
0.0
−0.2
−0.4
0.25
0.50
0.75
SNP effect on exposure

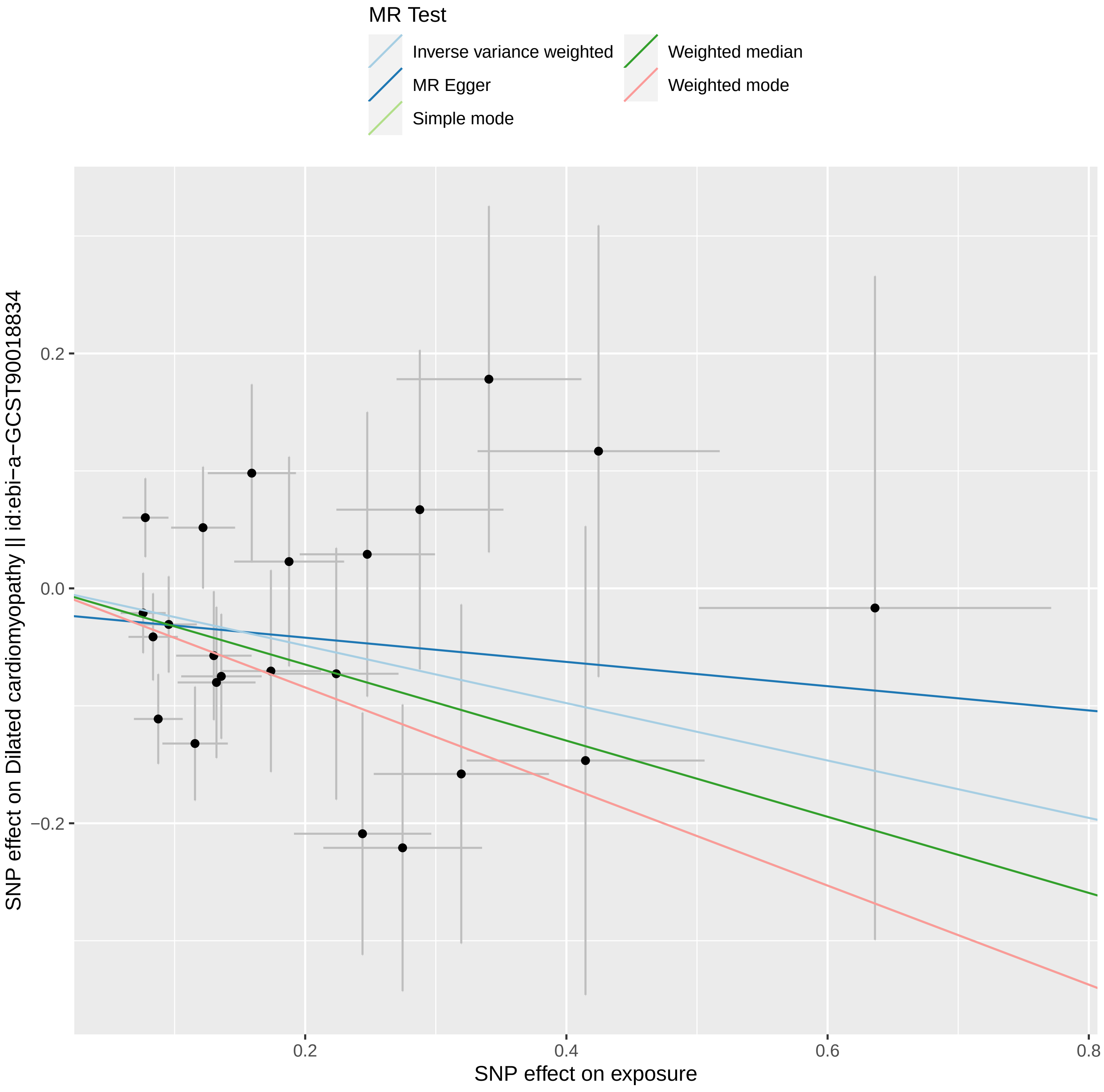

MR Test
Inverse variance weighted
MR Egger
Simple mode
Weighted median
Weighted mode
SNP effect on Dilated cardiomyopathy || id:ebi-a-GCST90018834
0.2
0.0
−0.2
0.2
0.4
0.6
0.8
SNP effect on exposure

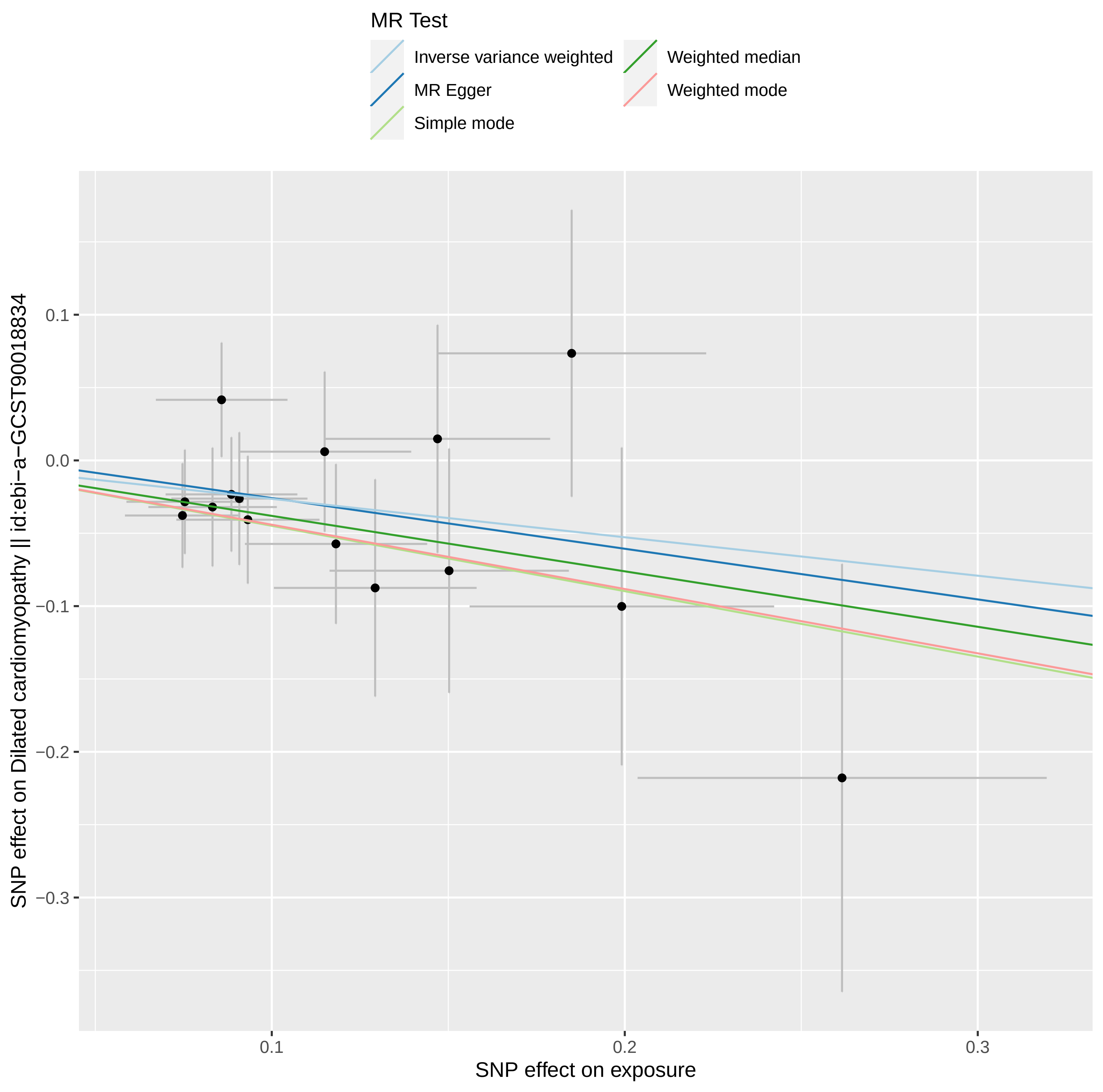
MR Test
Inverse variance weighted
MR Egger
Simple mode
Weighted median
Weighted mode
SNP effect on Dilated cardiomyopathy || id:ebi-a-GCST90018834
0.1
0.0
−0.1
−0.2
−0.3
0.1
0.2
0.3
SNP effect on exposure

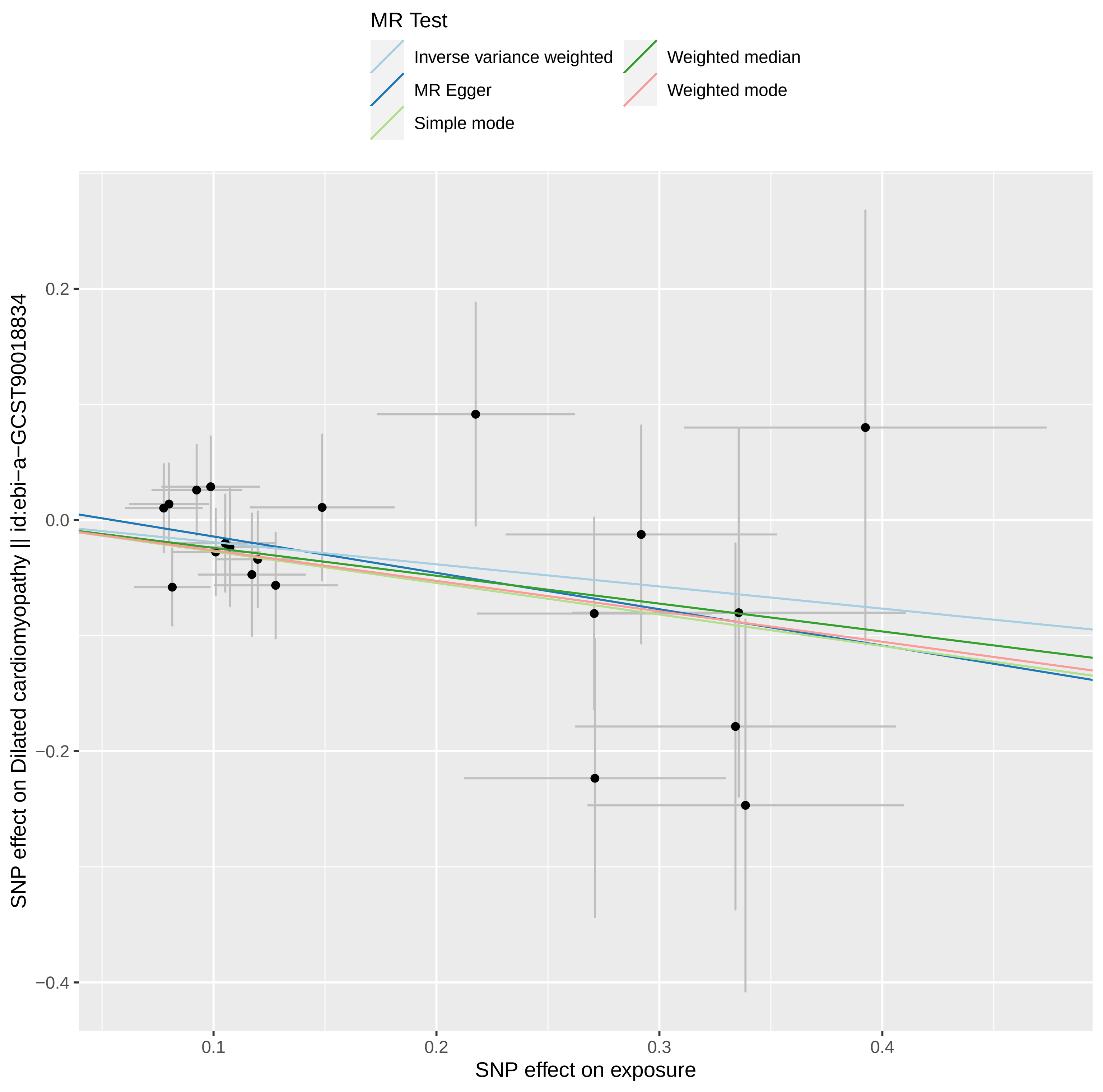

MR Test
Inverse variance weighted
MR Egger
Simple mode
Weighted median
Weighted mode
0.2
0.0
−0.2
−0.4
0.1
0.2
0.3
0.4
SNP effect on Dilated cardiomyopathy || id:ebi−a−GCST90018834
SNP effect on exposure

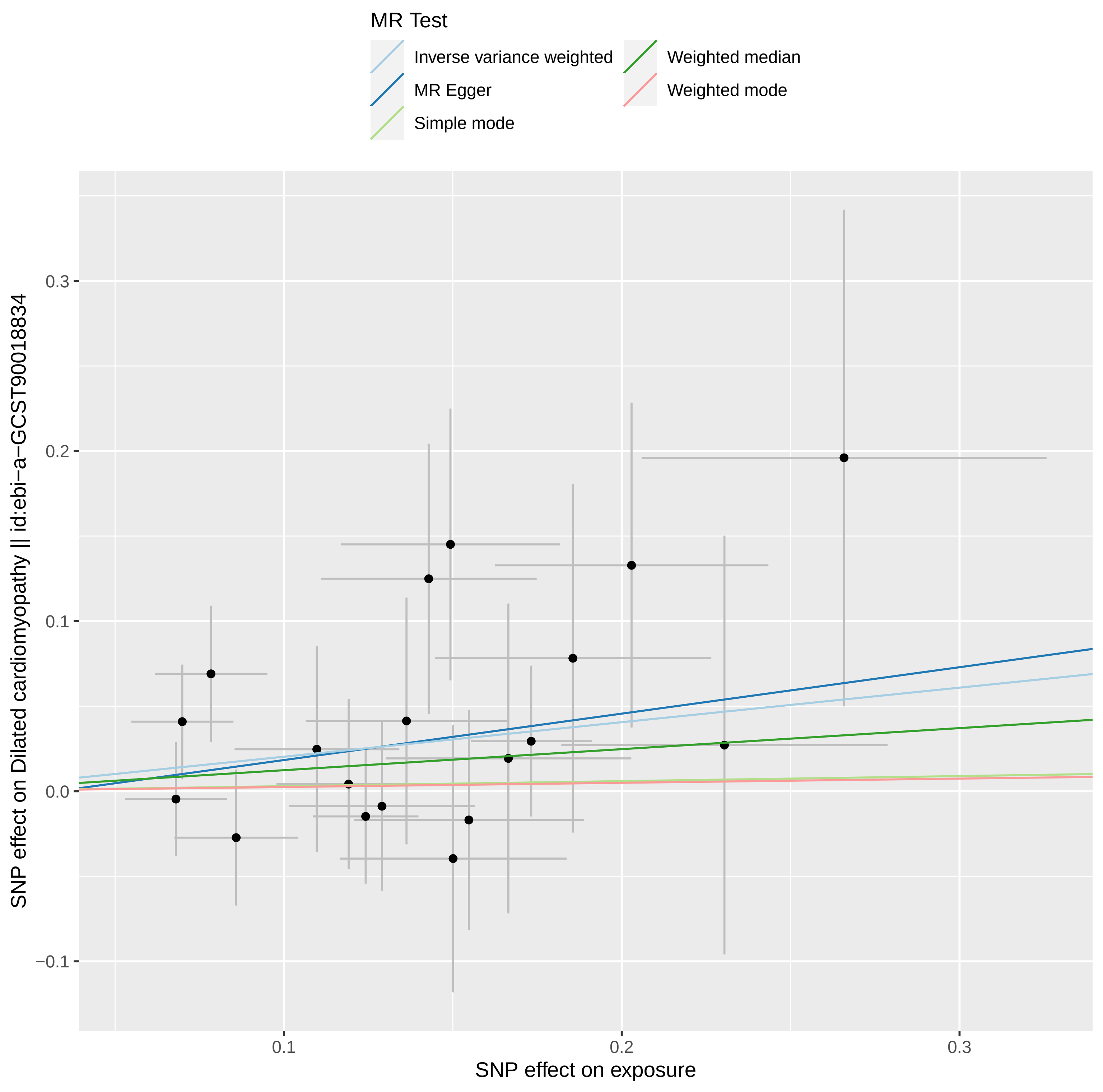
MR Test
Inverse variance weighted
MR Egger
Simple mode
Weighted median
Weighted mode
SNP effect on Dilated cardiomyopathy || id:ebi−a−GCST90018834
0.3
0.2
0.1
0.0
−0.1
0.1
0.2
0.3
SNP effect on exposure

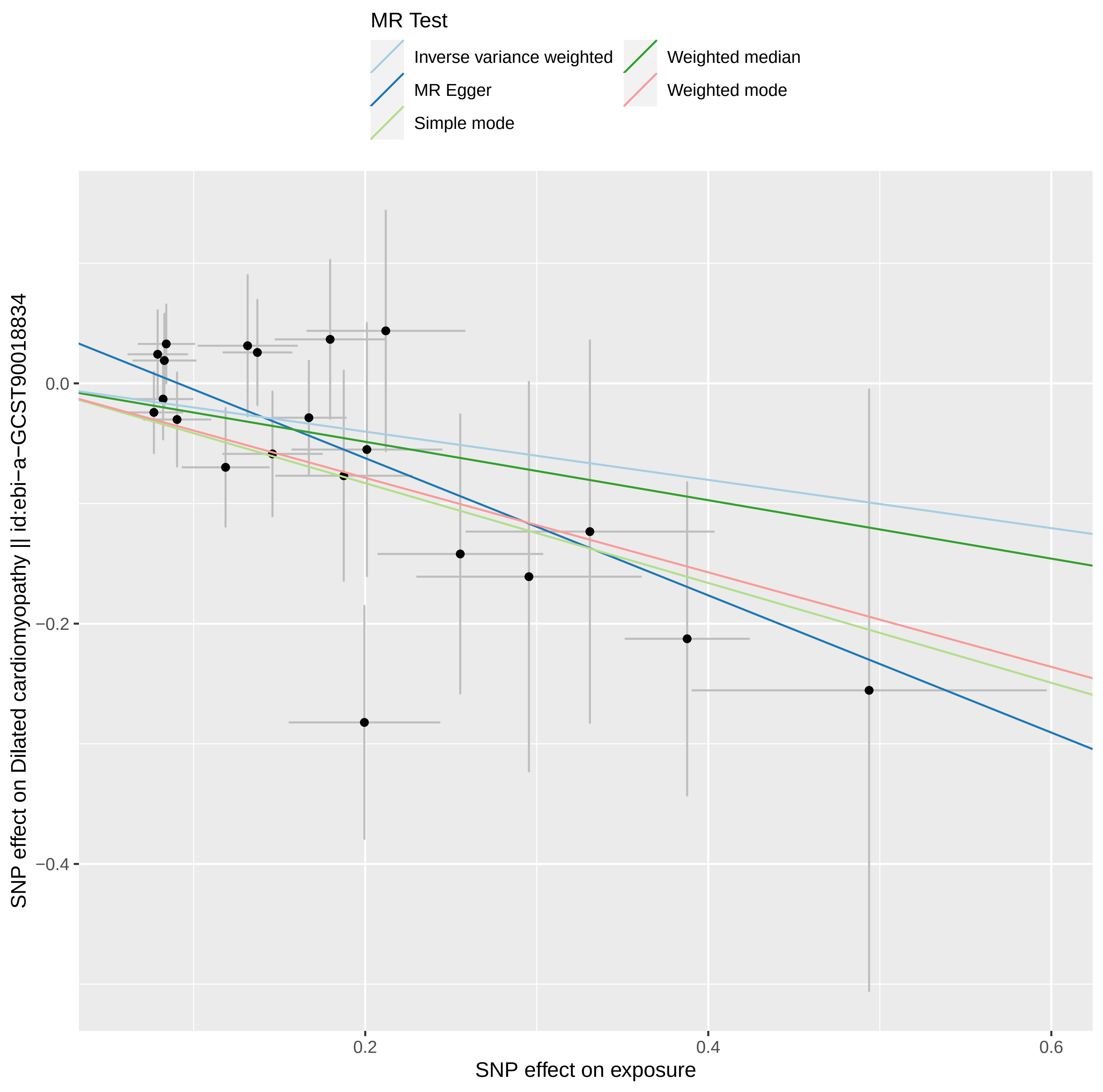

MR Test
Inverse variance weighted
MR Egger
Simple mode
Weighted median
Weighted mode
0.0
−0.2
−0.4
0.2
0.4
0.6
SNP effect on Dilated cardiomyopathy || id:ebi−a−GCST90018834
SNP effect on exposure

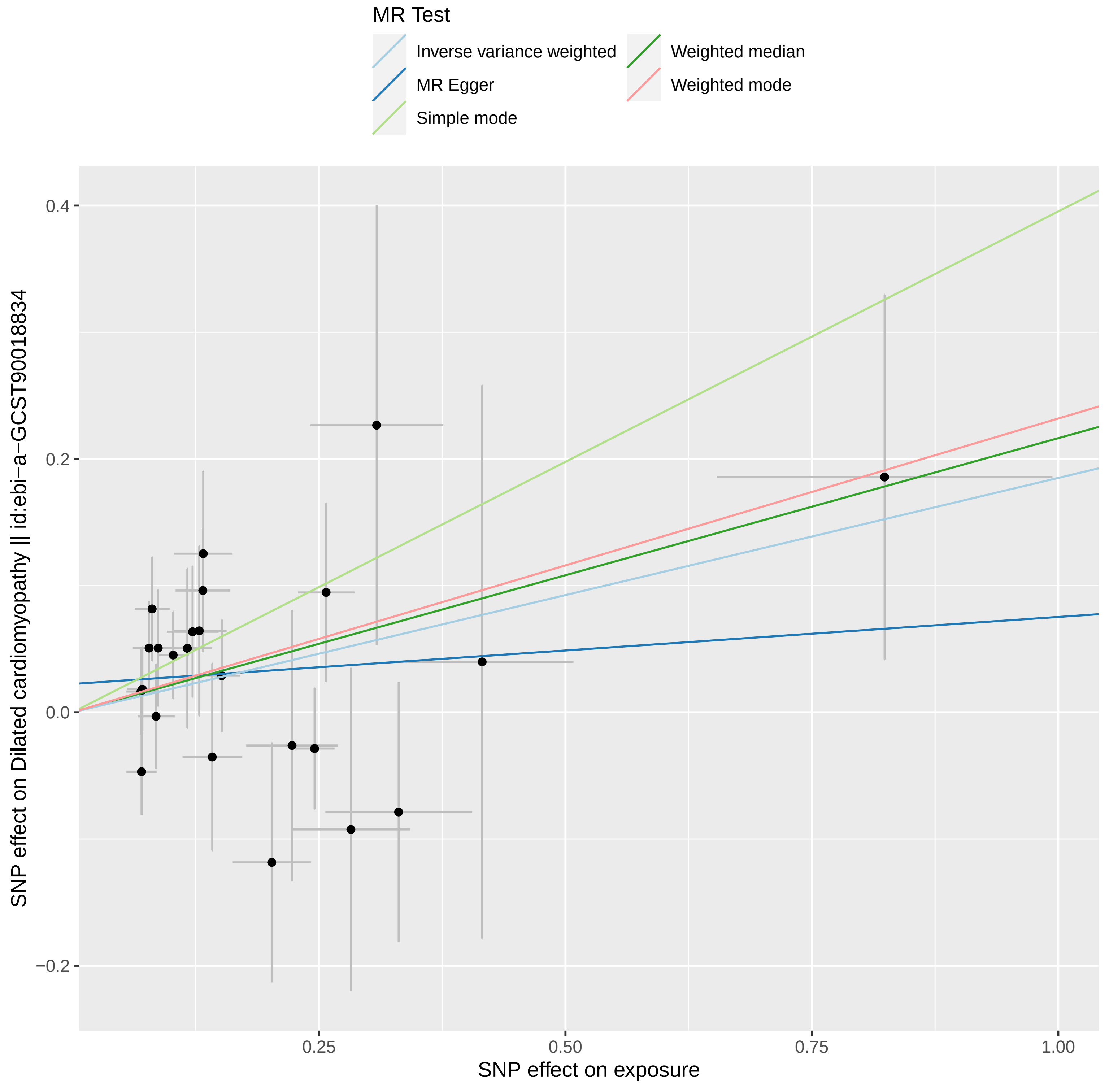

MR Test
Inverse variance weighted
MR Egger
Simple mode
Weighted median
Weighted mode
SNP effect on Dilated cardiomyopathy || id:ebi−a−GCST90018834
0.4
0.2
0.0
−0.2
0.25
0.50
0.75
1.00
SNP effect on exposure

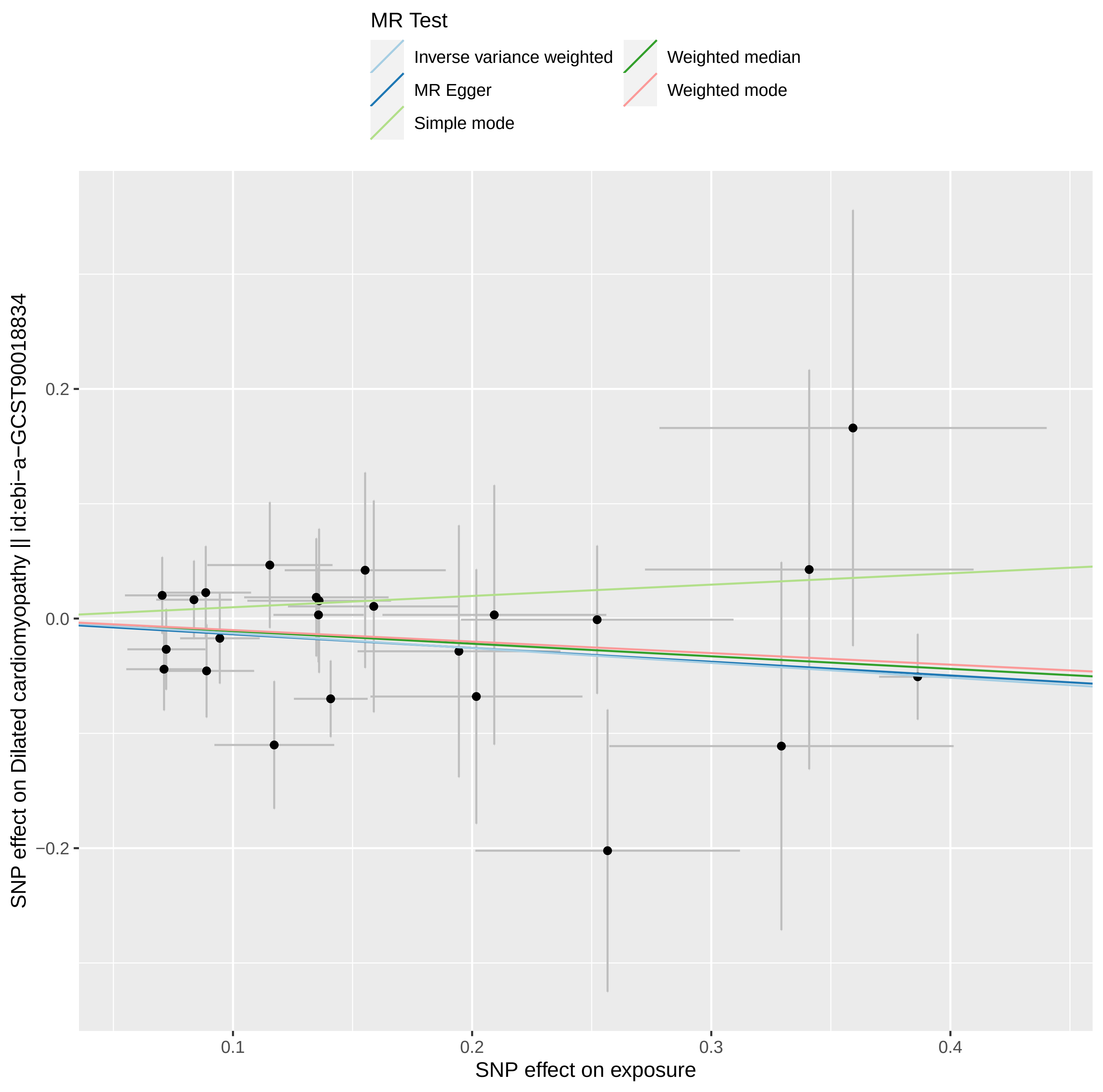

MR Test
Inverse variance weighted
MR Egger
Simple mode
Weighted median
Weighted mode
0.2
0.0
−0.2
0.1
0.2
0.3
0.4
SNP effect on Dilated cardiomyopathy || id:ebi−a−GCST90018834
SNP effect on exposure

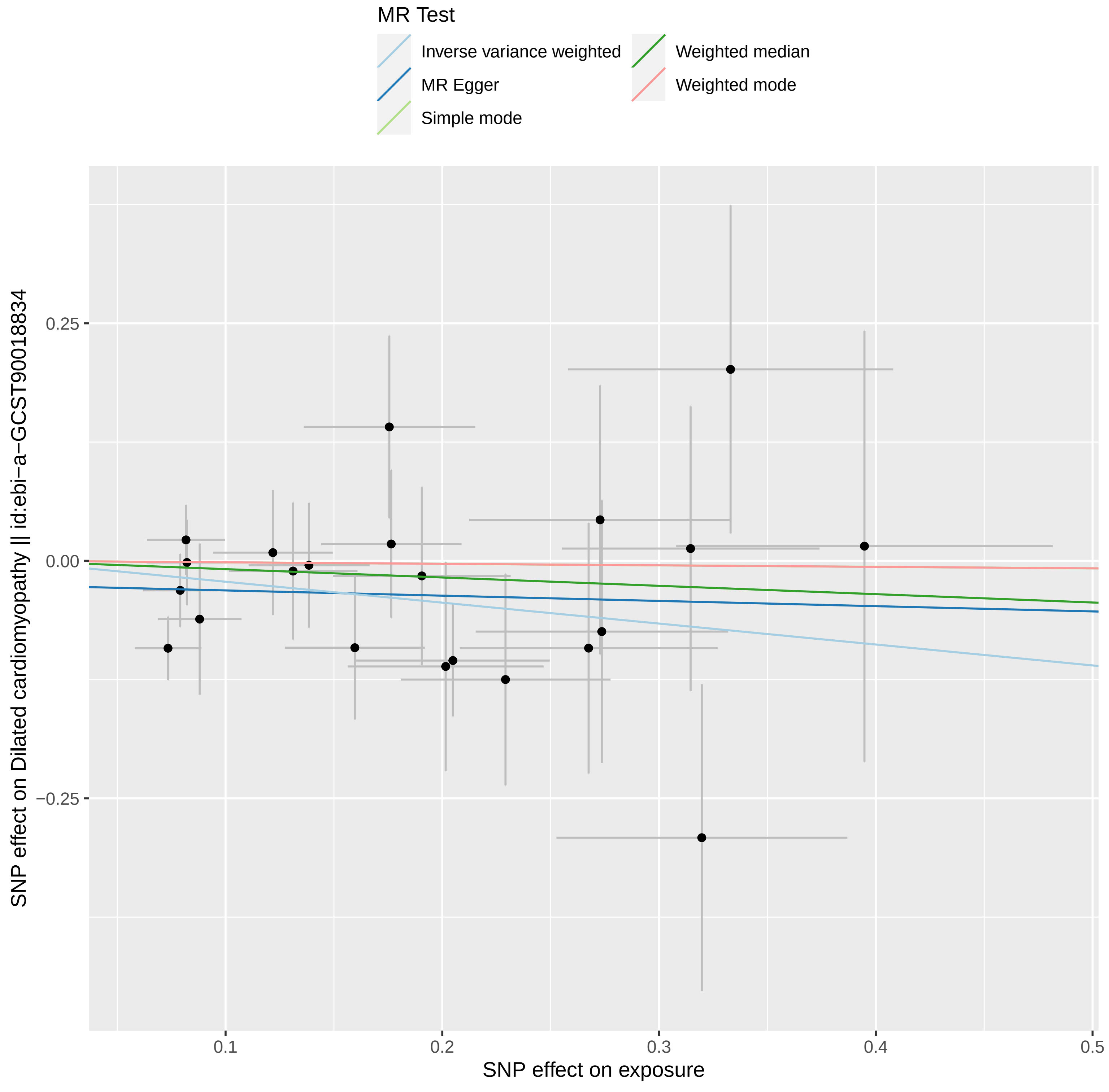
MR Test
Inverse variance weighted
MR Egger
Simple mode
Weighted median
Weighted mode
0.25
0.00
−0.25
0.1
0.2
0.3
0.4
0.5
SNP effect on exposure
SNP effect on Dilated cardiomyopathy || id:ebi−a−GCST90018834

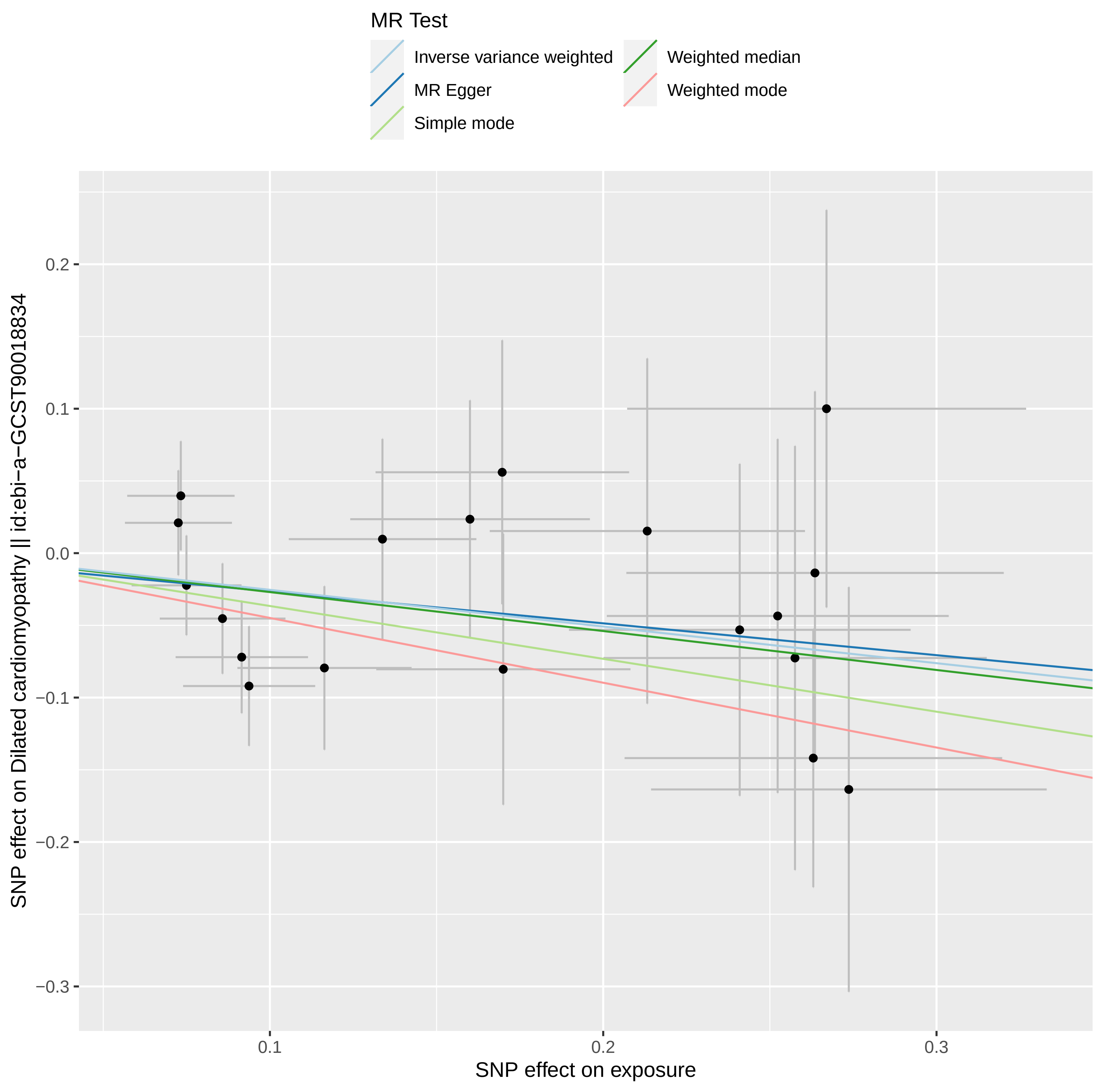
MR Test
Inverse variance weighted
MR Egger
Simple mode
Weighted median
Weighted mode
0.2
0.1
0.0
−0.1
−0.2
−0.3
SNP effect on Dilated cardiomyopathy || id:ebi−a−GCST90018834
0.1
0.2
0.3
SNP effect on exposure

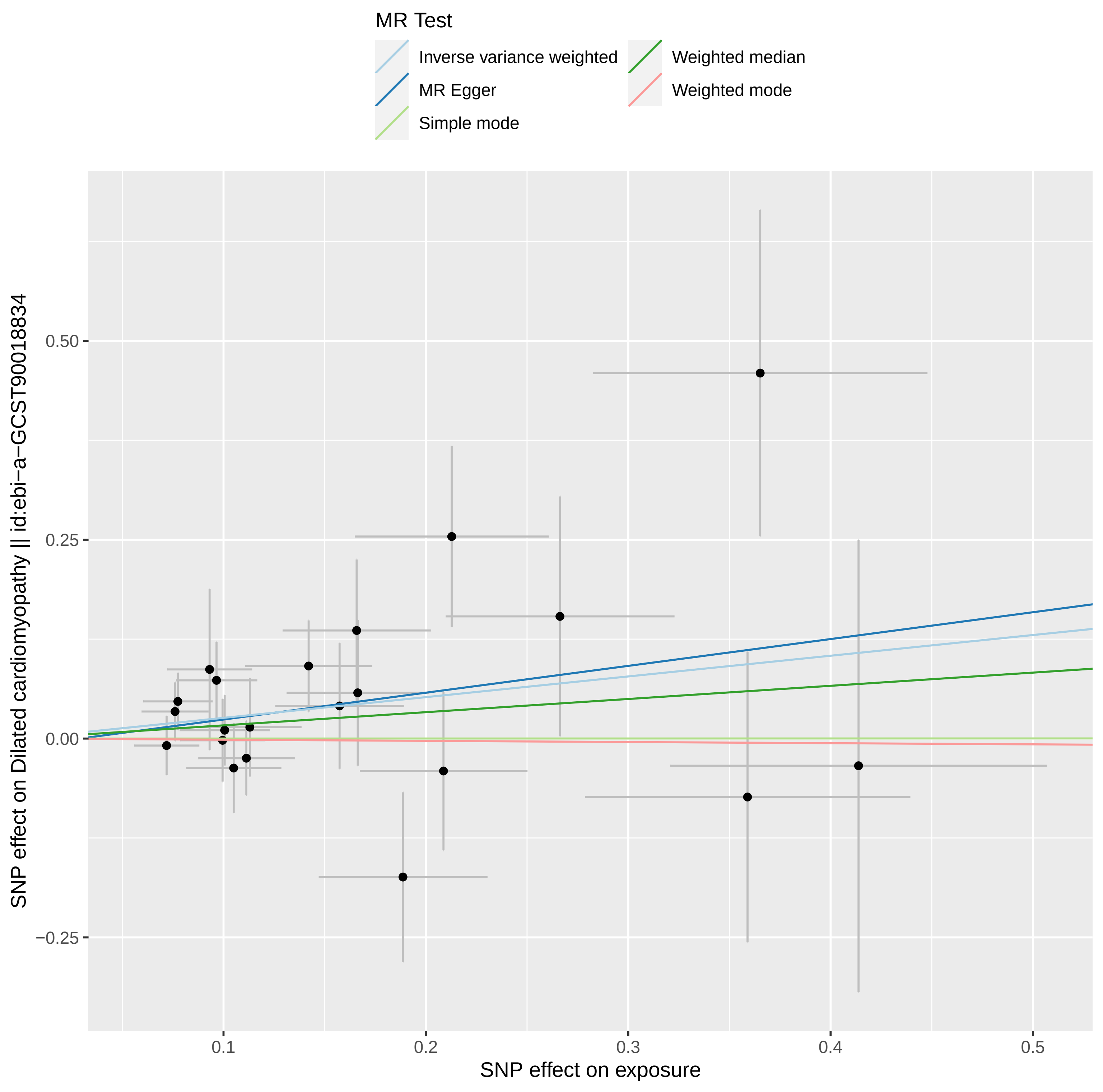
MR Test
Inverse variance weighted
MR Egger
Simple mode
Weighted median
Weighted mode
SNP effect on Dilated cardiomyopathy || id:ebi−a−GCST90018834
0.50
0.25
0.00
−0.25
0.1
0.2
0.3
0.4
0.5
SNP effect on exposure

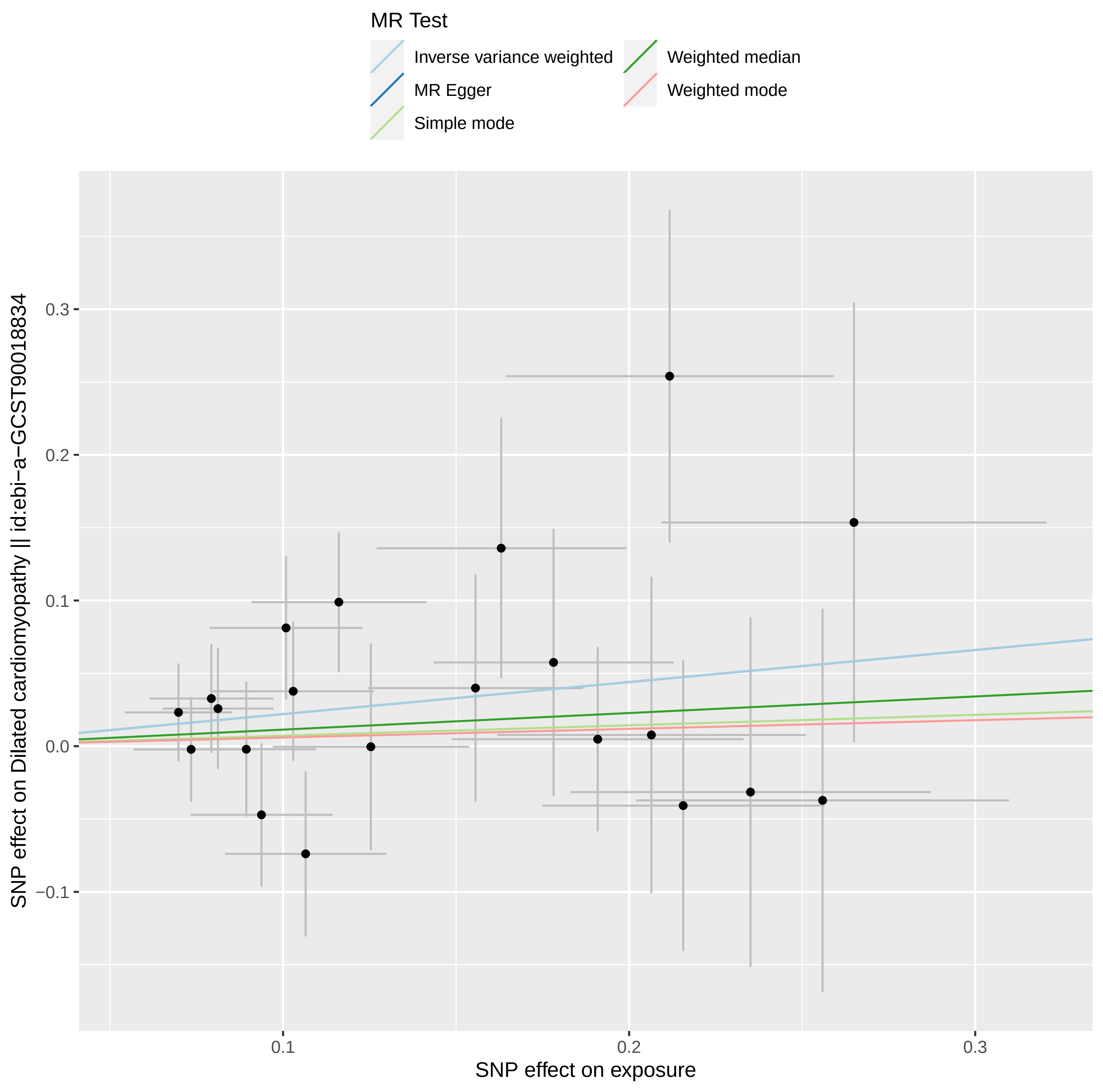
MR Test
Inverse variance weighted
MR Egger
Simple mode
Weighted median
Weighted mode
0.3
0.2
0.1
0.0
−0.1
SNP effect on Dilated cardiomyopathy || id:ebi−a−GCST90018834
0.1
0.2
0.3
SNP effect on exposure

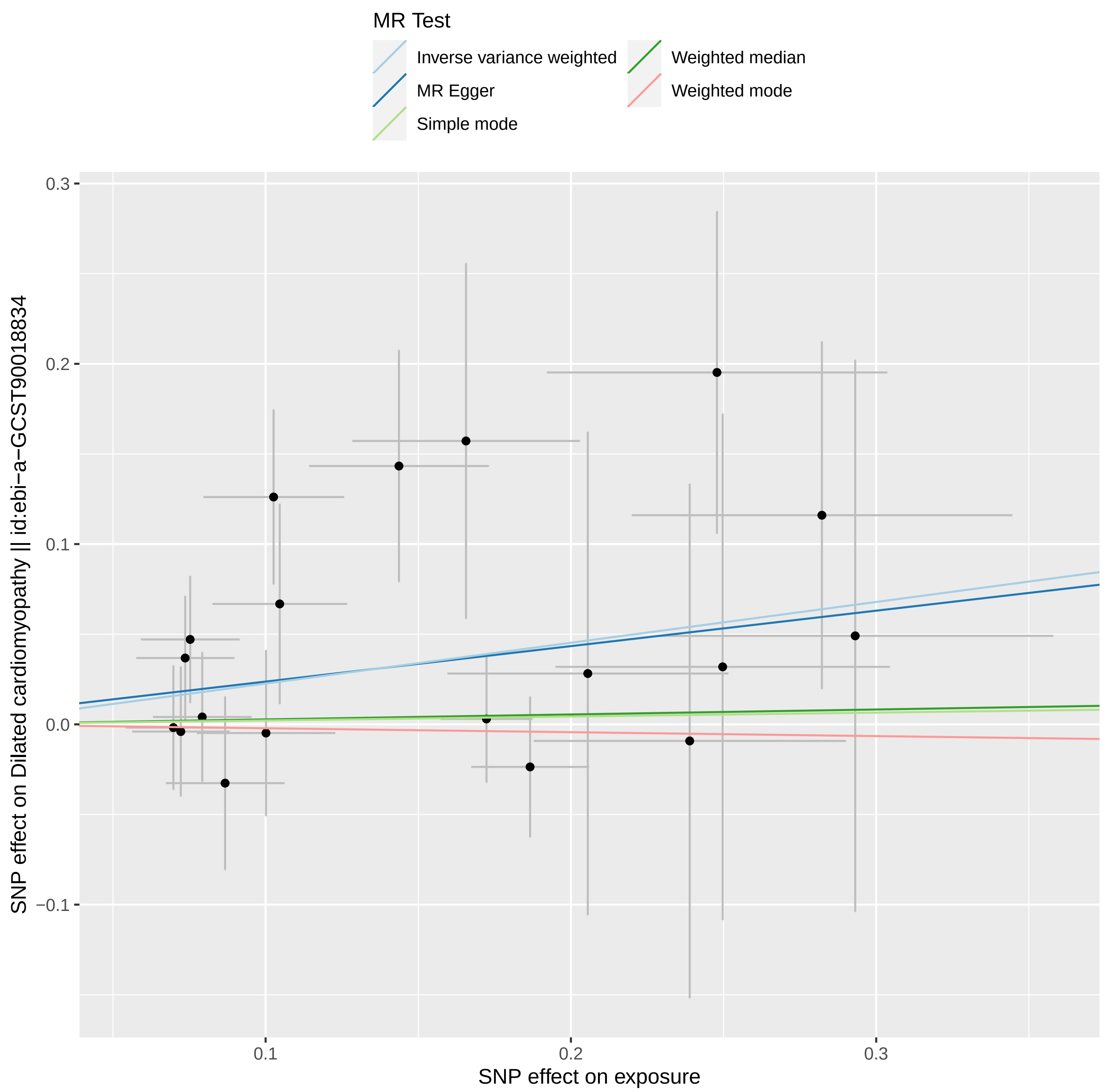

MR Test
Inverse variance weighted
MR Egger
Simple mode
Weighted median
Weighted mode
0.3
0.2
0.1
0.0
−0.1
0.1
0.2
0.3
SNP effect on Dilated cardiomyopathy || id:ebi−a−GCST90018834
SNP effect on exposure

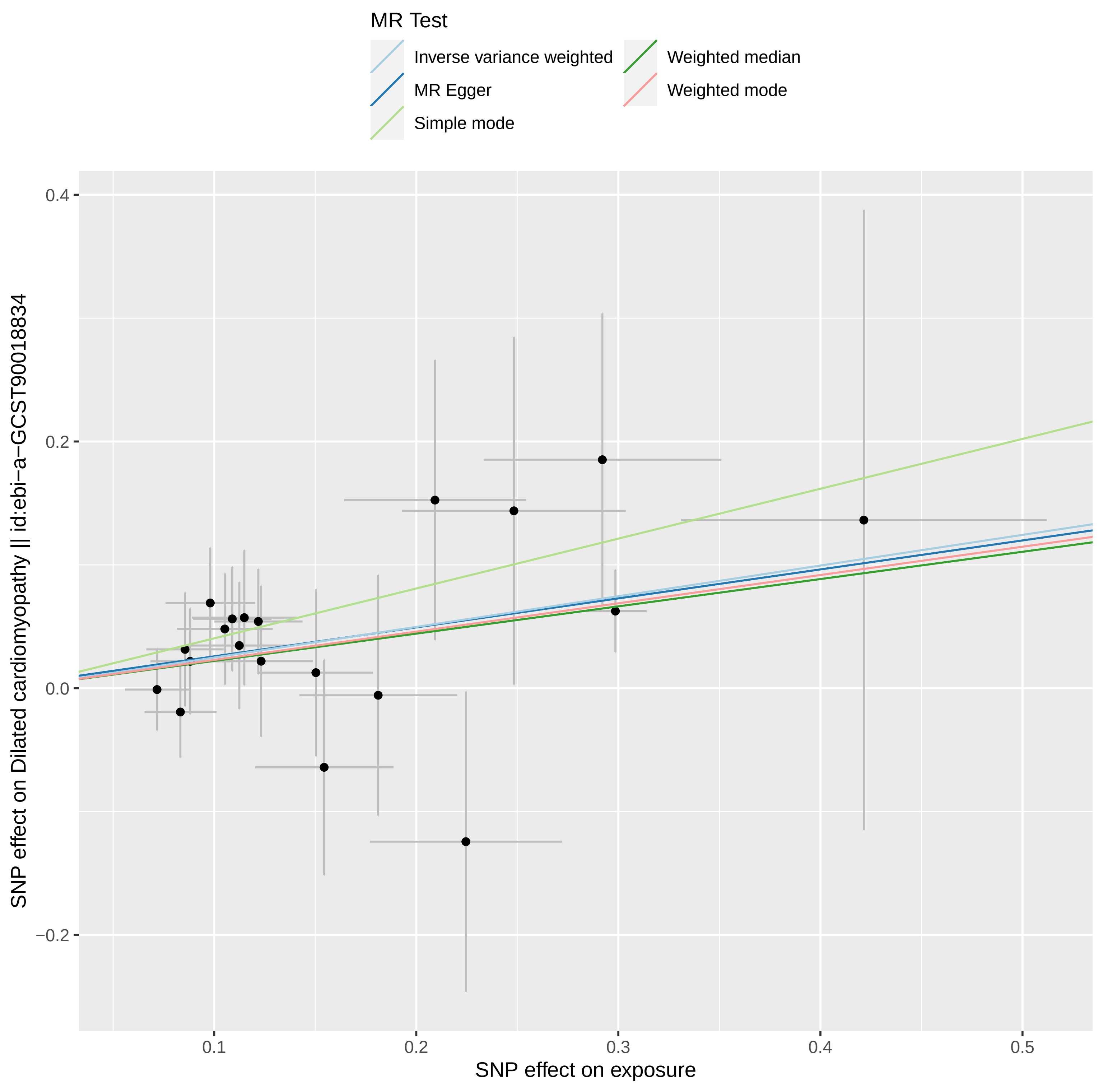
MR Test
Inverse variance weighted
MR Egger
Simple mode
Weighted median
Weighted mode
0.4
0.2
0.0
−0.2
0.1
0.2
0.3
0.4
0.5
SNP effect on Dilated cardiomyopathy || id:ebi−a−GCST90018834
SNP effect on exposure

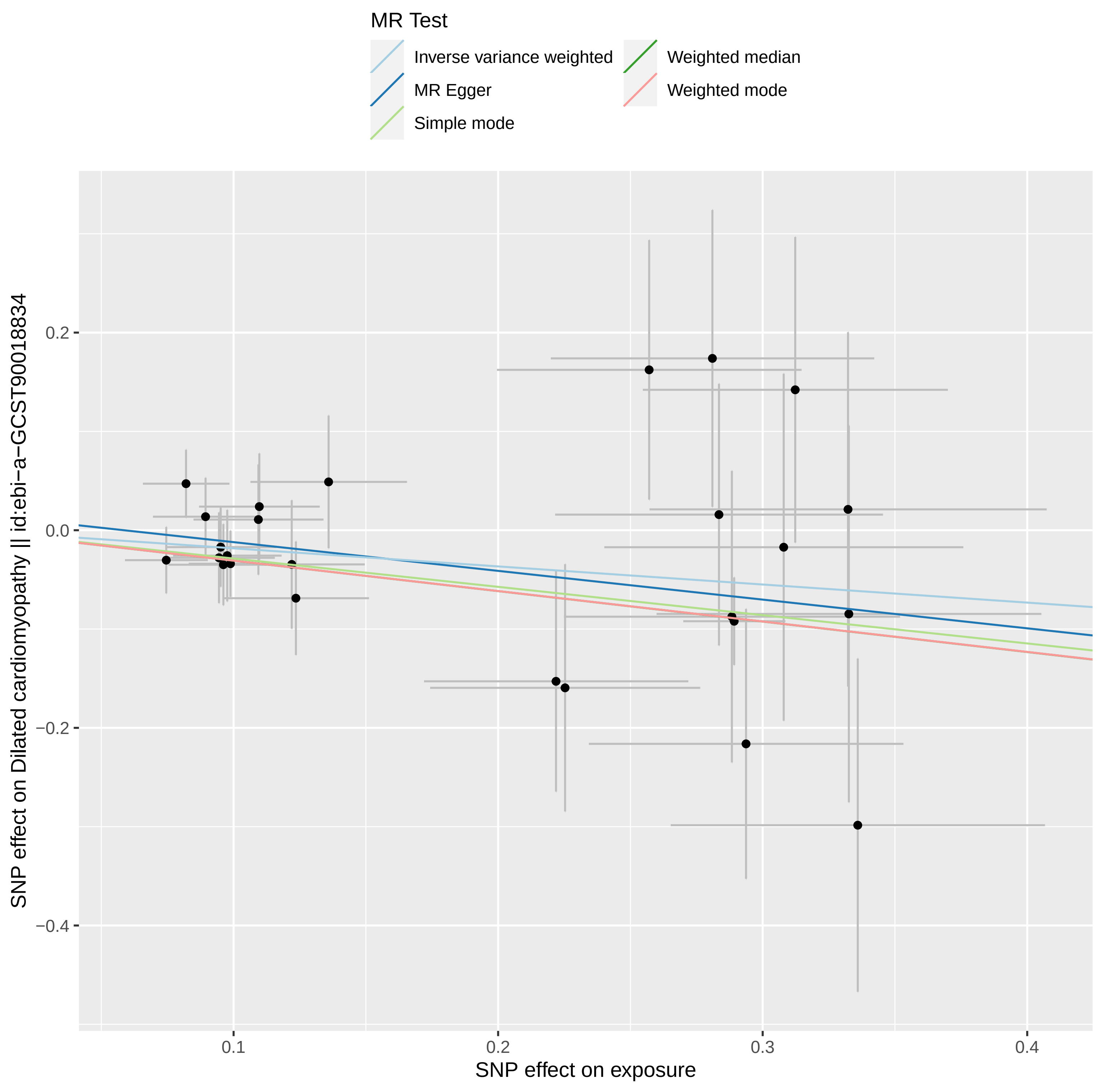
MR Test
Inverse variance weighted
MR Egger
Simple mode
Weighted median
Weighted mode
0.2
0.0
−0.2
−0.4
SNP effect on Dilated cardiomyopathy || id:ebi−a−GCST90018834
0.1
0.2
0.3
0.4
SNP effect on exposure

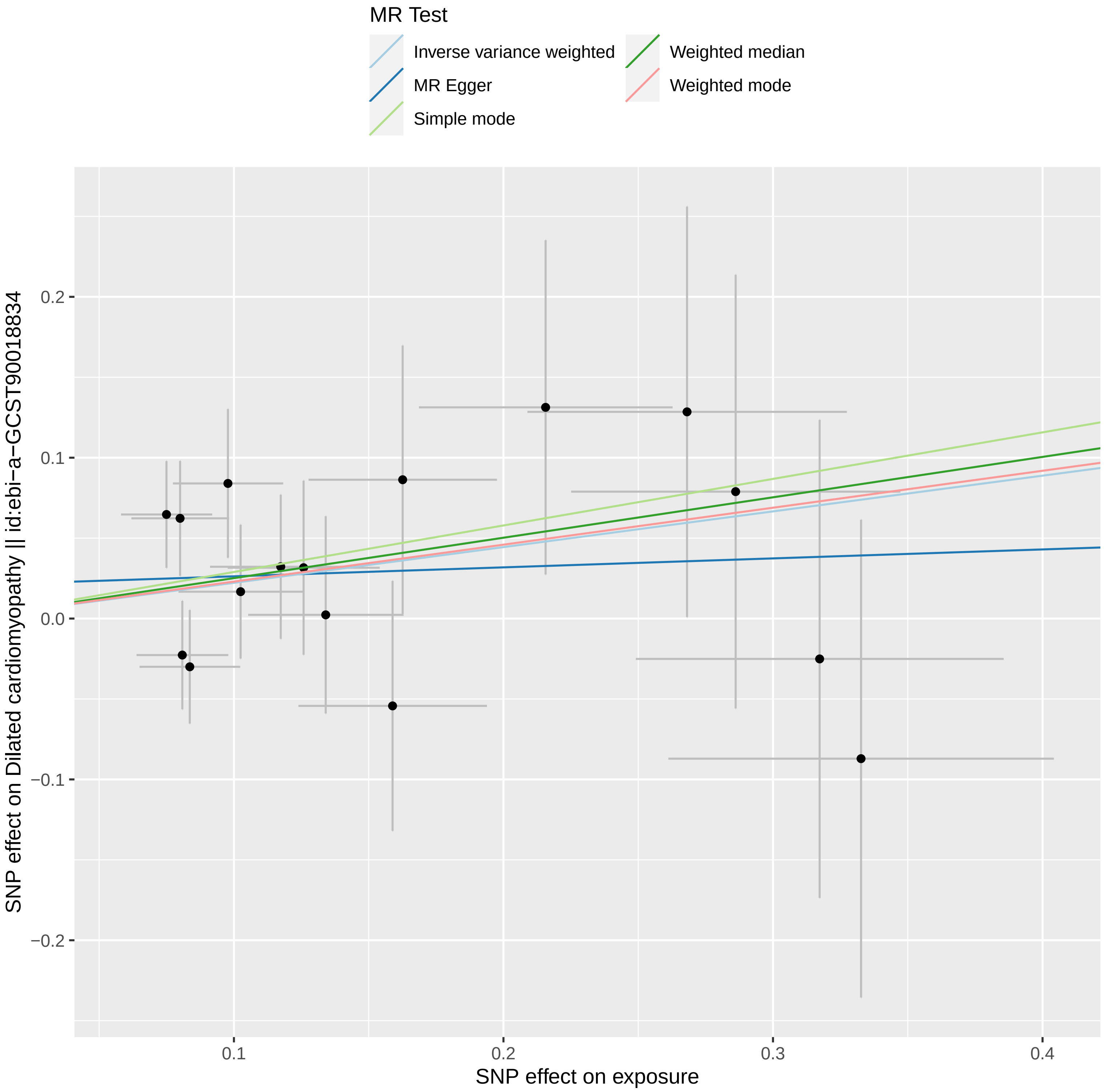
MR Test
Inverse variance weighted
MR Egger
Simple mode
Weighted median
Weighted mode
0.2
0.1
0.0
−0.1
−0.2
SNP effect on Dilated cardiomyopathy || id:ebi−a−GCST90018834
0.1
0.2
0.3
0.4
SNP effect on exposure

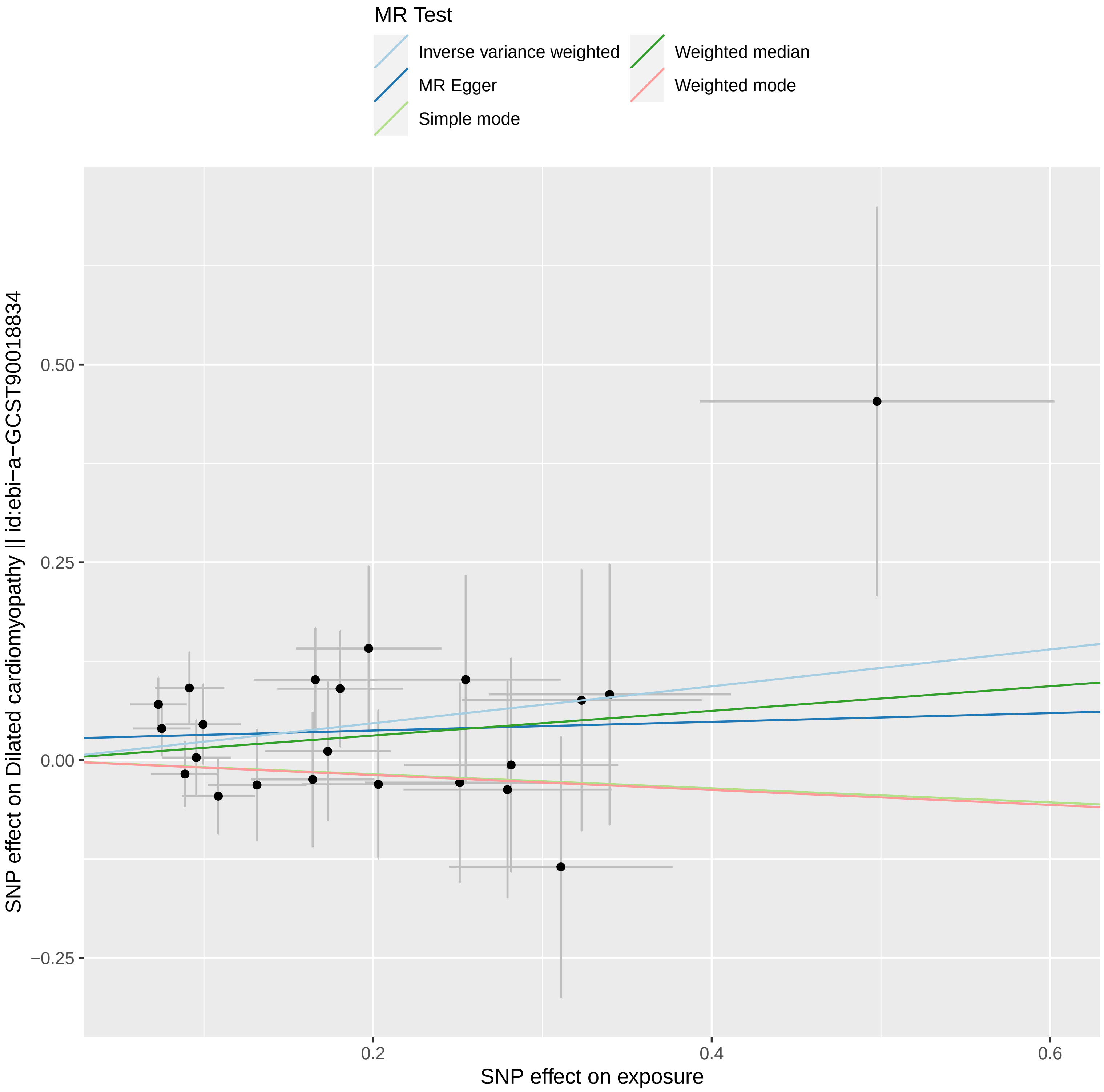
MR Test
Inverse variance weighted
MR Egger
Simple mode
Weighted median
Weighted mode
SNP effect on Dilated cardiomyopathy || id:ebi−a−GCST90018834
0.50
0.25
0.00
−0.25
0.2
0.4
0.6
SNP effect on exposure

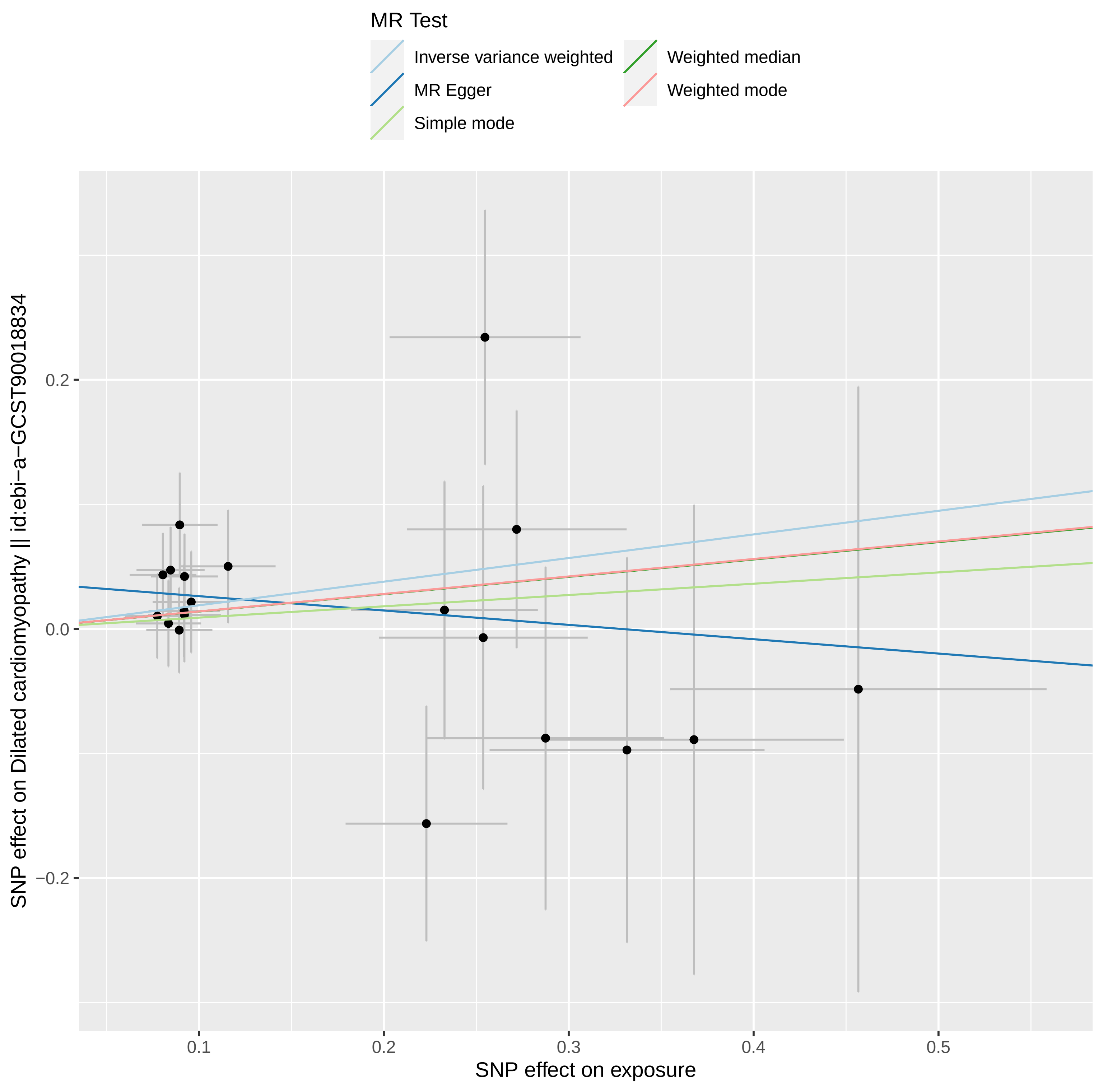
MR Test
Inverse variance weighted
MR Egger
Simple mode
Weighted median
Weighted mode
0.2
0.0
−0.2
0.1
0.2
0.3
0.4
0.5
SNP effect on exposure
SNP effect on Dilated cardiomyopathy || id:ebi−a−GCST90018834

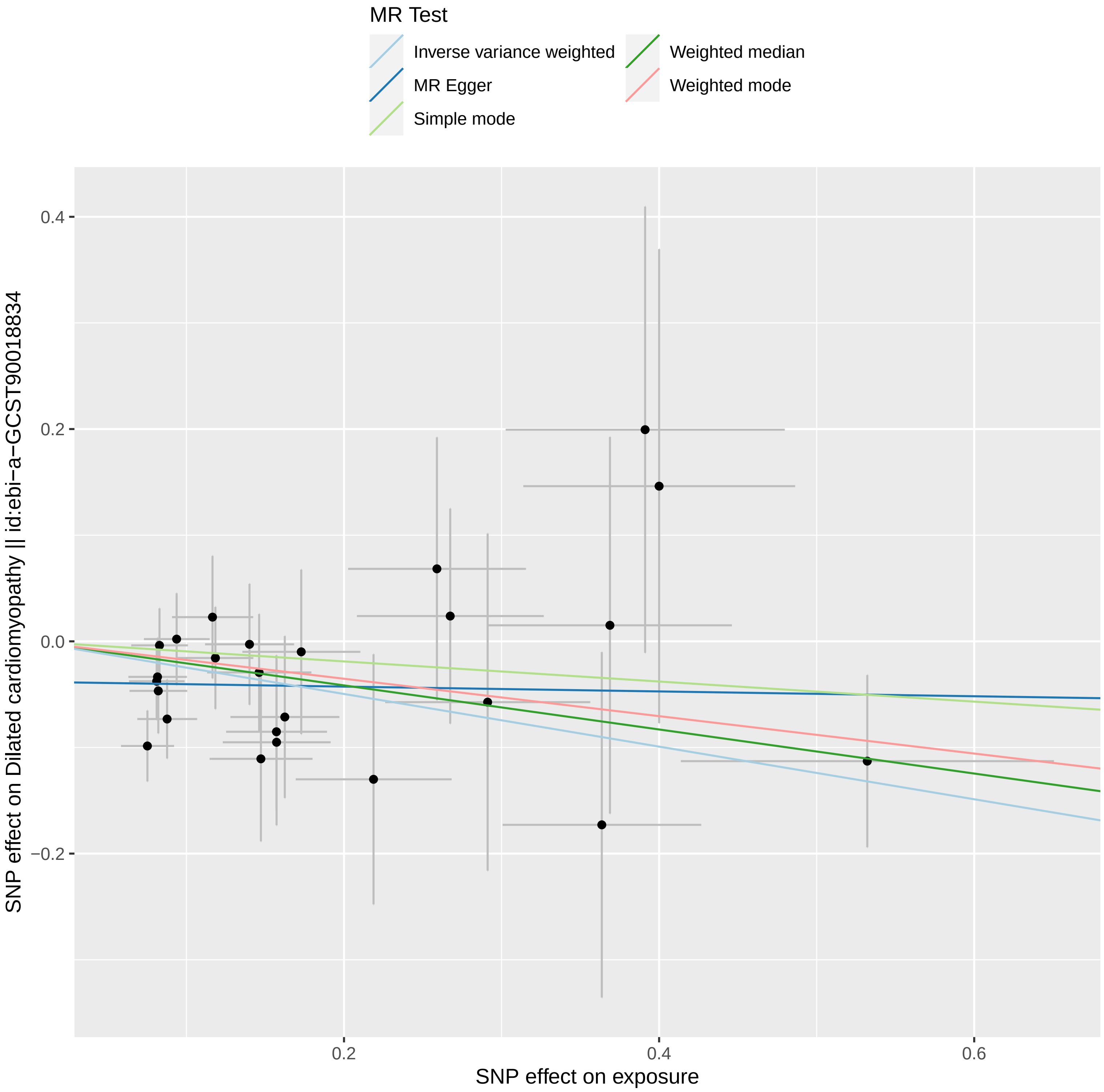
MR Test
Inverse variance weighted
MR Egger
Simple mode
Weighted median
Weighted mode
0.4
0.2
0.0
−0.2
SNP effect on Dilated cardiomyopathy || id:ebi−a−GCST90018834
0.2
0.4
0.6
SNP effect on exposure

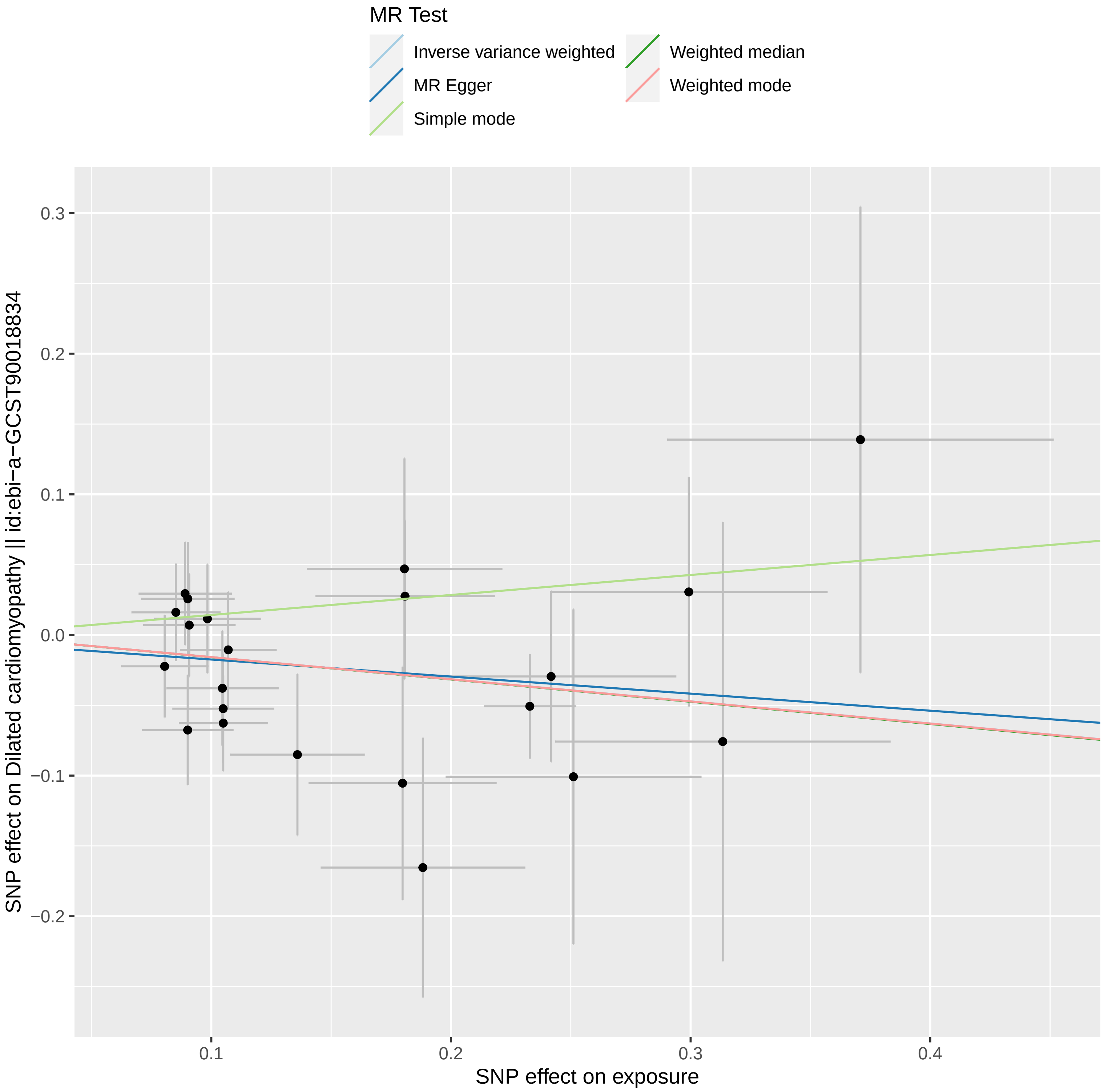

MR Test
Inverse variance weighted
MR Egger
Simple mode
Weighted median
Weighted mode
0.3
0.2
0.1
0.0
−0.1
−0.2
SNP effect on Dilated cardiomyopathy || id:ebi−a−GCST90018834
0.1
0.2
0.3
0.4
SNP effect on exposure

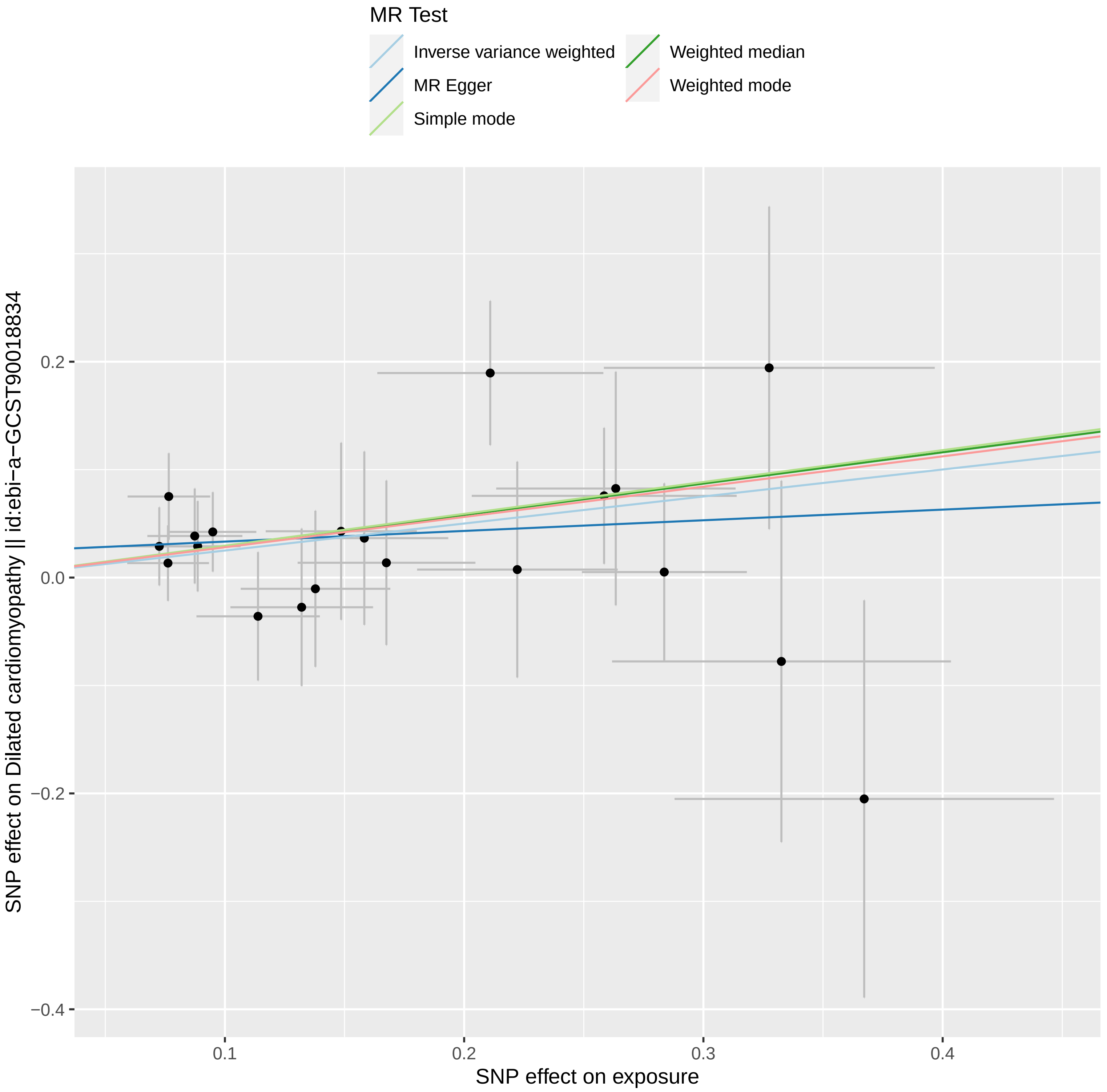

MR Test
Inverse variance weighted
MR Egger
Simple mode
Weighted median
Weighted mode
SNP effect on Dilated cardiomyopathy || id:ebi-a-GCST90018834
0.2
0.0
−0.2
−0.4
0.1
0.2
0.3
0.4
SNP effect on exposure

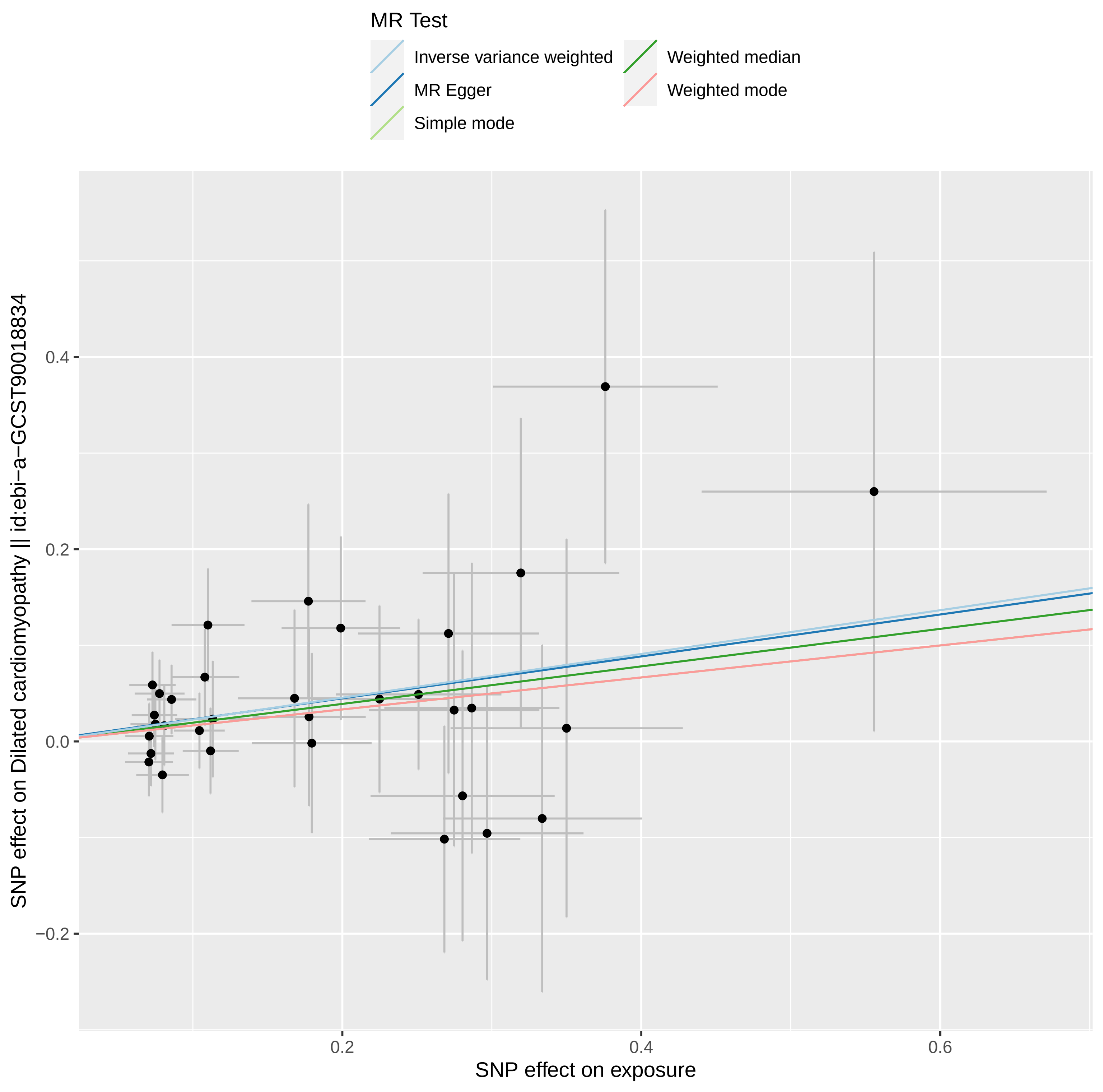
MR Test
Inverse variance weighted
MR Egger
Simple mode
Weighted median
Weighted mode
SNP effect on Dilated cardiomyopathy || id:ebi−a−GCST90018834
0.4
0.2
0.0
−0.2
0.2
0.4
0.6
SNP effect on exposure

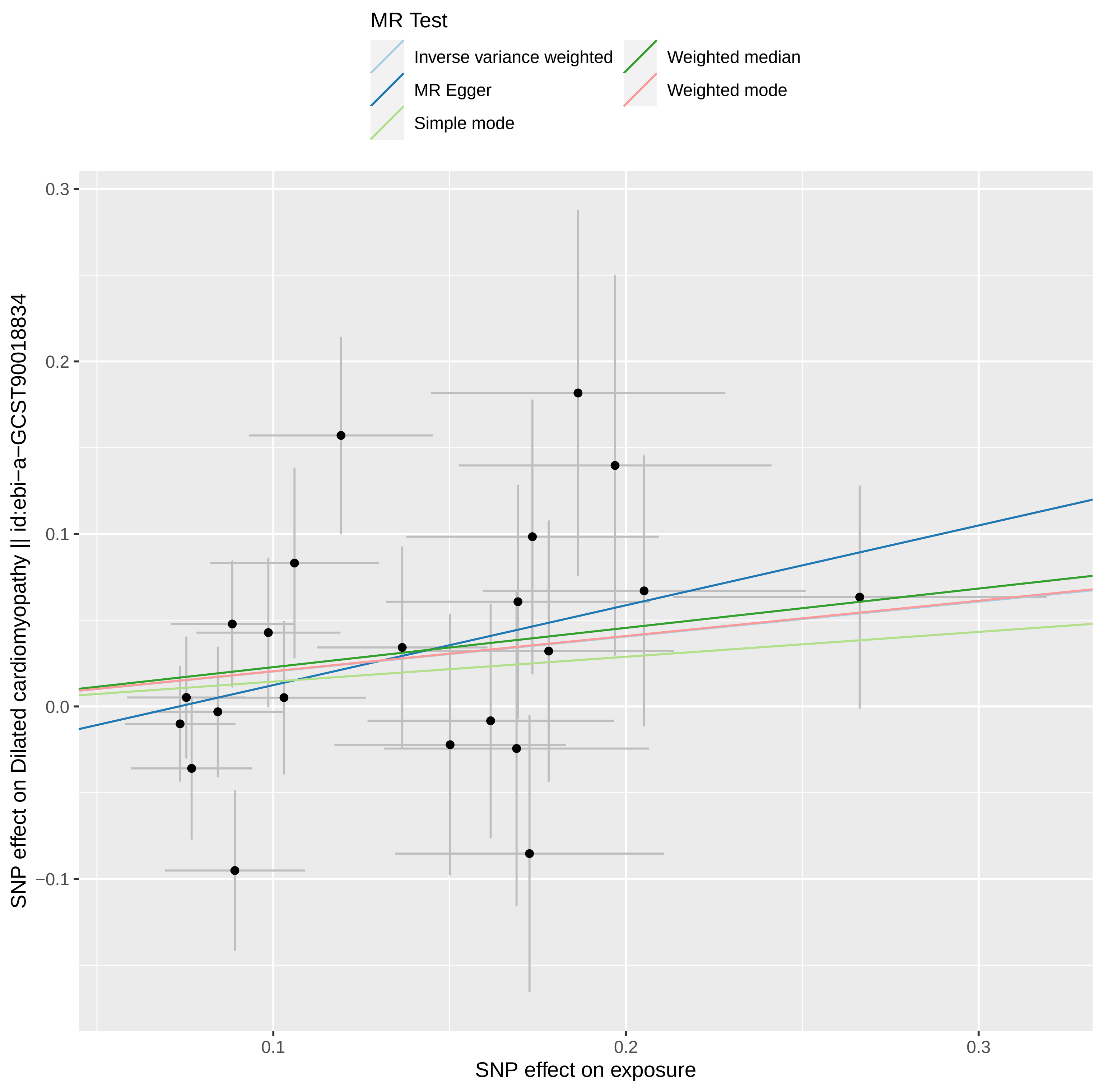
MR Test
Inverse variance weighted
MR Egger
Simple mode
Weighted median
Weighted mode
SNP effect on Dilated cardiomyopathy || id:ebi−a−GCST90018834
0.3
0.2
0.1
0.0
−0.1
0.1
0.2
0.3
SNP effect on exposure

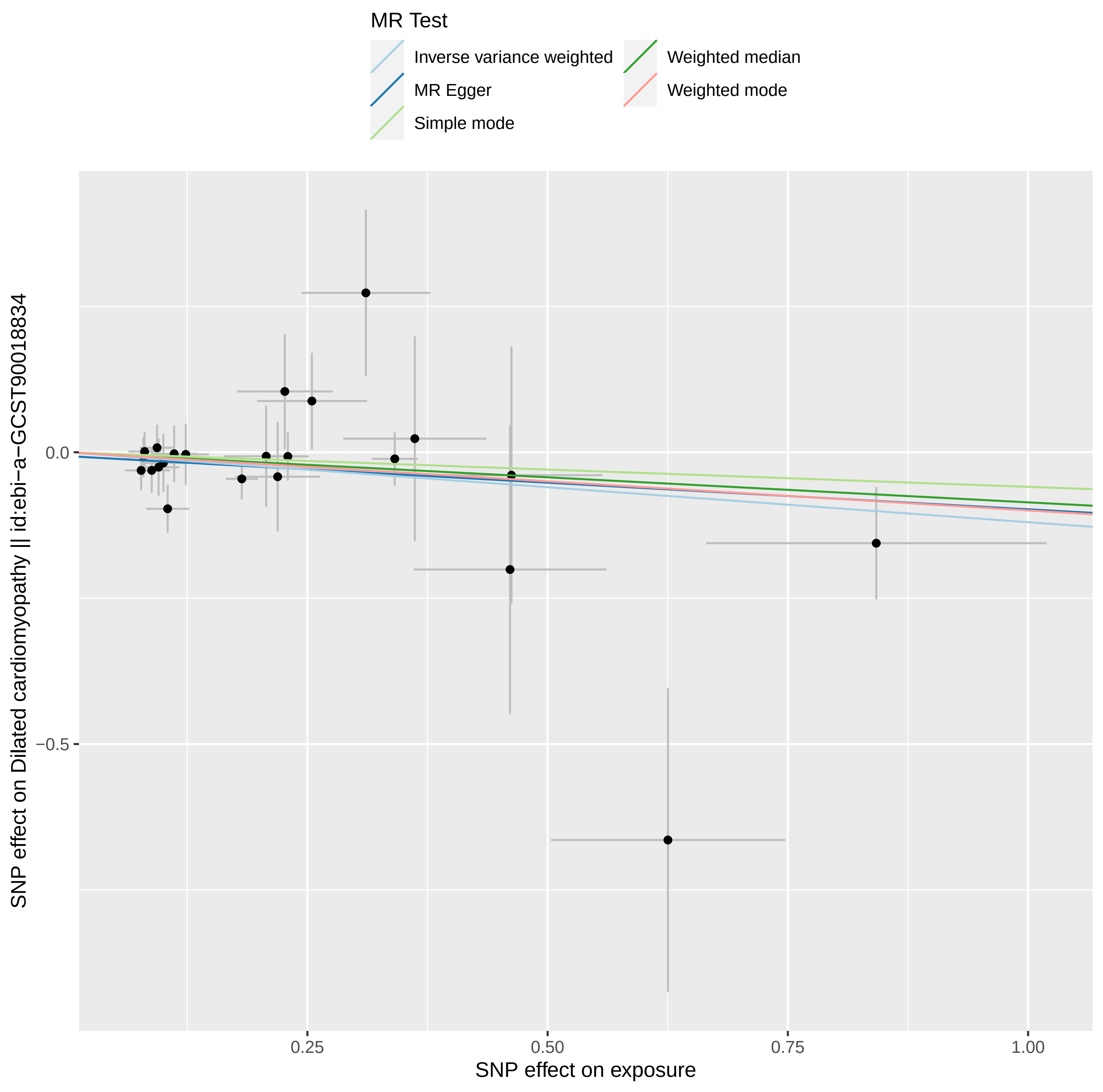
MR Test
Inverse variance weighted
MR Egger
Simple mode
Weighted median
Weighted mode
SNP effect on Dilated cardiomyopathy || id:ebi-a-GCST90018834
0.0
−0.5
0.25
0.50
0.75
1.00
SNP effect on exposure

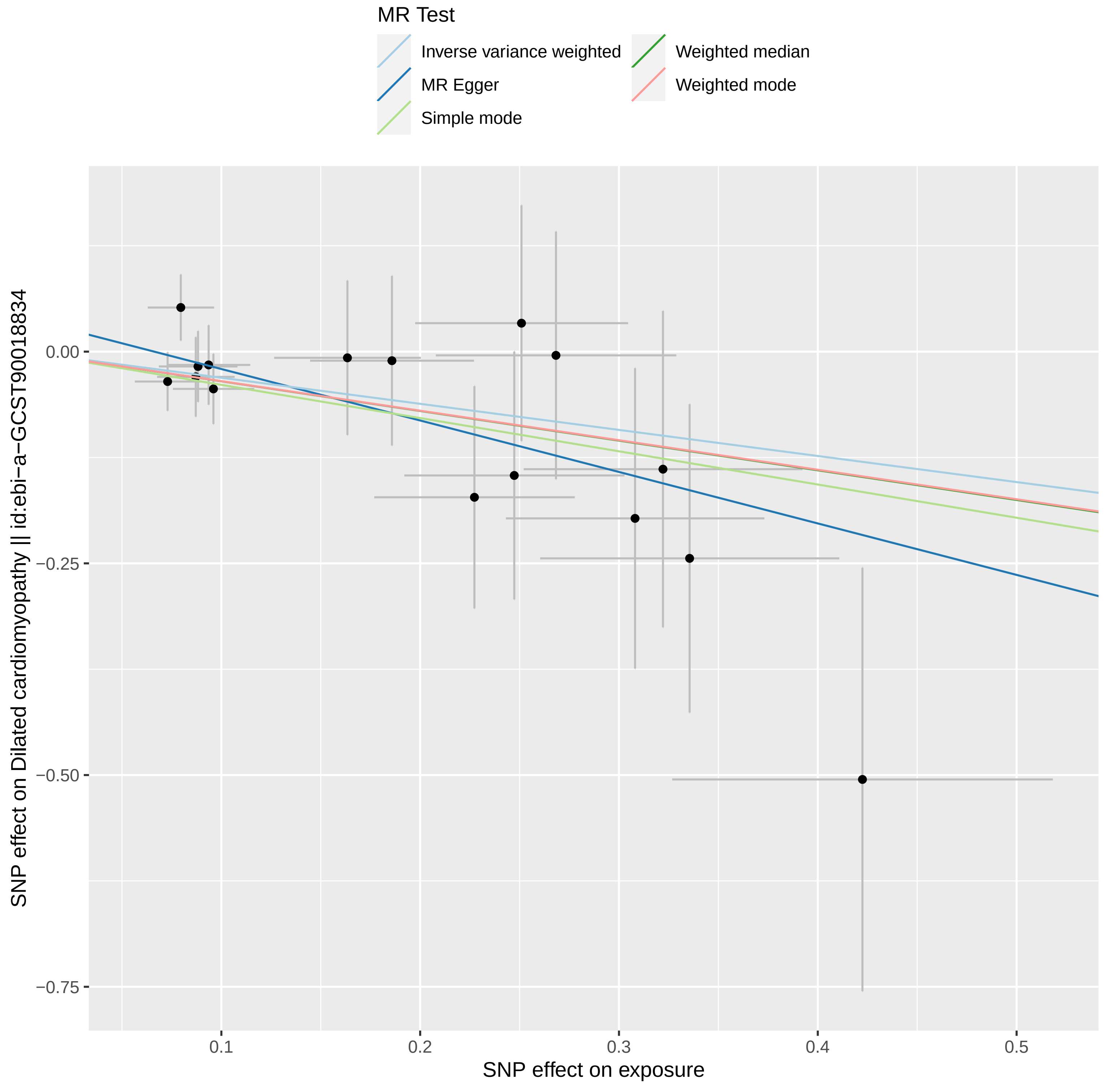
MR Test
Inverse variance weighted
MR Egger
Simple mode
Weighted median
Weighted mode
SNP effect on Dilated cardiomyopathy || id:ebi−a−GCST90018834
0.00
−0.25
−0.50
−0.75
0.1
0.2
0.3
0.4
0.5
SNP effect on exposure

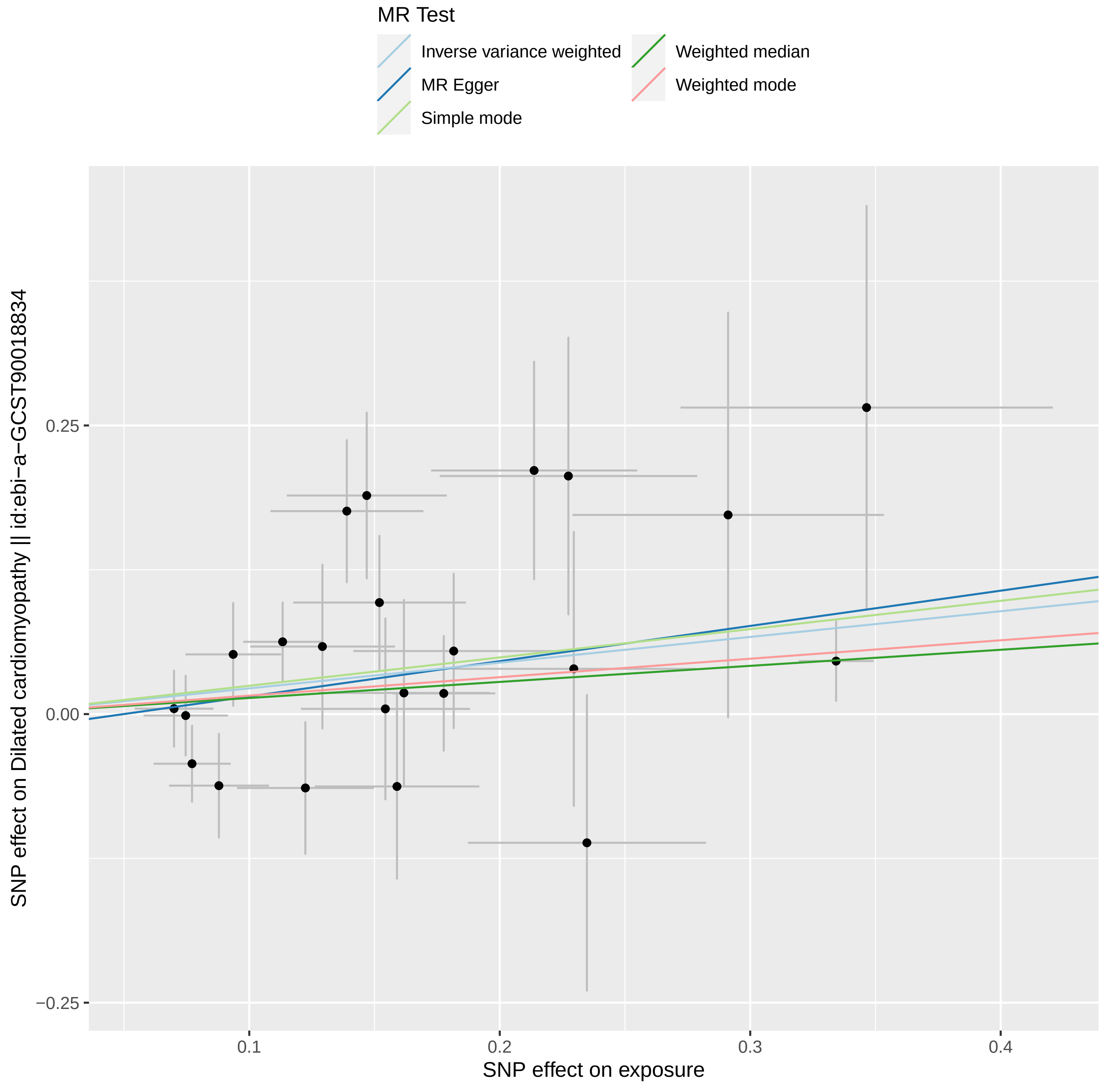

MR Test
Inverse variance weighted
MR Egger
Simple mode
Weighted median
Weighted mode
SNP effect on Dilated cardiomyopathy || id:ebi−a−GCST90018834
0.25
0.00
−0.25
0.1
0.2
0.3
0.4
SNP effect on exposure

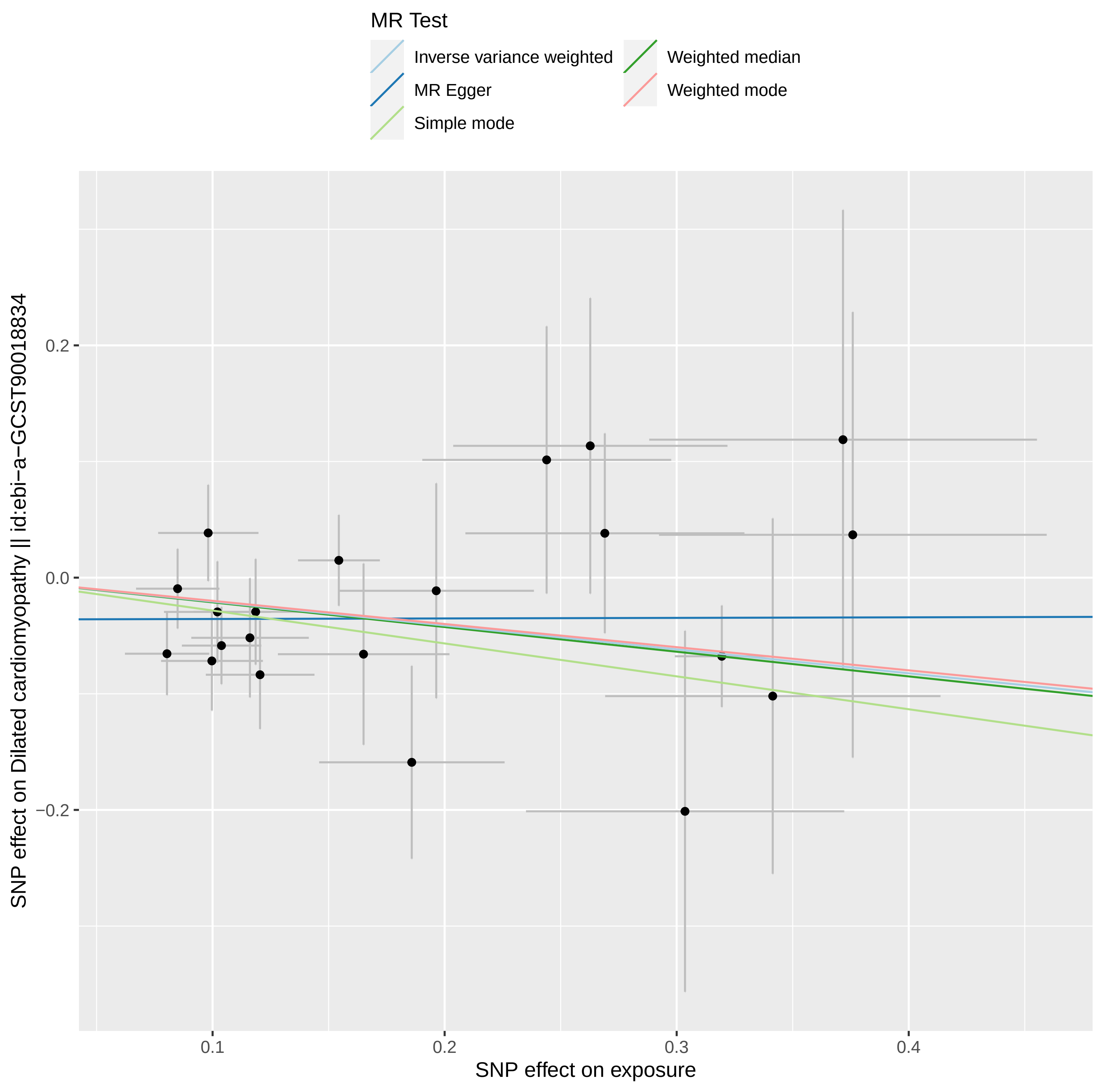

MR Test
Inverse variance weighted
MR Egger
Simple mode
Weighted median
Weighted mode
0.2
0.0
−0.2
0.1
0.2
0.3
0.4
SNP effect on exposure
SNP effect on Dilated cardiomyopathy || id:ebi−a−GCST90018834

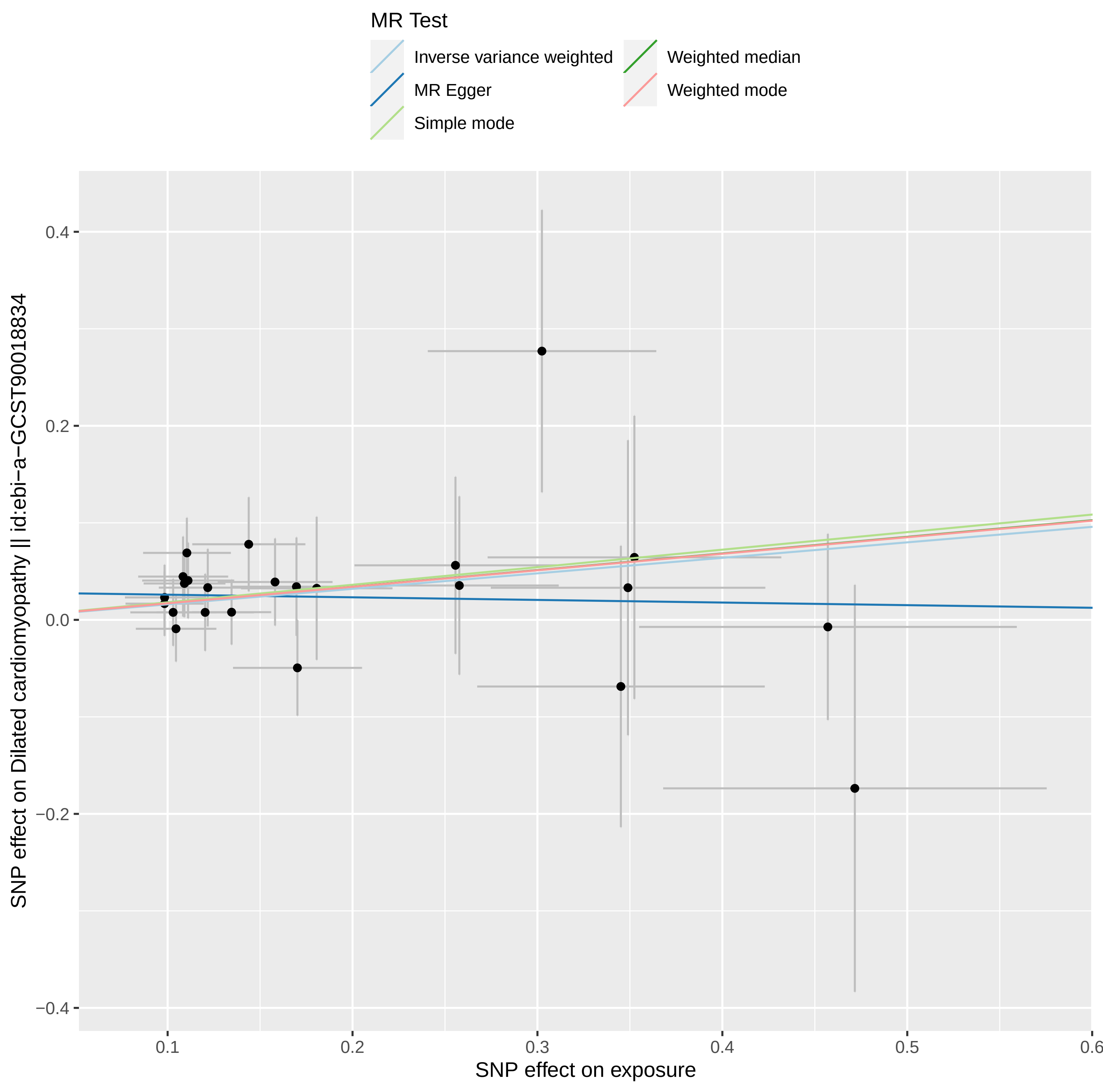
MR Test
Inverse variance weighted
MR Egger
Simple mode
Weighted median
Weighted mode
0.4
0.2
0.0
−0.2
−0.4
0.1
0.2
0.3
0.4
0.5
0.6
SNP effect on Dilated cardiomyopathy || id:ebi−a−GCST90018834
SNP effect on exposure

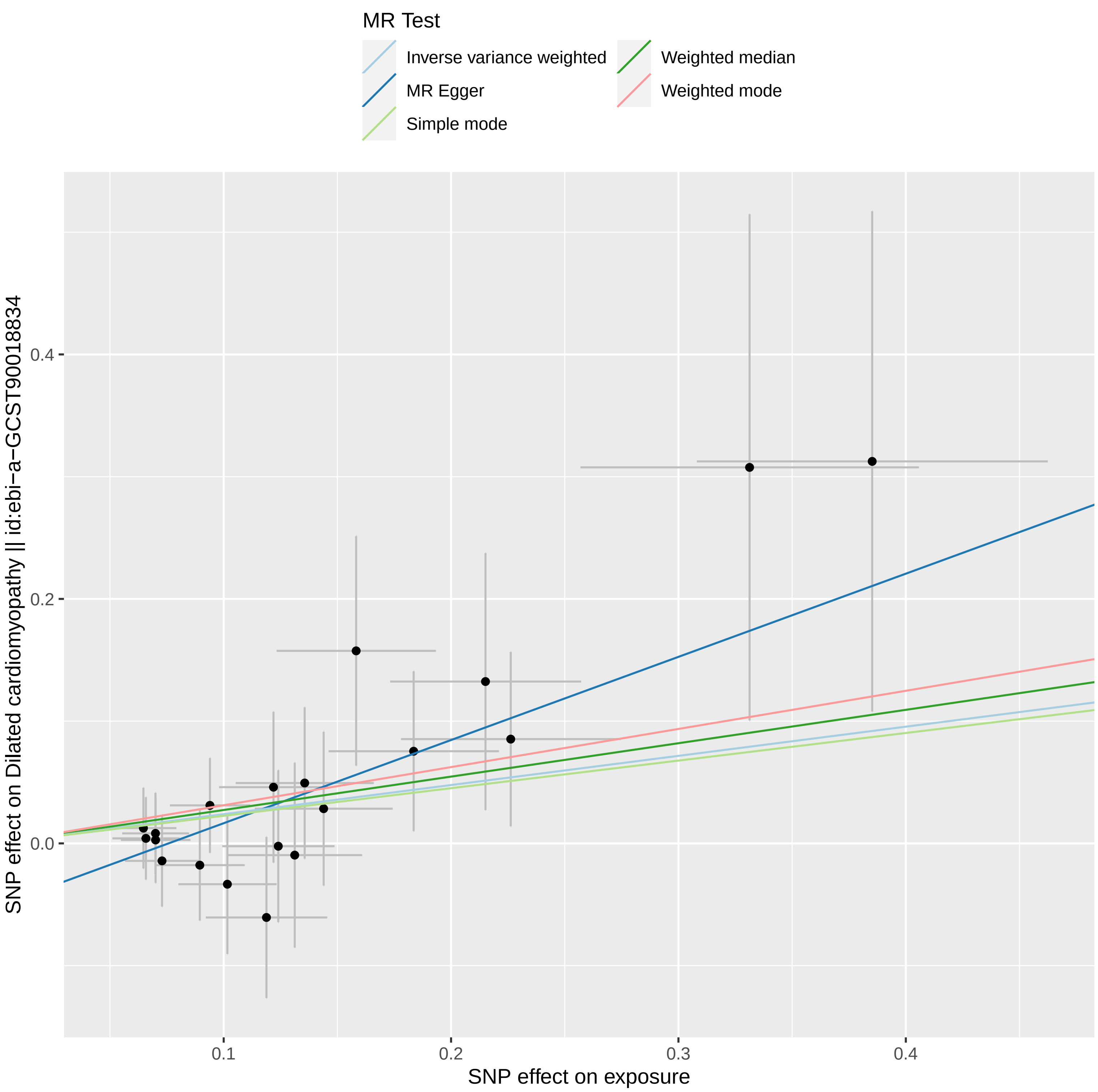
MR Test
Inverse variance weighted
MR Egger
Simple mode
Weighted median
Weighted mode
SNP effect on Dilated cardiomyopathy || id:ebi−a−GCST90018834
0.4
0.2
0.0
0.1
0.2
0.3
0.4
SNP effect on exposure

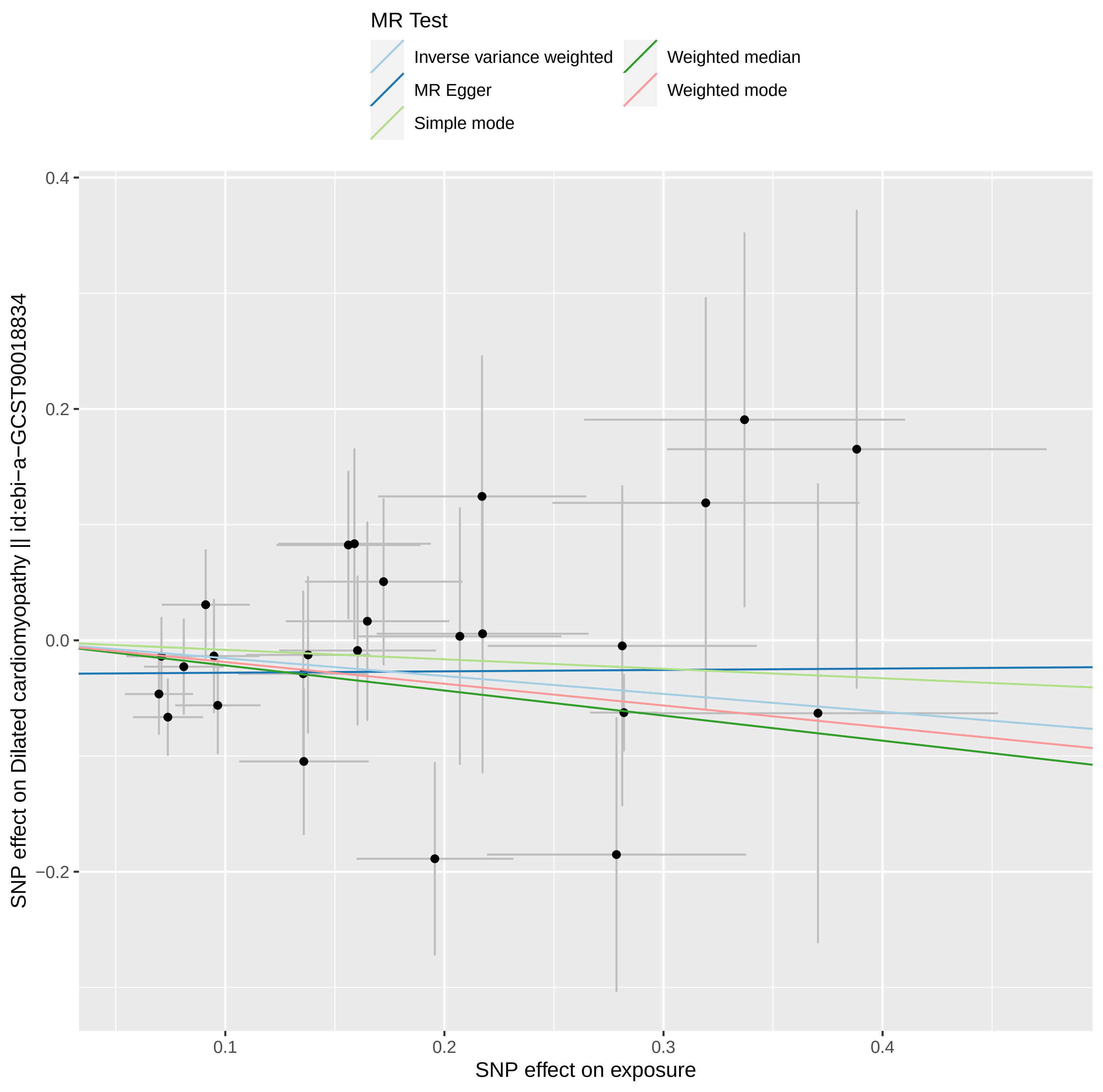
MR Test
Inverse variance weighted
MR Egger
Simple mode
Weighted median
Weighted mode
0.4
0.2
0.0
−0.2
0.1
0.2
0.3
0.4
SNP effect on exposure
SNP effect on Dilated cardiomyopathy || id:ebi−a−GCST90018834

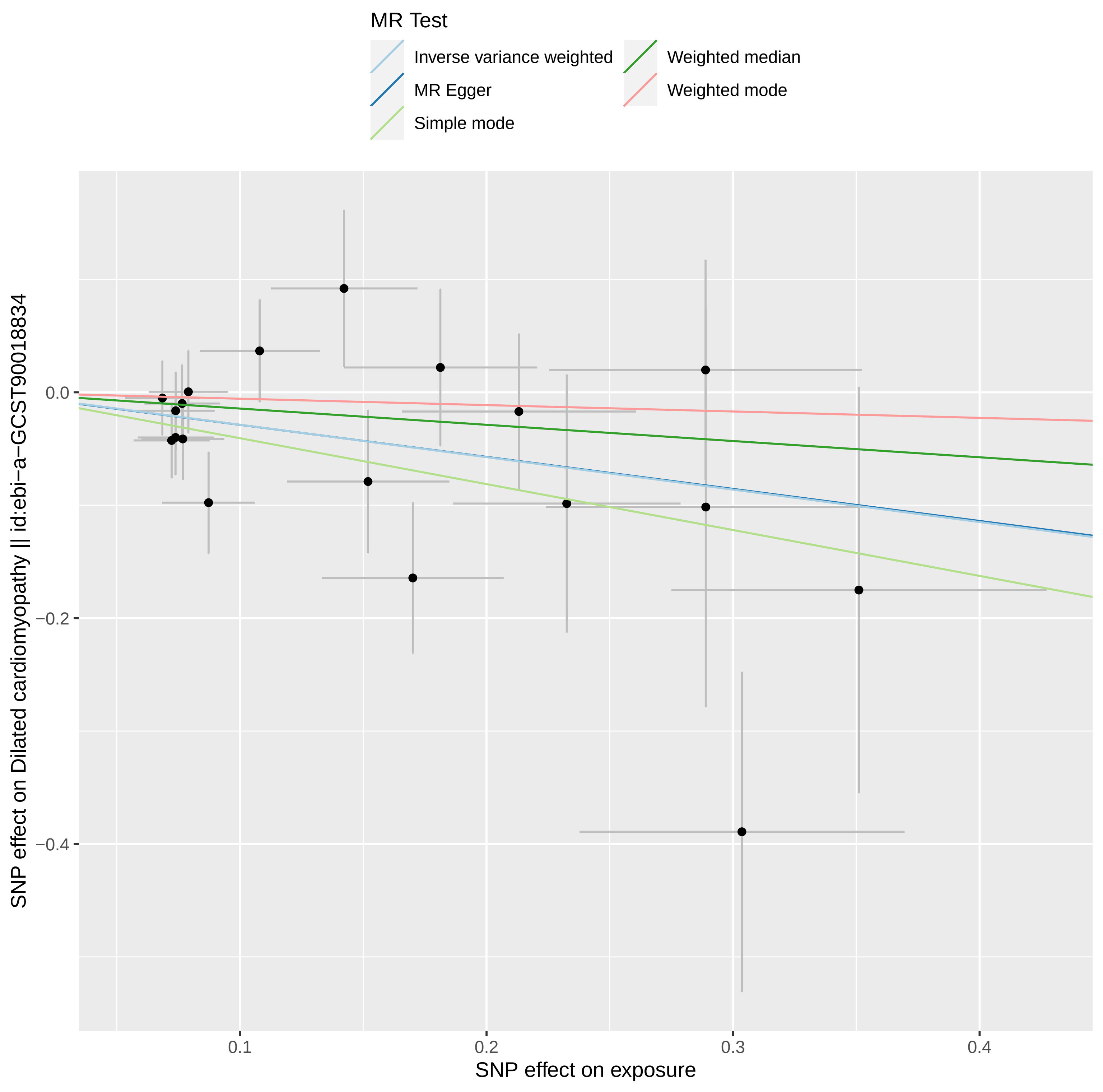

MR Test
Inverse variance weighted
MR Egger
Simple mode
Weighted median
Weighted mode
0.0
−0.2
−0.4
0.1
0.2
0.3
0.4
SNP effect on exposure
SNP effect on Dilated cardiomyopathy || id:ebi−a−GCST90018834

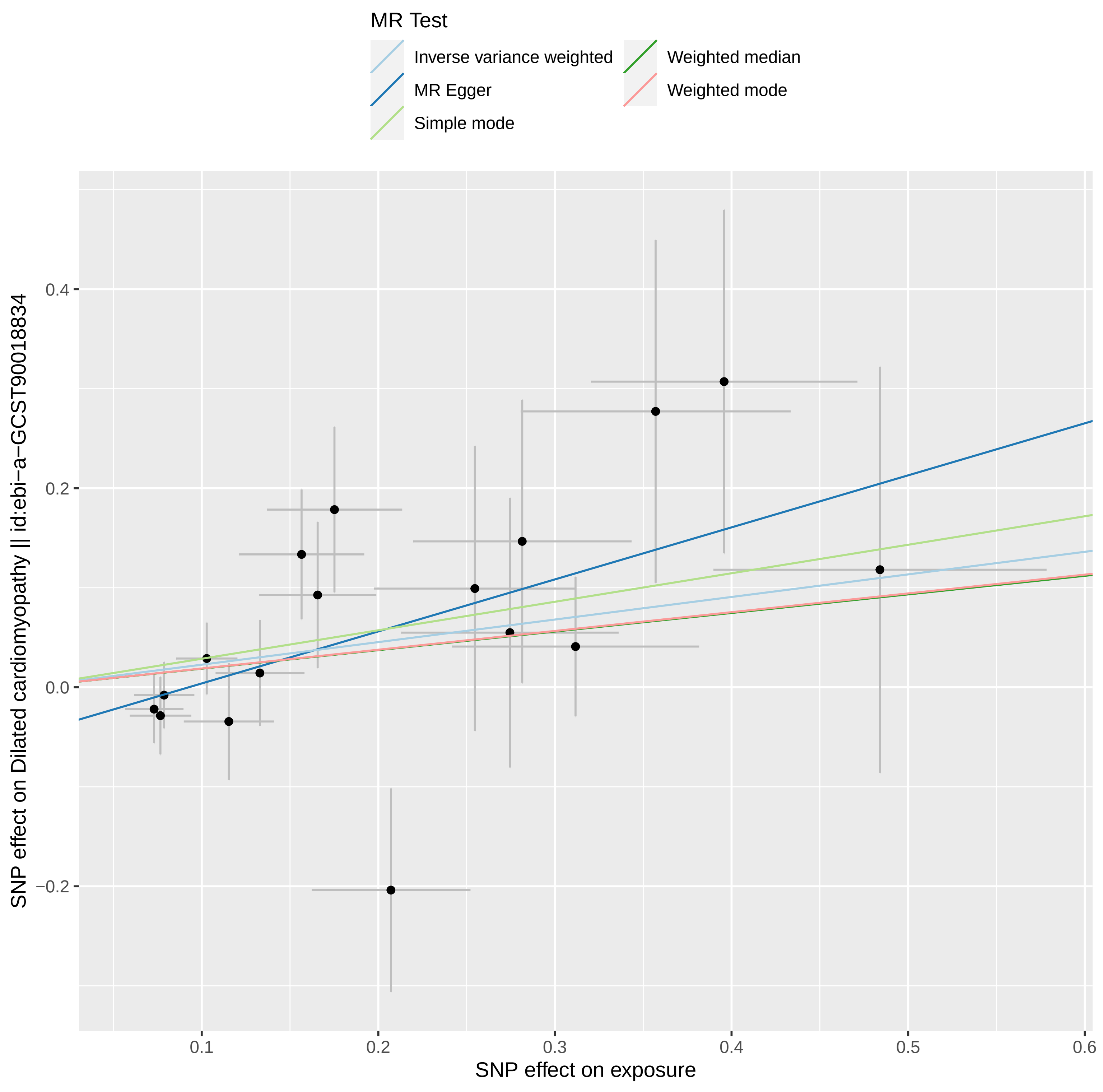

MR Test
Inverse variance weighted
MR Egger
Simple mode
Weighted median
Weighted mode
0.4
0.2
0.0
−0.2
0.1
0.2
0.3
0.4
0.5
0.6
SNP effect on exposure
SNP effect on Dilated cardiomyopathy || id:ebi−a−GCST90018834

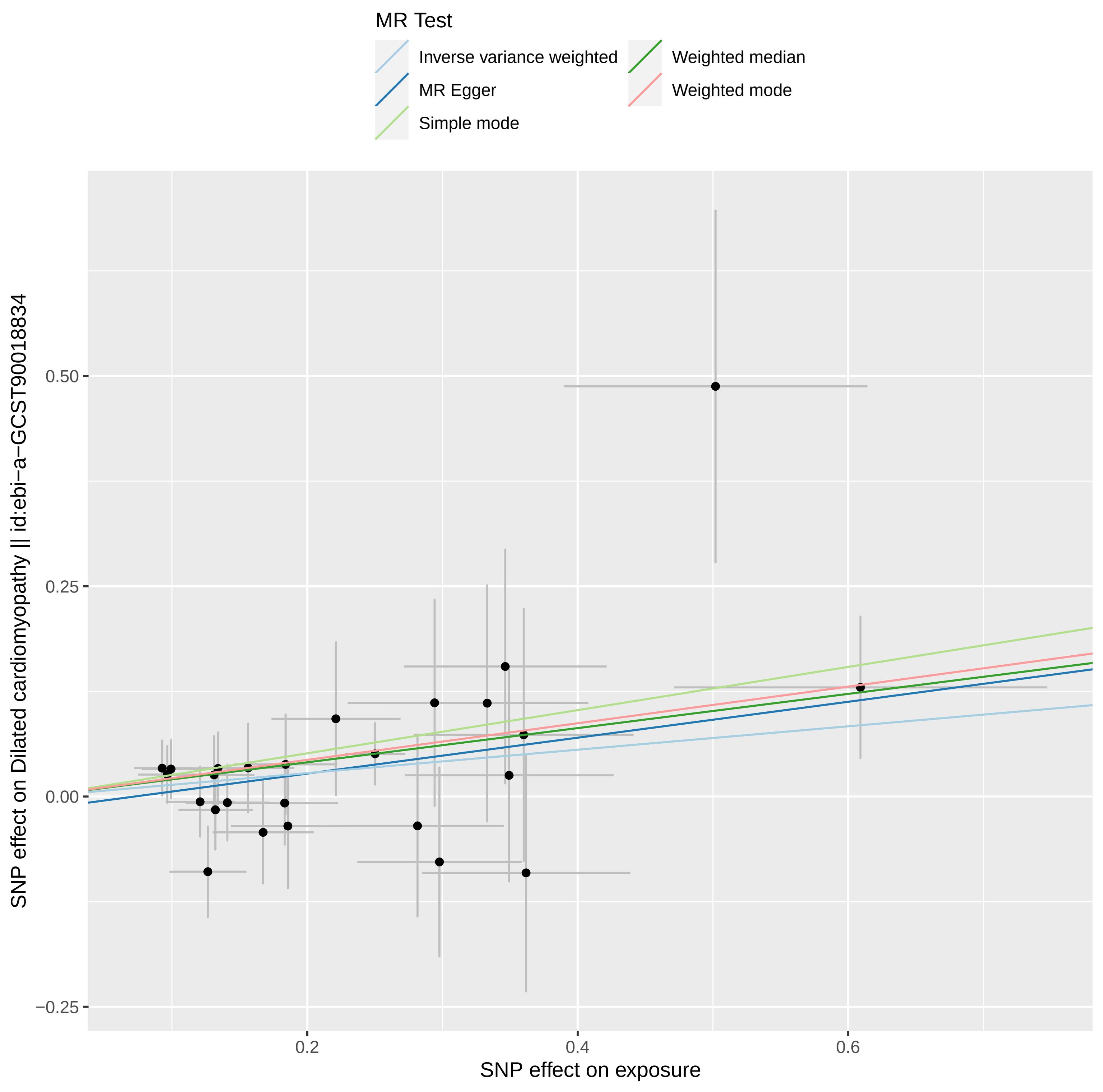

MR Test
Inverse variance weighted
MR Egger
Simple mode
Weighted median
Weighted mode
SNP effect on Dilated cardiomyopathy || id:ebi−a−GCST90018834
0.50
0.25
0.00
−0.25
0.2
0.4
0.6
SNP effect on exposure

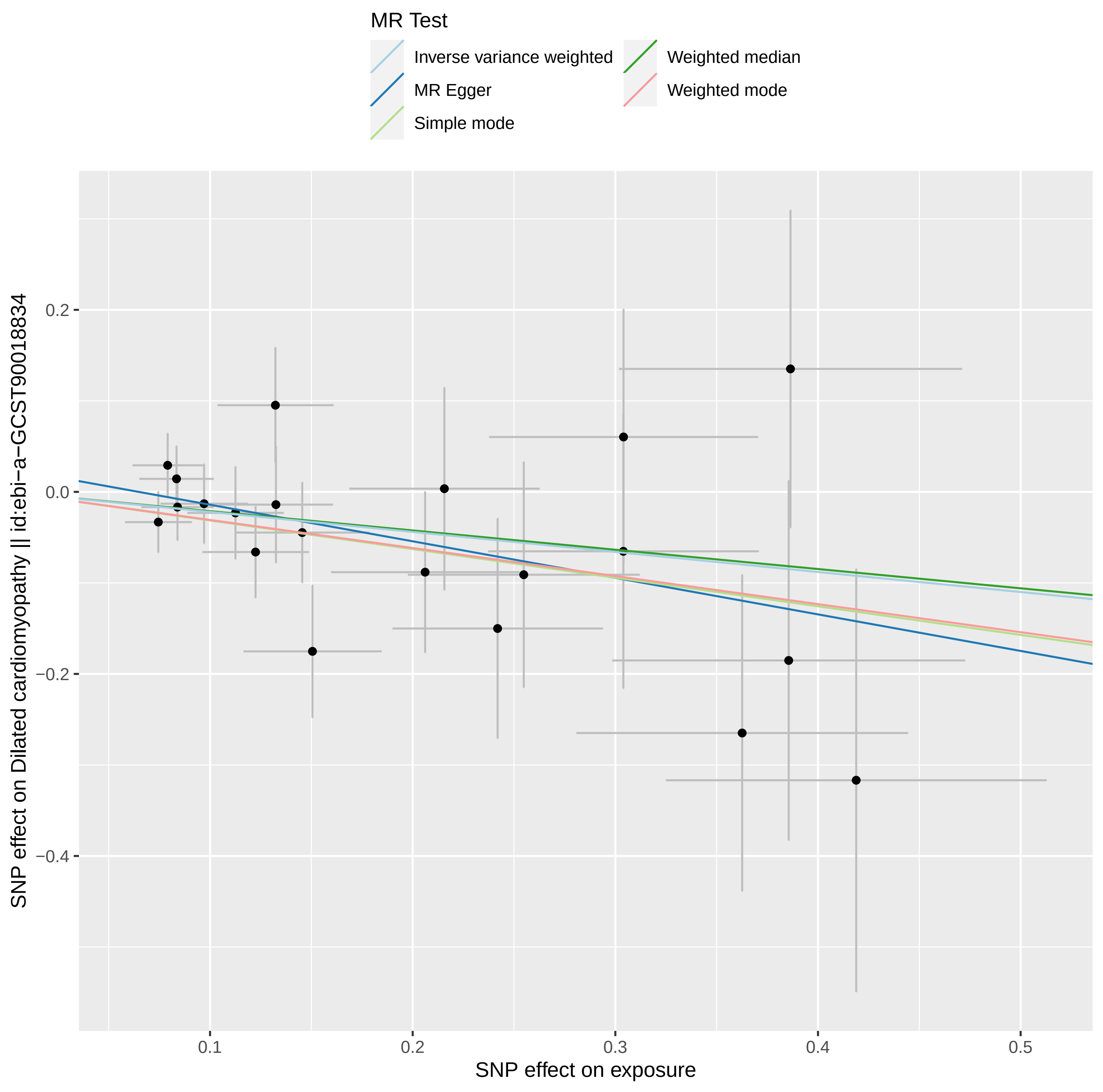

MR Test
Inverse variance weighted
MR Egger
Simple mode
Weighted median
Weighted mode
0.2
0.0
−0.2
−0.4
SNP effect on Dilated cardiomyopathy || id:ebi−a−GCST90018834
0.1
0.2
0.3
0.4
0.5
SNP effect on exposure
