# Supplementary material for: Causal association between 1400 metabolites and dilated cardiomyopathy: a bidirectional two-sample Mendelian randomization analysis
Source: Front Endocrinol (Lausanne). 2024 Sep 12;15:1423142. doi: 10.3389/fendo.2024.1423142 (PMC11424463; doi:10.3389/fendo.2024.1423142)
Supplement: Supplementary file 3 [file DataSheet3.pdf]

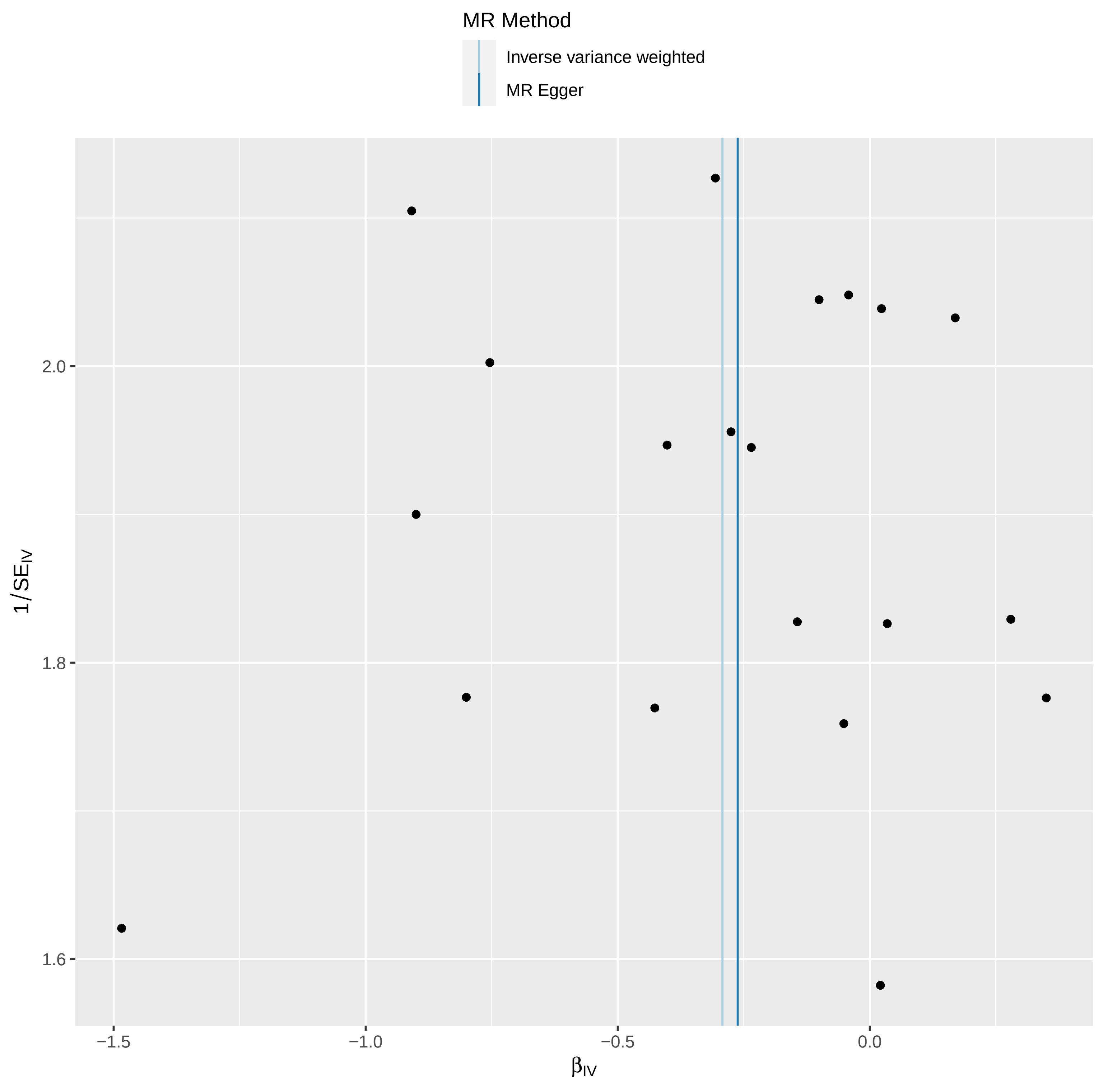
MR Method
Inverse variance weighted
MR Egger
2.0
1.8
1.6
$1/SE_{IV}$
−1.5
−1.0
−0.5
0.0
β_{IV}

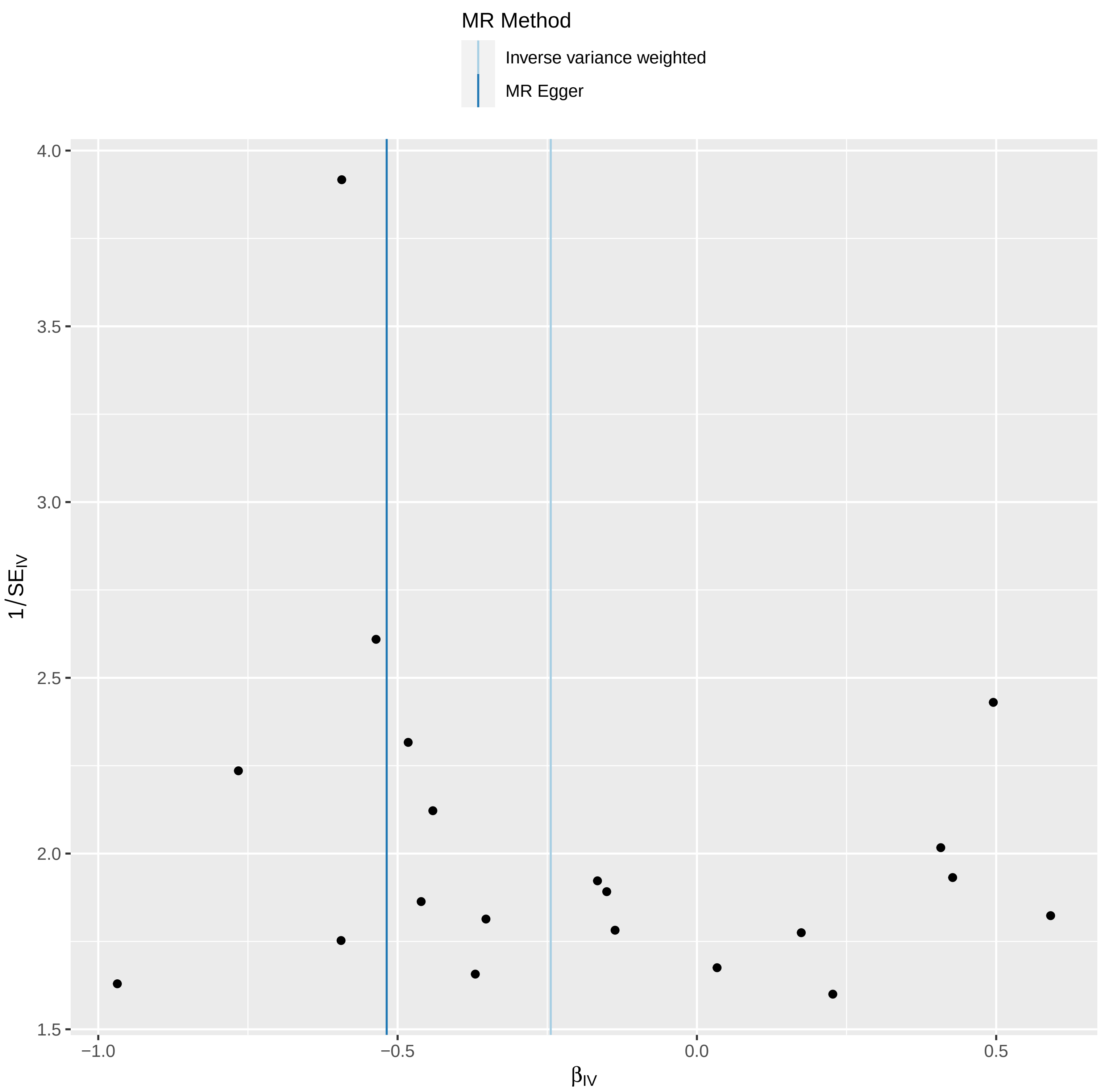
MR Method
Inverse variance weighted
MR Egger
4.0
3.5
3.0
2.5
2.0
1.5
$1/SE_{IV}$
−1.0
−0.5
0.0
0.5
β_{IV}

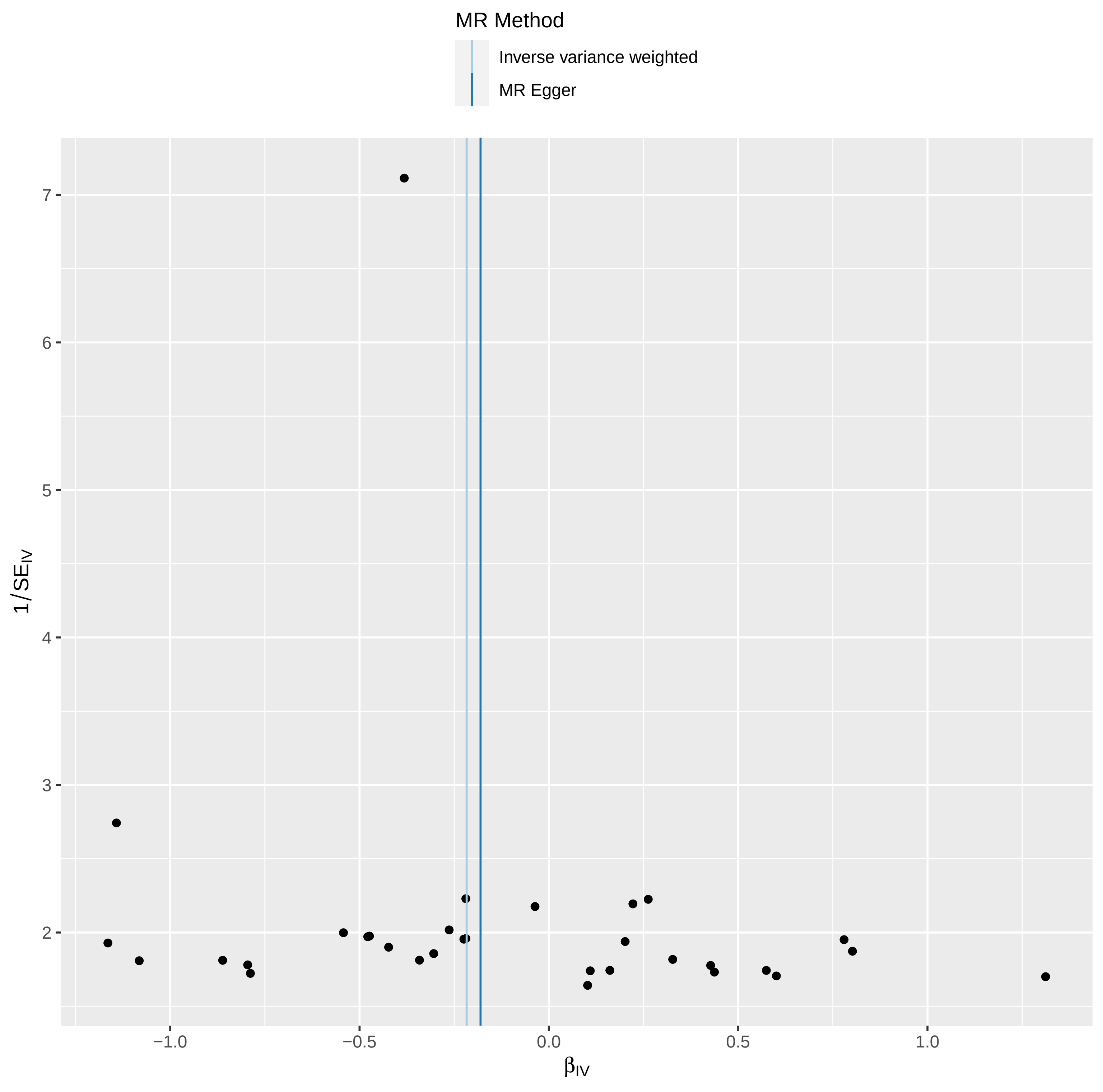
MR Method
Inverse variance weighted
MR Egger
$1/SE_{IV}$
7
6
5
4
3
2
−1.0
−0.5
0.0
0.5
1.0
β_{IV}

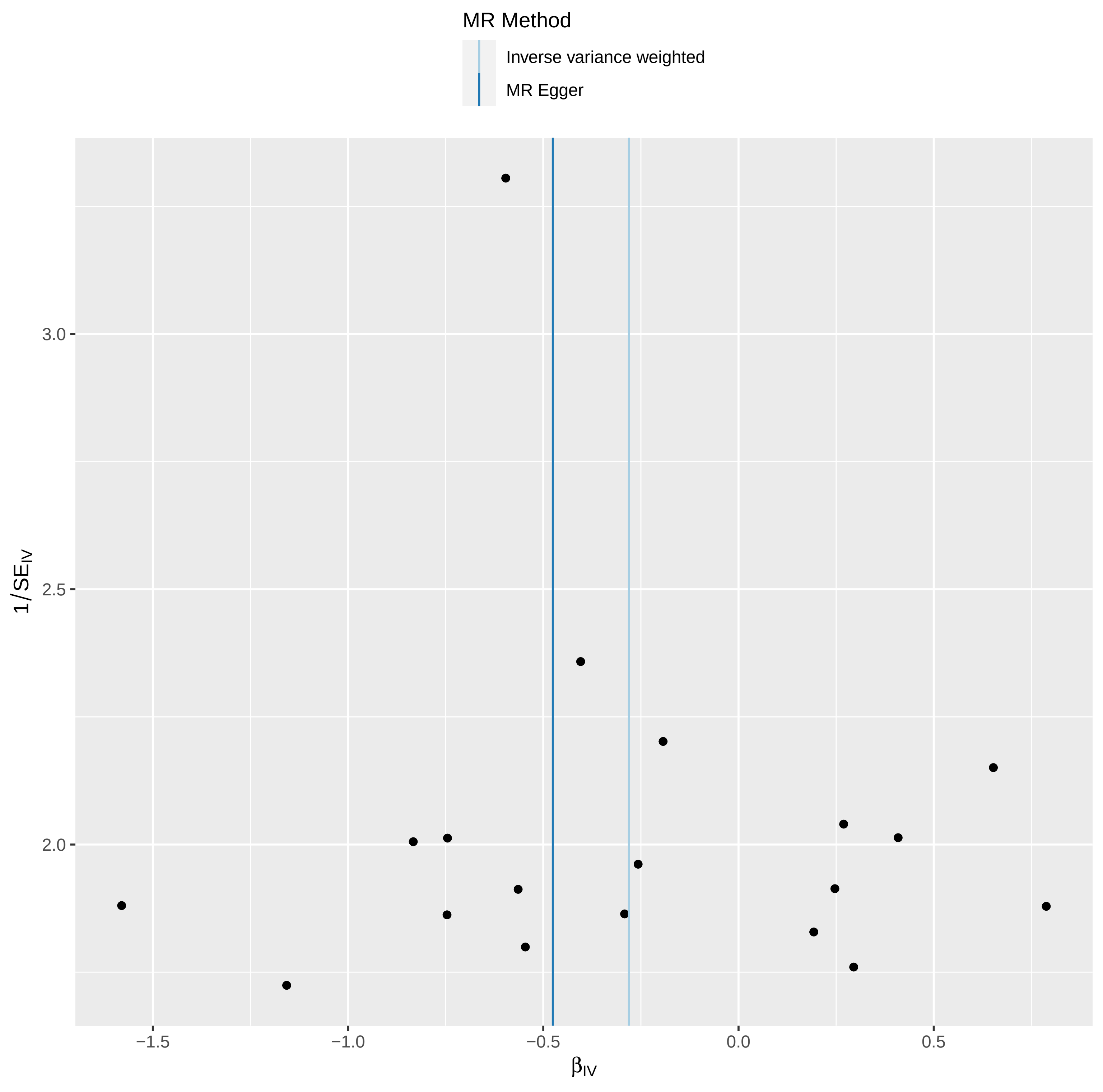

MR Method
Inverse variance weighted
MR Egger
3.0
2.5
2.0
$1/SE_{IV}$
−1.5
−1.0
−0.5
0.0
0.5
β_{IV}

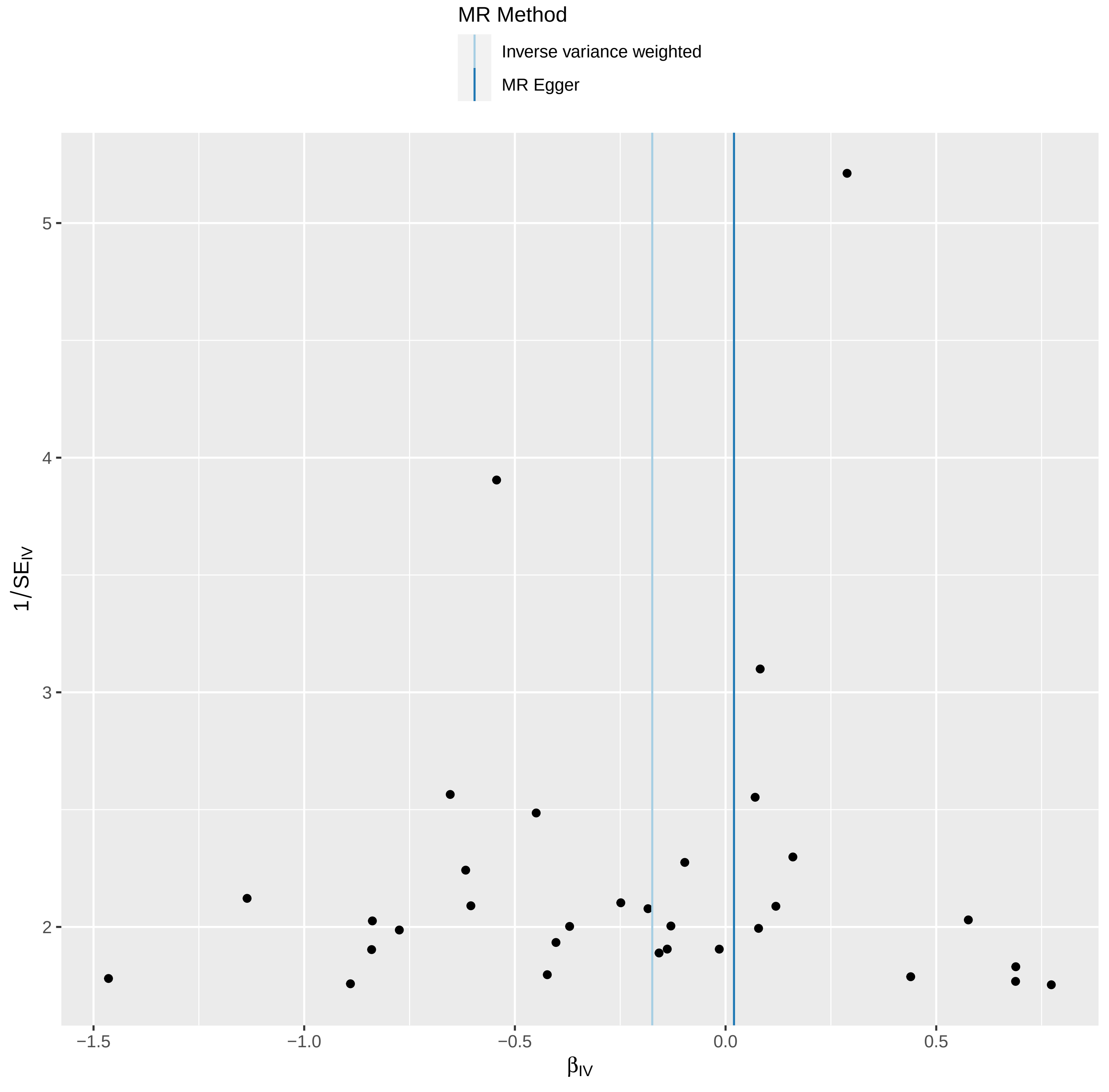

MR Method
Inverse variance weighted
MR Egger
5
4
3
2
1/SE$_{IV}$
−1.5
−1.0
−0.5
0.0
0.5
β$_{IV}$

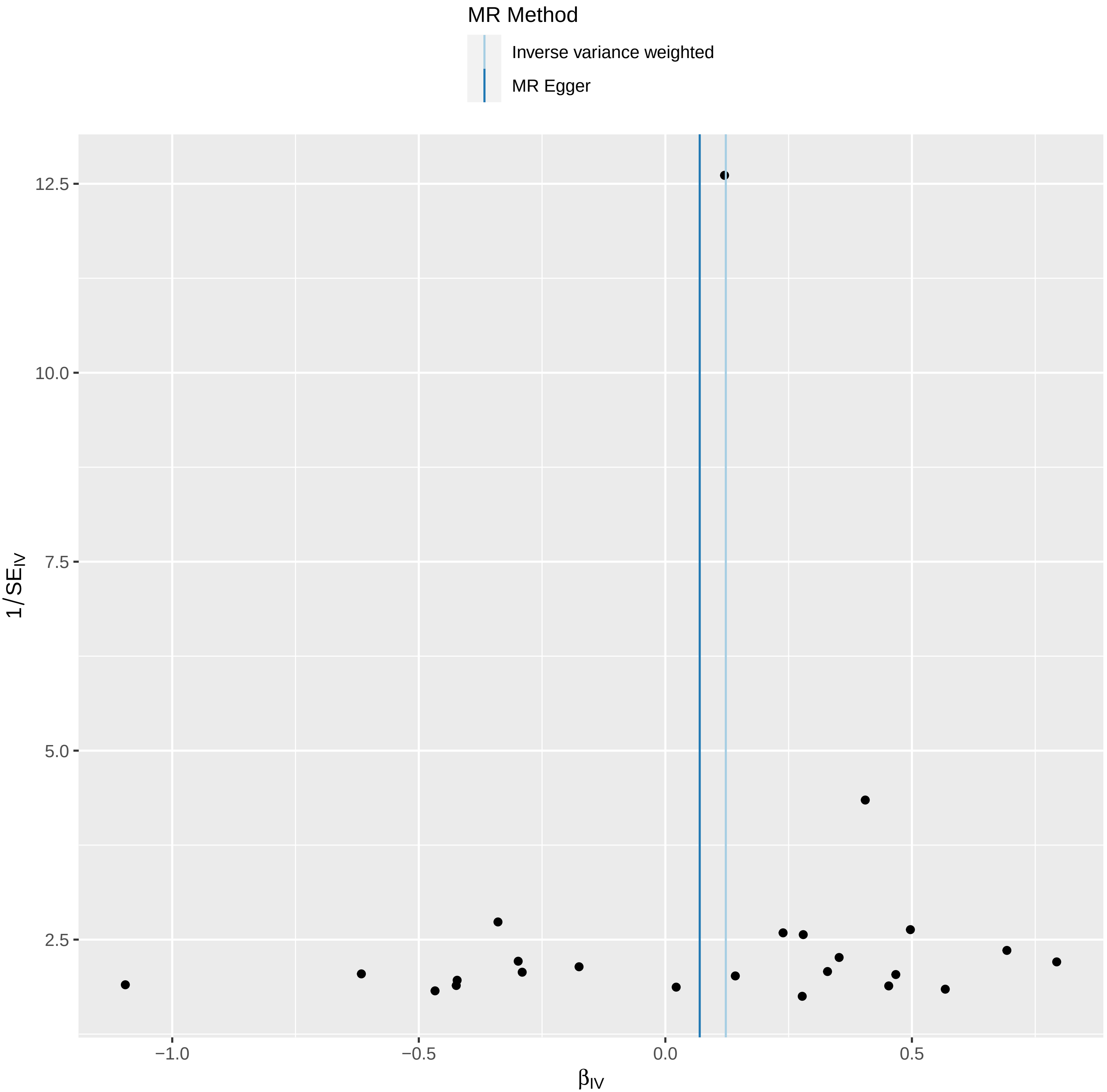
MR Method
Inverse variance weighted
MR Egger
12.5
10.0
7.5
5.0
2.5
1/SE_IV
−1.0
−0.5
0.0
0.5
β_IV

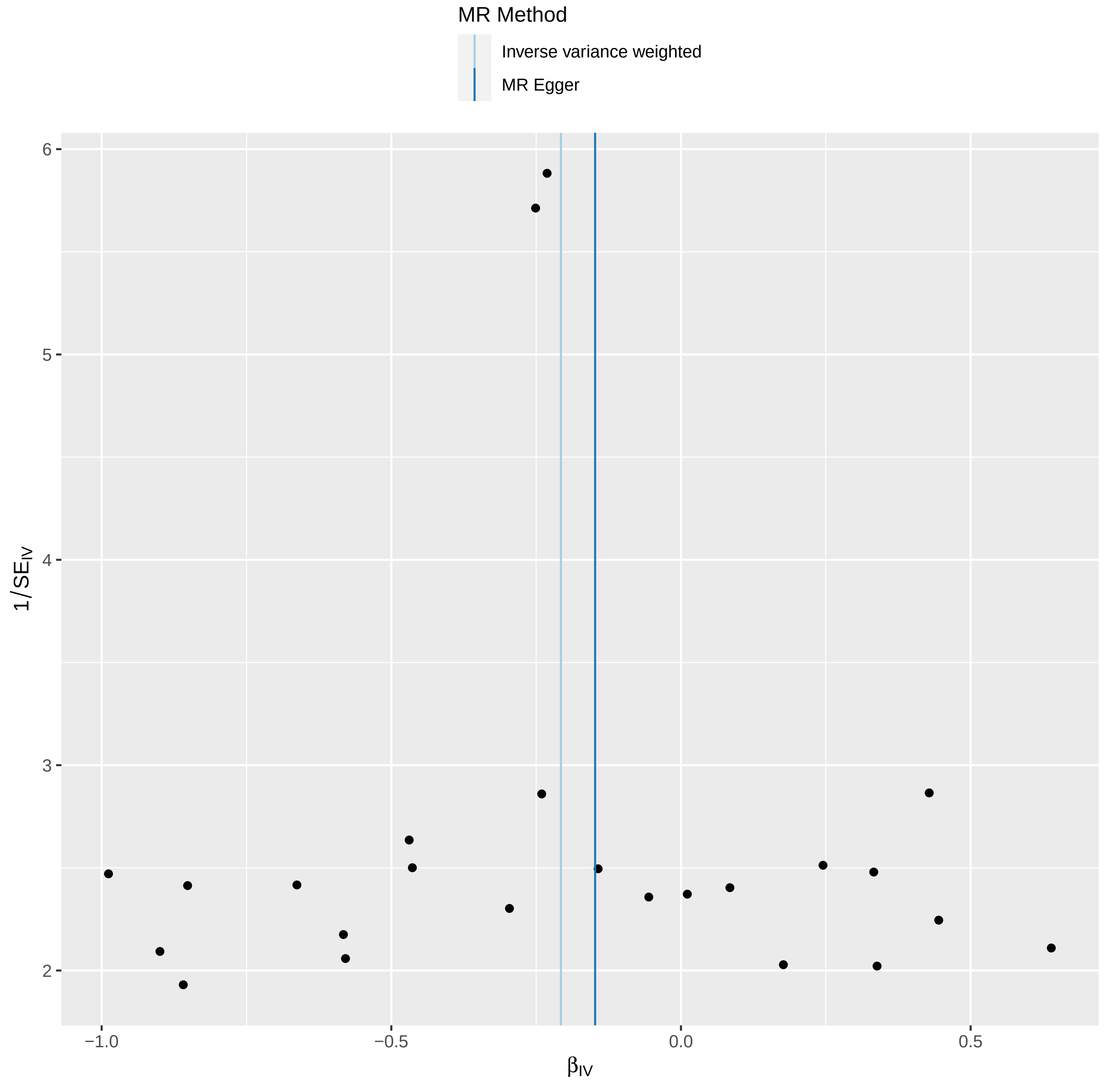
MR Method
Inverse variance weighted
MR Egger
6
5
4
3
2
1/SE_IV
−1.0
−0.5
0.0
0.5
β_IV

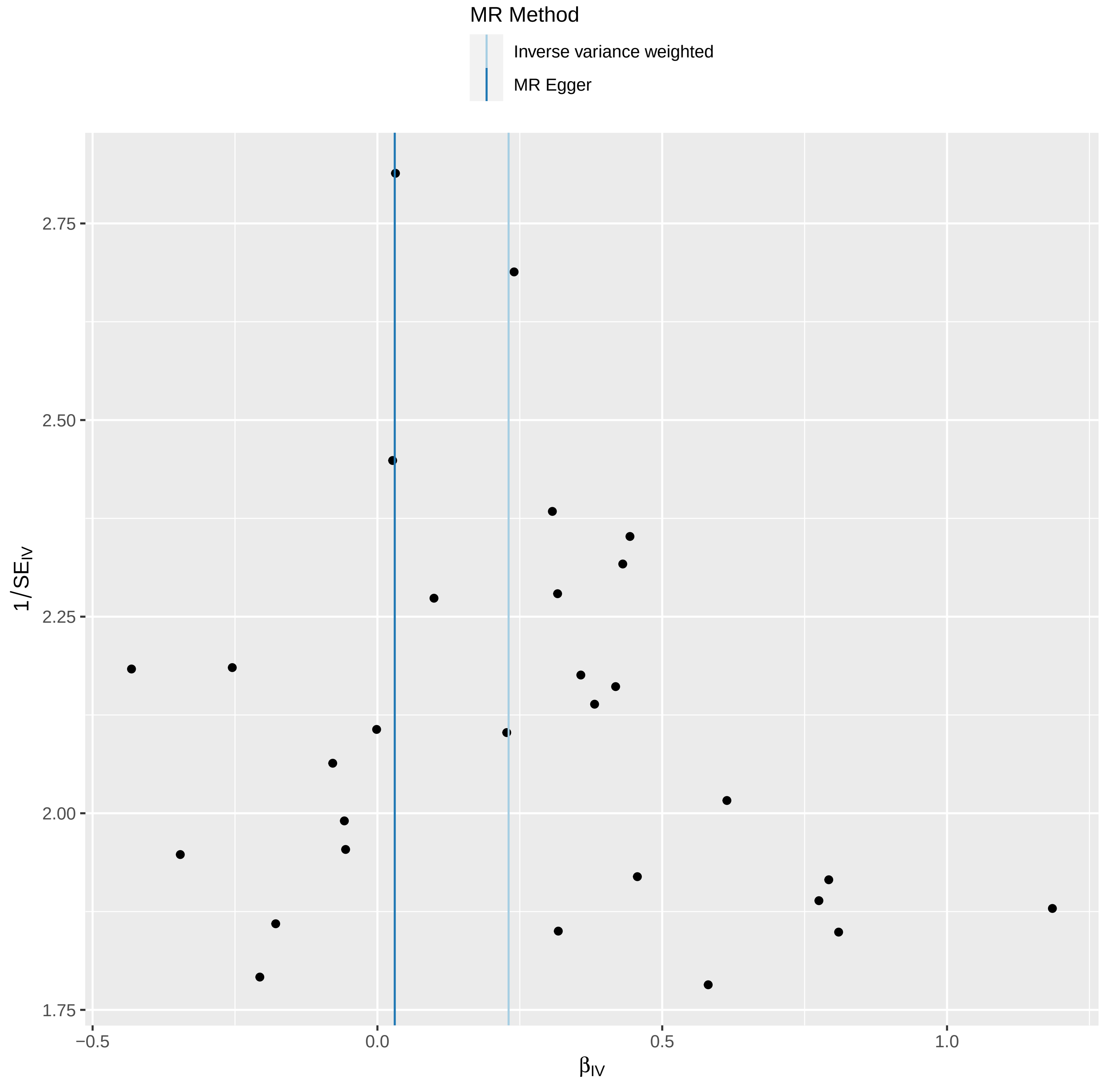
MR Method
Inverse variance weighted
MR Egger
2.75
2.50
2.25
2.00
1.75
1/SE_IV
-0.5
0.0
0.5
1.0
β_IV

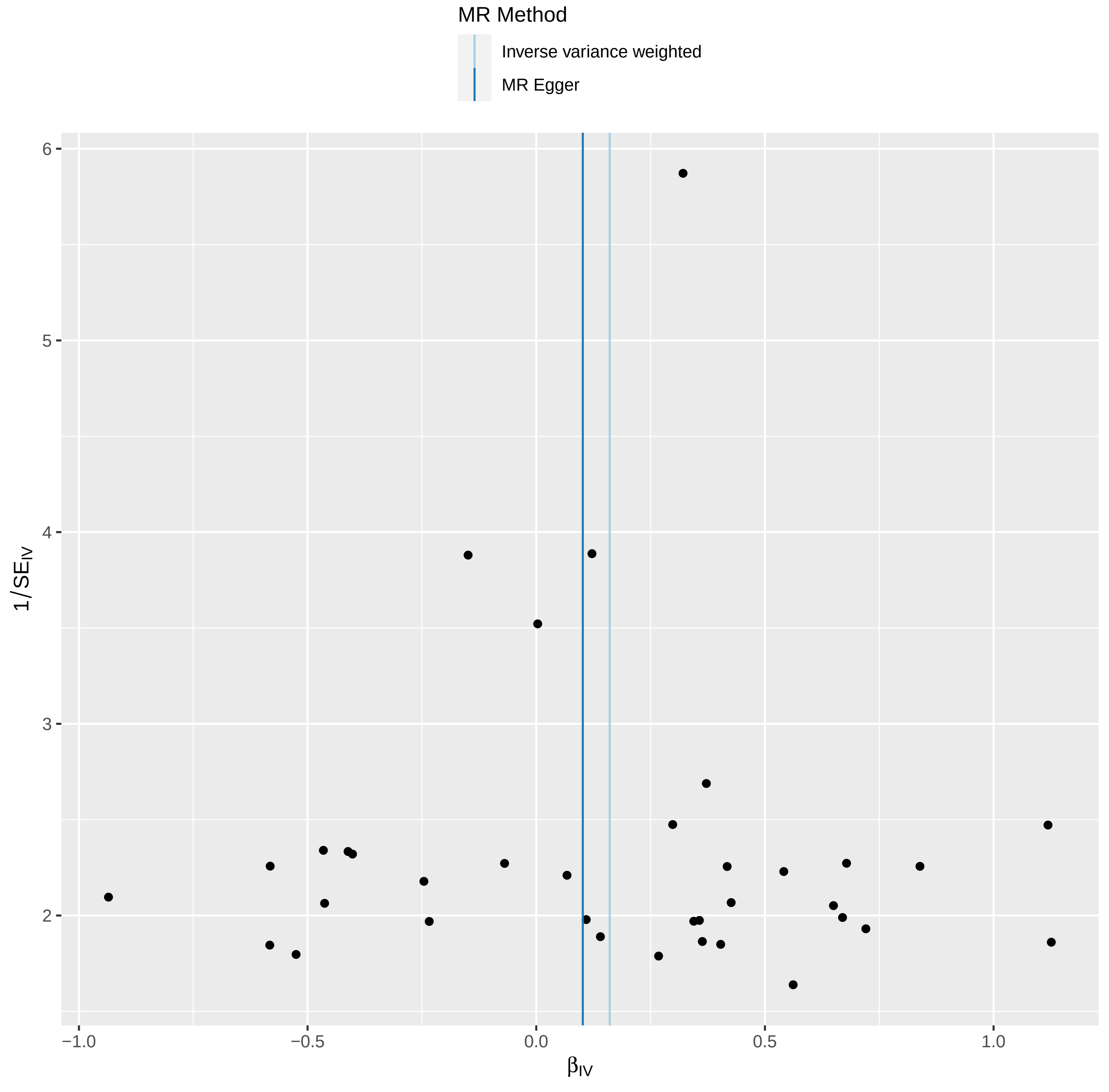

MR Method
Inverse variance weighted
MR Egger
6
5
4
3
2
1/SE$_{IV}$
−1.0
−0.5
0.0
0.5
1.0
β$_{IV}$

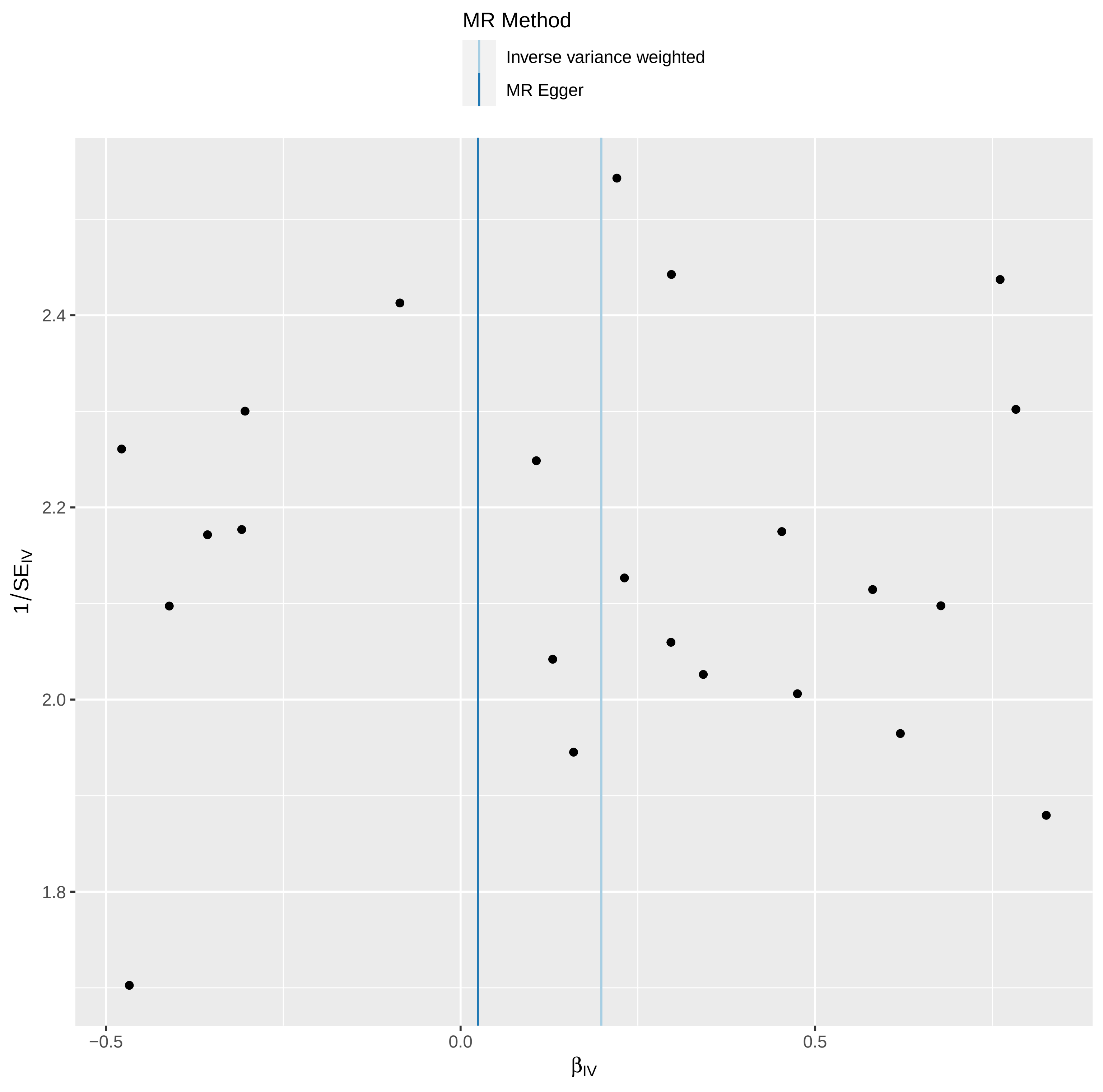

MR Method
Inverse variance weighted
MR Egger
2.4
2.2
2.0
1.8
1/SE$_{IV}$
−0.5
0.0
0.5
β$_{IV}$

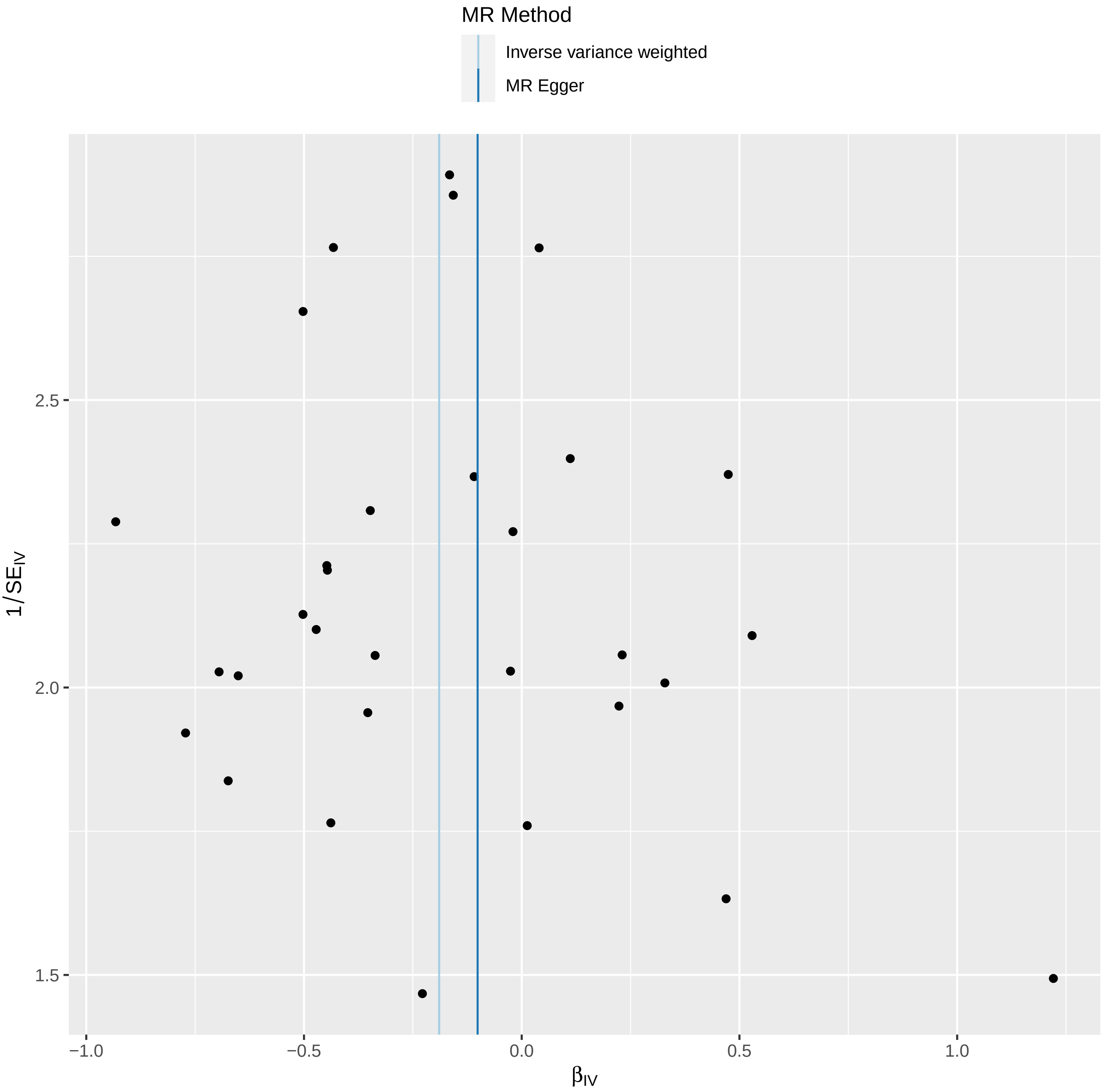

MR Method
Inverse variance weighted
MR Egger
2.5
2.0
1.5
1/SE$_{IV}$
−1.0
−0.5
0.0
0.5
1.0
β$_{IV}$

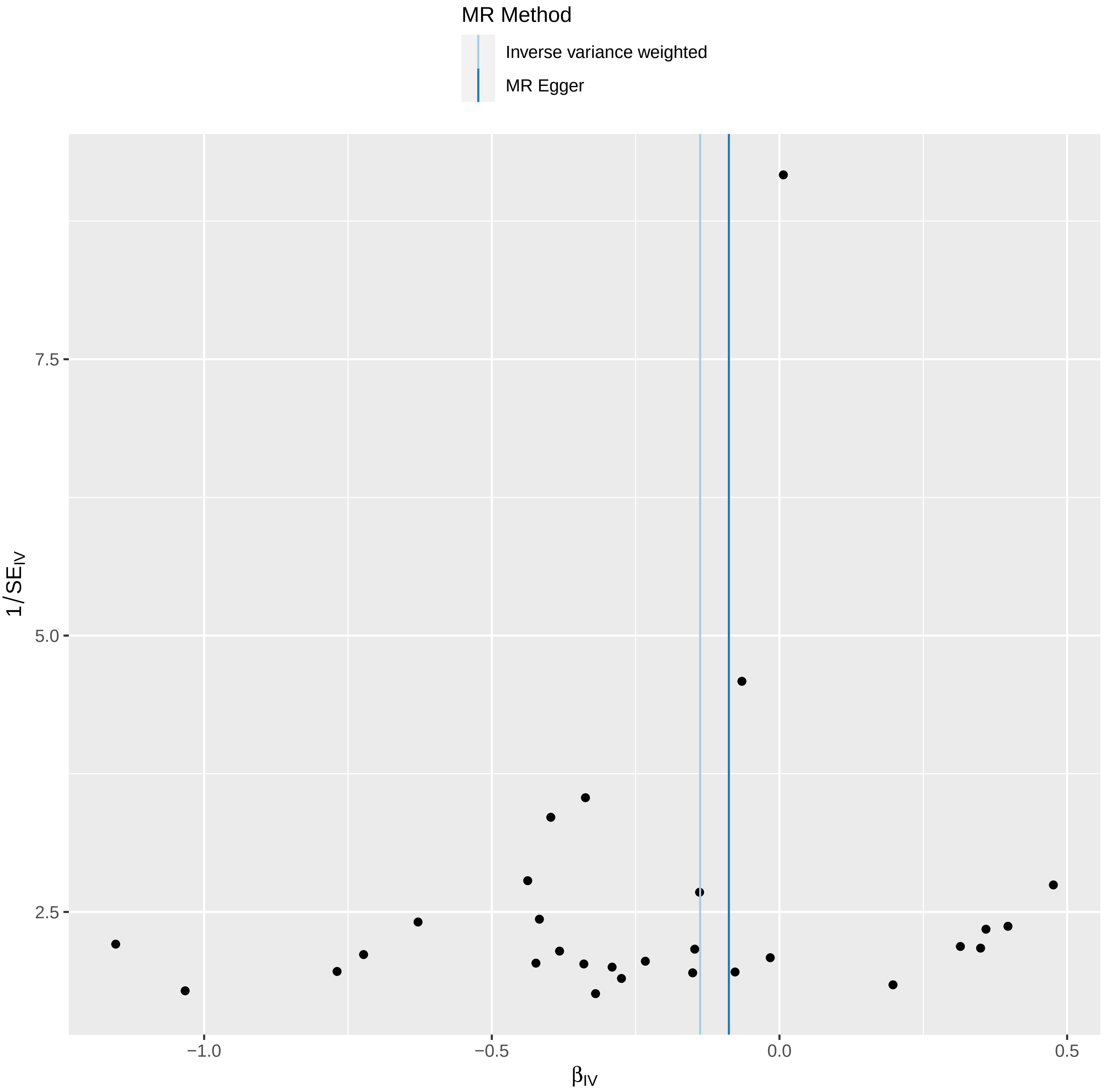

MR Method
Inverse variance weighted
MR Egger
7.5
5.0
2.5
1/SE_IV
−1.0
−0.5
0.0
0.5
β_IV

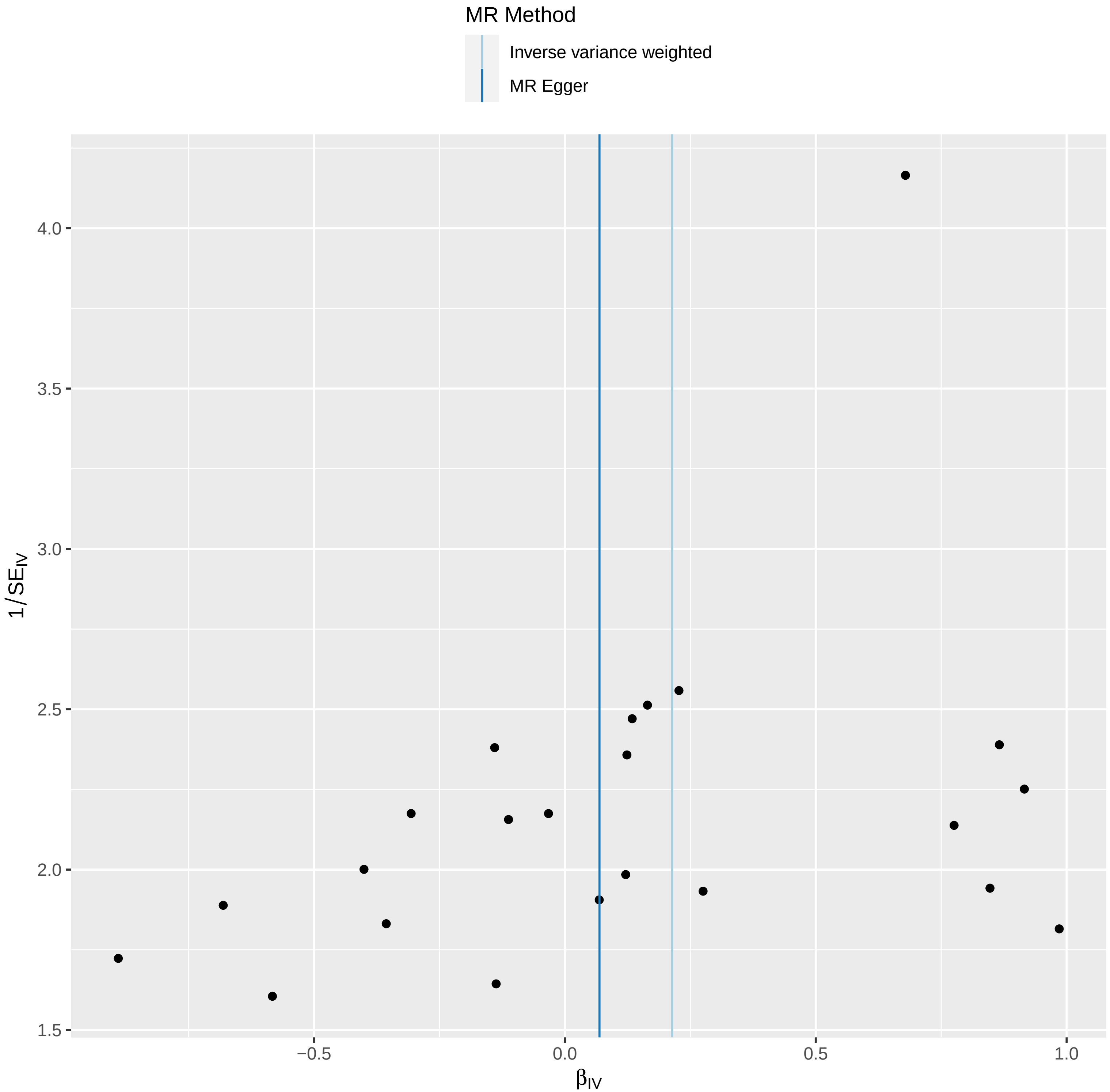

MR Method
Inverse variance weighted
MR Egger
4.0
3.5
3.0
2.5
2.0
1.5
1/SE_IV
−0.5
0.0
0.5
1.0
β_IV

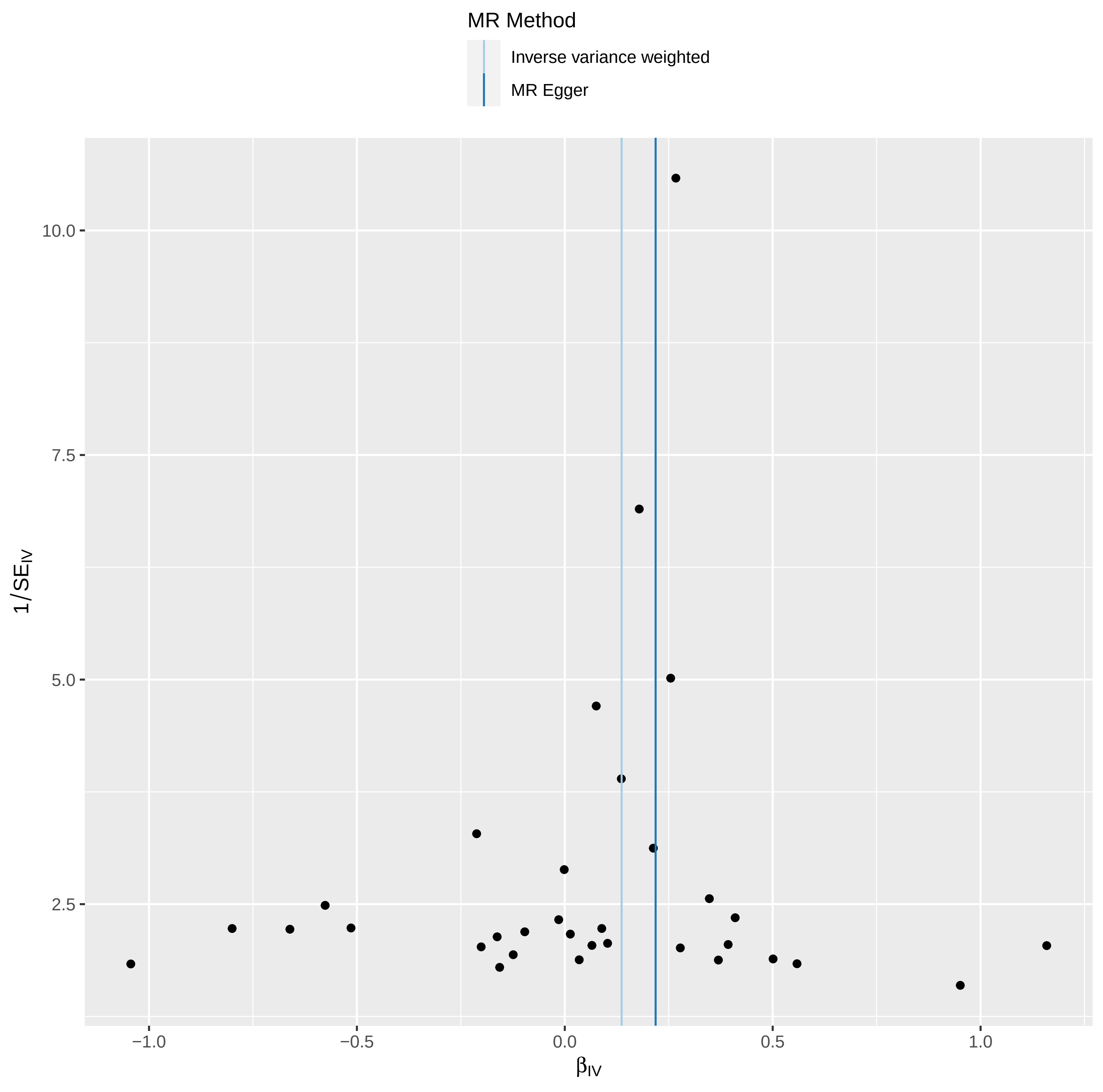

MR Method
Inverse variance weighted
MR Egger
10.0
7.5
5.0
2.5
1/SE$_{IV}$
−1.0
−0.5
0.0
0.5
1.0
β$_{IV}$

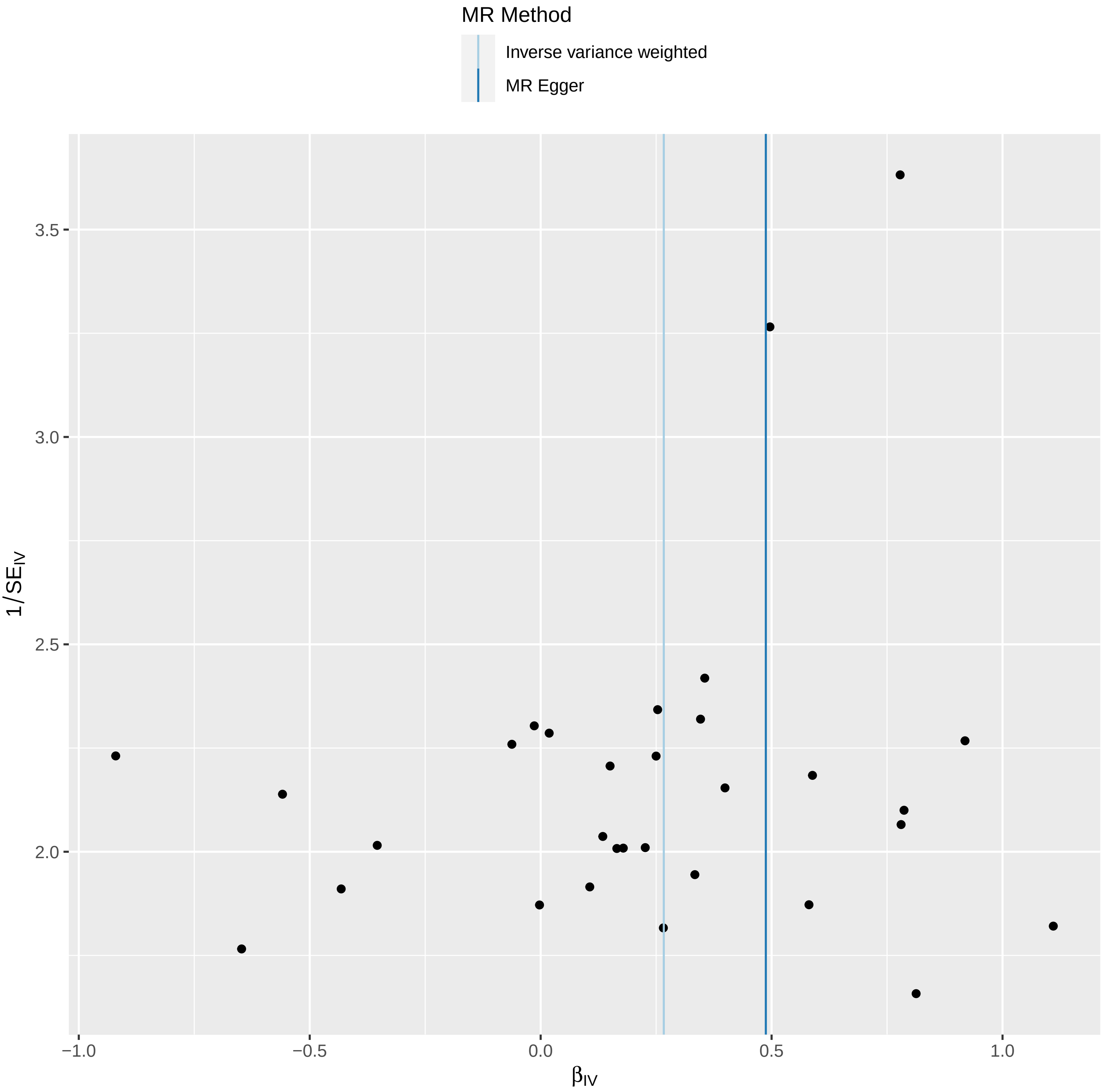

MR Method
Inverse variance weighted
MR Egger
3.5
3.0
2.5
2.0
1/SE$_{IV}$
−1.0
−0.5
0.0
0.5
1.0
β$_{IV}$

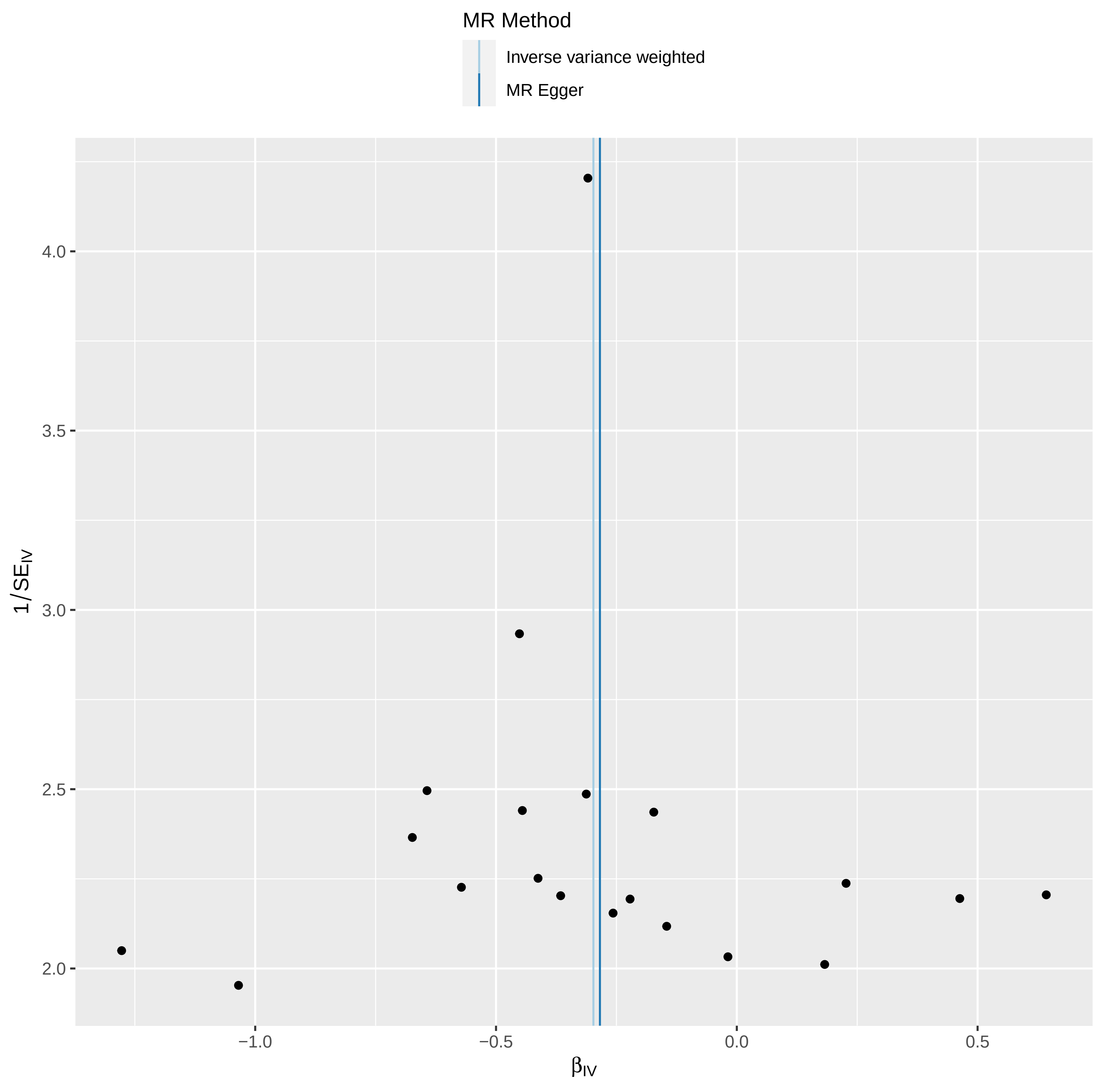
MR Method
Inverse variance weighted
MR Egger
4.0
3.5
3.0
2.5
2.0
1/SE_IV
−1.0
−0.5
0.0
0.5
β_IV

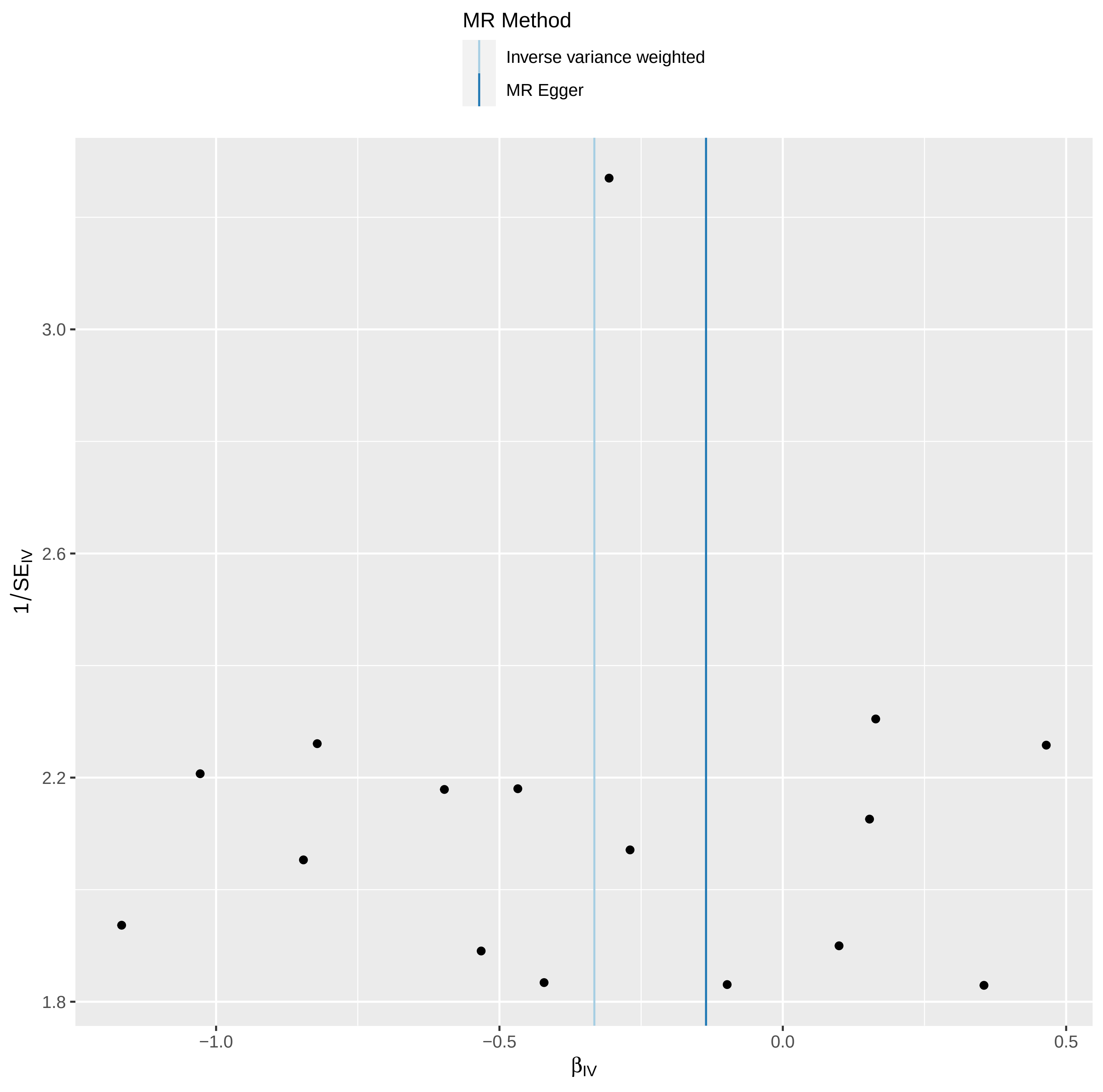
MR Method
Inverse variance weighted
MR Egger
1/SE$_{IV}$
3.0
2.6
2.2
1.8
−1.0
−0.5
0.0
0.5
β_{IV}

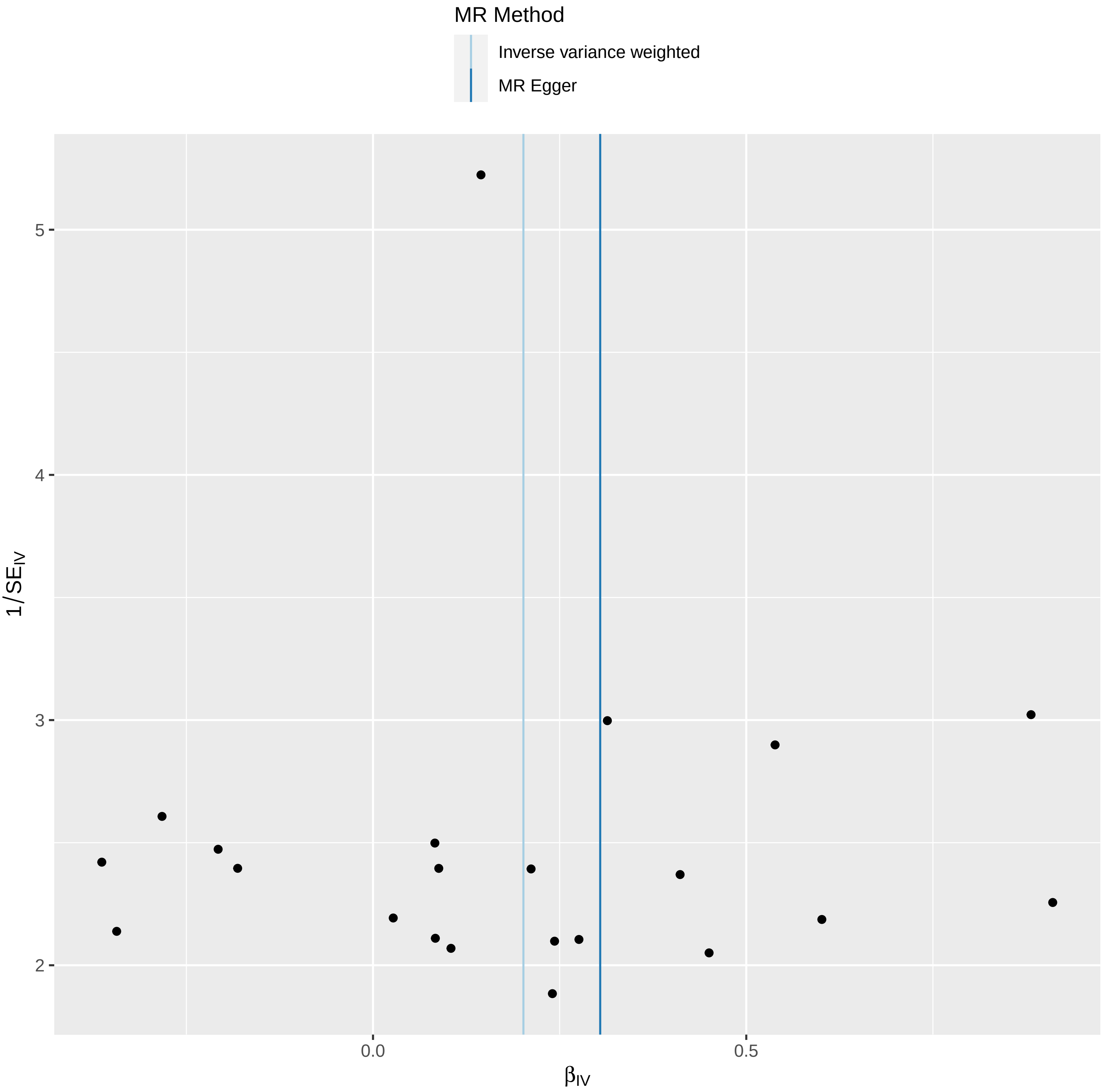
MR Method
Inverse variance weighted
MR Egger
5
4
3
2
1/SE_IV
0.0
0.5
β_IV

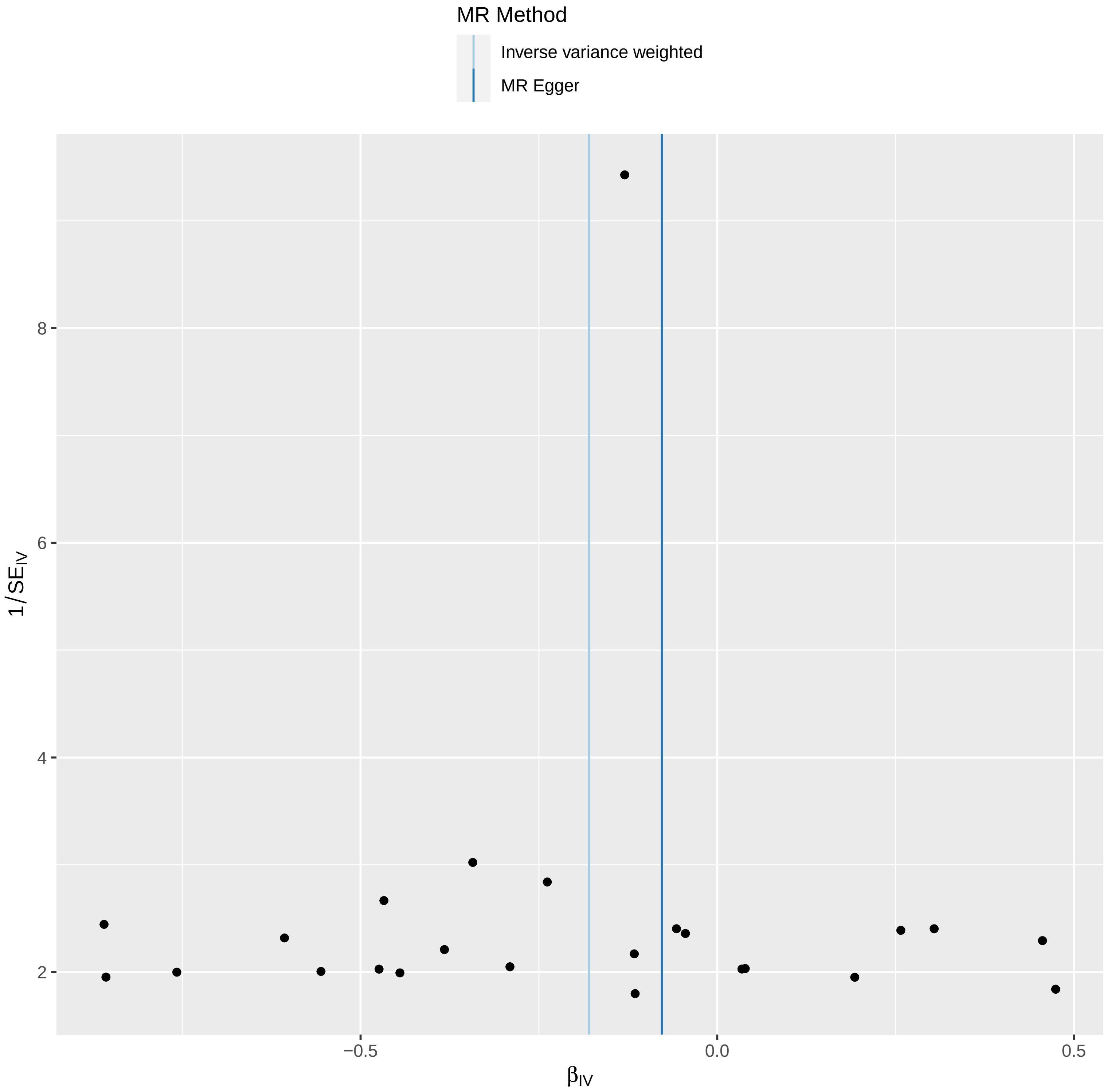
MR Method
Inverse variance weighted
MR Egger
1/SE$_{IV}$
8
6
4
2
−0.5
0.0
0.5
β$_{IV}$

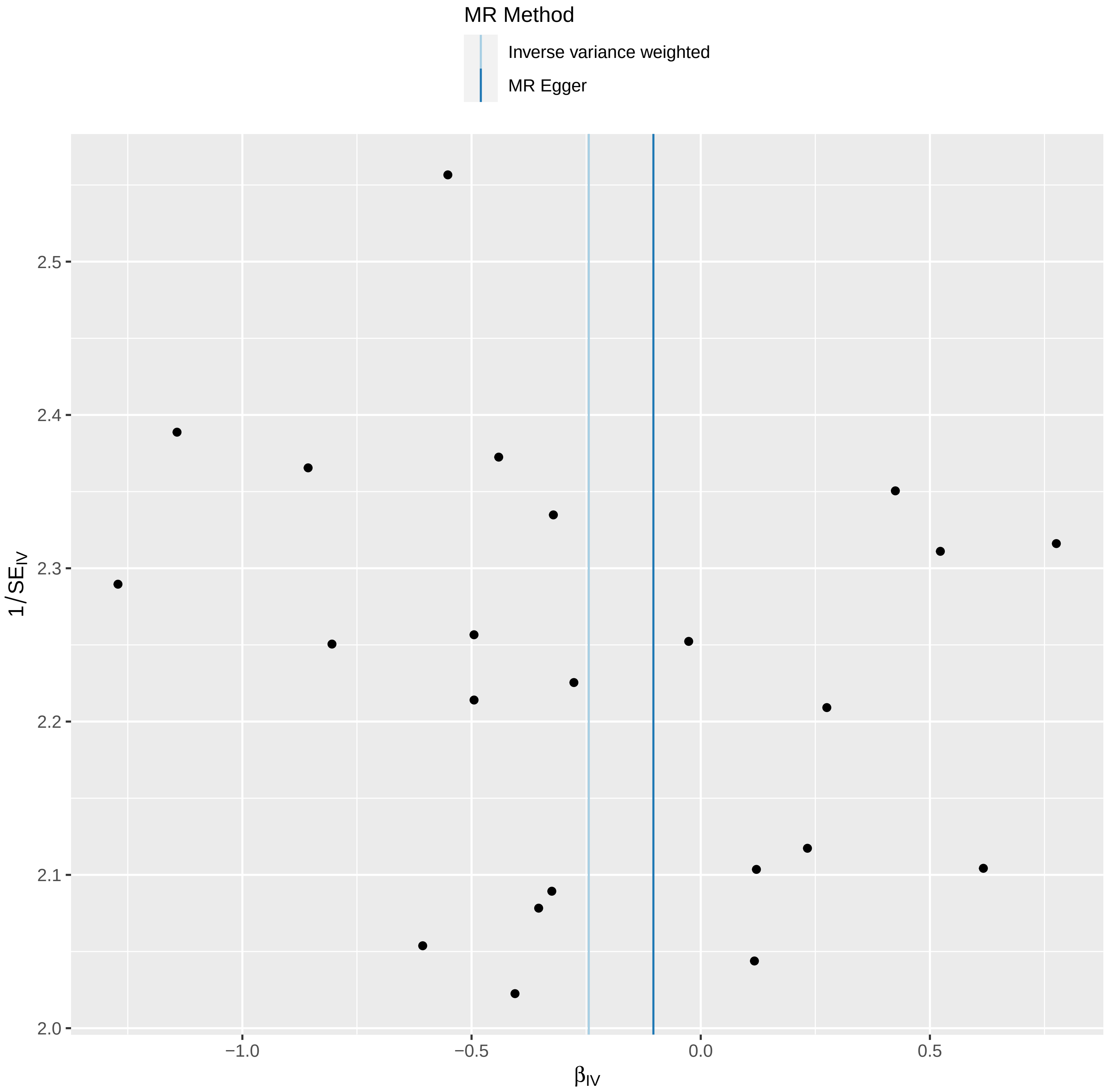

MR Method
Inverse variance weighted
MR Egger
2.5
2.4
2.3
2.2
2.1
2.0
1/SE_IV
−1.0
−0.5
0.0
0.5
β_IV

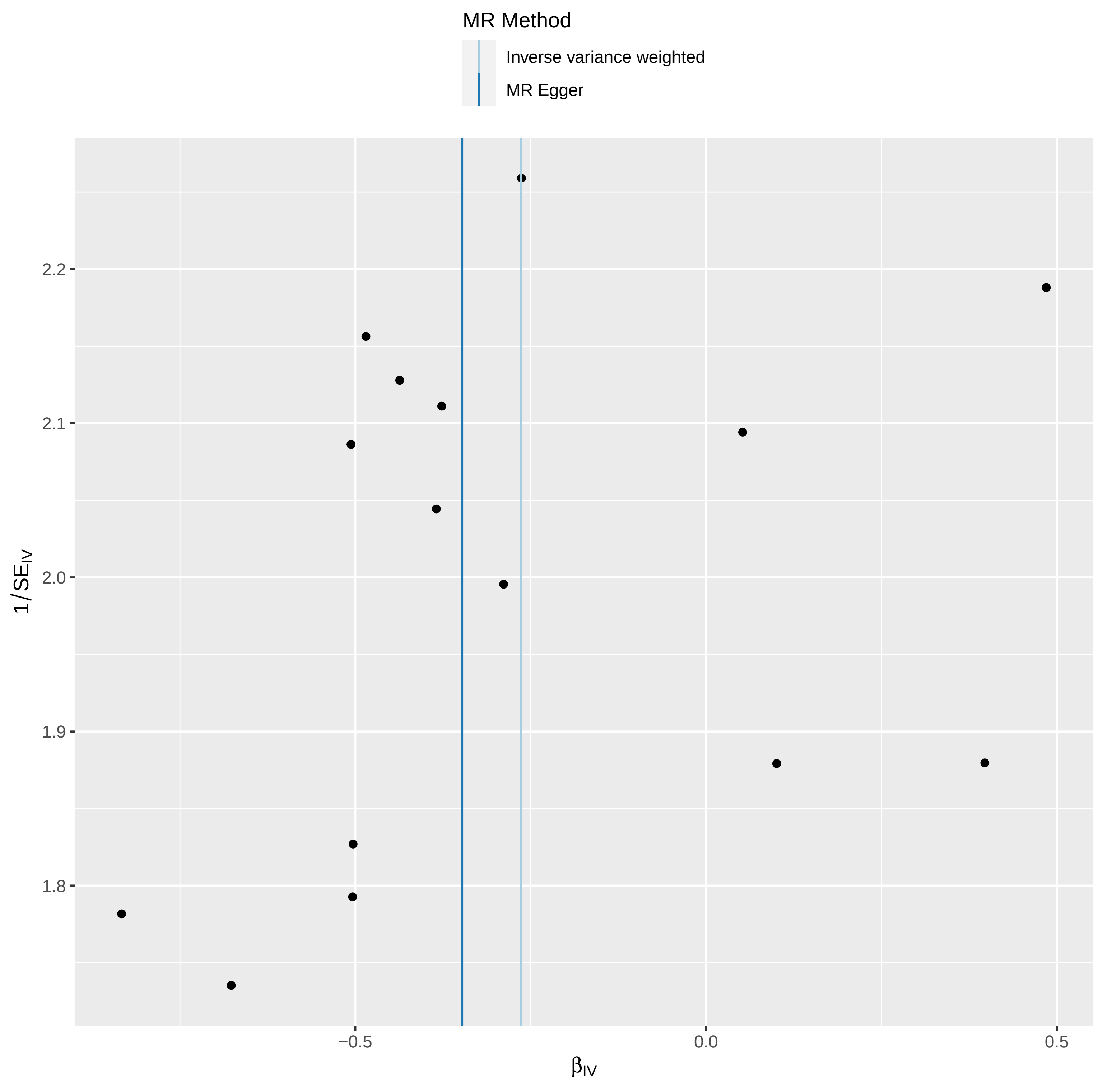

MR Method
Inverse variance weighted
MR Egger
2.2
2.1
2.0
1.9
1.8
$1/SE_{IV}$
−0.5
0.0
0.5
β_{IV}

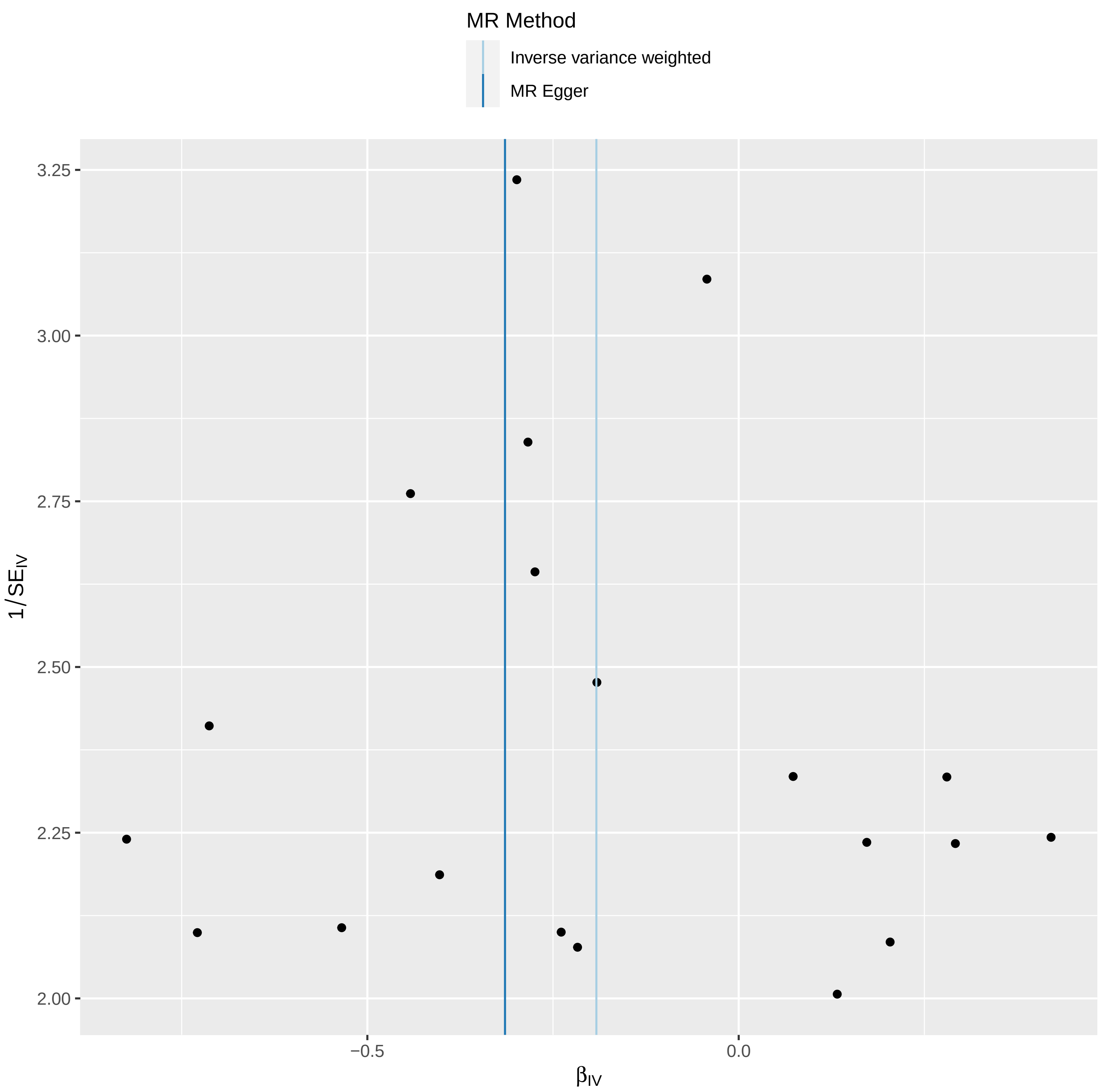
MR Method
Inverse variance weighted
MR Egger
3.25
3.00
2.75
2.50
2.25
2.00
1/SE$_{IV}$
−0.5
0.0
β$_{IV}$

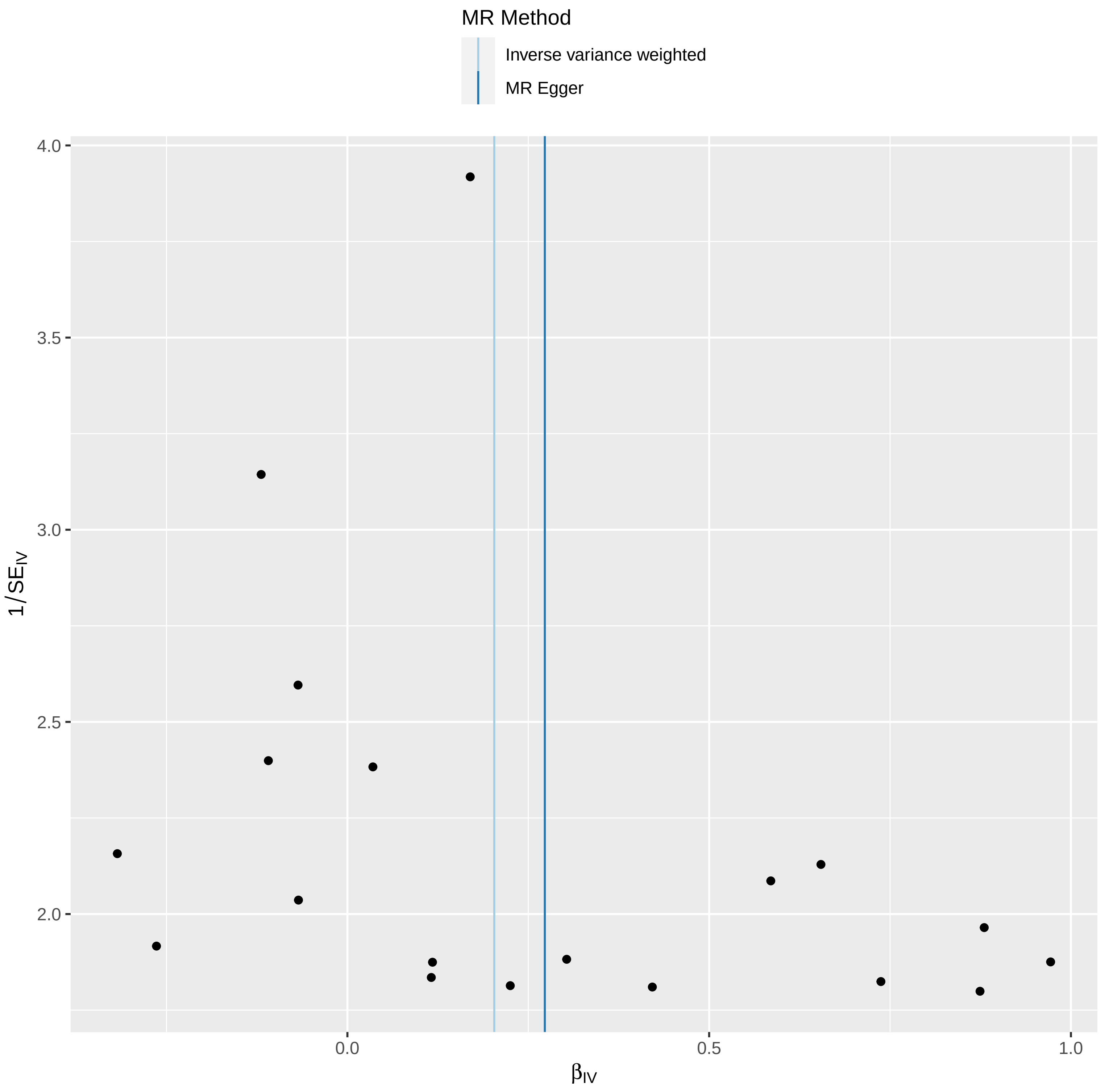

MR Method
Inverse variance weighted
MR Egger
4.0
3.5
3.0
2.5
2.0
0.0
0.5
1.0
$1/SE_{IV}$
β_{IV}

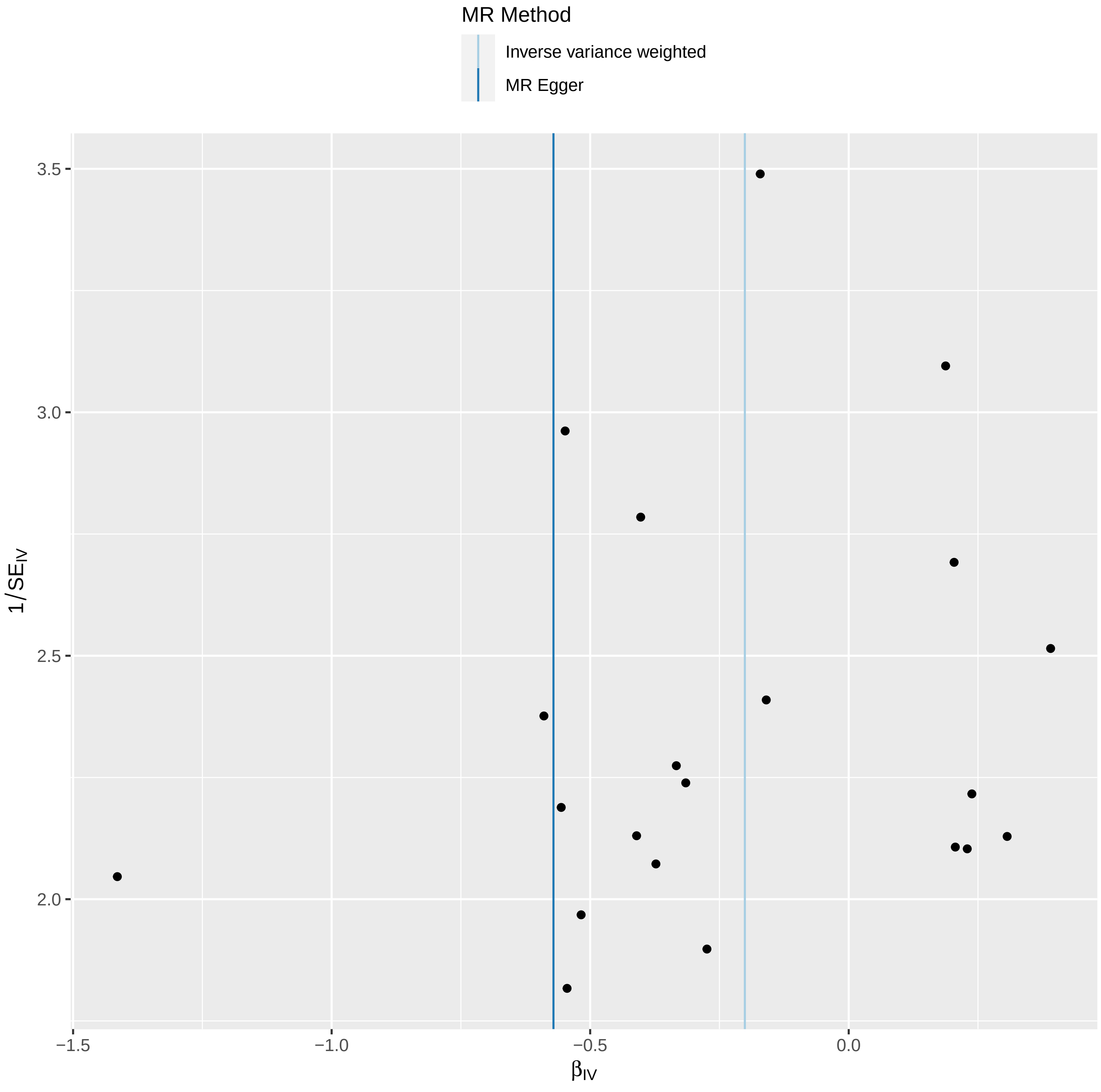

MR Method
Inverse variance weighted
MR Egger
3.5
3.0
2.5
2.0
$1/SE_{IV}$
−1.5
−1.0
−0.5
0.0
β_{IV}

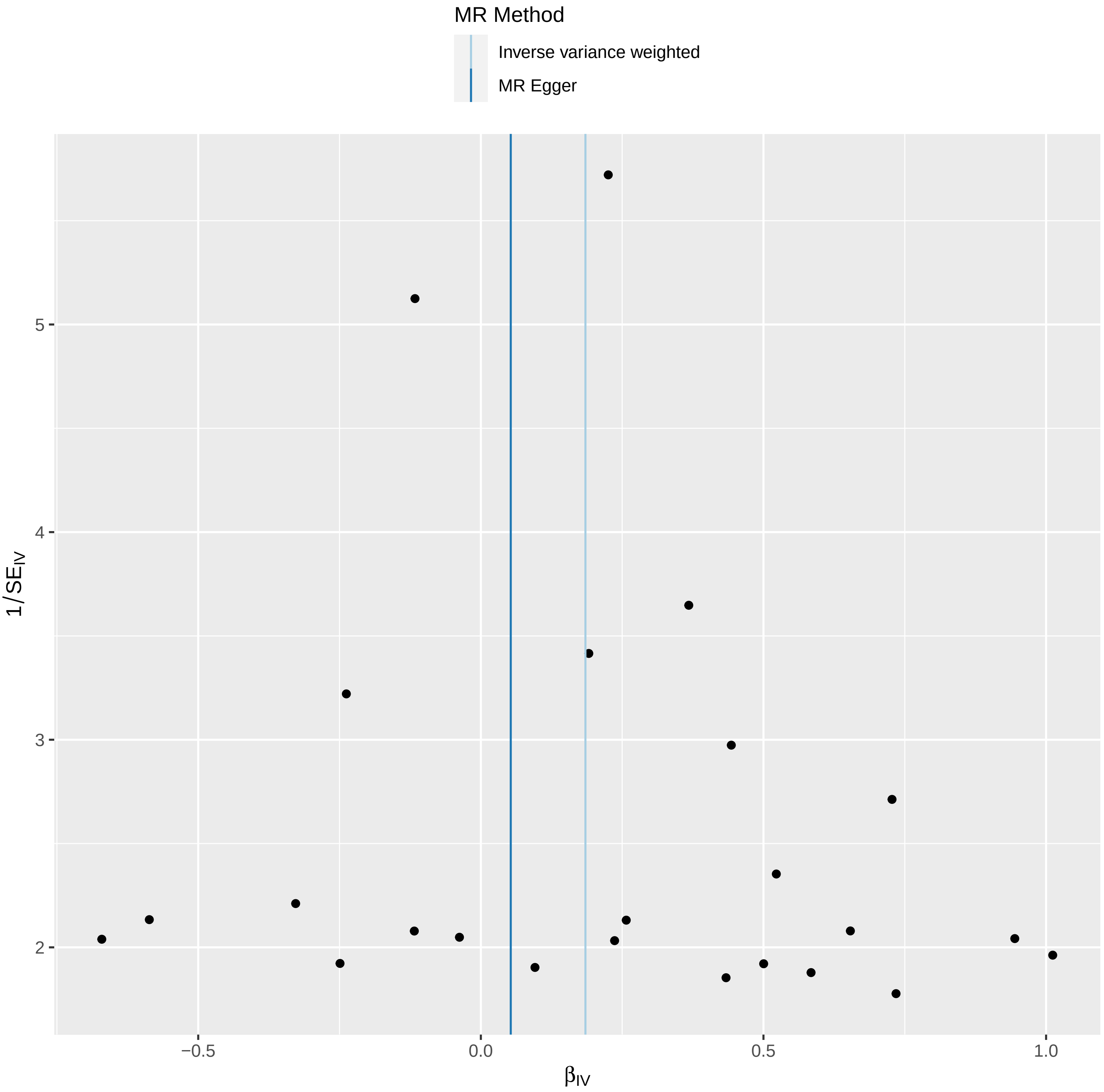

MR Method
Inverse variance weighted
MR Egger
5
4
3
2
1/SE_IV
−0.5
0.0
0.5
1.0
β_IV

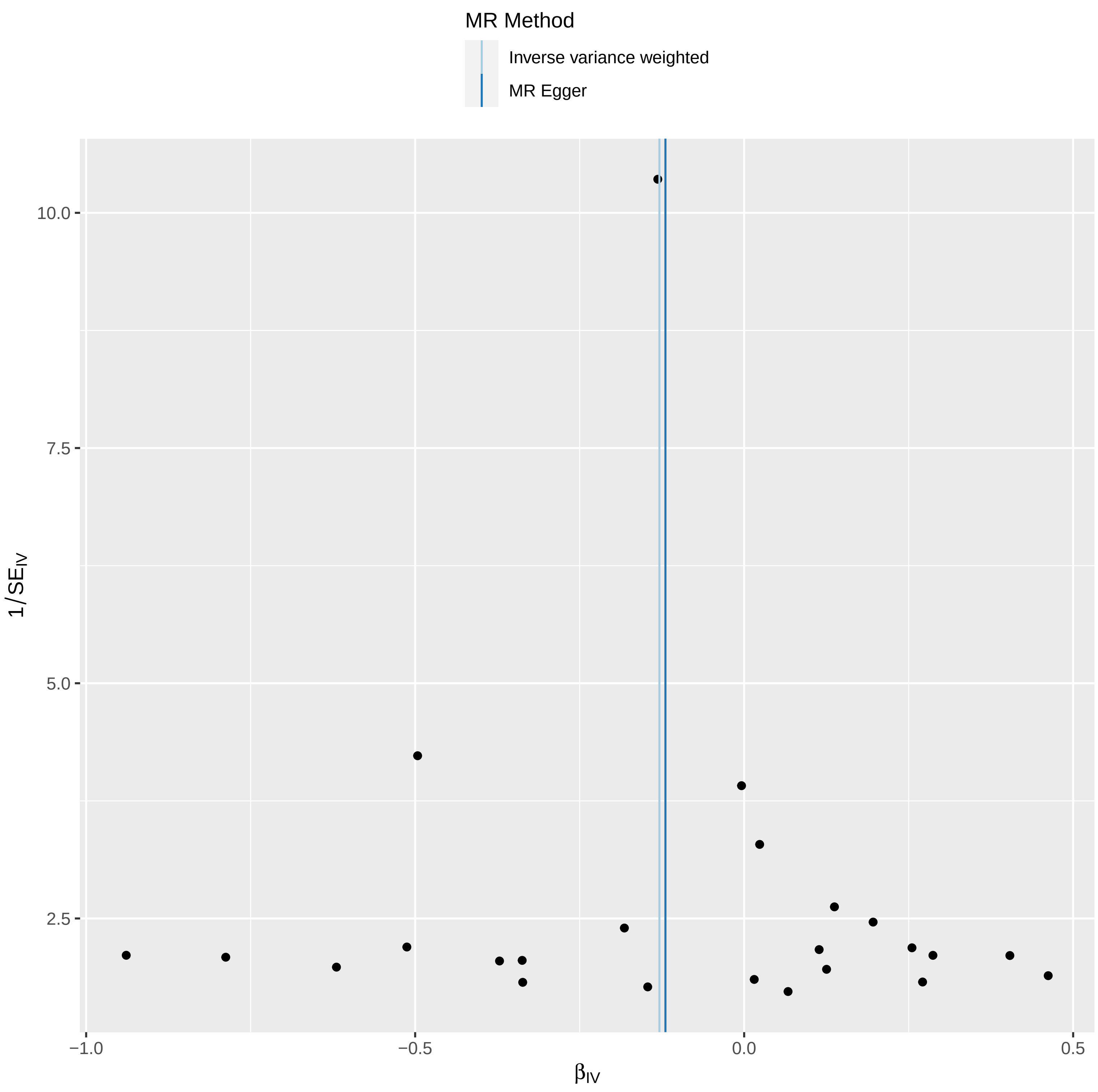

MR Method
Inverse variance weighted
MR Egger
10.0
7.5
5.0
2.5
1/SE$_{IV}$
−1.0
−0.5
0.0
0.5
β$_{IV}$

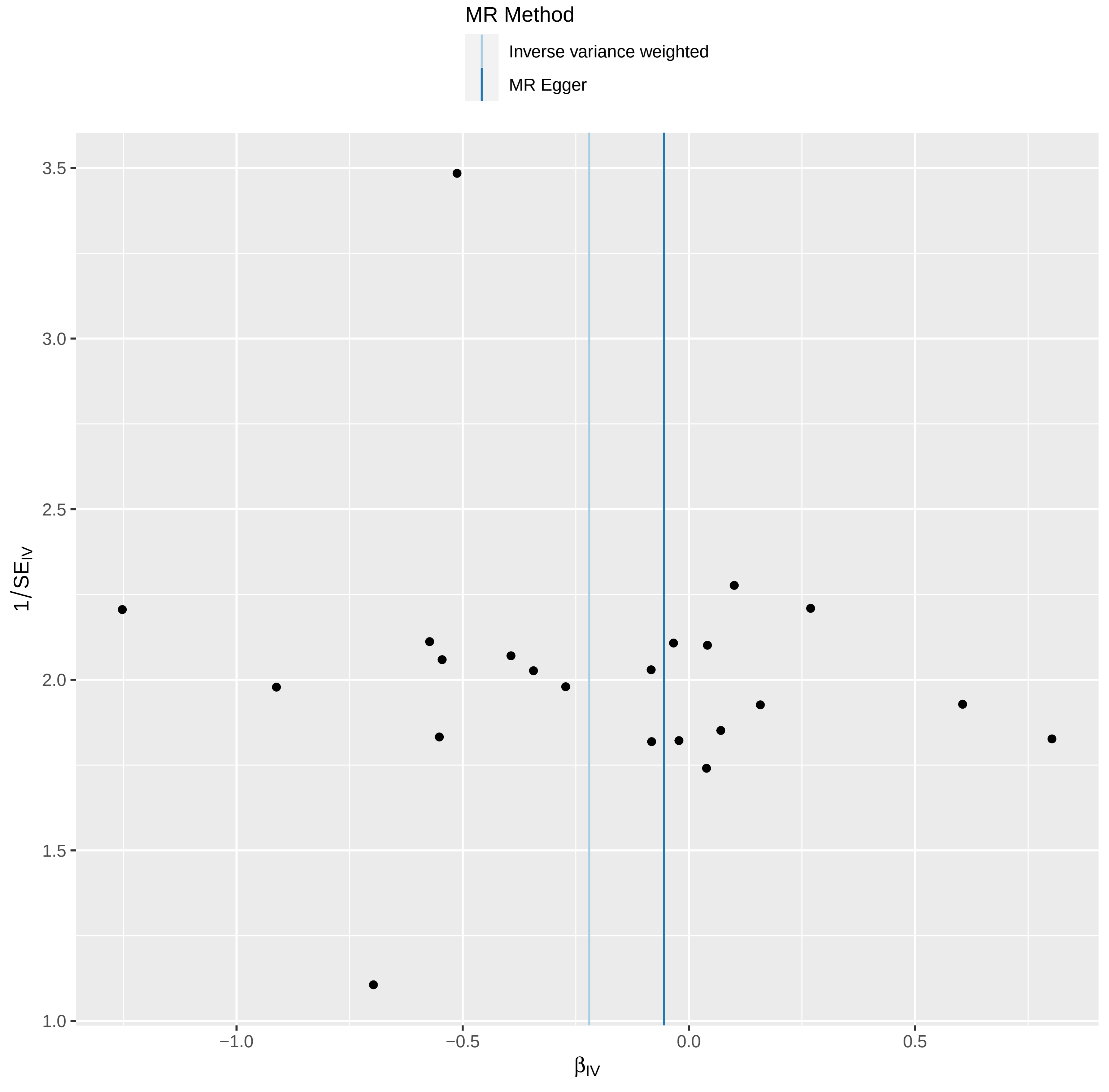
MR Method
Inverse variance weighted
MR Egger
3.5
3.0
2.5
2.0
1.5
1.0
1/SE_IV
−1.0
−0.5
0.0
0.5
β_IV

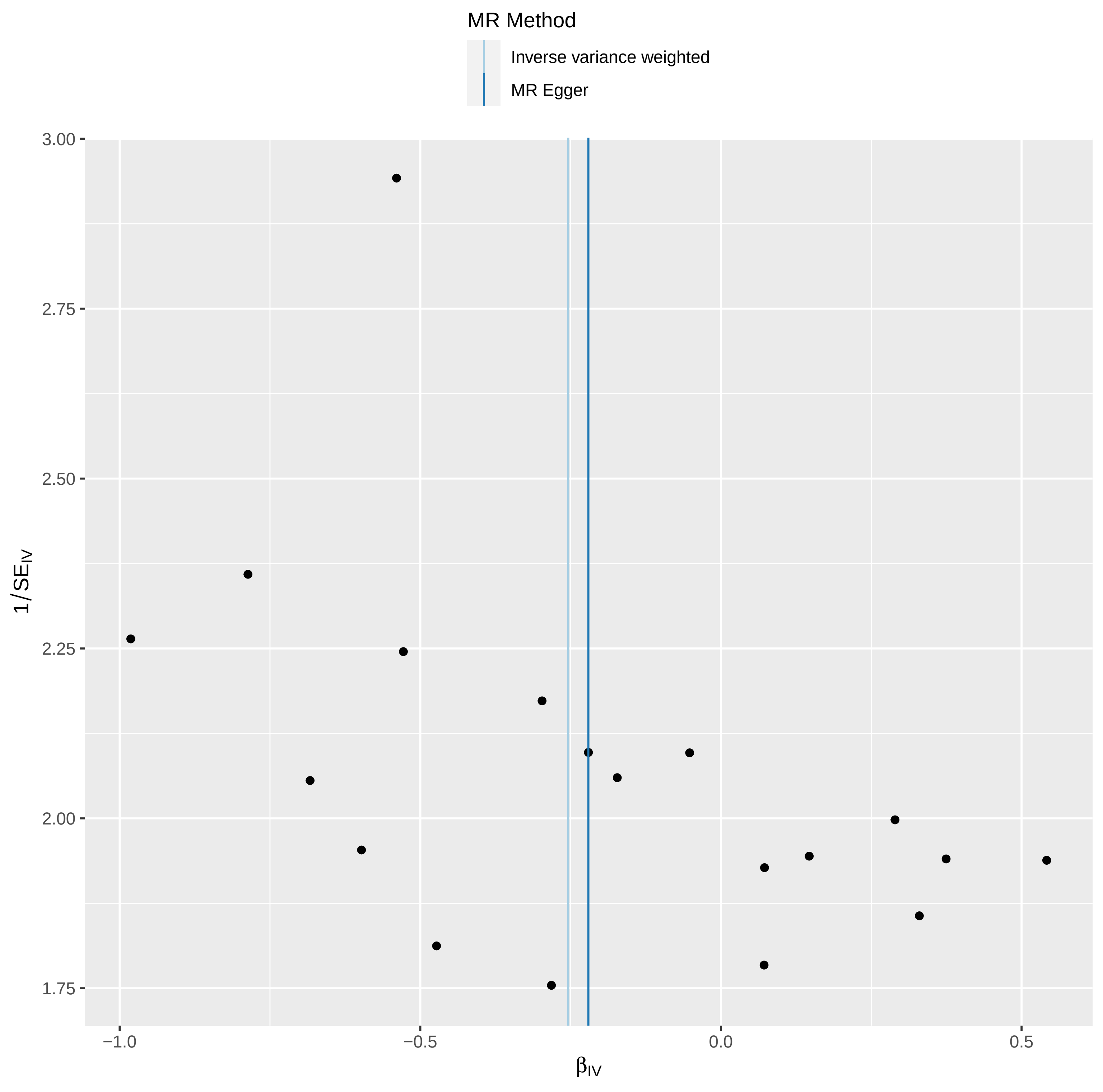

MR Method
Inverse variance weighted
MR Egger
3.00
2.75
2.50
2.25
2.00
1.75
$1/SE_{IV}$
−1.0
−0.5
0.0
0.5
β_{IV}

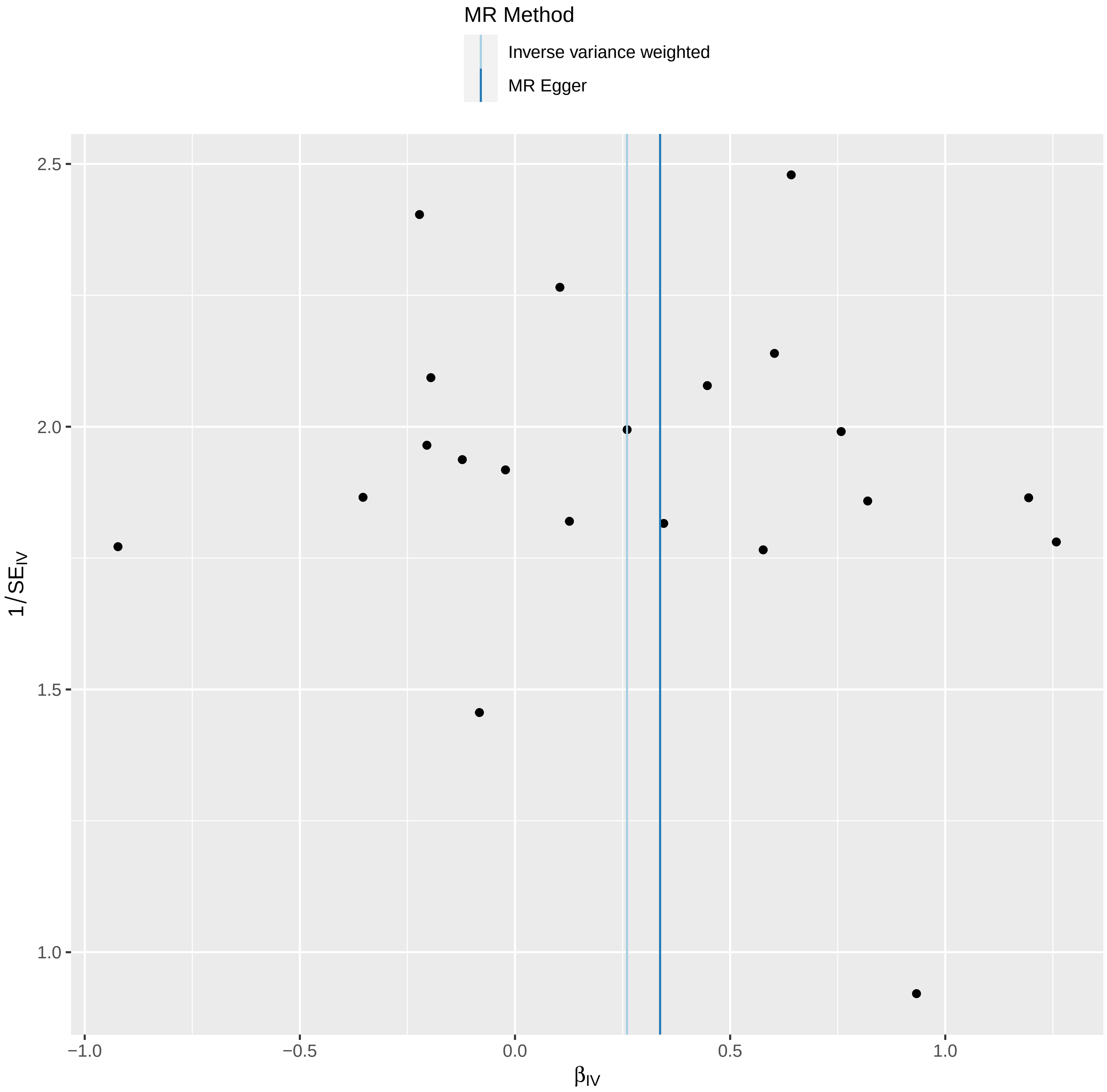

MR Method
Inverse variance weighted
MR Egger
2.5
2.0
1.5
1.0
1/SE_IV
−1.0
−0.5
0.0
0.5
1.0
β_IV

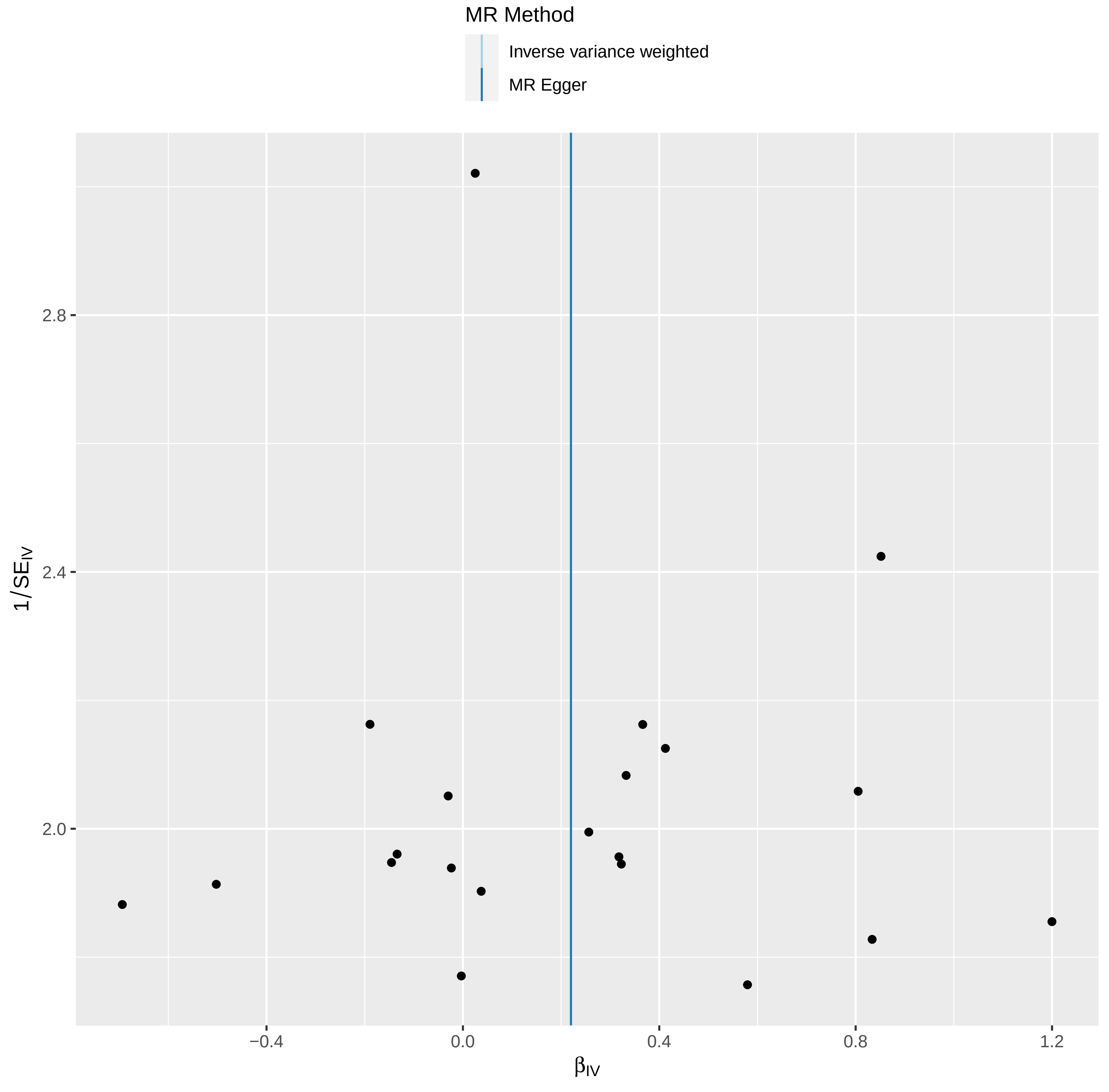
MR Method
Inverse variance weighted
MR Egger
1/SE$_{IV}$
2.8
2.4
2.0
−0.4
0.0
0.4
0.8
1.2
β$_{IV}$

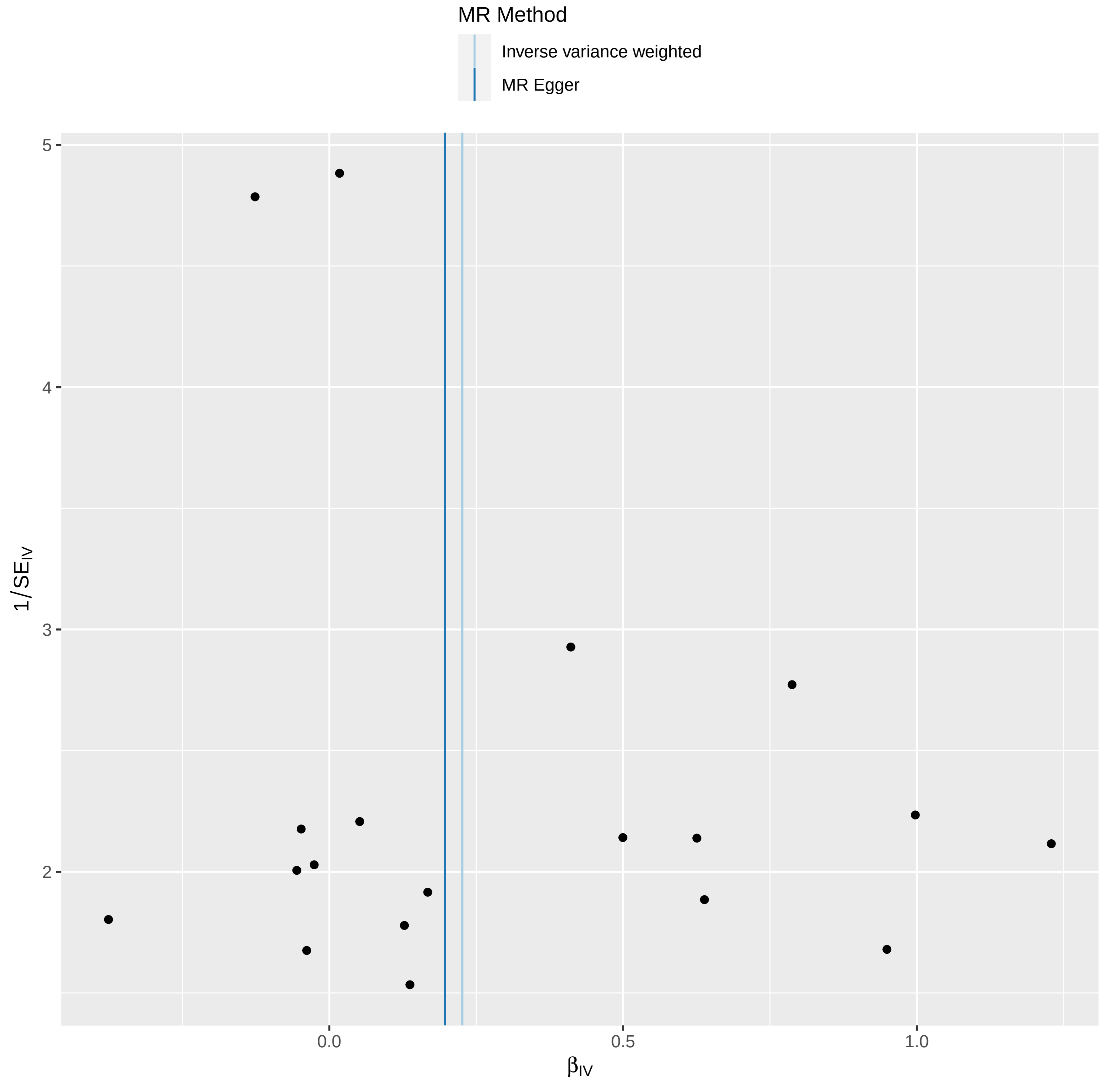
MR Method
Inverse variance weighted
MR Egger
5
4
3
2
1/SE_IV
0.0
0.5
1.0
β_IV

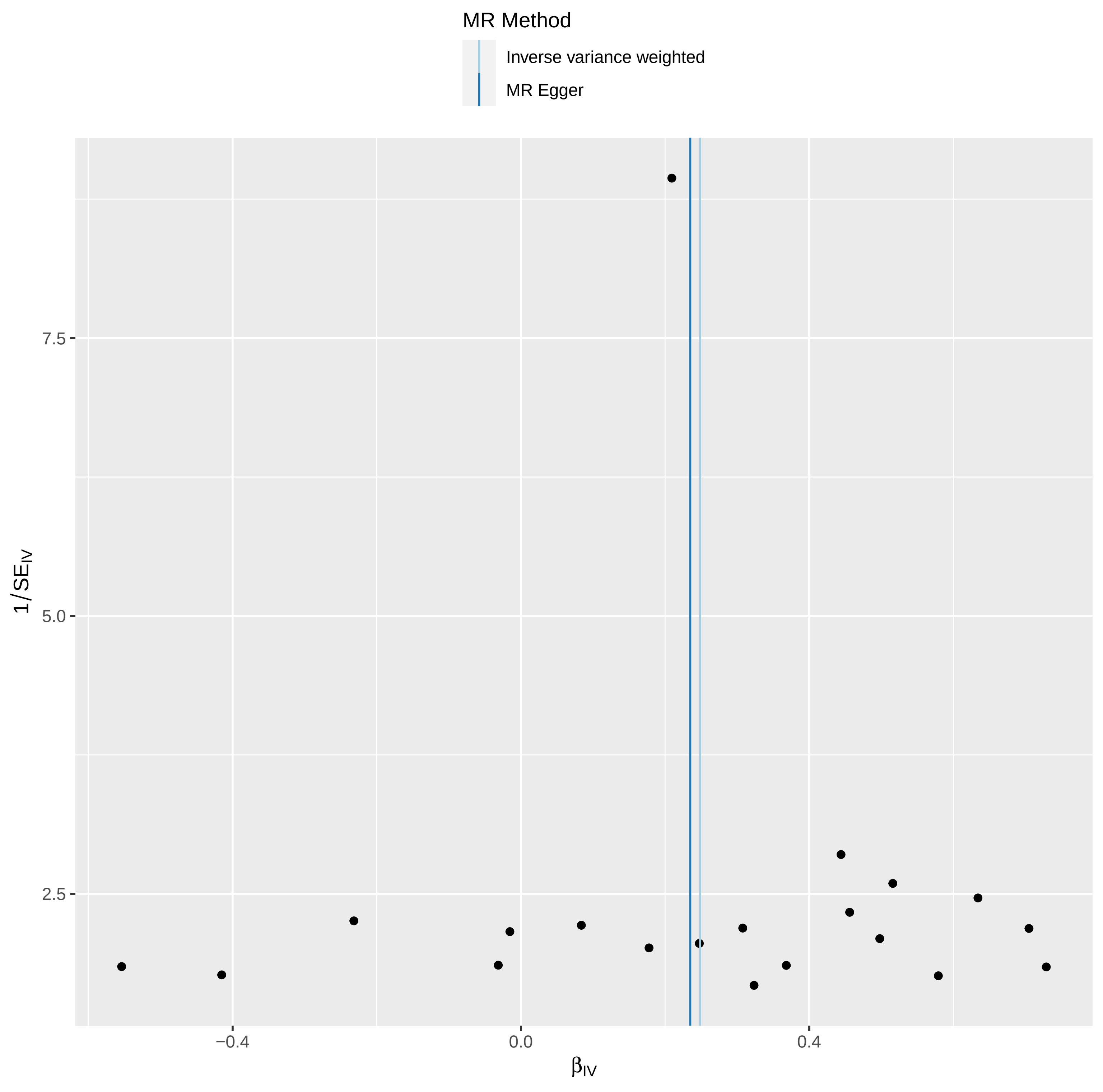

MR Method
Inverse variance weighted
MR Egger
7.5
5.0
2.5
−0.4
0.0
0.4
$1/SE_{IV}$
β_{IV}

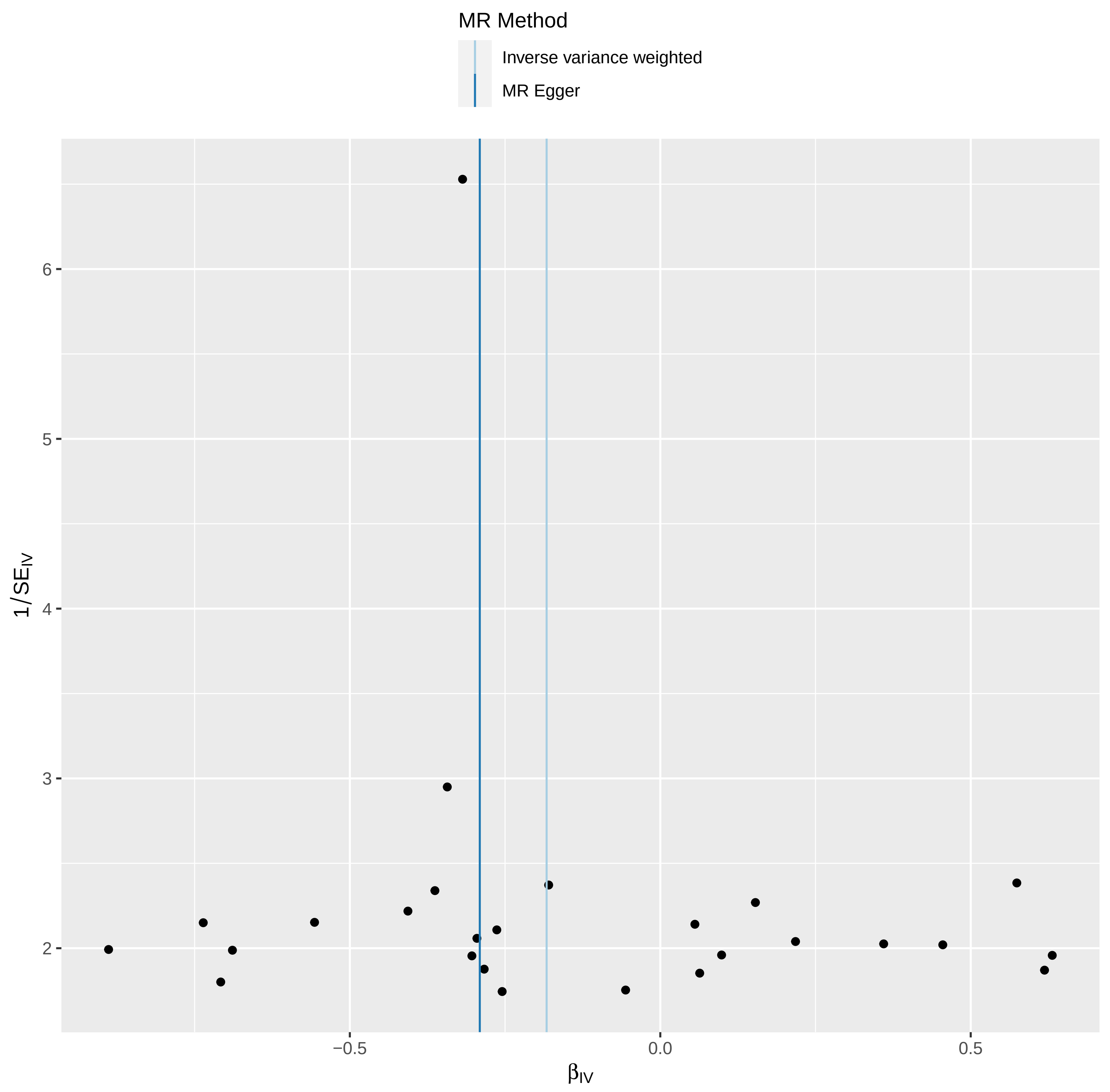

MR Method
Inverse variance weighted
MR Egger
6
5
4
3
2
−0.5
0.0
0.5
1/SE_IV
β_IV

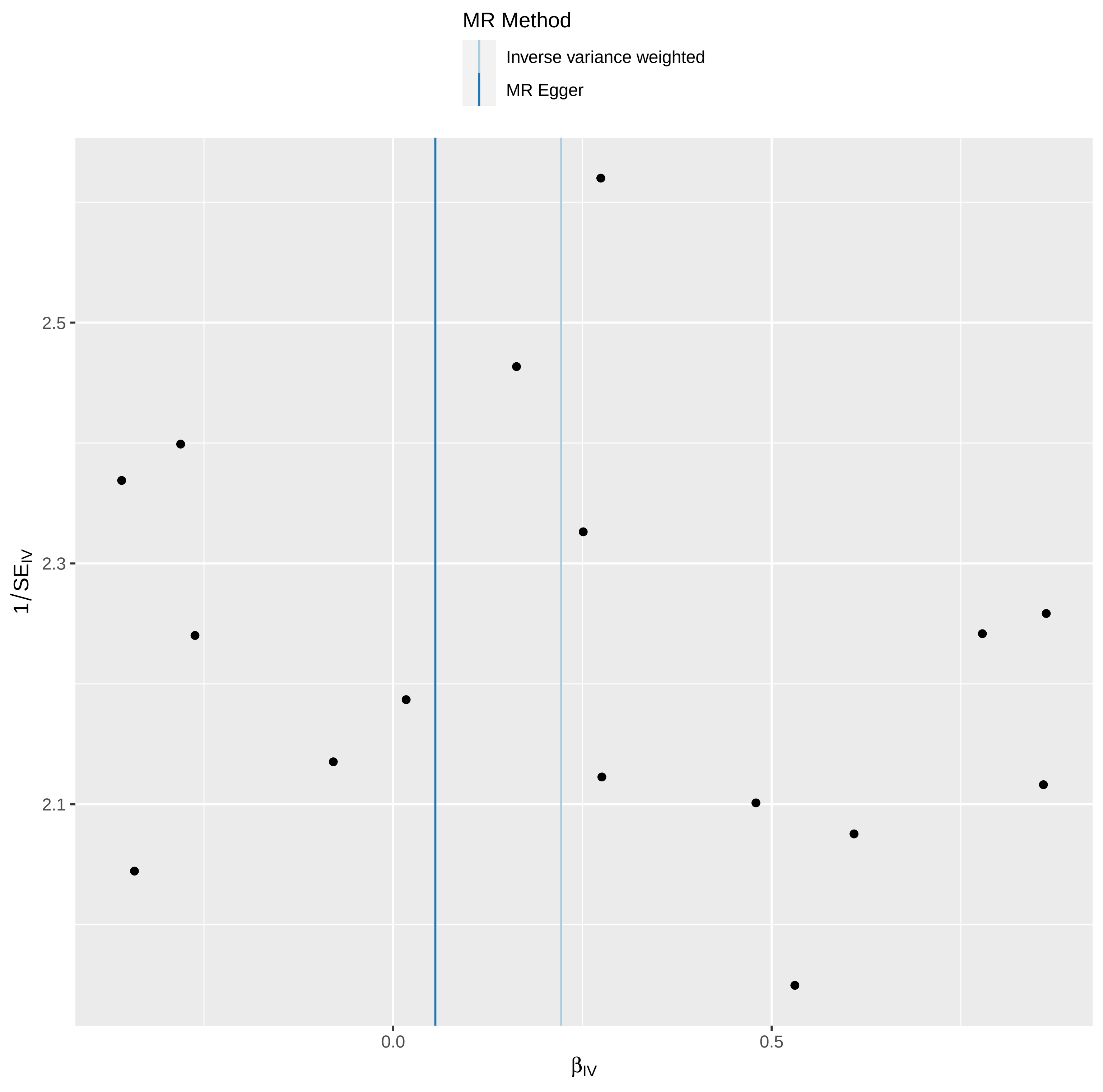

MR Method
Inverse variance weighted
MR Egger
2.5
2.3
2.1
1/SE_IV
0.0
0.5
β_IV

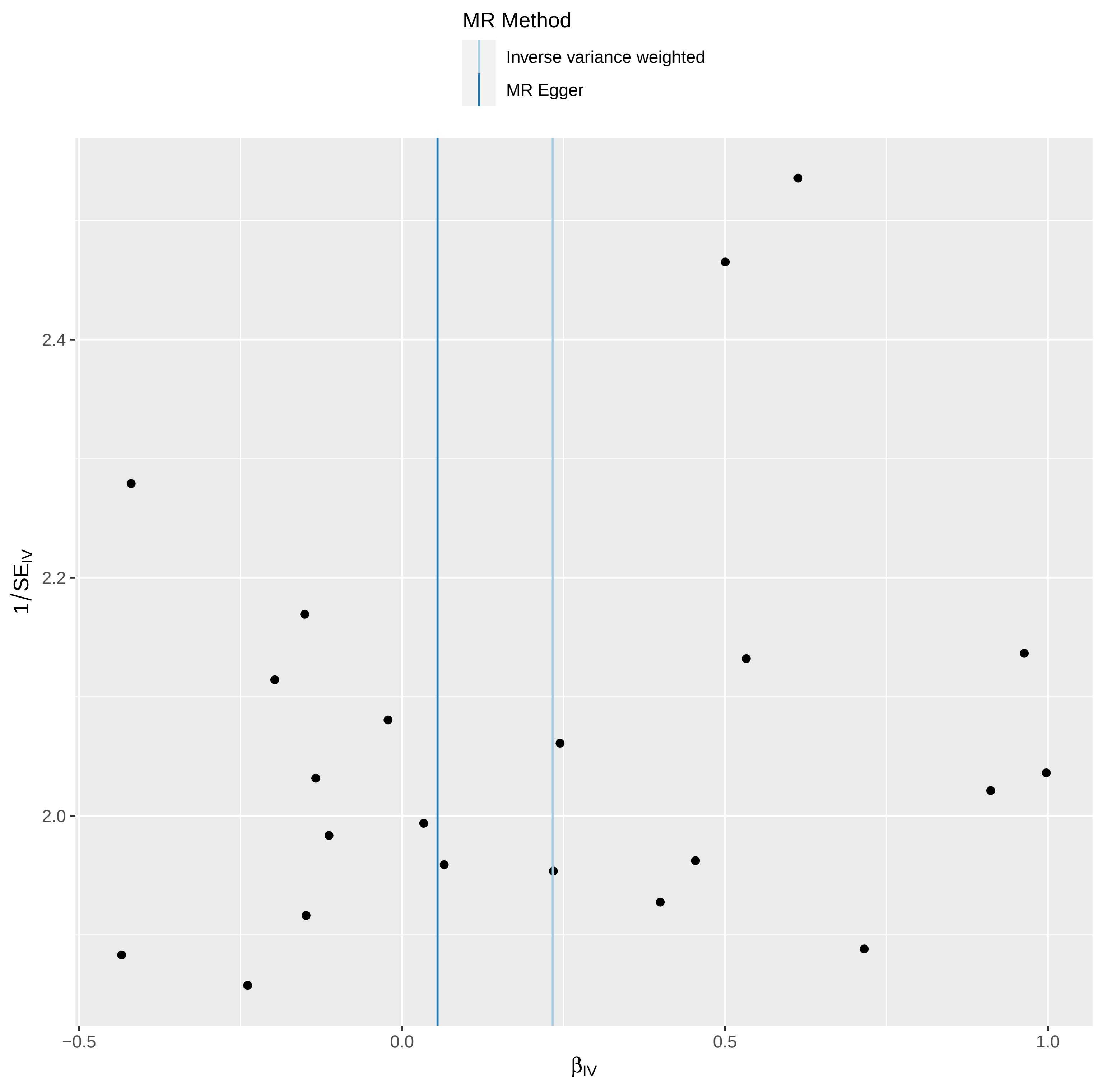

MR Method
Inverse variance weighted
MR Egger
2.4
2.2
2.0
1/SE$_{IV}$
−0.5
0.0
0.5
1.0
β$_{IV}$

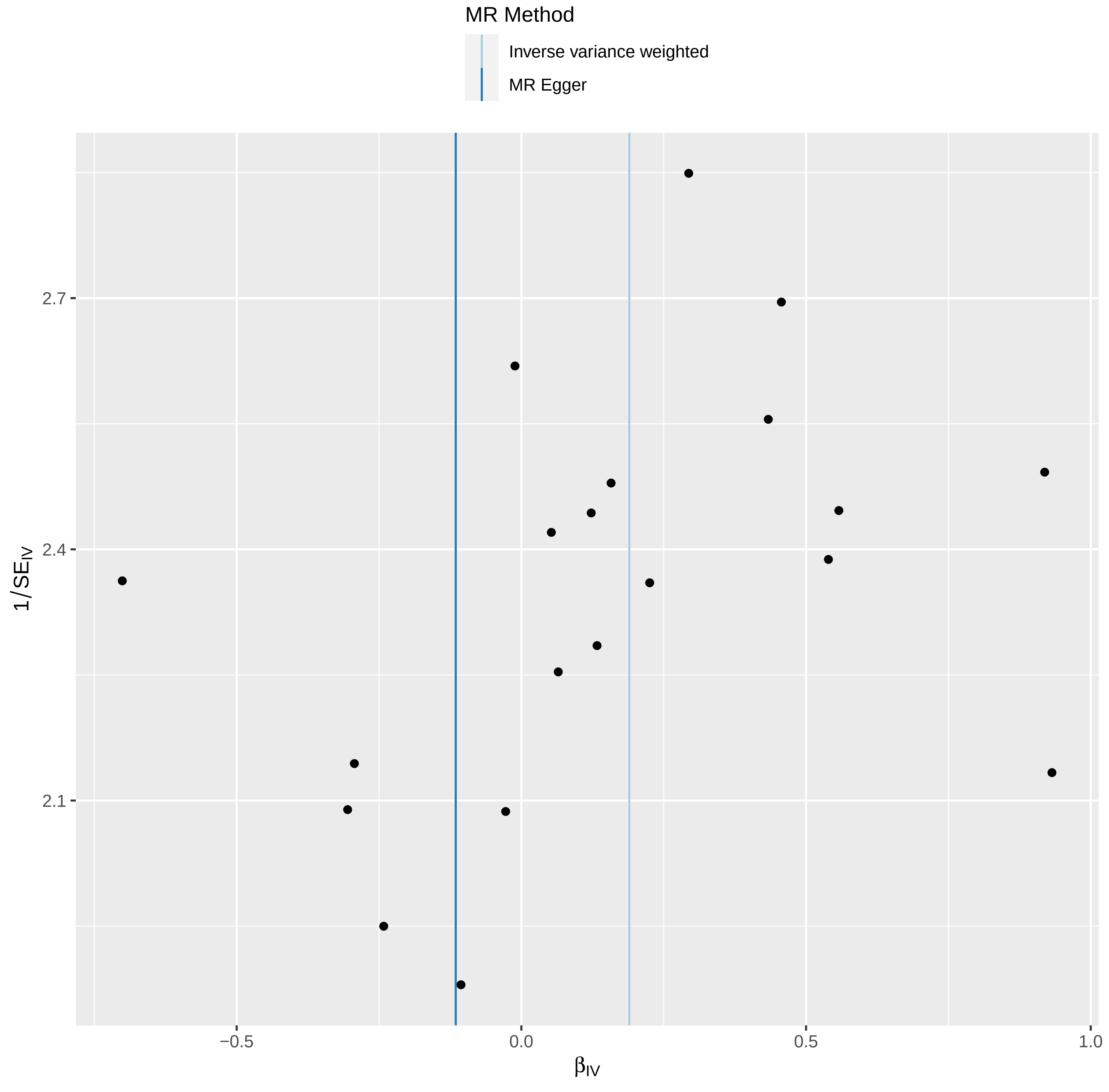

MR Method
Inverse variance weighted
MR Egger
2.7
2.4
2.1
1/SE$_{IV}$
−0.5
0.0
0.5
1.0
β$_{IV}$

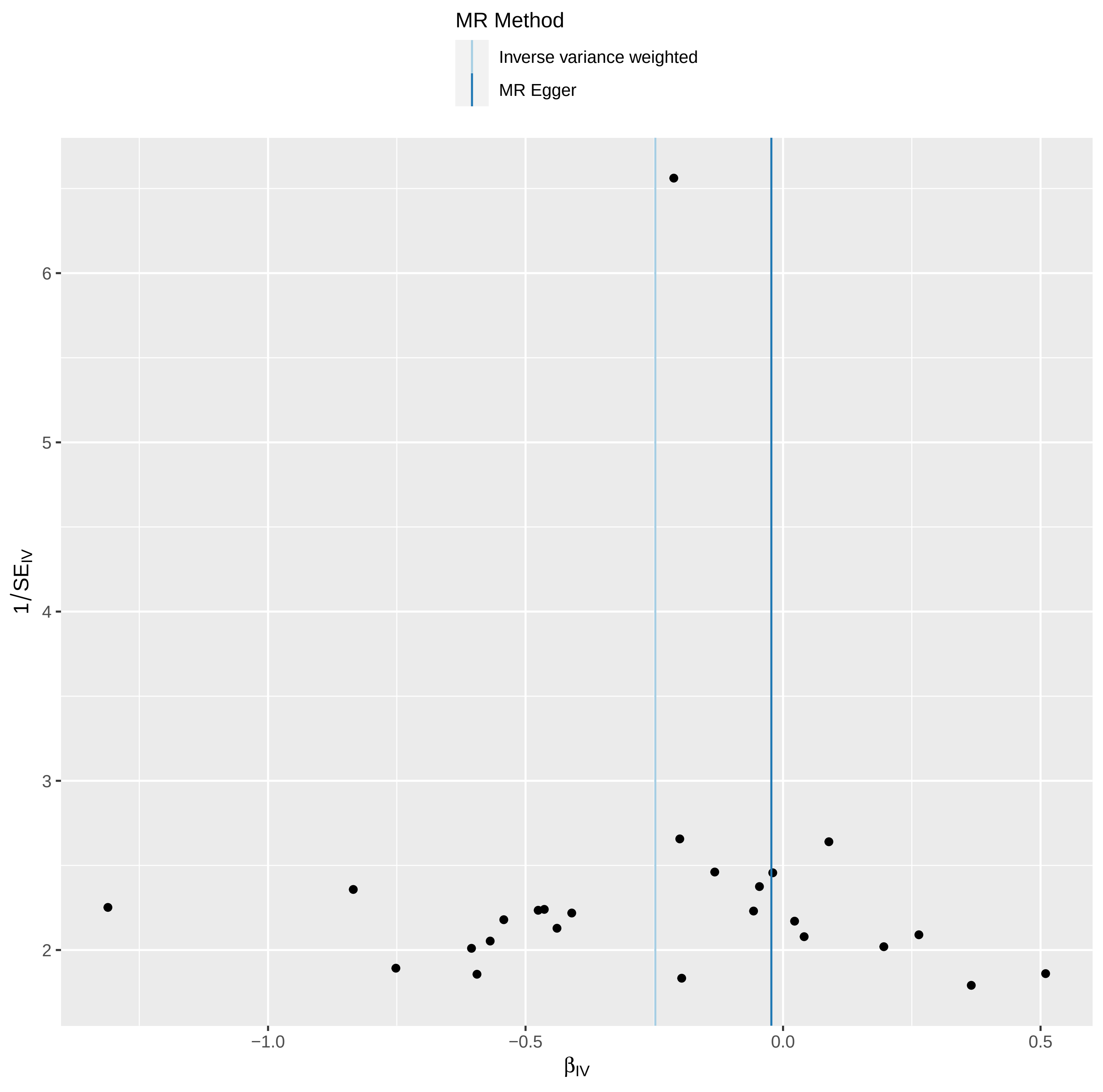

MR Method
Inverse variance weighted
MR Egger
6
5
4
3
2
1/SE$_{IV}$
−1.0
−0.5
0.0
0.5
β$_{IV}$

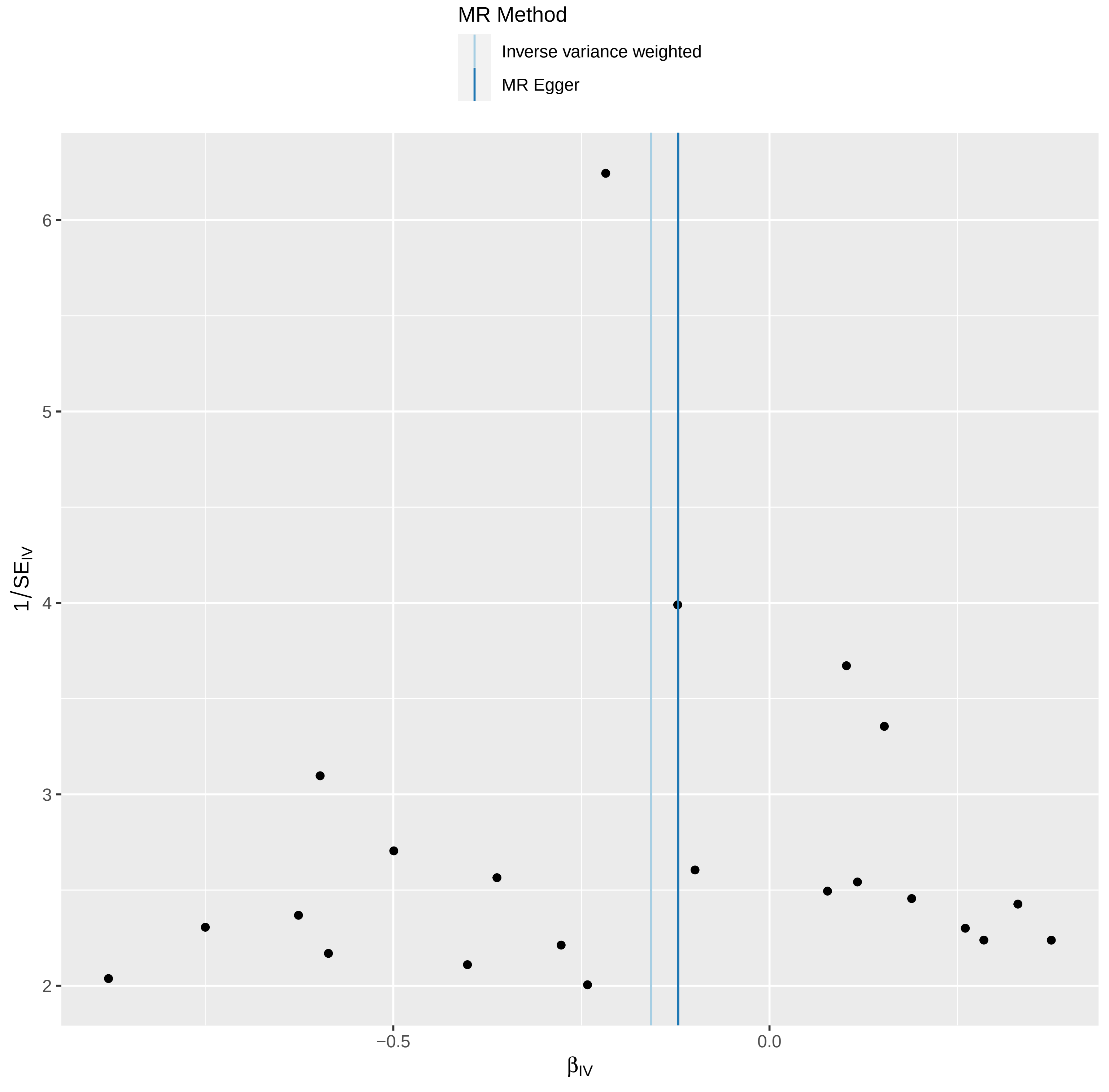

MR Method
Inverse variance weighted
MR Egger
6
5
4
3
2
$1/SE_{IV}$
−0.5
0.0
β_{IV}

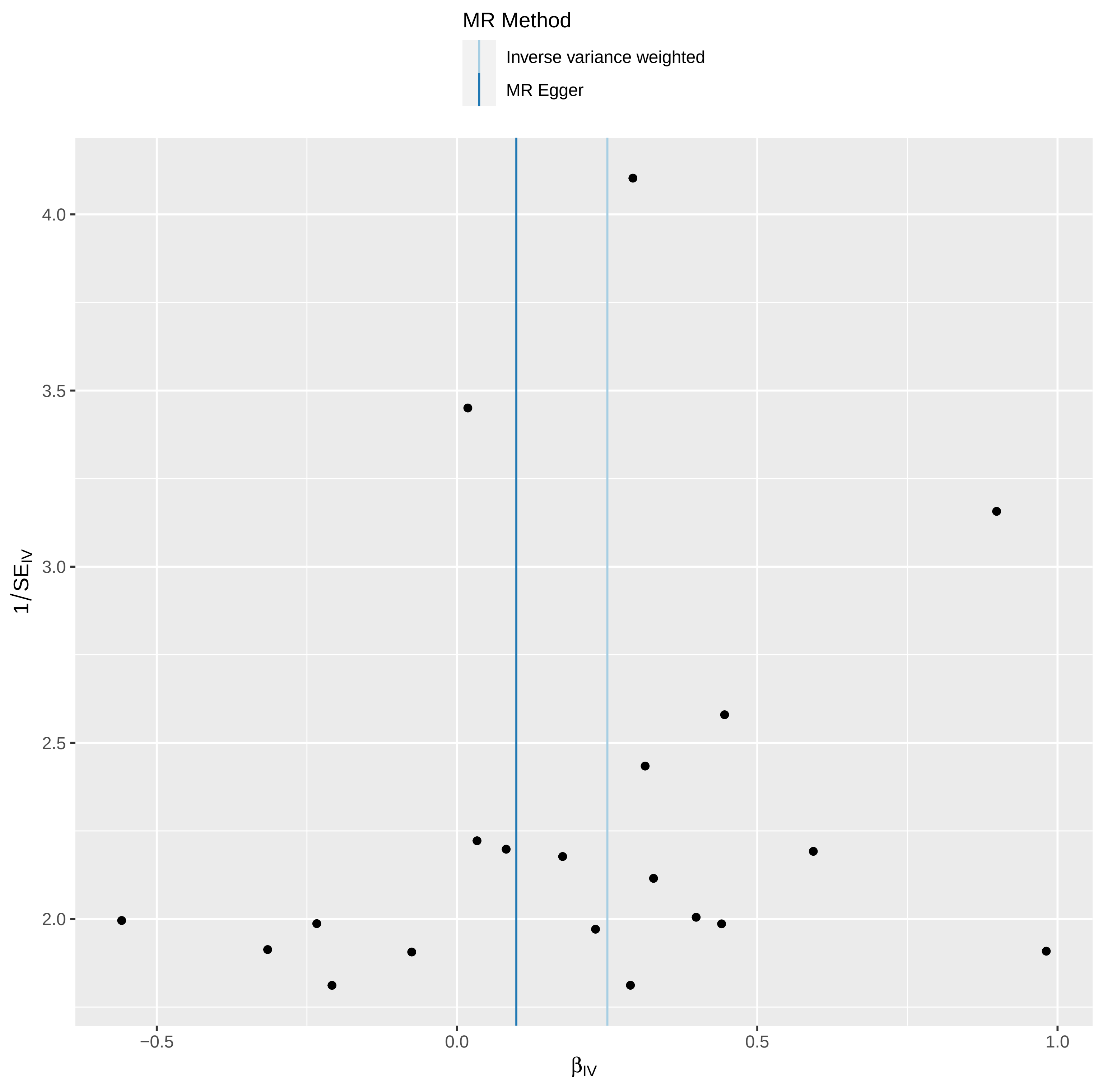

MR Method
Inverse variance weighted
MR Egger
4.0
3.5
3.0
2.5
2.0
1/SE_IV
−0.5
0.0
0.5
1.0
β_IV

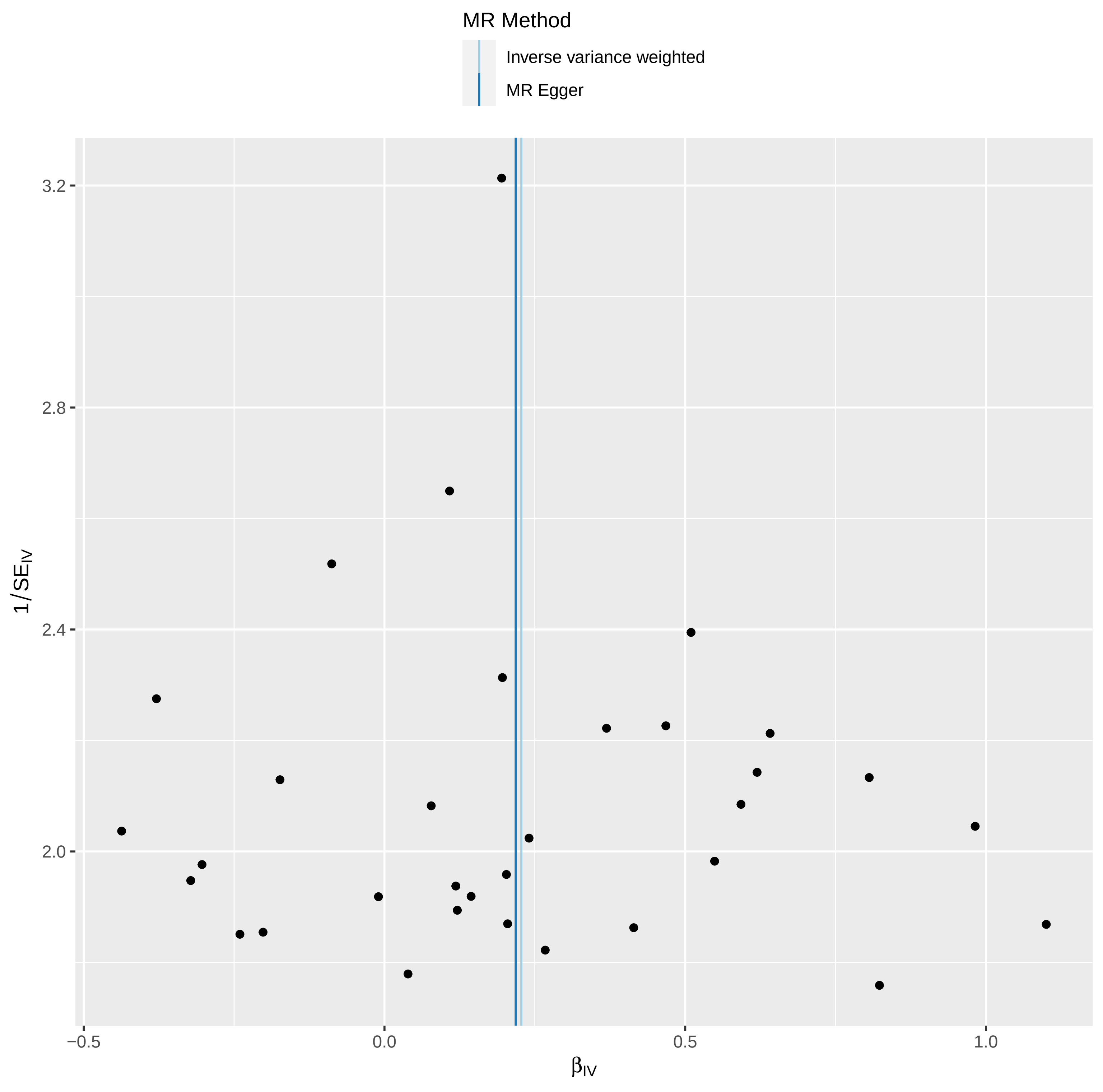

MR Method
Inverse variance weighted
MR Egger
3.2
2.8
2.4
2.0
$1/SE_{IV}$
−0.5
0.0
0.5
1.0
β_{IV}

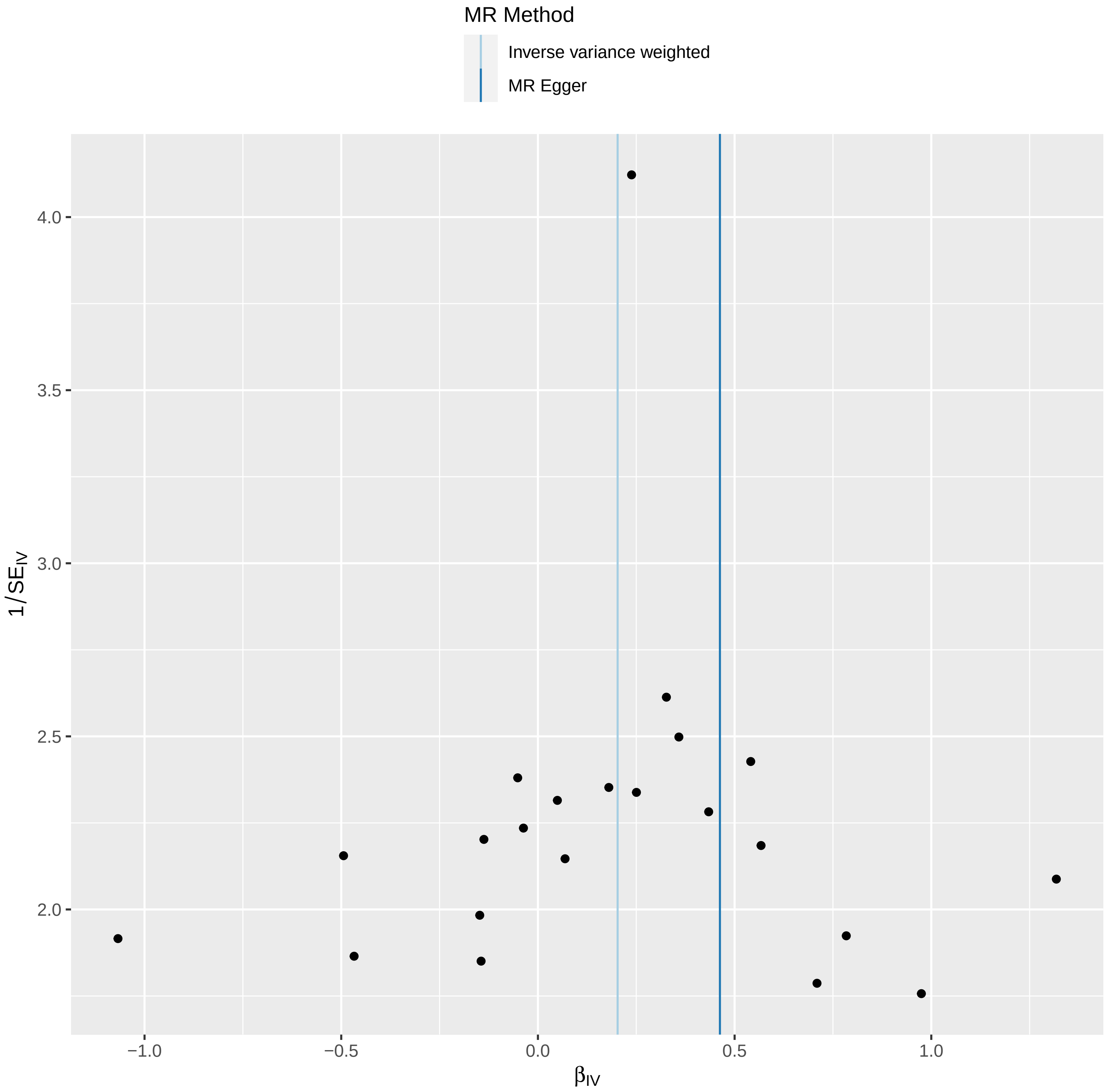

MR Method
Inverse variance weighted
MR Egger
1/SE_IV
β_IV
4.0
3.5
3.0
2.5
2.0
−1.0
−0.5
0.0
0.5
1.0

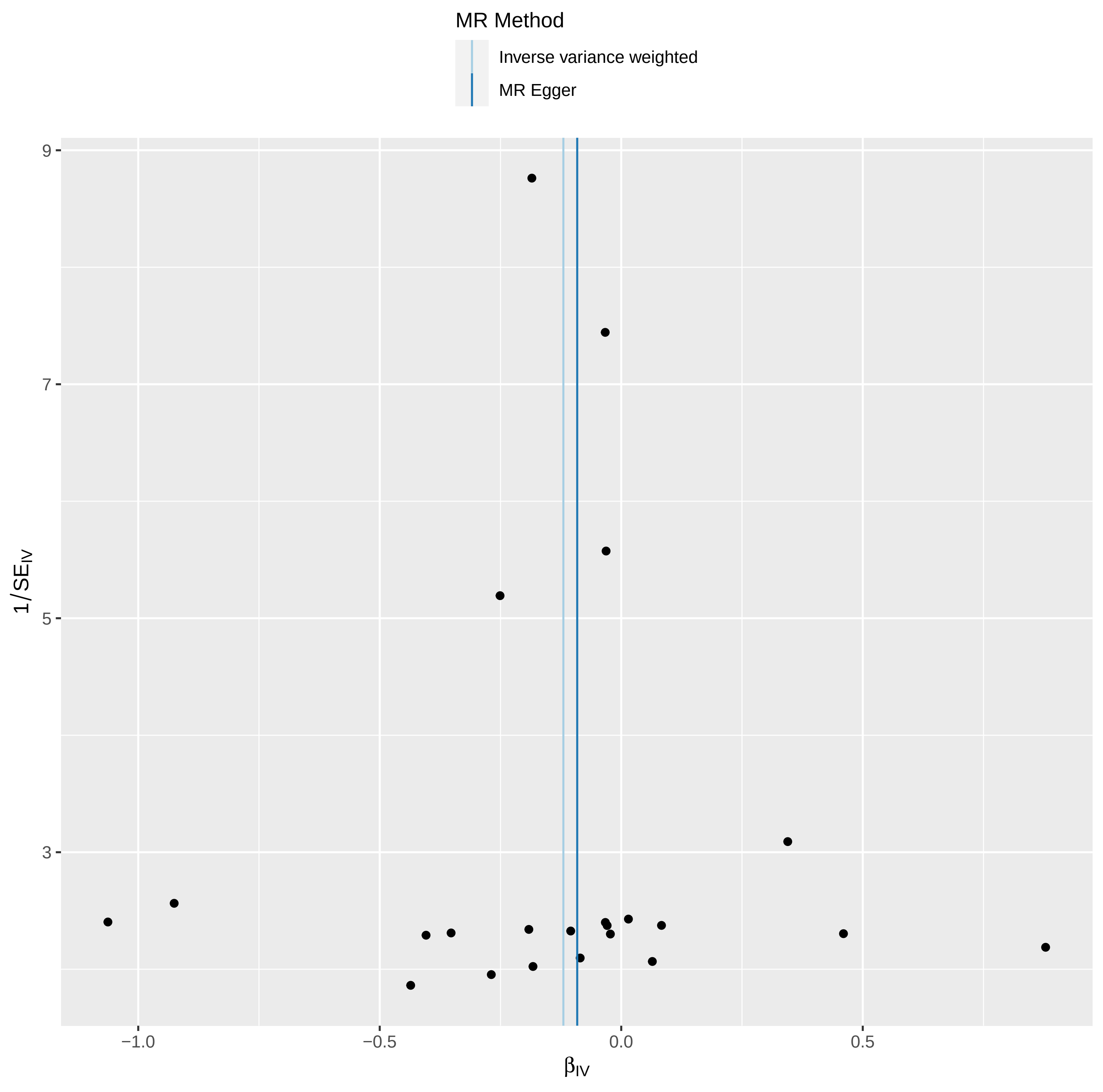

MR Method
Inverse variance weighted
MR Egger
9
7
5
3
1/SE_IV
−1.0
−0.5
0.0
0.5
β_IV

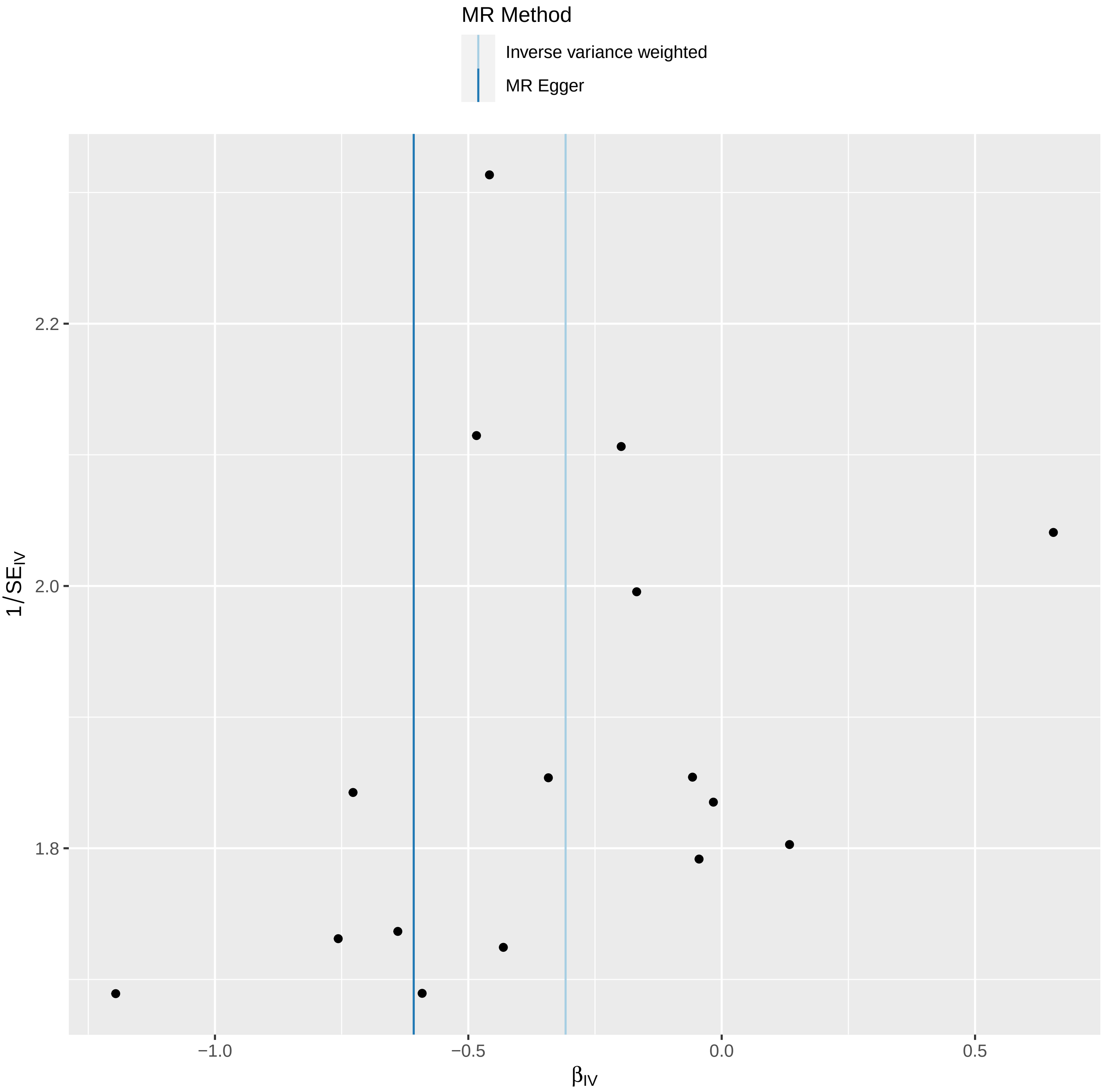

MR Method
Inverse variance weighted
MR Egger
2.2
2.0
1.8
1/SE$_{IV}$
−1.0
−0.5
0.0
0.5
β$_{IV}$

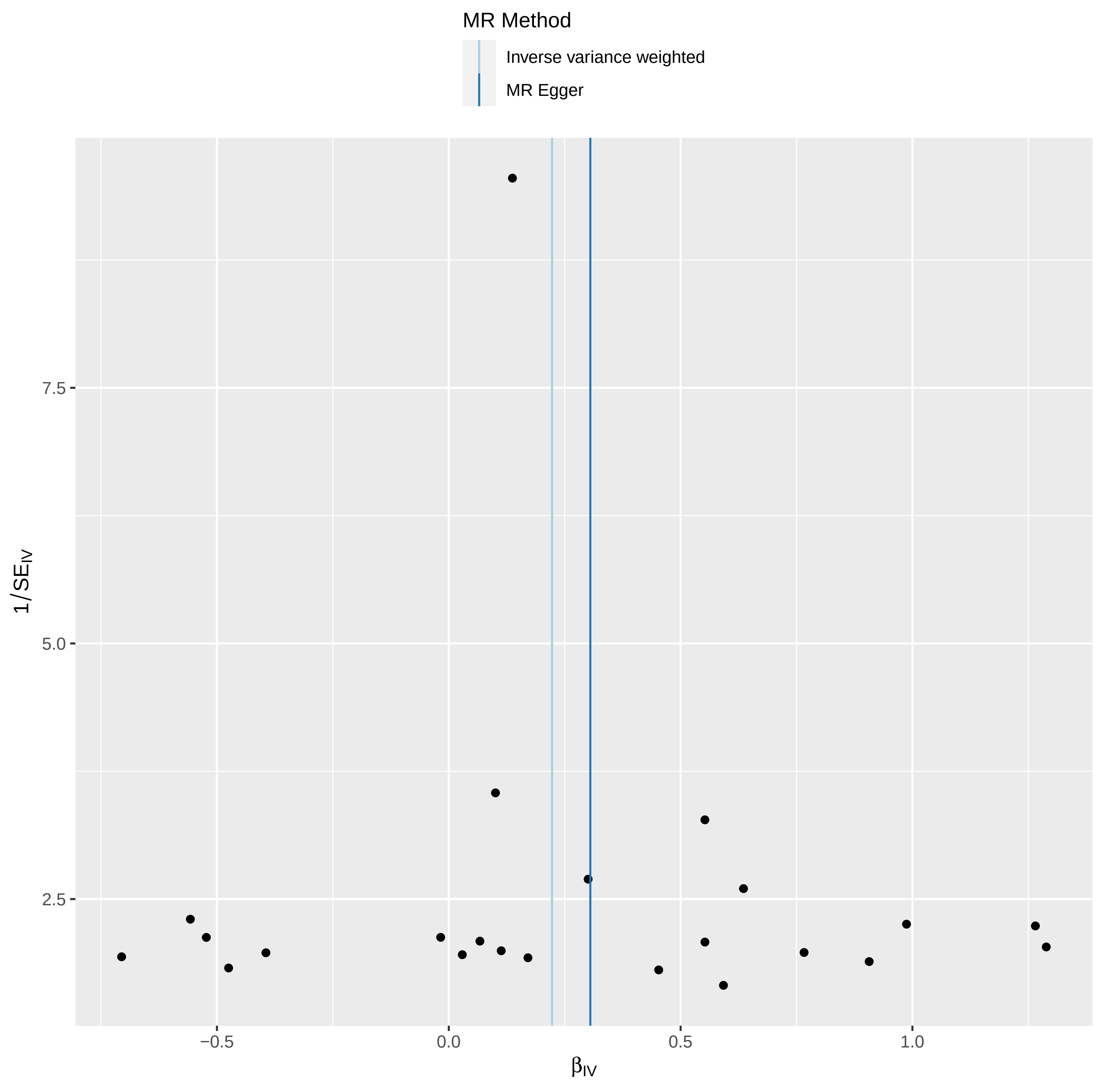
MR Method
Inverse variance weighted
MR Egger
7.5
5.0
2.5
1/SE_IV
−0.5
0.0
0.5
1.0
β_IV

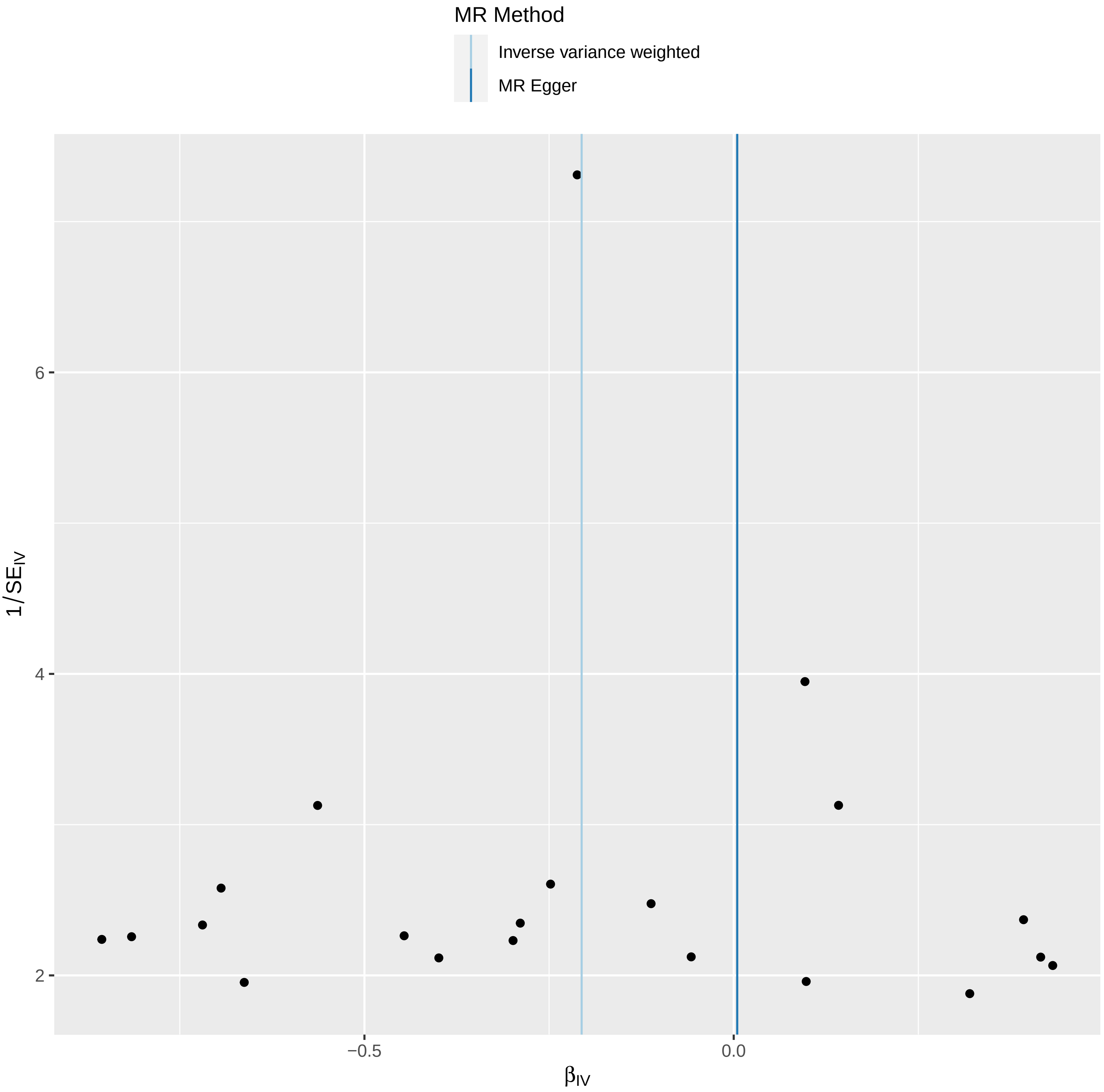

MR Method
Inverse variance weighted
MR Egger
6
4
2
$1/SE_{IV}$
−0.5
0.0
β_{IV}

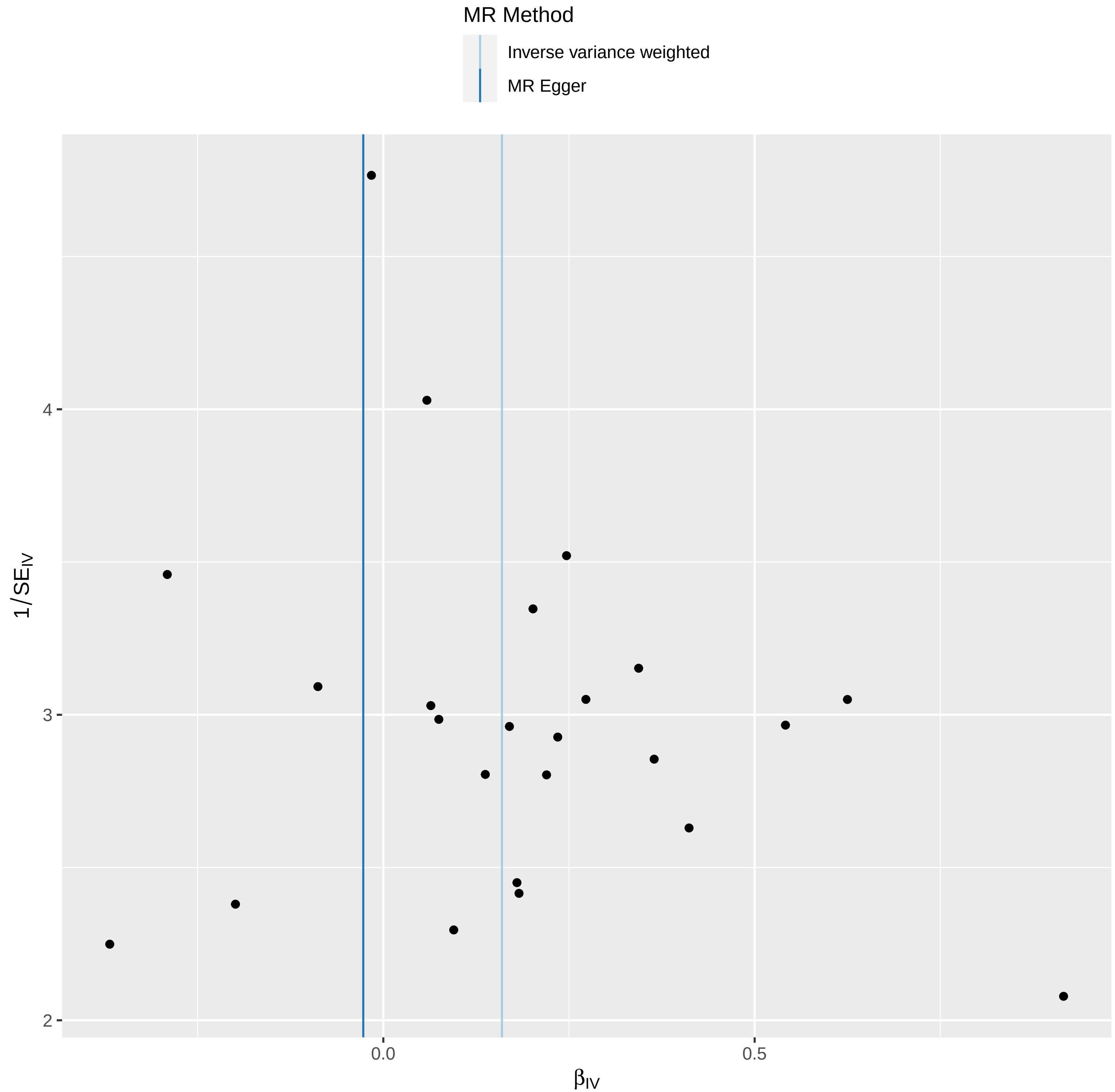
MR Method
Inverse variance weighted
MR Egger
4
3
2
0.0
0.5
$1/SE_{IV}$
β_{IV}

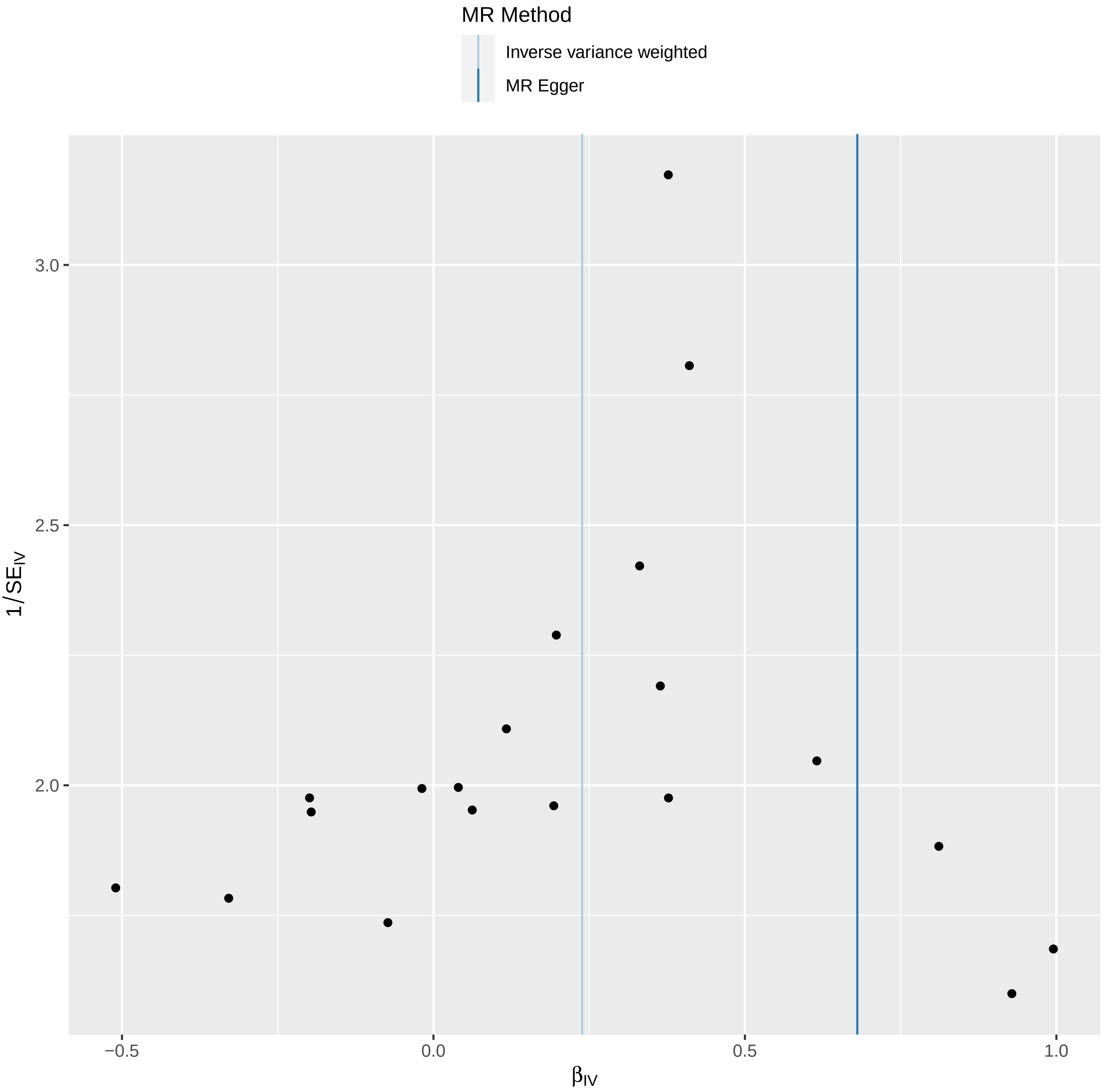

MR Method
Inverse variance weighted
MR Egger
3.0
2.5
2.0
1/SE_IV
−0.5
0.0
0.5
1.0
β_IV

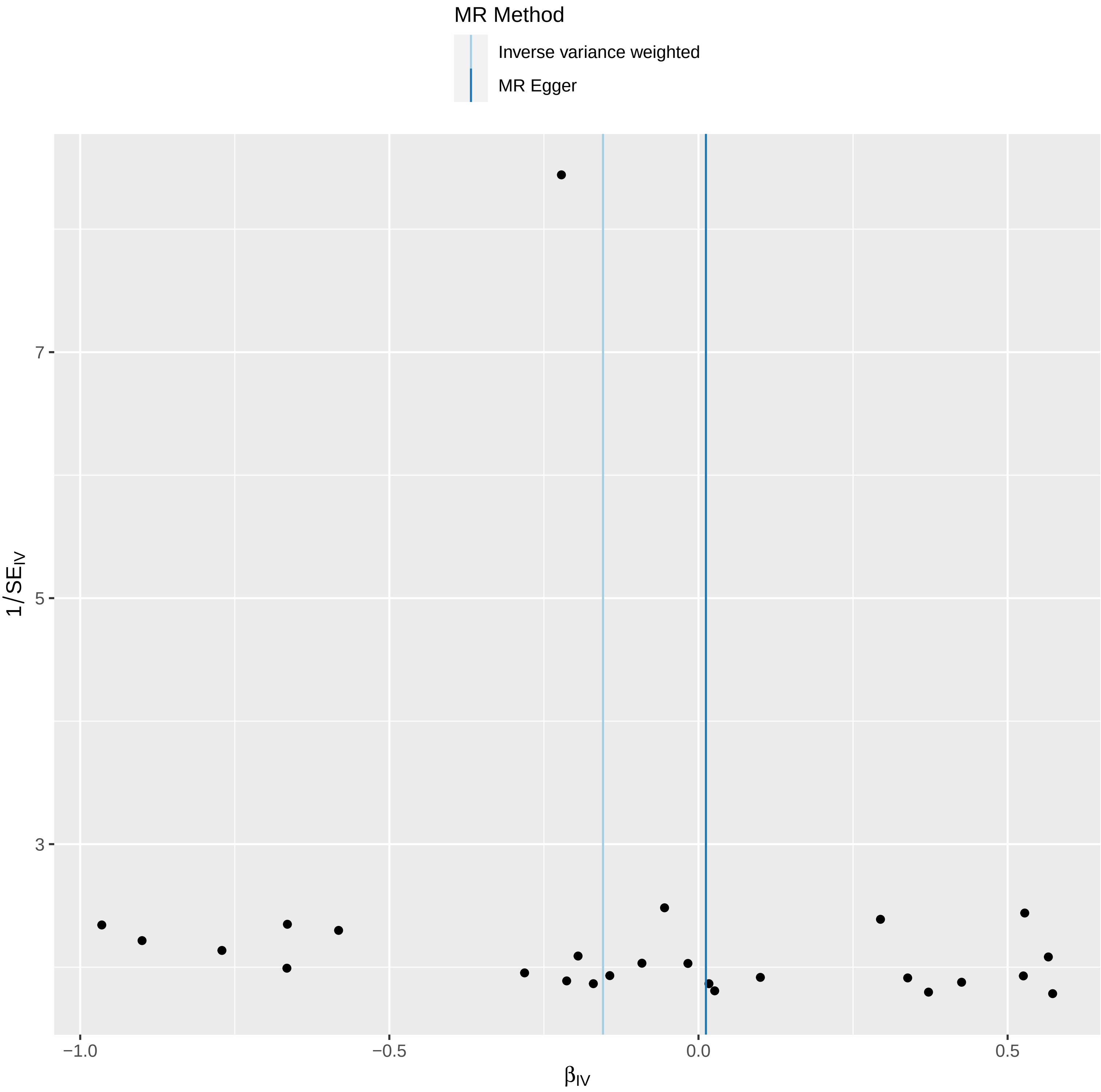

MR Method
Inverse variance weighted
MR Egger
7
5
3
1/SE_IV
−1.0
−0.5
0.0
0.5
β_IV

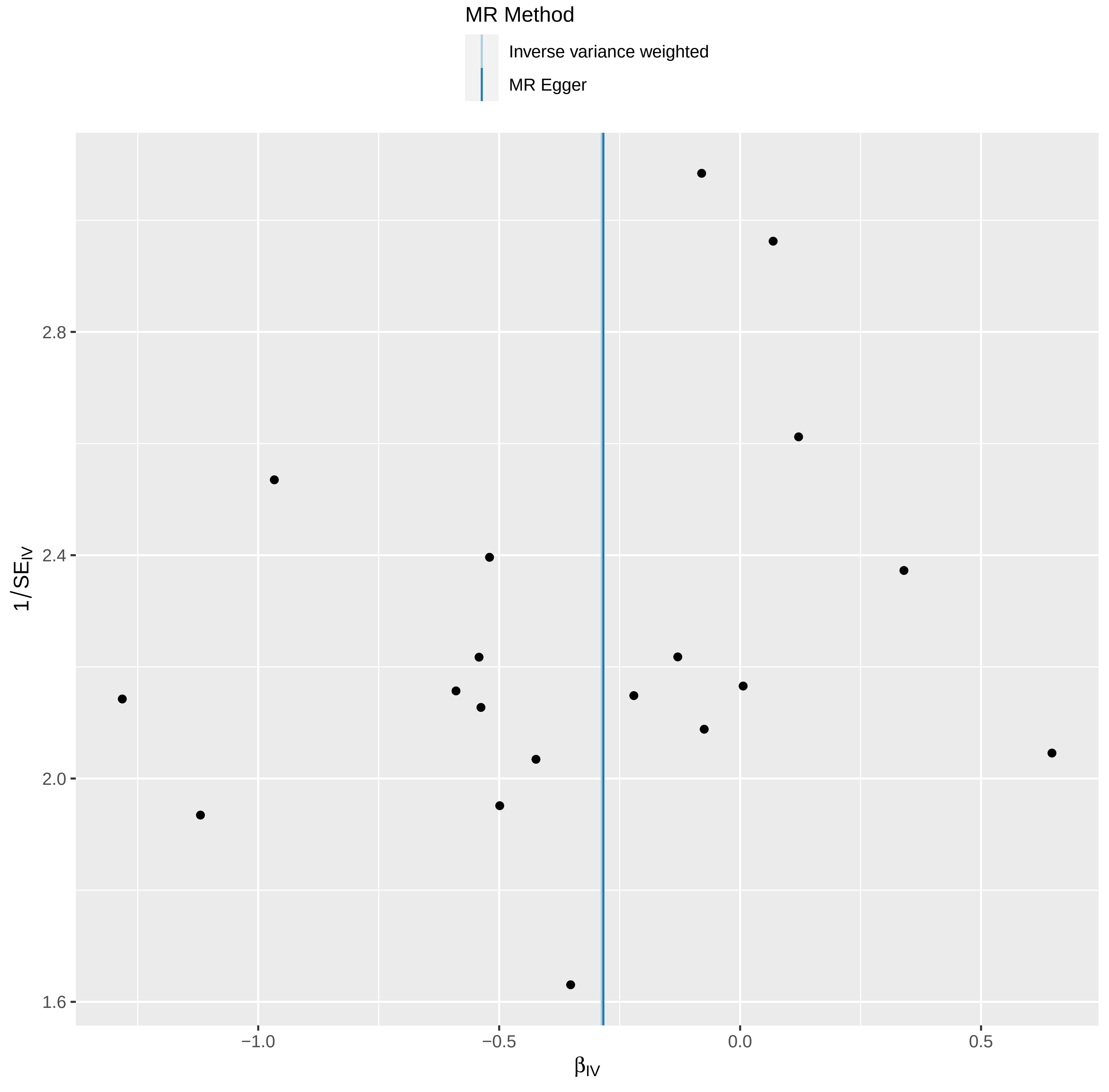

MR Method
Inverse variance weighted
MR Egger
2.8
2.4
2.0
1.6
$1/SE_{IV}$
−1.0
−0.5
0.0
0.5
β_{IV}

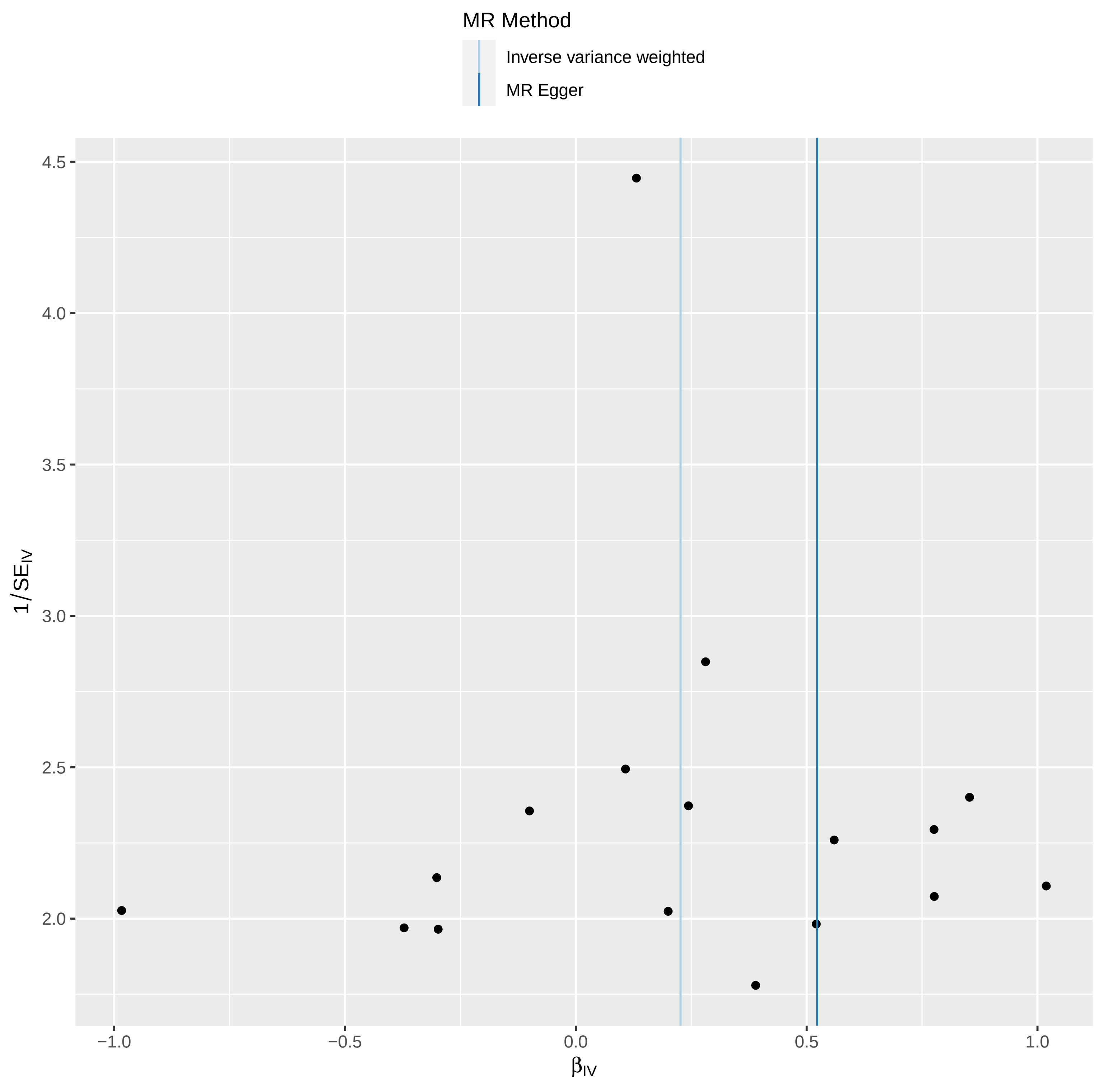

MR Method
Inverse variance weighted
MR Egger
4.5
4.0
3.5
3.0
2.5
2.0
1/SE$_{IV}$
−1.0
−0.5
0.0
0.5
1.0
β$_{IV}$

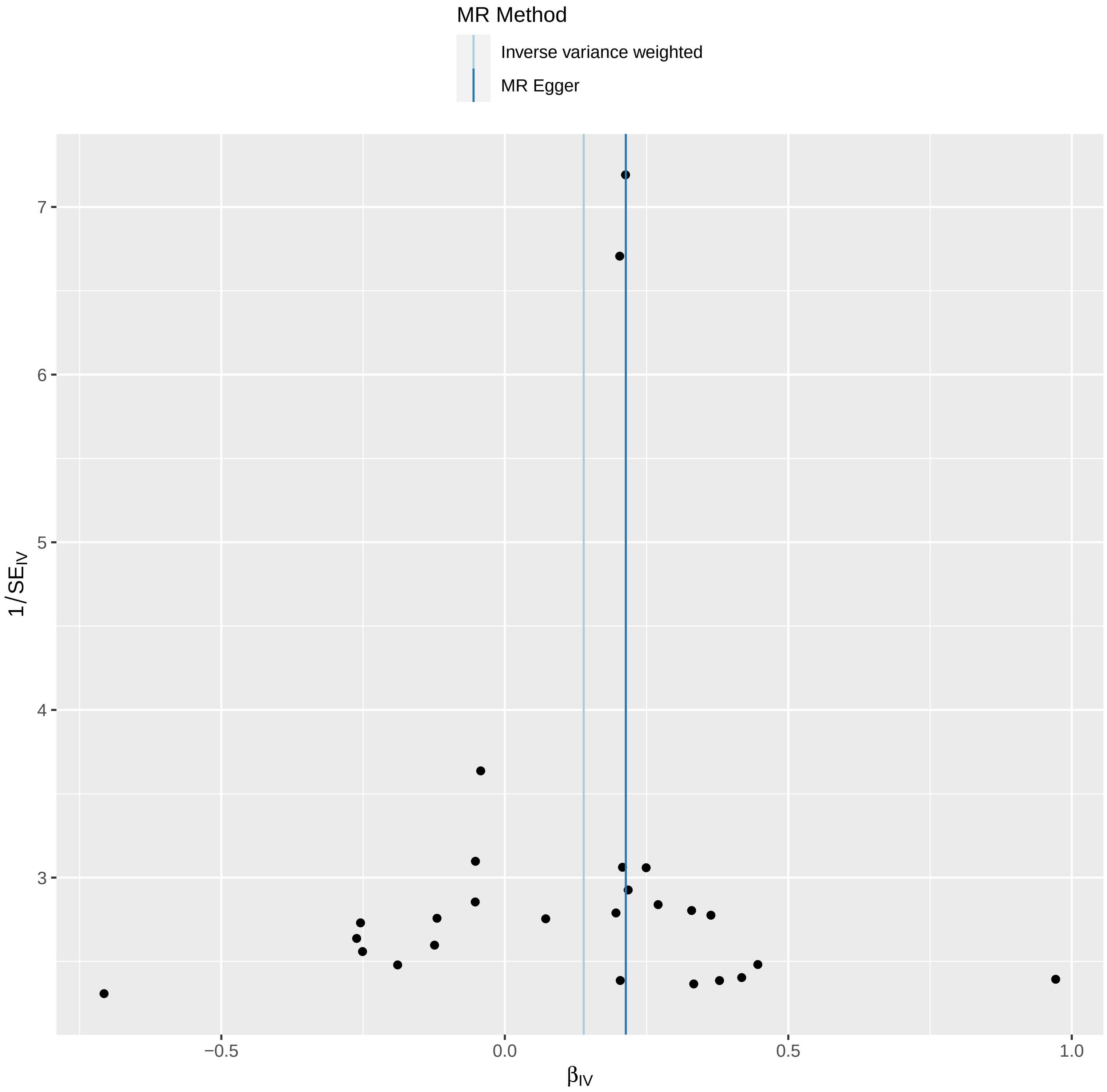

MR Method
Inverse variance weighted
MR Egger
7
6
5
4
3
1/SE_IV
−0.5
0.0
0.5
1.0
β_IV

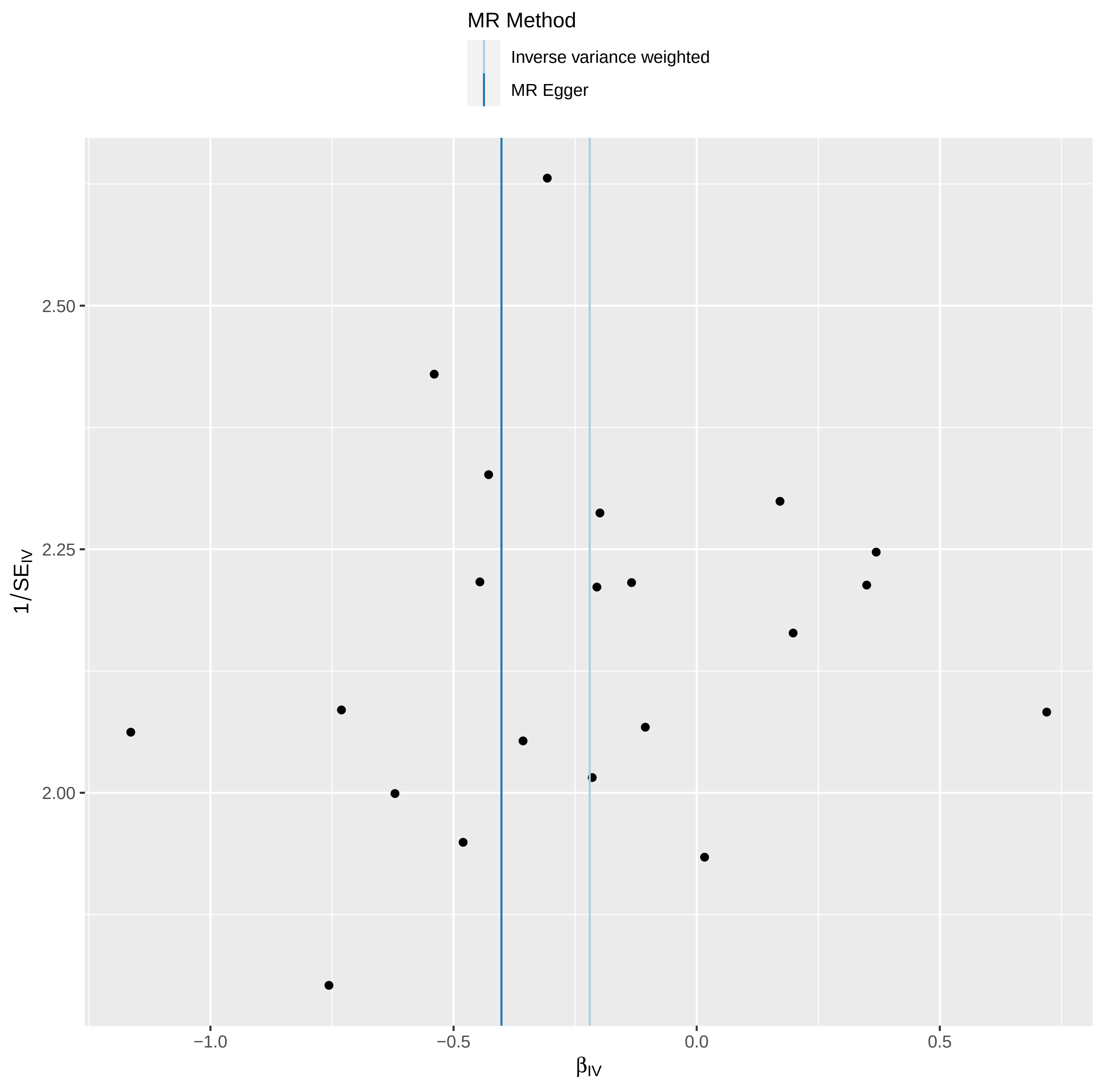

MR Method
Inverse variance weighted
MR Egger
2.50
2.25
2.00
1/SE$_{IV}$
−1.0
−0.5
0.0
0.5
β$_{IV}$
